# Supplementary material for: Impact of COVID-19 on the Health of the General and More Vulnerable Population and Its Determinants: Health Care and Social Survey–ESSOC, Study Protocol
Source: Int J Environ Res Public Health. 2021 Jul 31;18(15):8120. doi: 10.3390/ijerph18158120 (PMC8345631; doi:10.3390/ijerph18158120)
Supplement: Supplementary file 1 [file ijerph-18-08120-s001.zip › SupplementaryMaterial1_DataManagementPlanEnglish.pdf]

Impact of COVID-19 on the health of the general and more vulnerable population and its determinants: Health care and social survey-ESSOC, study protocol

Supplementary Material 1: Data Management Plan

Junta de Andalucía
Consejería de Salud y Familias
Escuela Andaluza de Salud Pública

SUPPLEMENTARY MATERIAL 1

DATA MANAGEMENT PLAN

The ESSOC is an activity included in the processing activities registry of the CSyF and is linked to the EAS, an official statistical operation included in the Andalusian Statistical and Cartographic Plan, with code 02.02.21. The IECA is responsible for the extraction of the random samples to contact by telephone in order to carry out the interviews from the BDLPA, whose content is the minimum required to be able to perform the interviews. The database (DB) developed with information obtained from the interviews is pseudonymized from that containing the personal data used to contact the interviewees by means of a unique key for each selected person.

The selected subjects receive a text message in which they are informed of the next interview call, the organization responsible for the interview (Andalusian School of Public Health, EASP), a telephone number with a 900 prefix for queries, and a web link to access more information. At the beginning of the interview, the interviewee is offered more information about the study and is requested to provide their verbal consent to conduct the interview. The interview is concluded by informing the interviewee of the likelihood of them receiving a new telephone contact for field control purposes or to obtain further measurements.

The information collected during the interview is linked to clinical, geographical, and auxiliary environmental information through the BPS, the geographical registries of the IECA, and the environmental registries of the Department of Agriculture, Livestock, Fisheries, and Sustainable Development of the Andalusian Regional Government. To this end, the CSyF issues a request to the IECA to obtain the Andalusian Unique Medical Record Number (NUHSA, Número Único de Historia de Salud de Andalucía) of the interviewees, as well as their postal addresses. Moreover, it requests the collection of clinical and environmental information to the SAS and the Department of Sustainable Development. The DB containing the clinical information of the interviewees and created from the BPS is pseudonymized from that containing the NUHSA and linked to the DB containing the information obtained from the interviews using the same unique key for each selected person.

It is guaranteed that the supplied data are protected by Statistical Secrecy and, therefore, that they are not misused, that they are processed anonymously and globally at all times, and that the information is disseminated at a provincial level with a sufficiently aggregated disaggregation to allow indirect identification. On the other hand, data disclosure is not foreseen in this study, except in those cases covered by Statistical Secrecy within the scope of the Andalusian Statistical and Cartographic System, which, in this case, corresponds to the IECA. The security measures are those provided for in Annex II (Security Measures) of Spanish Royal Decree 3/2010, of January 8th, which regulates the National Security Scheme in the context of Electronic Administration and are described in the documents that make up the data protection and information security policy of the IECA. Furthermore, the legal basis of the ESSOC are the provisions of the following articles of the General Data Protection Regulation (GDPR): 6.1.e) regarding the need to process data in order to accomplish a task carried out in the public interest or in the exercise of public powers vested in the data controller, and d), regarding the compulsory nature of such processing for the protection of the vital interests of the interested party or another natural person, as well as the specific applicable statistical regulations (Spanish Law 12/1989, of May 9th, on the Public Statistical Function; Spanish Law 4/2007, of April 4th, which amends Spanish Law 4/1989, of December 12th, on Statistics of the Autonomous Region of Andalusia, and approves the 2007–2010 Andalusian Statistics Plan, extended by virtue of Spanish Law 11/2010, of December 3rd, on fiscal measures for the reduction of public deficit and sustainability; and Spanish Decree 25/2020, of February 24th, approving the Statistical Cartographic Program of the Autonomous Region of Andalusia for the year 2020).

Regarding information management, the data are safeguarded at all times in servers of the EASP. The data recording and dumping process anonymously stores information, with the collected data being exclusively linked to a sample unit code. Access to the anonymized microdata is managed by EASP technicians through profiles boasting controlled permissions that allow for limiting, monitoring, and controlling access to the information. The results of this project are expected to be holistically beneficial to the general population, considering its socioeconomic and environmental context, as well as its evolution over several years since the beginning of the pandemic. Hence, the research team considers that the potential risk to the privacy of the study population is minimal compared with the potential benefit of its results. Furthermore, the data disclosure is carried out between official bodies within the Andalusian Public Health System and the Andalusian Regional Government itself, in the context of a research project that is exclusively in the public sphere and boasting a legitimate use of the BPS and the BDLPA as research infrastructures in accordance with the Spanish General Health Act (Ley General de Sanidad), the Spanish Law on Biomedical Research (Ley de Investigación Biomédica), and the Spanish General Public Health Act (Ley General de Salud Pública). Supervision of the data processing is assigned to the principal investigator, who is counseled by experts, and to the data protection officer of the project's coordinating center (EASP).

The IECA is responsible for the extraction of the random samples to contact by telephone in order to carry out the interview for all measurements, which, with the exception of the first one due to being carried out by the IECA itself, are subsequently transferred to the Health Care Statistics Service (Servicio de Estadísticas Sanitarias) of the CSyF, which is responsible for processing the application and, once received, for forwarding the extracted sample to the EASP, which, in turn, sends it to the company contracted for the fieldwork (file number of the EASP contract: 2019/00060; bid code: 2019-00000117908). These samples are sent through web platform "Consigna" of the Andalusian Regional Government in a compressed format requiring a password that is sent via a different channel than that used to notify their availability. The personal data content of these databases is the minimum necessary to be able to perform the telephone interviews; that is, they only contain the name, surname, and telephone number of the interviewees. In addition, the database developed with information obtained from the interviews is pseudonymized from the database containing the personal data used to contact the interviewees by means of a unique key for each selected person.

The interviewees selected for the first time receive a first text message from the IECA (for the 1st measurement) and the EASP (for the subsequent measurements) informing them of the next call that will be carried out to perform the interview. This text also describes the organization responsible for the interview (IECA for the 1st measurement and EASP for the subsequent ones), a free telephone number with a 900 prefix for any queries, and a web link to obtain more information about the survey. At the beginning of the telephone interview, the interviewee is offered more information about the survey (purpose, responsible organization, Internet website, telephone number with a 900 prefix for any queries, confidentiality aspects, and processing of the collected information) and is requested their verbal consent to conduct the interview. The interview is concluded by informing the interviewee of the likelihood of them receiving a new telephone contact, whether it be for field control tasks or for the conduct of subsequent survey-related measurements.

Those people who had already been interviewed for a previous measurement are sent a survey reminder text message that notifies them of the subsequent conduct of the telephone interview. At the beginning of each interview, they are provided with the same information that they were given in the previous case and are asked to provide their consent again in order to conduct the interview. Given the short time available for the fieldwork, a text message was not sent for the 2nd measurement. Regarding the subjects aged 16 and 17 years old selected for the survey, as they are over the age of 14, the interview is carried out on the basis of their own consents (article 7 of Spanish Organic Law 3/2018, of December 5th, on Data Protection).

As indicated in the "Variables" section, in addition to that collected during the actual telephone interview, clinical and auxiliary environmental information will also be obtained. Thus, on the one hand, in order to link the information obtained during the telephone interview with the clinical information extracted from the BPS, the Health Care of Statistics Service of the CSyF must issue another request to the IECA to obtain the NUHSA of the interviewees. Subsequently, a request is made to the General

Directorate of Health Care and Health Outcomes (DGAS, Dirección General de Asistencia Sanitaria y Resultados en Salud) of the Andalusian Health Service to obtain the clinical information of the selected sample subjects. The database containing the clinical information of the interviewees and created from the BPS is pseudonymized from the database containing the NUHSA and linked to the database containing the information obtained from the interviews using the same unique key for each selected person. On the other hand, the postal addresses of the interviewees are requested from the IECA in order to link the information obtained from the interviews with that of the geographical registries of the IECA and the environmental registries of the CSyF and the Department of Agriculture, Livestock, Fisheries, and Sustainable Development of the Andalusian Regional Government. The same procedure and processing described above is followed for this purpose. Concerning the transfer of these data by the responsible entities (DGAS and IECA) to the research team, the use of health data for research is contemplated without the need to request the consent of the person concerned, given that in this particular case, we are dealing with large databases for which obtaining this sort of consent would require a disproportionate effort with respect to the risks that it could entail for the safeguarding of the subject's privacy. Furthermore, the use of these data is of public interest in accordance with the Spanish General Health Act, the Spanish Law on Biomedical Research, and the Spanish General Public Health Act.

Below we present the DPIA carried out with the tool (https://gestiona.aepd.es/)) of the Spanish Data Protection Agency (AEPD, Agencia Española de Protección de Datos) in accordance with the specific adaptation of this methodology to research projects in the health care sector, as well as generic documents "Practical Guideline for Data Protection Impact Assessments (Guía Práctica para las Evaluaciones de Impacto en la Protección de los Datos)" of the AEPD, subject to the GDPR, and the "Horizon 2020 Program Guidance: How to complete your ethics self-assessment" (section 4 on personal data). The need for a DPIA was confirmed in the first place (Table S1). Then, the data lifecycle was defined (Table S2), and the need and proportionality of the processing were analyzed, (Table S3). And, finally, a risk assessment and action plan were developed (Table S4). Thus, the outcome of the DPIA is that the residual risk level is deemed acceptable.

Table S1. Analysis of the need for a Personal Data Protection Impact Assessment

QUESTION	YES/NO	RATIONALE
**Is the processing to be analyzed included in the list of types of data processing published by the AEPD that require a DPIA?**	NO	
**Will special data categories be processed at a large scale?**	YES	Following the guidelines of the GROUP FOR THE PROTECTION OF INDIVIDUALS WITH REGARD TO THE PROCESSING OF PERSONAL DATA, created by Directive 95/46/EC of the European Parliament and the Council, dated 24 October 1995 (wp243), it is difficult to establish whether the data will be processed at a large scale; however, given the sensitive nature of the data, we consider that it will
**Will special data categories be processed?**	YES	Health-related data will be processed, as data from the Andalusian Population Health Database will be linked
**Can the habits, behaviors, preferences, likes, interests, etc. of identified or identifiable people be determined as a result of the processing operations?**	YES	The objective of the data processing is to describe the situation and evolution of the general Andalusian population with respect to their health status, social determinants, living conditions, and cohabitation
**The evaluated processing operations are covered by adherence to a code of conduct within the limits set by the GDPR**	YES	The legal basis of this survey are the provisions of the following articles of the GDPR: 6.1.e) regarding the need to process data in order to accomplish a task carried out in the public interest or in the exercise of public powers vested in the data controller, and d), regarding the compulsory nature of such processing for the protection of the vital interests of the interested party or another natural person, as well as the specific applicable statistical regulations (Spanish Law 12/1989, of May 9th, on the Public Statistical Function; Spanish Law 4/2007, of April 4th, which amends Spanish Law 4/1989, of December 12th, on Statistics of the Autonomous Region of Andalusia, and approves the 2007–2010 Andalusian Statistics Plan, extended by virtue of Spanish Law 11/2010, of December 3rd, on fiscal measures for the reduction of public deficit and sustainability; and Spanish Decree 25/2020, of February 24th, approving the Statistical Cartographic Program of the Autonomous Region of Andalusia for the year 2020)
**Will personal data be processed for profiling, categorization/segmentation, rating/scoring, or decision-making?**	YES	Segmentation is essential to obtain proper estimates
**Will the processing involve data of minors subject to Data Protection (14 years according to the Spanish Organic Law on Personal Data**	NO	Minors are expressly excluded from the sample

QUESTION	YES/NO	RATIONALE
**Protection and the Guarantee of Digital Rights [LOPDGDD, *Ley Orgánica de Protección de Datos Personales y Garantía de los Derechos Digitales*])?**		
**Does the purpose of the processing involve the specific use of data of disabled people or any other particularly vulnerable population group?**	YES	As they are not excluded, they could be included in the selected sample
**Are non-irretrievably anonymized personal data used?**	YES	The database containing the clinical information of the interviewees and created from the BPS is pseudonymized from the database containing the NUHSA and linked to the database containing the information obtained from the interviews using the same unique key for each selected person
**Is the legal basis for the processing the subjects' consent?**	YES	Consent is requested from the person to be interviewed at the beginning of the telephone interview
**Will the data processing result in making decisions that could significantly affect or harm the data subjects in any way?**	NO	No decisions will be made on the basis of the data processing
**Are customer data processed to perform bad debt management tasks or using external files such as those of the National Association of Financial Institutions (ASNEF, *Asociación Nacional de Entidades Financieras*) or the Central Risk Information Center of the Bank of Spain (CIRBE, *Central de Información de Riesgos del Banco de España*)**	NO	No such data will be processed
**Will data relating to large-scale systematic observations of publicly accessible areas be processed?**	NO	No such data will be processed
**Will the processing involve a large number of persons (beyond those necessary to carry out the processing) having access to the processed personal data? For example, a department not involved in the processing**	NO	The research group is small, and all its members are identified and bound to statistical secrecy, most of them being registered in the Andalusian Registry of Statistical Agents (*Registro de Agentes Estadísticos de Andalucía*)
**In carrying out this processing, will datasets used by other data controllers whose purposes differ significantly from the interested party's expectations be combined?**	YES	The survey data are collected precisely for such purpose, but the BPS data are not. In this case, the principles of the GDPR of the European Parliament on the use of data for purposes other than those for which it was collected in the field of research are followed

QUESTION	YES/NO	RATIONALE
**Is the use of technologies that may be perceived as immature, newly developed or commercialized, or with a high risk of unauthorized access anticipated?**	NO	None of the technologies used to collect or process the information can be considered newly developed. In fact, other than a questionnaire administered by telephone and the precise software used for the analyses, no other technologies will be used in this study
**Will data transfers be made to other entities, whether belonging to the same group or external suppliers (if so, please specify which)?**	NO	Data disclosures to third parties are not anticipated. All data processing activities will be carried out by members of the research group belonging to the IECA, the EASP, the CSyF, and the SAS
**Will international data transfers be made to countries outside the European Union that do not implement personal data protection measures similar to those established by the Supervisory Authority (if so, please specify which ones)?**	NO	No international data transfers will be made
**Have any of the processing tasks been delegated to an external supplier outside the entity (if so, please specify which)?**	NO	All data processing activities will be carried out by members of the research group belonging to the IECA, the EASP, the CSyF, and the SAS. Some of the data processing tasks (e.g., calibration or spatio-temporal modeling) will be delegated to members of the research team of the Universities of Granada and Girona, although these tasks will be performed on previously anonymized data; therefore, this processing will always remain within the scope of the project
**Is this processing similar to any other processing for which a DPIA was required?**	NO	No link has been made between data from previous surveys and the Population Health Database
**Regardless of the questions contained in this form, is the analysis of the potential risks to personal data throughout the processing lifecycle (collection, storage/categorization, use/processing, and destruction) deemed advisable?**	YES	Given the sensitive nature of the data, a risk analysis has been considered

QUESTION	YES/NO	RATIONALE
**Does the processing have its legal basis in the European Union or a Member State Law, and has the DPIA already been carried out in this context?**	YES	The legal basis of this survey are the provisions of the following articles of the GDPR: 6.1.e) regarding the need to process data in order to accomplish a task carried out in the public interest or in the exercise of public powers vested in the data controller, and d), regarding the compulsory nature of such processing for the protection of the vital interests of the interested party or another natural person, as well as the specific applicable statistical regulations (Spanish Law 12/1989, of May 9th, on the Public Statistical Function; Spanish Law 4/2007, of April 4th, which amends Spanish Law 4/1989, of December 12th, on Statistics of the Autonomous Region of Andalusia, and approves the 2007–2010 Andalusian Statistics Plan, extended by virtue of Spanish Law 11/2010, of December 3rd, on fiscal measures for the reduction of public deficit and sustainability; and Spanish Decree 25/2020, of February 24th, approving the Statistical Cartographic Program of the Autonomous Region of Andalusia for the year 2020)
**Other reasons**	NO	

Table S2. Data lifecycle

<table>
<tr><th rowspan="2">ELEMENTS</th><th colspan="5">STAGES</th></tr>
<tr><th>Data capture</th><th>Categorization / storage</th><th>Processing / use</th><th>Disclosure or transfer of the data to a third party for their processing</th><th>Destruction</th></tr>
<tr><td>Process activities</td><td>• Sample extraction (people to be interviewed)
• Telephone interviews
• Request of the contact and clinical data for each measurement to those responsible for the BDLPA and BPS files
• Telephone interviews</td><td>• Receipt and checking of the selected samples
• Data recording in the IECA and EASP servers
• Field preparation for the interviews</td><td>• Fieldwork control and monitoring
• Data recording, coding, and debugging
• Data anonymization
• Data processing and analysis</td><td>• Processing performed by members of the research group of the IECA, EASP, CSyF, and SAS
• Certain tasks delegated to members of the UGR and UdG research team on anonymized data</td><td>Deletion of data in systems or files</td></tr>
<tr><td>Processed data</td><td colspan="3">• Longitudinal Andalusian Population Database (BDLPA): contact telephone numbers of the selected sample necessary to conduct the interviews and postal address to link the information with context environmental indicators.
• Survey (ESSOC): household data, sociodemographic and economic characteristics, habits and lifestyles, health, and emotional well-being.
• Population Health Database (BPS): clinical information related to chronic diseases, functional and cognitive assessment, health resources, population stratification, and drugs consumed</td><td>Linked and anonymized databases (BDLPA, ESSOC, and BPS)</td><td>Personal data (contact information, identifying data, and postal address)</td></tr>
<tr><td>Participants involved</td><td>•EASP
•IECA
•Sampling company (interviews)
•CSyF
•DGSPyOF
•DGAS, SAS (BPS)</td><td>•EASP
•IECA</td><td>•EASP
•IECA
•CSyF
•DGSPyOF 4 DGAS</td><td>•EASP
•UGR
•UdG</td><td>•EASP
•IECA</td></tr>
<tr><td>Technologies involved</td><td colspan="3">• The collected information is safeguarded at all times in servers of the EASP. The data recording and dumping process anonymously stores information, with the collected data being exclusively linked to a sample unit code.
• Access to the anonymized microdata is managed by EASP technicians through profiles boasting controlled permissions that allow for limiting, monitoring, and controlling access to the information.
• The data are sent through web platform “Consigna” of the Andalusian Regional Government in a compressed format requiring a password that is sent via a different channel than that used to notify their availability.</td><td>The data are sent through web platform “Consigna” of the Andalusian Regional Government in a compressed format requiring a password that is sent via a different channel than that used to notify their availability</td><td>Physical and logical deletion of all files at the operating system level</td></tr>
</table>

* BDLPA: Longitudinal Andalusian Population Database; BPS: Population Health Database; CSyF: Department of Health and Families of the Andalusian Regional Government; DGAS: General Directorate of Health Care and Health Outcomes; DGSPyOF: General Directorate of Public Health and Pharmaceutical Regulations (*Dirección General de Salud Pública y Ordenación Farmacéutica*); EASP: Andalusian School of Public Health; ESSOC: Health Care and Social Survey; IECA: Andalusian Institute of Statistics and Cartography; SAS: Andalusian Health Service; SES: Health Care Statistics Service (*Servicio de Estadísticas Sanitarias*); UdG: University of Girona; UGR: University of Granada.

Table S3. Analysis of the need and proportionality of the processing

Question	YES/NO	Rationale
**Legitimation**	Public interest or exercise of public powers	In addition to the consent obtained by telephone, the legal basis of this survey are the provisions of the following articles of the GDPR: 6.1.e) regarding the need to process data in order to accomplish a task carried out in the public interest or in the exercise of public powers vested in the data controller, and d), regarding the compulsory nature of such processing for the protection of the vital interests of the interested party or another natural person, as well as the specific applicable statistical regulations (Spanish Law 12/1989, of May 9th, on the Public Statistical Function; Spanish Law 4/2007, of April 4th, which amends Spanish Law 4/1989, of December 12th, on Statistics of the Autonomous Region of Andalusia, and approves the 2007–2010 Andalusian Statistics Plan, extended by virtue of Spanish Law 11/2010, of December 3rd)
**The intended purpose requires all the data to be collected for all persons concerned/interested parties (data minimization principle)**	YES	A random, representative sample has been obtained by means of an efficient sample design that minimizes the number of people to be interviewed
**The technologies used for the processing are suitable for the stated purpose with respect to the compliance with fundamental privacy principles**	YES	Only corporate and secure servers will be used, as well as secure and locally installed (not distributed) software
**The data are stored longer than necessary for data processing purposes (storage period limitation principle)**	NO	All data will be destroyed once the analyses have been completed
**The collected data will be used exclusively for the stated purpose and not for any other undeclared purpose incompatible with their legitimate use (purpose limitation principle)**	YES	No other analyses or statistical operations will be carried out beyond those described above

Table S4. Risk Management

Risk	Likelihood	Impact	Residual Risk	Measure*
**Error in the configuration of a system, application, workstation, printer, or network component**	Limited	Limited	Low	Equipment maintenance
	Limited	Limited	Low	Management of technical vulnerabilities
**Human errors in maintenance tasks**	Negligible	Significant	Low	Acceptable use of assets
	Negligible	Significant	Low	Equipment maintenance
	Negligible	Significant	Low	Management of changes to services provided by third parties
**Theft or loss of equipment, media, or devices containing personal data**	Limited	Maximum	Medium	Mobility device usage policy
	Limited	Maximum	Medium	Information security awareness, education, and training
	Limited	Maximum	Medium	Management of removable media
	Limited	Maximum	Medium	Management of registrations/de-registrations in the user registry
	Limited	Maximum	Medium	Restriction of access to information
	Limited	Maximum	Medium	Secure login procedures
	Limited	Maximum	Medium	Security incident responses
	Limited	Maximum	Medium	Evidence gathering
**Access to information, services, applications, or devices in an unauthorized way, by unauthorized persons, or breaking through barriers (hacking)**	Limited	Maximum	Medium	Access control policy
	Limited	Maximum	Medium	Management of registrations/de-registrations in the user registry
	Limited	Maximum	Medium	Notification of security events to the Competent Authority and interested parties
	Limited	Maximum	Medium	Evidence gathering
**Denial of service attack**	Negligible	Limited	Low	Network controls
	Negligible	Limited	Low	External firewalls, IDS/IPS
	Negligible	Limited	Low	Distributed Denial of Service (DDoS) Protection
**Existence of technical errors or failures leading to the unavailability of the information systems**	Limited	Limited	Low	Information security continuity planning
	Limited	Limited	Low	Implementation of the information security continuity
	Limited	Limited	Low	Notification of security events to the Competent Authority and interested parties
	Limited	Limited	Low	Evidence gathering
**Natural disasters affecting relevant infrastructures (floods, hurricanes, earthquakes, etc.)**	Negligible	Negligible	Low	Protection against external and environmental threats

* The entity responsible for these measures is the Andalusian School of Public Health (EASP). All these measures have been implemented, except for the "Information security awareness, education, and training", which is currently in the process of being implemented.
