# Supplementary material for: Impact of COVID-19 on the Health of the General and More Vulnerable Population and Its Determinants: Health Care and Social Survey–ESSOC, Study Protocol
Source: Int J Environ Res Public Health. 2021 Jul 31;18(15):8120. doi: 10.3390/ijerph18158120 (PMC8345631; doi:10.3390/ijerph18158120)
Supplement: Supplementary file 1 [file ijerph-18-08120-s001.zip › SupplementaryMaterial2_QuestionnairesSpanish.pdf]

Impact of COVID-19 on the health of the general and more vulnerable population and its determinants: Health care and social survey-ESSOC, study protocol

Cuestionarios de la Encuesta Sanitaria y Social (ESSOC)

Junta de Andalucía
Consejería de Salud y Familias
Escuela Andaluza de Salud Pública

Índice

1. Introducción 3
2. Cuestionarios de ESSOCgeneral 5
   Medición 1 - ESSOCgeneral. Cuestionario 5
   Medición 2 - ESSOCgeneral. Cuestionario muestra longitudinal 15
   Medición 2 - ESSOCgeneral. Cuestionario muestra nueva 29
   Medición 3 - ESSOCgeneral. Cuestionario muestra longitudinal 42
   Medición 3 - ESSOCgeneral. Cuestionario muestra nueva 54
   Medición 4 - ESSOCgeneral. Cuestionario muestra longitudinal 66
   Medición 4 - ESSOCgeneral. Cuestionario muestra nueva 79
   Medición 5 - ESSOCgeneral. Cuestionario muestra longitudinal 92
   Medición 5 - ESSOCgeneral. Cuestionario muestra nueva 105
4. Cuestionarios de ESSOCzonas 118
   Medición 1 - ESSOCzonas. Cuestionario 118
   Medición 2 - ESSOCzonas. Cuestionario muestra longitudinal 130
   Medición 2 - ESSOCzonas. Cuestionario muestra nueva 143
5. Cuestionarios de ESSOC+55 156
   Medición 1 – ESSOC+55. Cuestionario 156
   Medición 2 – ESSOC+55. Cuestionario muestra longitudinal 171
   Medición 2 – ESSOC+55. Cuestionario muestra nueva 171

1. Introducción

Los cuestionarios utilizados en la Encuesta Sanitaria y Social (ESSOC) son una de las fuentes de información del proyecto junto con, por ejemplo, la Base Poblacional de Salud (BPS) y la Base de Datos Longitudinal de Población de Andalucía (BDLPA).

La primera medición realizada de la ESSOC, se realizó con un cuestionario elaborado con el objetivo de describir la situación de la población andaluza de 16 y más años respecto de su estado de salud, determinantes sociales y condiciones de vida y convivencia durante el estado de alarma por la pandemia COVID-19 declarado por el Gobierno español el 14 de marzo de 2020 (Real Decreto 463/2020). Para su elaboración, se han utilizado preguntas ampliamente utilizadas en otras encuestas poblacionales de salud como la Encuesta Andaluza de Salud o la Encuesta Nacional de Salud de España, así como escalas ya validadas en otros estudios como la de bienestar emocional o de apoyo social.

En las siguientes mediciones de la ESSOC se utilizan dos tipos de cuestionarios, uno para la muestra longitudinal que ya ha contestado la encuesta en, al menos, la medición anterior, y otro para la muestra nueva que contesta la encuesta por primera vez. Cada cuestionario está organizado en bloques de información que en su mayoría son comunes a todas las mediciones (Tabla 1).

De una medición a la siguiente puede verse modificado el cuestionario debido a cambios menores como actualizaciones en las referencias temporales de algunas preguntas, o en ocasiones, cambios mayores que implican la eliminación de preguntas o la inclusión de preguntas o escalas compuestas.

Tabla 1. Bloques de información y variables introducidas en cada medición.

Área temática	1ª medición (M1)	2ª medición (M2)	3ª medición (M3)	4ª y 5ª mediciones (M4 y M5)
Características del hogar y la vivienda	Municipio, vivienda habitual, tipo vivienda, superficie, instalaciones, cambios en hogar, personas convivientes (<6/<16/>60), tipo hogar, equipamiento	Municipio, vivienda habitual, tipo vivienda, superficie, instalaciones, cambios en hogar[b], personas convivientes (<6/<16/>60), tipo hogar, equipamiento, habitaciones, personas con limitación y con cuidados	Municipio, vivienda habitual, tipo vivienda, superficie, cambios en hogar, personas convivientes (<6/<16/>60), tipo hogar, habitaciones, personas con limitación y con cuidados	Municipio, vivienda habitual, tipo vivienda, superficie, cambios en hogar, personas convivientes (<6/<16/>60), tipo hogar, habitaciones, personas con limitación y con cuidados
Uso del tiempo y convivencia	Tareas domésticas, cuidados, actividades diarias durante confinamiento (en el hogar y fuera), convivencia y relaciones, razones para optimismo			Tareas domésticas, cuidados, acompañamiento, actividades diarias[b] y dedicación
Salud y Bienestar Emocional	Diagnóstico COVID, gravedad, diagnóstico en entorno, autopercepción de salud general y mental (actual y último año), bienestar emocional[c], dificultad en confinamiento, molestias, enfermedad crónica, modificación de medicación	Diagnóstico COVID, gravedad, pruebas diagnósticas, diagnóstico en entorno, autopercepción de salud general y mental, bienestar emocional[bc], convivencia, dificultad en el confinamiento, felicidad, apoyo social y afectivo[c], molestias[b], enfermedades crónicas (padecer y limitación), medicamentos (uso y modificación de uso[b])	Diagnóstico COVID[b], gravedad, pruebas diagnósticas, diagnóstico en entorno, autopercepción de salud general y mental, bienestar emocional[bc], felicidad, apoyo social y afectivo[bc], molestias[b], enfermedades crónicas (padecer y limitación), medicamentos (uso y modificación de uso[b])	Diagnóstico COVID[b], gravedad, pruebas diagnósticas, diagnóstico en entorno, autopercepción de salud general y mental, bienestar emocional[bc], felicidad, apoyo social y afectivo[bc], molestias[b], enfermedades crónicas (padecer y limitación), medicamentos (uso y modificación de uso[b])
Hábitos y estilo de vida	Modificación de hábitos (ejercicio, tabaco, alcohol, dormir y alimentación)	Modificación del hábito[b] de: ejercicio, bebida, tabaco, dormir, alimentación; frecuencia de verduras y frutas al día, ejercicio, peso y altura[c], tabaco, alcohol, sueño, vacunación de la gripe	Modificación del hábito[b] de: ejercicio, bebida, tabaco, dormir, alimentación; frecuencia de verduras y frutas al día, ejercicio, peso y altura[c], tabaco, alcohol, sueño, vacunación de la gripe[b]	Modificación del hábito[b] de: ejercicio, bebida, tabaco, dormir, alimentación; frecuencia de verduras y frutas al día, ejercicio, peso y altura[c], tabaco, alcohol, sueño, vacunación de la gripe[b] y de covid-19
Situación económica y características sociodemográficas	Estudios, situación laboral, trabajo desde casa, tipo contrato, ocupación[c], convivencia en pareja, identificación de persona con más ingresos (estudios, situación laboral, tipo contrato, ocupación), dificultad de llegar fin de mes, retraso en pagos, ingresos, preocupaciones futuras, confianza en las instituciones públicas	Situación laboral, estudios, ocupación[c], desarrollo[b], capacidad laboral, persona con más ingresos (estudios, situación laboral, ocupación), dificultad de llegar fin de mes, retraso en pagos, cambio en la situación económica, estudios de padre y madre, preocupaciones futuras	Situación laboral, estudios, ocupación[c], desarrollo, capacidad laboral, persona con más ingresos (estudios, situación laboral, ocupación), dificultad de llegar fin de mes, retraso en pagos[b], cambio en la situación económica, estudios de padre y madre, preocupaciones futuras	Situación laboral, estudios, ocupación[c], desarrollo, capacidad laboral, persona con más ingresos (estudios, situación laboral, ocupación), dificultad de llegar fin de mes, retraso en pagos[b], cambio en la situación económica, estudios de padre y madre, preocupaciones futuras

[b] Variables que presentan modificaciones en su ámbito temporal en relación a la medición anterior
[c] Variables compuestas: bienestar emocional, apoyo social y afectivo, índice de masa corporal a partir de peso y altura, clase social a partir de ocupacion.

A continuación, se muestran los cuestionarios usados en todas las mediciones de ESSOCgeneral, ESSOCzonas y ESSOC+55.

2. Cuestionarios de ESSOCgeneral

Para la ESSOCgeneral se realizan 5 mediciones. En todas, excepto en la primera medición, se utiliza un cuestionario longitudinal y otro para la muestra nueva.

Medición 1 - ESSOCgeneral. Cuestionario

Este cuestionario estructura la información recogida en cuatro bloques de información:

- BLOQUE I. CARACTERÍSTICAS DEL HOGAR Y LA VIVIENDA
- BLOQUE II. USO DEL TIEMPO Y CONVIVENCIA
- BLOQUE III. SALUD Y BIENESTAR EMOCIONAL
- BLOQUE IV. EFECTOS ECONÓMICOS Y CARACTERÍSTICAS SOCIODEMOGRÁFICAS Y SOCIOECONÓMICAS

BLOQUE I. CARACTERÍSTICAS DEL HOGAR Y LA VIVIENDA

1. ¿Actualmente se encuentra en su vivienda habitual o reside en una vivienda distinta?
    1. Habitual
    2. Segunda residencia
    3. Otras:

POST: P1=1 ir a P2

   1. a ¿En qué municipio se encuentra actualmente:
   PRE: P1!=1
   Municipio: ____________

   1. b ¿Por qué motivos se encuentra en una vivienda distinta de la habitual? (Respuesta múltiple)
   PRE: P1!=1
   1. Las condiciones de esta vivienda son más confortables que las de su vivienda habitual
   2. Ha trasladado su residencia temporalmente por motivos laborales
   3. Ha trasladado su residencia para ayudar en el cuidado de familiares
   4. Otros motivos: ___

2. La vivienda en la que se encuentra actualmente es una:
    1. Vivienda unifamiliar independiente, adosada o pareada
    2. Piso o apartamento en un edificio con menos de 10 viviendas
    3. Piso o apartamento en un edificio con 10 o más viviendas
    4. Otro tipo de vivienda (Ej: Vivienda situada en un edificio destinado principalmente a otros fines: barraca, cabaña, chabola, etc.) Especifique.

3. ¿Cuál es la superficie útil de la vivienda en la que se encuentra actualmente?
    1. Menos de 46 m^2 útiles
    2. Entre 46 y 60
    3. Entre 61 y 75
    4. Entre 76 y 90
    5. Entre 91 y 120
    6. Más de 120
    98. No sabe
    99. No contesta

4. En la vivienda en la que está actualmente ¿disfruta de alguna de las siguientes instalaciones? (Respuesta múltiple). Nota: no se incluyen las instalaciones comunitarias.

1. Patio
2. Jardín
3. Azotea
4. Terraza
5. Balcón
6. Luz natural suficiente
7. Ventanas al exterior

5. A raíz del confinamiento ¿ha cambiado el grupo de personas con las que convive?

1. Sí
2. No

POST: P5=2 ir a P6

5a. A raíz del confinamiento ¿se han incorporado miembros nuevos en la vivienda en la que se encuentra actualmente?:
PRE: P5=1
1. -Personas de más de 60 años: nº I_I_I
2. -Personas de otros grupos de edad: nº I_I_I

5b. A raíz del confinamiento ¿se han ausentado miembros en la vivienda en la que se encuentra actualmente?:
PRE: P5=1
1. - Personas de más de 60 años: nº I_I_I
2. - Personas de otros grupos de edad: nº I_I_I

6. Para conocer las condiciones en las que está pasando este periodo de confinamiento me podría decir ¿qué número de personas residen actualmente incluyéndose usted?

Número de personas: |_|_|
6a. ¿De estas personas cuántas son mayores de 60 años?...... |_|_|
6b. ¿Y cuántas son menores de 16 años?... |_|_|
6c. ¿De las personas menores de 16, cuántas son menores de 6 años? I_I_I

7. A efectos de caracterizar su situación de convivencia durante el confinamiento, ¿me podría decir cuál de las siguientes alternativas lo refleja mejor?

1. Hogar unipersonal
2. Padre o madre solo/a que conviva con algún hijo/a
3. Pareja sin hijos que convivan en el hogar
4. Pareja con hijos/as que convivan en el hogar
5. Otro tipo de hogar

8. En relación con el equipamiento en su vivienda actual dispone de...(Respuesta múltiple)

1. Teléfono móvil
2. Ordenador/portátil
3. Tableta
4. Ninguna de las anteriores

9. ¿Dispone en su vivienda actual de acceso Internet/Wifi (a través de cualquier dispositivo fijo o Móvil, se incluye teléfono móvil)? Respuesta múltiple.

1. Sí, con datos de teléfono móvil
2. Sí, con Wifi
3. No

BLOQUE II. USO DEL TIEMPO Y CONVIVENCIA

Ahora vamos a hablar sobre cómo ha sido la situación de confinamiento para Ud.

10.Quienes de las siguientes personas ha realizado las siguientes acciones en su hogar durante el periodo de confinamiento.

1. Siempre yo
2. Habitualmente yo
3. Mi pareja y yo por igual
4. Habitualmente mi pareja
5. Siempre mi pareja
6. Madre de la persona entrevistada
7. Padre de la persona entrevistada
8. Persona remunerada
9. Otras personas. Especificar
10. No sabe/No procede

1. Las tareas domésticas (fregar, planchar, hacer las comidas…)
2. Las tareas de cuidado de hijos/as u otros menores del hogar
3. Tareas en adultos y menores con problemas de salud, movilidad, etc. que requieren algún tipo de dedicación o de cuidados especiales

11. ¿De las siguientes acciones que le voy a leer a continuación, dígame con qué frecuencia las ha realizado durante la última semana de confinamiento?

1. A diario
2. Varios días a la semana
3. Una vez a la semana
4. Menos de una vez a la semana
5. Nunca o casi nunca

1. Videollamadas y llamadas
2. Ver series, películas, televisión, radio, podcast.
3. Usar redes sociales
4. Actividades artísticas
5. Hacer ejercicio físico, actividades de relajación (yoga, mindfulness, etc)
6. Leer, estudiar...
7. Participar en actividades solidarias o de voluntariado
8. Otra actividad. Cuál: ____

12. ¿Podría decirme con qué frecuencia ha realizado durante la última semana de confinamiento las siguientes tareas fuera del hogar?

1. A diario
2. Varios días a la semana
3. Una vez a la semana
4. Menos de una vez a la semana
5. Nunca o casi nunca

-9. No Procede

1. Ir a trabajar
2. Coger el transporte público
3. Salir a comprar comida
4. Salir a por medicamentos
5. Sacar al perro
6. Salir para llevar comida o medicamentos a otros familiares, vecinos, etc.

7. Sacar la basura
8. Pasear y/o practicar deporte
9. Visita médica (propia o de acompañante)
10. Visitar y/o atender a familiares, vecinos, amigos…
11. Cuidado de campo, parcelas, animales…
12. Compras y servicios que no son comida: bancos, gasolinera, estanco, etc.
13. Otras tareas fuera del hogar: _______

13. Dígame ahora el grado de acuerdo con las siguientes frases que han expresado otras personas en referencia al periodo de confinamiento. Utilice una escala del 1 al 5, donde 1 es nada de acuerdo y 5 totalmente de acuerdo. (No Procede constará para todos los ítems)

1. Las relaciones con algunas personas con las que convivo han empeorado
2. He tenido ganas de hablar porque estaba angustiado/a, pero no lo he hecho
3. He sentido que estaba más unido/a con el resto de la sociedad
4. Me preocupa el nivel de estrés de mis hijos

14. Podría decirme qué aspectos le han permitido mantener el optimismo durante el periodo de confinamiento. (Máximo tres respuestas).

1. ___________
2. ___________
3. ___________

BLOQUE III. SALUD Y BIENESTAR EMOCIONAL

Ahora vamos a hablar de su estado de salud.

15. En primer lugar ¿tiene o ha tenido diagnosticado coronavirus?

1. Sí
2. No
3. Estoy a la espera del resultado
98. No sabe
99. No contesta

POST: P15=2 || P15=98 || P15=99 ir a P17

16. ¿Cómo de grave ha sido o es su proceso con la enfermedad?

PRE: P15=1 || P15=3

1. Muy leve
2. Leve
3. Grave
4. Muy grave

17. ¿Hay en su entorno alguien que tiene o ha tenido algún caso diagnosticado de infección por coronavirus? Respuesta múltiple

1. Persona/s de mi familia con la que resido en el hogar
2. Persona/s con la que resido en el hogar que no es familiar
3. Familiar/es que no conviven conmigo
4. Amigos, compañeros de trabajo/estudios, vecinos con lo que no resido en el hogar
5. Nadie de mi entorno

Hablemos a hora de forma general de cómo se encuentra…

18. En general diría que su salud es...

1. Excelente
2. Muy buena
3. Buena
4. Regular

5. Mala
6. No sabe/No está seguro
99. No contesta

19. Y en los 12 meses anteriores al coronavirus, diría que su estado era…
1. Excelente
2. Muy buena
3. Buena
4. Regular
5. Mala
6. No sabe/No está seguro
99. No contesta

20. En la actualidad, en general diría que su salud mental es…
1. Excelente
2. Muy buena
3. Buena
4. Regular
5. Mala
6. No sabe/No está seguro
99. No contesta

21. Y en los últimos 12 meses anteriores al coronavirus, diría que su salud mental era...
1. Excelente
2. Muy buena
3. Buena
4. Regular
5. Mala
6. No sabe/No está seguro
99. No contesta

22. A continuación, le voy a leer una lista de sentimientos o comportamientos que quizás haya tenido Vd. desde que empezó el confinamiento. Por favor, dígame con qué frecuencia, durante la última semana, se ha sentido...
1. En ningún momento o en casi ningún momento
2. En algún momento
3. Buena parte del tiempo
4. Todo o casi todo el tiempo
98. No sabe

1. Deprimido/a
2. Feliz
3. Solo/a
4. Ha tenido la sensación de disfrutar de la vida
5. Rebosante de energía
6. Tranquilo/a y relajado/a

23. Si toma también como referencia el periodo de confinamiento, dígame si está Ud. muy de acuerdo, de acuerdo, en desacuerdo o muy en desacuerdo con las siguientes frases.
1. Muy de acuerdo
2. De acuerdo
3. Ni de acuerdo ni en desacuerdo
4. En desacuerdo
5. Muy en desacuerdo

98. No sabe

1. He sido optimista respecto a mi futuro
2. Por lo general me he sentido bien conmigo mismo

24. En relación con antes del confinamiento, diría Vd. que…
1. Más/Mejor
2. Igual
3. Menos/Peor
-9. No procede

1. He realizado más, igual o menos ejercicio que antes del confinamiento
2. He consumido más, igual o menos bebidas alcohólicas que antes del confinamiento
3. He fumado más, igual, o menos que antes del confinamiento
4. He dormido mejor, igual o peor que antes del confinamiento
5. Me he alimentado mejor, igual o peor que antes del confinamiento

25. ¿Cómo le ha resultado hasta ahora la situación de confinamiento en el domicilio, muy fácil, bastante fácil, bastante difícil o muy difícil?
1. Muy fácil
2. Bastante fácil
3. Ni fácil ni difícil
4. Bastante difícil
5. Muy difícil

26. ¿Y si el confinamiento se prolongara dos semanas más?
1. Muy fácil
2. Bastante fácil
3. Ni fácil ni difícil
4. Bastante difícil
5. Muy difícil

27. Desde que empezó el confinamiento, ¿hasta qué punto ha notado Vd. alguna de las siguientes molestias? Utilice una escala del 1 al 5 en la que 1 es Nunca he sentido esa molestia y 5 Con mucha frecuencia.
1. Problemas de sueño
2. Problemas de estómago
3. Incremento de la tensión arterial
4. Mareos
5. Dolores de cabeza
6. Dolores musculares o de articulaciones
7. Sensación de opresión en el pecho
8. Otra molestia ¿Cuál? ________

28. ¿Padece Ud. alguna enfermedad crónica?
1. Si
2. No
POST: P28=2 ir a P30

29. ¿En qué medida se ha visto modificada la medicación que usted tomaba antes del confinamiento/coronavirus con respecto a ahora?
PRE: P28=1
1. He disminuido mi medicación
2. Sigo igual, no ha afectado en nada

3. He aumentado mi medicación (incluye si antes no consumía, pero ahora sí)
4. No consumo medicación

BLOQUE IV. EFECTOS ECONÓMICOS Y CARACTERÍSTICAS SOCIODEMOGRÁFICAS Y SOCIOECONÓMICAS.

En la última parte del cuestionario le vamos a hacer unas preguntas para poder clasificar estadísticamente sus respuestas. Las preguntas referidas al hogar se refieren a su hogar habitual no a la situación temporal durante el confinamiento.

30. ¿Cuáles son sus estudios terminados de más alto nivel?

Entrevistador: Anote el literal y señale la opción que corresponda. El informante debe especificar en su respuesta lo suficiente como para que se pueda codificar correctamente, por ejemplo: ha asistido menos de cinco años a la escuela, tres cursos completos de Ciencias Económicas y Empresariales, doctorado en Ciencias Sociales, etc.

Estudios terminados de más alto nivel: ________________________

1. Analfabetos/as y estudios primarios incompletos
2. Educación primaria
3. Primera etapa de la Educación Secundaria y similar
4. Segunda etapa de la Educación Secundaria y similar (Bachillerato y FP de Grado Medio)
5. Educación postsecundaria no superior
6. Formación Profesional de Grado Superior y títulos propios De universidades de duración igual o superior a 2 años
7. Diplomados/as universitarios/as, títulos propios universitarios de Experto o especialista y similares (Grados universitarios de 240 créditos ECTS, Bolonia)
8. Licenciados, másteres y especialidades en Ciencias de la Salud por el sistema de residencia y similares. Grados universitarios de más 240 créditos ECTS (Bolonia)
9. Título de Doctorado
10. No se puede codificar

31. ¿En qué situación de las siguientes se encuentra en la actualidad? (Si se encuentra en varias situaciones, indique solo la que considere principal).

1. Asalariado/a a tiempo completo
2. Asalariado/a a tiempo completo en ERTE
3. Asalariado/a a tiempo parcial
4. Asalariado/a a tiempo parcial por ERTE
5. Empresario/a con asalariados/as
6. Empresario/a sin asalariados/as o trabajador/a independiente o miembro de cooperativa
7. Trabajos familiares no remunerados (ayudas familiares)
8. Parado/a
9. Estudiante
10. Jubilado/a, retirado/a o jubilado/a anticipado/a
11. Incapacitado/a permanente
12.Realizando tareas de voluntariado social
13.Dedicado/a a las labores del hogar, al cuidado de niños u otras personas
14.Otra clase de inactividad económica
98.No sabe
98.No contesta

POST: P31=8 ir a P34

POST: P31=9 || P31=10 || P31=11 || P31=12 || P31=13 || P31=14 || P31=98 || P31=99 ir a P37

32. Respecto a la situación en la que desarrolla su trabajo ¿cómo ha sido durante el confinamiento?

PRE: P31=1 || P31=2 || P31=3 || P31=4 || P31=5 || P31=6 || P31=7

1. He trabajado desde casa

2. He trabajado fuera de casa
3. He trabajado desde casa y desde fuera de casa
4. He estado de permiso para ocuparme de mis hijos
5. He estado permiso para ocuparme de otros familiares
6. He estado de vacaciones anuales retribuidas
7. He estado de permiso no retribuido
8. Baja por enfermedad, accidente o situación de riesgo
9. Situación de ERTE o Cese de Actividad
99. NC

POST1: (P32=2 || P32=4 || P32=5 || P32=6 || P32=7 || P32=8) & (P31=1 || P31=2 || P31=3 || P31=4) ir a P33
POST2: (P32=2 || P32=4 || P32=5 || P32=6 || P32=7 || P32=8) & (P31=5 || P31=6 || P31=7) ir a P36

32.a.- ¿Trabajaba desde casa antes del confinamiento?
PRE: (P32=1 || P32=3) & (P31=1 || P31=2 || P31=3 || P31=4 || P31=5 || P31=6 || P31=7)
1. Sí
2. No

POST: (P31=5 || P31=6 || P31=7) ir a P36

33. ¿Qué tipo de contrato tiene en la actualidad?
PRE: P31=1 || P31=2 || P31=3 || P31=4
1. Contrato indefinido
2. Contrato temporal

POST: ir a P36

34. Me ha dicho que está Vd. en situación de desempleo. ¿En cuál de las siguientes situaciones se encuentra?
PRE: P31=8
1. Estoy desempleado por un ERTE debido al coronavirus
2. Estoy desempleado por un despido debido al coronavirus (sin ERTE)
3. He tenido que cerrar mi negocio
4. He tenido que cesar la actividad que me aportaba ingresos
5. Tenía opciones de comenzar en un trabajo pero se han postpuesto/cancelado debido al estado de alarma
6. Mi situación de desempleo es previa al estado de alarma y no se ha visto alterada
7. Otras situaciones: ________

35. En su último empleo, usted...
PRE: P31=8
1. Trabajaba por cuenta propia
2. Era asalariado con contrato indefinido
3. Era asalariado con contrato temporal o de duración determinada

36. En su empleo principal o en su último empleo ¿cuál es/era la ocupación, profesión u oficio que desempeña/ba?
PRE: P31=1 || P31=2 || P31=3 || P31=4 || P31=5 || P31=6 || P31=7 || P31=8
Precise lo más posible, por ejemplo: dependiente de comercio, mecánico de automóviles, tornero, peón de la construcción, operador de grúa, etc.). Entrevistador: Anote el literal y señale la opción que corresponda según la tabla de clasificación del manual. El informante debe especificar suficientemente para que se pueda codificar correctamente
Ocupación: ________________________

37. ¿Ha convivido durante el confinamiento con su pareja?
1. Sí

2. No. Tengo pareja, pero no he convivido con ella
3. No tengo pareja

Identificación de la persona de referencia del hogar (la que aporta más ingresos)

38. ¿En su hogar quién es la persona que aporta más ingresos?
1. Yo
2. Otra persona
3. Otra persona y yo a partes iguales

POST: P38=1 || P38=3 ir a P43

39. Y esa persona es…

PRE: P38=2
1. Su cónyuge/pareja
2. Su padre
3. Su madre
4. Otra persona
99. No Contesta

POST: P39=99 ir a P43

40. ¿Cuáles son los estudios terminados de más alto nivel de la persona que aporta más ingresos en el hogar?

PRE: P38=2 & P39!=99

Anote el literal y señale la opción que corresponda. El informante debe especificar en su respuesta lo suficiente como para que se pueda codificar correctamente, por ejemplo: ha asistido menos de cinco años a la escuela, tres cursos completos de Ciencias Económicas y Empresariales, doctorado en Ciencias Sociales, etc.

Estudios terminados de más alto nivel: ______________________

Agente CATI: no leer. Clasificar en función del literal contestado
1. Analfabetos/as y estudios primarios incompletos
2. Educación primaria
3. Primera etapa de la Educación Secundaria y similar
4. Segunda etapa de la Educación Secundaria y similar (Bachillerato y FP de Grado Medio
5. Educación postsecundaria no superior
6. Formación Profesional de Grado Superior y títulos propios De universidades de duración igual o superior a 2 años
7. Diplomados/as universitarios/as, títulos propios universitarios de Experto o especialista y similares (Grados universitarios de 240 créditos ECTS, Bolonia)
8. Licenciados, másteres y especialidades en Ciencias de la Salud por el sistema de residencia y similares. Grados universitarios de más 240 créditos ECTS (Bolonia)
9. Título de Doctorado
10. No se puede codificar

41. ¿En qué situación de las siguientes se encuentra en la actualidad la persona que aporta más ingresos al hogar? (Si se encuentra en varias situaciones, indique solo la que considere principal).

PRE: P38=2 & P39!=99
1. Asalariado/a a tiempo completo
2. Asalariado/a a tiempo completo en ERTE
3. Asalariado/a a tiempo parcial
4. Asalariado/a a tiempo parcial por ERTE
5. Empresario/a con asalariados/as
6. Empresario/a sin asalariados/as o trabajador/a independiente o miembro de cooperativa
7. Trabajos familiares no remunerados (ayudas familiares)
8. Parado/a

9. Estudiante
10. Jubilado/a, retirado/a o jubilado/a anticipado/a
11. Incapacitado/a permanente
12. Realizando tareas de voluntariado social
13. Dedicado/a a las labores del hogar, al cuidado de niños u otras personas
14. Otra clase de inactividad económica
98. No sabe
99. No contesta

POST: P41=9 || P41=10 || P41=11 || P41=12 || P41=13 || P41=14 || P41=98 || P41=99 ir a P43

42. En su empleo principal o en su último empleo ¿cuál es/era la ocupación, profesión u oficio que desempeña/ba la persona que aporta más ingresos al hogar?
PRE: P38=2 & P39!=99 & (P41=1 || P41=2 || P41=3 || P41=4 || P41=5 || P41=6 || P41=7 || P41=8)
Precise lo más posible, por ejemplo: dependiente de comercio, mecánico de automóviles, tornero, peón de la construcción, operador de grúa, etc.). Entrevistador: Anote el literal y señale la opción que corresponda según la tabla de clasificación del manual. El informante debe especificar suficientemente para que se pueda codificar correctamente
Ocupación: ______________________

Ingresos y situación económica del hogar

43. Un hogar puede tener diferentes fuentes de ingresos y más de un miembro del hogar puede contribuir con sus ingresos. En relación con el total de ingresos de su hogar, ¿cómo suelen llegar a fin de mes?
1. Con mucha facilidad
2. Con facilidad
3. Con cierta facilidad
4. Con cierta dificultad
5. Con dificultad
6. Con mucha dificultad
98. No sabe
99. No contesta

44. Desde que comenzó el confinamiento ¿ha tenido en su hogar algún retraso en el pago de recibos como la hipoteca, el alquiler, o suministros como la luz, el agua, impuestos, etc.? (Respuesta múltiple).
1. Hipoteca
2. Alquiler
3. Cuotas de compras aplazadas
4. Cuotas de seguros
5. Impuestos (IBI, los relacionados con vehículos, etc.)
6. Pagos de recibos de agua, gas, calefacción, electricidad, comunidad
7. Tasa de autónomo
8. Pago de colegios
9. Actividades extraescolares
10. Otros. Especificar. ____
11. Ninguna

45. Dígame en qué intervalo, de los que le leeré a continuación, se encuentran los ingresos mensuales netos del conjunto de miembros de su hogar EN EL MES DE FEBRERO.
1. Sin ingresos (no leer)
2. Menos de 450 euros

3. De 451 a 900 euros
4. De 901 a 1.600 euros
5. De 1.601 a 2.500 euros
6. De 2.501 a 3.000 euros
7. Más de 3.000 euros
98. No sabe
99. No contesta

Ya para terminar…
46. ¿Hasta qué punto cree que es probable que a usted o a su familia le afecte directamente en el futuro alguna de las siguientes situaciones que le voy a nombrar?

1. Muy probable
2. Bastante probable
3. Poco probable
4. Nada probable
5. Ya me ha ocurrido
98. No sabe
99. No contesta
-9. No procede

• Contraer la infección por coronavirus
• Crisis de abastecimiento (luz, agua, telefonía, alimentos, medicamentos)
• Pérdida de ingresos
• Desempleo
• Problemas con los estudios
• Revueltas, Inseguridad
• Saturación de los servicios sanitarios

47. En el caso de que se diera alguno de los problemas señalados en la pregunta anterior, ¿hasta qué punto cree Vd. que las instituciones públicas podrían resolver la situación?

1. Muy probable
2. Bastante probable
3. Poco probable
4. Nada probable
98. NS
99. NC

Medición 2 - ESSOCgeneral. Cuestionario muestra longitudinal

Este cuestionario se ha elaborado a partir de la 1ª medición de la ESSOC y de preguntas de la Encuesta Andaluza de Salud en su VI edición. En gris aparecen las preguntas que tienen un filtro asociado. La mayoría son preguntas ya recogidas en la 1ª de la ESOC. La estructura del cuestionario se mantiene en cuatro grandes bloques.

- BLOQUE I. CARACTERÍSTICAS DEL HOGAR Y LA VIVIENDA
- BLOQUE II. SALUD, BIENESTAR EMOCIONAL
- BLOQUE III: HÁBITOS Y ESTILOS DE VIDA
- BLOQUE IV. SITUACIÓN ECONÓMICA Y CARACTERÍSTICAS SOCIODEMOGRÁFICAS.

BLOQUE I. CARACTERÍSTICAS DEL HOGAR Y LA VIVIENDA

P.1. Desde la realización de la anterior encuesta, el dia <*fecha de la anterior entrevista*>, ¿Ha cambiado su residencia a una vivienda diferente?

1. Sí → pasar a P.2.
2. No → pasar a P.6.
3. No lo recuerdo/No estoy seguro/a → pasar a P.2.

P.2. ¿Actualmente se encuentra en su vivienda habitual o reside en una vivienda distinta?

1. Habitual → P.3.
2. Segunda residencia → P.2.a
3. Otras: → P.2.a

P.2.a. ¿En qué municipio se encuentra actualmente: Municipio: _________

P.2.b. ¿Por qué motivos se encuentra en una vivienda distinta de la habitual? (Respuesta múltiple)

1. Las condiciones de esta vivienda son más confortables que las de su vivienda habitual
2. Ha trasladado su residencia temporalmente por motivos laborales
3. Ha trasladado su residencia para ayudar en el cuidado de familiares
4. Otros motivos: ___
5. NS/NC

P.3. La vivienda en la que se encuentra actualmente es una:

1. Vivienda unifamiliar independiente, adosada o pareada
2. Piso o apartamento en un edificio con menos de 10 viviendas
3. Piso o apartamento en un edificio con 10 o más viviendas
4. Otro tipo de vivienda (Ej: Vivienda situada en un edificio destinado principalmente a otros fines barraca, cabaña, chabola, etc.) Especifique.
5. NS/NC

P.4. ¿Cuál es la superficie útil de la vivienda en la que se encuentra actualmente?

1. Menos de 46 m^2 útiles
2. Entre 46 y 60
3. Entre 61 y 75
4. Entre 76 y 90
5. Entre 91 y 120
6. Más de 120
7. No sabe
8. No contesta

P.5. En la vivienda en la que está actualmente ¿disfruta de alguna de las siguientes instalaciones? (Respuesta múltiple). Nota: no se incluyen las instalaciones comunitarias.

1. Patio
2. Jardín
3. Azotea
4. Terraza
5. Balcón

6. Luz natural suficiente
7. Ventanas al exterior

P.6. A raíz de la última entrevista ¿ha cambiado el grupo de personas con las que convive?

1. Sí → pasar a P.6.a.
2. No →pasar a P.11.
3. No lo recuerdo/No estoy seguro/a → pasar a P.6.a.

P.6.a. ¿Se han incorporado miembros nuevos en la vivienda en la que se encuentra actualmente?:

1.- Personas de más de 60 años: nº I_I_I
2.- Personas de 18-35 años: nº I_I_I
3.- Personas de otros grupos de edad: nº I_I_I
4.- No

P.6.b.¿Se han ausentado miembros en la vivienda en la que se encuentra actualmente?:

1.- Personas de más de 60 años: nº I_I_I
2.- Personas de 18-35 años: nº I_I_I
3.- Personas de otros grupos de edad: nº I_I_I
4. No

P.7. Para conocer las condiciones en las que está actualmente me podría decir ¿qué número de personas residen actualmente incluyéndose usted?
Número de personas: |_|_|

P.7.a. ¿De estas personas cuántas son mayores de 60 años?...... |_|_|
P.7.b. ¿Y cuántas son menores de 16 años?... |_|_|
P.7.c. ¿De las personas menores de 16, cuántas son menores de 6 años? I_I_I

P.8. A efectos de caracterizar su situación de convivencia, ¿me podría decir cuál de las siguientes alternativas lo refleja mejor?

1. Hogar unipersonal
2. Padre o madre solo/a que conviva con algún hijo/a
3. Pareja sin hijos que convivan en el hogar
4. Pareja con hijos/as que convivan en el hogar
5. Otro tipo de hogar
6. NC

P.9. En relación con el equipamiento en su vivienda actual dispone de... (Respuesta múltiple)

1. Teléfono móvil
2. Ordenador/portátil
3. Tableta
4. Ninguna de las anteriores

P.10. ¿Dispone en su vivienda actual de acceso Internet/Wifi (a través de cualquier dispositivo fijo o móvil, se incluye teléfono móvil)? Respuesta múltiple.

1. Sí, con datos de teléfono móvil
2. Sí, con Wifi
3. No

P.11. ¿Cuántas habitaciones tiene su vivienda? (no contar la cocina, los baños/lavabos o el comedor/sala de estar).

Número de habitaciones: |_|_|

P.12. Indique el número de personas (<16; 16-59; >=60) que presentan alguna de las condiciones que voy a leerle…

P.12.a. Tener alguna limitación, discapacidad de tipo físico, sensorial o psíquico o mental

1. SI, de que tramo de edad:
    - <16 |_|_|
    - 16-59 |_|_|
    - >=60 |_|_|
2. No

P.12.b. Requiere algún tipo de dedicación o de cuidados especiales

1. SI, de que tramo de edad:
    - <16 |_|_|
    - 16-59 |_|_|
    - >=60 |_|_|
2. No

BLOQUE II. SALUD, BIENESTAR EMOCIONAL

Identificación de la infección

P.13. En primer lugar (aunque ya nos lo indicó en la anterior entrevista, nos gustaría confirmar si…) ¿tiene o ha tenido diagnóstico de coronavirus?

1. Sí →pasar a P.13.a
2. No
3. Estoy a la espera del resultado
4. No sabe
5. No contesta

Filtro si 1 en P.13

P.13.a. ¿Cómo de grave ha sido o es su proceso con la enfermedad?

1. Muy leve
2. Leve
3. Grave
4. Muy grave

P.14. ¿Le han hecho alguna vez, alguna de las siguientes pruebas para ver si estaba infectado por el coronavirus? (MULTIRRESPUESTA)

1. Muestra de la garganta o la nariz Sí/No/NC
2. Muestra de sangre en el dedo Sí/No/NC
3. Muestra de sangre en vena Sí/No/NC

P.15. ¿Hay en su entorno alguien que tiene o ha tenido algún caso diagnosticado de infección por coronavirus? Respuesta múltiple

1. Persona/s de mi familia con la que resido en el hogar
2. Persona/s con la que resido en el hogar que no es familiar
3. Familiar/es que no conviven conmigo
4. Amigos, compañeros de trabajo/estudios, vecinos con lo que no resido en el hogar
5. Nadie de mi entorno
6. NC

Hablemos ahora de forma general de cómo se encuentra…

Autopercepción de salud

P.16. En la actualidad, en general diría que su salud es...

1. Excelente
2. Muy buena
3. Buena
4. Regular
5. Mala
6. No sabe/No está seguro.
7. No contesta.

P.17. En la actualidad, en general diría que su salud mental es...

1. Excelente
2. Muy buena
3. Buena
4. Regular
5. Mala
6. No sabe/No está seguro.
7. No contesta.

Bienestar emocional

P.18. A continuación, le voy a leer una lista de sentimientos o comportamientos que quizás haya tenido Vd. **durante la última semana**. Por favor, dígame con qué frecuencia, se ha sentido...

1. En ningún momento o en casi ningún momento
2. En algún momento,
3. Buena parte del tiempo,
4. Todo o casi todo el tiempo
5. No sabe

1. Deprimido/a
2. Feliz
3. Solo/a
4. Ha tenido la sensación de disfrutar de la vida
5. Rebosante de energía
6. Tranquilo/a y relajado/a

P.19. Si toma también como referencia **la última semana**, dígame Ud. su grado de acuerdo o desacuerdo con las siguientes frases.

1. Muy de acuerdo
2. De acuerdo
3. Ni de acuerdo ni en desacuerdo
4. En desacuerdo
5. Muy en desacuerdo
6. No sabe

1. Me he sido optimista respecto a mi futuro
2. Por lo general me he sentido bien conmigo mismo

Convivencia y relaciones

P.20. Dígame ahora el grado de acuerdo con las siguientes frases **durante el último mes.** Utilice una escala del 1 al 5, donde 1 es nada de acuerdo y 5 totalmente de acuerdo. (No Procede constará para los ítems uno y cinco)

1. Las relaciones con algunas personas con las que convivo han empeorado
2. Las relaciones con algunas personas con las que NO convivo han empeorado
3. He tenido ganas de hablar porque estaba angustiado/a, pero no lo he hecho
4. He sentido que estaba más unido/a con el resto de la sociedad.
5. Me preocupa el nivel de estrés de mis hijos.

P.21. ¿Cómo le ha resultado hasta ahora la situación de confinamiento durante el **último mes**, muy fácil, bastante fácil, bastante difícil o muy difícil?

1. Muy fácil
2. Bastante fácil
3. Ni fácil ni difícil (No leer)
4. Bastante difícil
5. Muy difícil

<u>Felicidad y apoyo social y afectivo</u>

P.22. En términos generales, ¿en qué medida se considera Ud. una persona feliz o infeliz? Por favor, use una escala de 0 a 10, en la que 0 significa que se considera "completamente infeliz" y 10 que se considera "completamente feliz".

0 1 2 3 4 5 6 7 8 9 10 Ns/Nc 99

P.23. En la siguiente lista se muestran algunas cosas que otras personas hacen por nosotros o nos proporcionan. **Desde el principio del confinamiento**:

1. Tanto como deseo
2. Casi como deseo
3. Ni mucho ni poco
4. Menos de lo que deseo
5. Mucho menos de lo que deseo

P.23.1. Recibo visitas/llamadas de mis amigos y familiares
P.23.2. Recibo ayuda en asuntos relacionados con mi casa
P.23.3. Recibo elogios y reconocimientos cuando hago bien mi trabajo
P.23.4. Cuento con personas que se preocupan de lo que me sucede
P.23.5. Recibo amor y afecto
P.23.6. Tengo la posibilidad de hablar con alguien de mis problemas en el trabajo y/o en la casa
P.23.7. Tengo la posibilidad de hablar con alguien de mis problemas personales y familiares
P.23.8. Tengo posibilidad de hablar con alguien de mis problemas económicos
P.23.9. Recibo invitaciones para distraerme y salir con otras personas
P.23.10. Recibo consejos útiles cuando me ocurre algún acontecimiento importante en mi vida
P.23.11. Recibo ayuda cuando estoy enfermo/a en la cama

<u>Molestias y enfermedades crónicas</u>

P.24. Durante el **último mes**, ¿hasta qué punto ha notado Vd. alguna de las siguientes molestias? Utilice una escala del 1 al 5 en la que 1 es Nunca he sentido esa molestia y 5 Con mucha frecuencia.

1. Problemas de sueño
2. Problemas de estómago
3. Incremento de la tensión arterial
4. Mareos
5. Dolores de cabeza
6. Dolores musculares o de articulaciones
6a. Otros dolores
7. Sensación de opresión en el pecho
8. Otra molestia

Filtro si 5, 6 o 6a en P.24.
P.24.a. Alguno de estos dolores, ¿ha aparecido por primera vez durante el confinamiento?
1- Sí
2- No
9- NS/NC

P.25. ¿Le ha dicho un/a profesional de la salud (médico/a, enfermero/a) que usted padece actualmente alguna de las siguientes enfermedades crónicas? Si responde "SI" hacer P.25.a. ¿Esa condición le limita alguna de sus actividades?

ENFERMEDAD CRÓNICA	P.25. ¿Le ha dicho un/a profesional de la salud si tiene esta condición? (si es 'No', saltar a la siguiente)			P.25.a ¿Esa condición le limita alguna de sus actividades?	
	Sí	No	No lo sabe	Sí	No
1. Diabetes/azúcar alta en sangre/azúcar en orina	☐	☐	☐	☐	☐
2. Hipertensión/presión arterial alta	☐	☐	☐	☐	☐
3. Colesterol alto	☐	☐	☐	☐	☐
4. Enfermedad crónica del pulmón / enfisema / bronquitis crónica	☐	☐	☐	☐	☐
5. Asma	☐	☐	☐	☐	☐
6. Enfermedades del corazón (Trastornos cardíacos/insufiencia cardíaca /insuficiencia cardíaca congestiva; angina de pecho; Infarto de miocardio/ataque al corazón)	☐	☐	☐	☐	☐
7. Dolor crónico (pecho; espalda, cuello, hombro, cintura (cervical/lumbar; Migrañas/Jaquecas/ Cefaleas crónicas / dolor de cabeza frecuente)	☐	☐	☐	☐	☐
8. Alergias crónicas (como rinitis, inflamación de ojos, dermatitis, alergia a los alimentos, etc), asma alérgica excluida	☐	☐	☐	☐	☐
9. Mala circulación	☐	☐	☐	☐	☐
10. Varices en las piernas	☐	☐	☐	☐	☐
11. Trastornos depresivos	☐	☐	☐	☐	☐
12. Trastornos de ansiedad	☐	☐	☐	☐	☐

13. Otra enfermedad crónica	☐	☐	☐	☐	☐

Medicamentos

P.26. ¿En qué medida se ha visto modificada la medicación que usted tomaba antes del último mes con respecto a ahora?

1. He disminuido mi medicación
2. Sigo igual, no ha afectado en nada
3. He aumentado mi medicación (incluye si antes no consumía, pero ahora sí)
4. No consumo medicación.

Filtro si 1,2 o 3 en la P.26.

P.26.a. Y En general, ha cambiado la manera en que tomo mis medicamentos

1. Sí, ahora estoy cumpliendo mejor con la medicación
2. Sí, ahora estoy cumpliendo peor con la medicación
3. Sigo tomando los mismos medicamentos de la misma manera

P.27. (PARA TODOS). Y en las **últimas 2 semanas**, ¿ha utilizado algún tipo de medicamento (gotas, pastillas, inyecciones, supositorios, etc.)?

1. Sí
2. No
9. NC

Filtro Si 1 en P.27.

P.27.a. Si ha tomado medicamentos, ¿Qué tipo de medicamento?

1. Medicinas para el catarro, gripe, garganta, bronquios
2. Medicinas para el dolor
3. Medicinas para para bajar la fiebre
4. Antibióticos
5. Tranquilizantes, sedantes
6. Pastillas para dormir
7. Medicamentos antidepresivos
8. Medicamentos para la alergia
9. Medicinas para el corazón
10. Medicinas para la tensión arterial
11. Medicamentos para la diabetes
12. Medicamentos para bajar el colesterol
13. Otros medicamentos

BLOQUE III: HÁBITOS Y ESTILOS DE VIDA

P.28. En relación al **último mes**, diría Vd. que…

1. He realizado más, igual o menos ejercicio
2. He consumido más, igual o menos bebidas alcohólicas
3. He fumado más, igual, o menos
4. He dormido mejor, igual o peor
5. Me he alimentado mejor, igual o peor que antes
6. Mi peso ha aumentado, se ha mantenido o ha disminuido

Alimentación

P.29. ¿Cuántas raciones de verdura u hortalizas consume al día (las guarniciones o acompañamientos contabilizan como ½ ración)?

1. Dos o más al día (al menos una de ellas en ensalada o crudas)
2. Dos o más al día (aunque ninguna de ellas en ensalada o crudas)
3. Diariamente, aunque menos de dos al día
4. No diariamente, pero tres o más por semana
5. Menos de tres veces por semana
6. No consume
9. NS/NC

P.30. ¿Cuántas piezas de fruta (incluyendo zumo natural) consume al día?

1. Tres o más al día
2. Diariamente, aunque menos de tres veces al día
3. No diariamente, pero tres o más veces por semana
4. Menos de tres veces por semana
5. No consume
9. NS/NC

Actividad física

P.31. Durante las **últimas dos semanas**, ¿Cuántos días a la semana practica normalmente durante más de 30 minutos …? *(incluyendo desplazamientos al trabajo o actividades similares)*

Nº de días/sem

1. Caminar …………………………………………………………………………………………………… …. |_|_|
2. Una actividad física ligera como gimnasia suave, juegos que requieren poco esfuerzo y similares ……. …………………………………………………. ………………………………………………………… ……. |_|_|
3. Una actividad física moderada como montar en bicicleta, gimnasia, aeróbic, correr, natación ………………………………………………………………………………………………………..….… |_|_|
4. Entrenamiento físico deportivo como fútbol, baloncesto, ciclismo, natación de competición, judo, kárate o similares ……………………………………………………… |_|_|

P.32. ¿Podría decirme cuánto pesa, aproximadamente, sin zapatos ni ropa? (si está embarazada en la actualidad, debe indicar cuánto pesaba antes del inicio del embarazo)

Kilos: |_|_|_|
No sabe 998
No contesta 999

P.33.¿Y cuánto mide, aproximadamente, sin zapatos?

Cms.: |_|_|_|
No sabe 998
No contesta 999

Tabaco

P.34. Cambiando de tema, ¿podría usted decirme si fuma?

1. Sí, fuma diariamente

2. Sí fuma, pero no diariamente
3. No fuma, pero ha fumado
4. No fuma ni ha fumado nunca de manera habitual
9. No contesta

Alcohol

P.35. ¿Ha bebido usted en estas dos últimas semanas alguna bebida alcohólica?
1. Sí, diariamente → pasar a P.35.a
2. Sí, pero no diariamente. → pasar a P.35.a
3. No
9. NS/NC

P.35.a. Durante las **últimas 2 semanas** ¿alguna vez ha tomado 6/5 (hombre/mujer, respectivamente) (5/4 PARA MAYORES DE 65 AÑOS) o más bebidas estándar en una misma ocasión? (Por "ocasión" entendemos tomar las bebidas en un intervalo aproximado de cuatro a seis horas)."
1. Sí, alguna vez
2. Sí, varias veces
3. No
8. No sabe
9. No contesta

P.36. Durante las últimas 2 semanas, ¿ha utilizado la bebida para calmar su ansiedad?

1. Totalmente cierto
2. Bastante cierto
3. Bastanto falso
4. Totalmente falso
5. NS/NC

Sueño

P.37. ¿Podría indicarme, aproximadamente, cuántas horas al día duerme habitualmente?

Horas:	|_|_|
NS/NC	99

P.37.a. Las horas que duerme, ¿le permiten descansar lo suficiente?
1. Sí
2. No
8. NS
9. NC

Vacunas

P.38. Pasando a otro tema, querríamos saber si se ha vacunado de la gripe en la última campaña
1. Sí
2. No
3. NS/NC

BLOQUE IV. EFECTOS ECONÓMICOS Y CARACTERÍSTICAS SOCIODEMOGRÁFICAS Y SOCIOECONÓMICAS.

Situación laboral

P.39. ¿Ha cambiado su situación laboral desde que realizó la primera encuesta?

1. Sí → pasar a P.40.
2. No → pasar a P.42.
3. No lo recuerdo/No estoy seguro/a → pasar a P.40.

P.40. ¿En qué situación de las siguientes se encuentra en la actualidad? (Si se encuentra en varias situaciones, indique solo la que considere principal).

1.Asalariado/a a tiempo completo.
2.Asalariado/a a tiempo completo en ERTE
3.Asalariado/a a tiempo parcial
4.Asalariado/a a tiempo parcial por ERTE
5.Empresario/a con asalariados/as
6.Empresario/a sin asalariados/as o trabajador/a independiente o miembro de cooperativa
7.Trabajos familiares no remunerados (ayudas familiares).
8.Parado/a
9.Estudiante
10.Jubilado/a, retirado/a o jubilado/a anticipado/a
11.Incapacitado/a permanente
12.Realizando tareas de voluntariado social
13.Dedicado/a a las labores del hogar, al cuidado de niños u otras personas. 10
14.Otra clase de inactividad económica
98.No sabe
98.No contesta

Filtro: P40=1,2,3,4
P.41. ¿Qué tipo de contrato tiene en la actualidad?

1.Contrato indefinido
2.Contrato temporal.

Para trabajadores: Filtro: P40=1,2,3,4,5,6,7 o P31= 1,2,3,4,5,6,7 de la primera oleada
P.42. Respecto a la situación en la que desarrolla su trabajo ¿cómo es **actualmente**?

1. Trabajo desde casa
2. Trabajo fuera de casa
3. Trabajo desde casa y desde fuera de casa
4. Estoy de permiso para ocuparme de mis hijos
5. Estoy permiso para ocuparme de otros familiares
6. Estoy de vacaciones anuales retribuidas
7. Estoy de permiso no retribuido
8. Otra situación

Para trabajadores Filtro: P40=1,2,3,4,5,6,7 o P31= 1,2,3,4,5,6,7 de la primera oleada
P.43. Asumiendo que su capacidad laboral en su mejor estado de salud tuviera un valor de 10 puntos, ¿cuántos puntos daría a su capacidad laboral actual? 0 (totalmente incapacitado) …10 (muy buena capacidad laboral)

0 1 2 3 4 5 6 7 8 9 10 Ns/Nc 99

Filtro: P40=8 o P31=8 de la primera oleada
P.44. Me ha dicho que está Vd. en situación de desempleo. ¿En cuál de las siguientes situaciones se encuentra actualmente?

1. Estoy desempleado por un ERTE debido al coronavirus.

2. Estoy desempleado por un despido debido al coronavirus (sin ERTE).
3. He tenido que cerrar mi negocio.
4. He tenido que cesar la actividad que me aportaba ingresos
5. Tenía opciones de comenzar en un trabajo, pero se han postpuesto/cancelado debido al estado de alarma
6. Mi situación de desempleo es previa al estado de alarma y no se ha visto alterada
7. Otras situaciones:

Identificación de la persona de referencia del hogar (la que aporta más ingresos)

P.45. Actualmente, la persona que aporta más ingresos al hogar, ¿es la misma que cuando le hicieron la encuesta anterior?

1. SÍ → pasar a P.50.
2. NO → pasar a P.46.
3. No lo recuerdo/No estoy seguro/a → pasar a P.46.

P.46. ¿En su hogar quién es la persona que aporta más ingresos?

1. Yo (ir a P.50.)
2. Otra persona (ir a P47)
3. Otra persona y yo a partes iguales (ir a P.50.)

P.47. Y esa persona es...

1. Su cónyuge/pareja
2. Su padre
3. Su madre
4. Otra persona.
5. No Contesta

P.48. ¿Cuáles son los estudios terminados de más alto nivel de la persona que aporta más ingresos en el hogar?

Anote el literal y señale la opción que corresponda. El informante debe especificar en su respuesta lo suficiente como para que se pueda codificar correctamente, por ejemplo: ha asistido menos de cinco años a la escuela, tres cursos completos de Ciencias Económicas y Empresariales, doctorado en Ciencias Sociales, etc.
Estudios terminados de más alto nivel: ________________________
Agente CATI: no leer. Clasificar en función del literal contestado

1. Analfabetos/as y estudios primarios incompletos.
2. Educación primaria.
3. Primera etapa de la Educación Secundaria y similar.
4. Segunda etapa de la Educación Secundaria y similar
5. Bachillerato y FP de Grado Medio.
6. Educación postsecundaria no superior.
7. Formación Profesional de Grado Superior y títulos propios De universidades de duración igual o superior a 2 años.
8. Diplomados/as universitarios/as, títulos propios universitarios de Experto o especialista y similares (Grados universitarios de 240 créditos ECTS, Bolonia).
9. Licenciados, másteres y especialidades en Ciencias de la Salud por el sistema de residencia y similares. Grados universitarios de más 240 créditos ECTS (Bolonia).
10. Título de Doctorado.
11. No se puede codificar.

P.49. ¿En qué situación de las siguientes se encuentra en la actualidad la persona que aporta más ingresos al hogar? (Si se encuentra en varias situaciones, indique solo la que considere principal).

1. Asalariado/a a tiempo completo
2. Asalariado/a a tiempo completo en ERTE
3. Asalariado/a a tiempo parcial
4. Asalariado/a a tiempo parcial por ERTE
5. Empresario/a con asalariados/as
6. Empresario/a sin asalariados/as o trabajador/a independiente o miembro de cooperativa
7. Trabajos familiares no remunerados (ayudas familiares).
8. Parado/a
9. Estudiante
10. Jubilado/a, retirado/a o jubilado/a anticipado/a
11. Incapacitado/a permanente
12. Realizando tareas de voluntariado social
13. Dedicado/a a las labores del hogar, al cuidado de niños u otras personas.
14. Otra clase de inactividad económica
98. No sabe
99. No contesta

Filtro: P49=1 a 8
P.49.a. En su empleo principal o en su último empleo ¿cuál es/era la ocupación, profesión u oficio que desempeña/ba la persona que aporta más ingresos al hogar?

Precise lo más posible, por ejemplo: dependiente de comercio, mecánico de automóviles, tornero, peón de la construcción, operador de grúa, etc.). Entrevistador: Anote el literal y señale la opción que corresponda según la tabla de clasificación del manual. El informante debe especificar suficientemente para que se pueda codificar correctamente
Ocupación: ___________________

Ingresos y situación económica del hogar

P.50. Un hogar puede tener diferentes fuentes de ingresos y más de un miembro del hogar puede contribuir con sus ingresos. En relación con el total de ingresos de su hogar, ¿cómo suelen llegar a fin de mes?

1. Con mucha facilidad.
2. Con facilidad.
3. Con cierta facilidad.
4. Con cierta dificultad.
5. Con dificultad
6. Con mucha dificultad
7. No sabe.
8. No contesta.

P.51. Desde que comenzó el confinamiento ¿ha tenido en su hogar algún retraso en el pago de recibos como la hipoteca, el alquiler, o suministros como la luz, el agua, impuestos, etc.? (Respuesta múltiple). SÍ/NO Y EN CASO AFIRMATIVO SI SE HA RESUELTO

1. Hipoteca
2. Alquiler
3. Cuotas de compras aplazadas
4. Cuotas de seguros
5. Impuestos (IBI, los relacionados con vehículos, etc.)

6. Pagos de recibos de agua, gas, calefacción, electricidad, comunidad
7. Tasa de autónomo
8. Pago de colegios
9. Actividades extraescolares
10. Otros
11. Ninguna

P.52. ¿Ha cambiado su situación económica desde que realizó la primera encuesta?

1. Sí, ha mejorado → pasar a P.53.
2. Sí, ha empeorado→ pasar a P.53.
3. No → pasar a P.54.
4. No lo recuerdo/Ne estoy seguro/a → pasar a P.53.

P.53. Dígame en qué intervalo, de los que le leeré a continuación, se encuentran los ingresos mensuales netos del conjunto de miembros de su hogar EN EL MES ANTERIOR

1. Sin ingresos (no leer)
2. Menos de 450 euros
3. De 451 a 900 euros
4. De 901 a 1.600 euros
5. De 1.601 a 2.500 euros
6. De 2.501 a 3.000 euros
7. Más de 3.000 euros

98. NS
98. NC

P52a. ¿Cuáles son los estudios terminados de más alto nivel de su padre?
P52b. ¿Y de su madre?

- Primarios o menos
- Secundarios
- Universitarios
- No tiene padre
- No contesta

Ya para terminar… sobre la preocupación en el futuro

P.54. ¿Hasta qué punto le preocupa que a usted o a su familia le afecte directamente en el futuro alguna de las siguientes situaciones que le voy a nombrar?

1. Mucho
2. Bastante
3. Algo
4. Poco
5. Nada
6. Ya me OCURRE / ha ocurrido.
7. No sabe.
8. No contesta
9. No procede

1. Contagiarse por coronavirus (IESA)
2. Crisis de abastecimiento (luz, agua, telefonía, alimentos, medicamentos)
3. Pérdida de ingresos
4. Desempleo
5. Problemas con los estudios
6. Revueltas, Inseguridad
7. No recibir una la atención sanitaria que necesite

Esto es todo, muchas gracias por su colaboración y por el tiempo que nos ha dedicado respondiendo esta encuesta. Le recuerdo que tiene a su disposición el número de teléfono **900 10 17 49** por si necesita resolver cualquier duda o información.

Medición 2 - ESSOCgeneral. Cuestionario muestra nueva

Este cuestionario se ha elaborado fundamentalmente a partir del cuestionario de la muestra longitudinal de la 2ª medición de la ESSOC modificando aquellas preguntas que hacían referencia a la 1ª medición realizada por el IECA. La estructura del cuestionario se mantiene en cuatro grandes bloques.

- BLOQUE I. CARACTERÍSTICAS DEL HOGAR Y LA VIVIENDA
- BLOQUE II. SALUD, BIENESTAR EMOCIONAL
- BLOQUE III: HÁBITOS Y ESTILOS DE VIDA
- BLOQUE IV. SITUACIÓN ECONÓMICA Y CARACTERÍSTICAS SOCIODEMOGRÁFICAS.

BLOQUE I. CARACTERÍSTICAS DEL HOGAR Y LA VIVIENDA

P.2. ¿Actualmente se encuentra en su vivienda habitual o reside en una vivienda distinta?

1. Habitual → P.3.
2. Segunda residencia → P.2.a
3. Otras: → P.2.a

P.2.a. ¿En qué municipio se encuentra actualmente: Municipio: _________

P.2.b. ¿Por qué motivos se encuentra en una vivienda distinta de la habitual? (Respuesta múltiple)

1. Las condiciones de esta vivienda son más confortables que las de su vivienda habitual
2. Ha trasladado su residencia temporalmente por motivos laborales
3. Ha trasladado su residencia para ayudar en el cuidado de familiares
4. Otros motivos: ___
5. NS/NC

P.3. La vivienda en la que se encuentra actualmente es una:

1. Vivienda unifamiliar independiente, adosada o pareada
2. Piso o apartamento en un edificio con menos de 10 viviendas
3. Piso o apartamento en un edificio con 10 o más viviendas
4. Otro tipo de vivienda (Ej: Vivienda situada en un edificio destinado principalmente a otros fines barraca, cabaña, chabola, etc.) Especifique.
5. NS/NC

P.4. ¿Cuál es la superficie útil de la vivienda en la que se encuentra actualmente?

1. Menos de 46 m^2 útiles
2. Entre 46 y 60
3. Entre 61 y 75
4. Entre 76 y 90
5. Entre 91 y 120
6. Más de 120
7. No sabe
8. No contesta

P.5. En la vivienda en la que está actualmente ¿disfruta de alguna de las siguientes instalaciones? (Respuesta múltiple). Nota: no se incluyen las instalaciones comunitarias.

1. Patio
2. Jardín
3. Azotea
4. Terraza
5. Balcón
6. Luz natural suficiente
7. Ventanas al exterior

P.6.a. ¿Se han incorporado miembros nuevos en la vivienda en la que se encuentra actualmente, desde el inicio del estado de alarma?:

1.- Personas de más de 60 años: nº I_I_I
2.- Personas de 18-35 años: nº I_I_I
3.- Personas de otros grupos de edad: nº I_I_I
4.- No

P.6.b.¿Se han ausentado miembros en la vivienda en la que se encuentra actualmente, desde el inicio del estado de alarma?:

1.- Personas de más de 60 años: nº I_I_I
2.- Personas de 18-35 años: nº I_I_I
3.- Personas de otros grupos de edad: nº I_I_I
4. No

P.7. Para conocer las condiciones en las que está actualmente me podría decir ¿qué número de personas residen actualmente incluyéndose usted?

Número de personas: |_|_|
P.7.a. ¿De estas personas cuántas son mayores de 60 años?...... |_|_|
P.7.b. ¿Y cuántas son menores de 16 años?... |_|_|
P.7.c. ¿De las personas menores de 16, cuántas son menores de 6 años? I_I_I

P.8. A efectos de caracterizar su situación de convivencia, ¿me podría decir cuál de las siguientes alternativas lo refleja mejor?

1. Hogar unipersonal
2. Padre o madre solo/a que conviva con algún hijo/a
3. Pareja sin hijos que convivan en el hogar
4. Pareja con hijos/as que convivan en el hogar
5. Otro tipo de hogar
6. NC

P.9. En relación con el equipamiento en su vivienda actual dispone de... (Respuesta múltiple)

1. Teléfono móvil
2. Ordenador/portátil
3. Tableta
4. Ninguna de las anteriores

P.10. ¿Dispone en su vivienda actual de acceso Internet/Wifi (a través de cualquier dispositivo fijo o móvil, se incluye teléfono móvil)? Respuesta múltiple.

1. Sí, con datos de teléfono móvil
2. Sí, con Wifi
3. No

P.11. ¿Cuántas habitaciones tiene su vivienda? (no contar la cocina, los baños/lavabos o el comedor/sala de estar).
Número de habitaciones: |_|_|

P.12. Indique el número de personas (<16; 16-59; >=60) que presentan alguna de las condiciones que voy a leerle…

P.12.a. Tener alguna limitación, discapacidad de tipo físico, sensorial o psíquico o mental

1. SI, de que tramo de edad:
   - <16 |_|_|
   - 16-59 |_|_|
   - >=60 |_|_|
2. No

P.12.b. Requiere algún tipo de dedicación o de cuidados especiales

1. SI, de que tramo de edad:
   - <16 |_|_|
   - 16-59 |_|_|
   - >=60 |_|_|
2. No

BLOQUE II. SALUD, BIENESTAR EMOCIONAL

<u>Identificación de la infección</u>

P.13. En primer lugar ¿tiene o ha tenido diagnóstico de coronavirus?

1. Sí →pasar a P.13.a
2. No
3. Estoy a la espera del resultado
4. No sabe
5. No contesta

Filtro si 1 en P.13
P.13.a. ¿Cómo de grave ha sido o es su proceso con la enfermedad?

1. Muy leve
2. Leve
3. Grave
4. Muy grave

P.14. ¿Le han hecho alguna vez, alguna de las siguientes pruebas para ver si estaba infectado por el coronavirus? (MULTIRRESPUESTA)

1. Muestra de la garganta o la nariz Sí/No/NC
2. Muestra de sangre en el dedo Sí/No/NC
3. Muestra de sangre en vena Sí/No/NC

P.15. ¿Hay en su entorno alguien que tiene o ha tenido algún caso diagnosticado de infección por coronavirus? Respuesta múltiple

1. Persona/s de mi familia con la que resido en el hogar
2. Persona/s con la que resido en el hogar que no es familiar
3. Familiar/es que no conviven conmigo
4. Amigos, compañeros de trabajo/estudios, vecinos con lo que no resido en el hogar
5. Nadie de mi entorno
6. NC

Hablemos ahora de forma general de cómo se encuentra…

Autopercepción de salud

P.16. En la actualidad, en general diría que su salud es…

1. Excelente
2. Muy buena
3. Buena
4. Regular
5. Mala
6. No sabe/No está seguro.
7. No contesta.

P16a. Y en los 12 meses anteriores al coronavirus, diría que su estado era…

1. Excelente
2. Muy bueno
3. Bueno
4. Regular
5. Malo
6. No sabe/No está seguro
7. No contesta

P.17. En la actualidad, en general diría que su salud mental es…

1. Excelente
2. Muy buena
3. Buena
4. Regular
5. Mala
6. No sabe/No está seguro.
7. No contesta.

P17a. Y en los últimos 12 meses anteriores al coronavirus, diría que su salud mental era…

1. Excelente
2. Muy buena
3. Buena
4. Regular
5. Mala
6. No sabe/No está seguro
7. No contesta

Bienestar emocional

P.18. A continuación, le voy a leer una lista de sentimientos o comportamientos que quizás haya tenido Vd. **durante la última semana**. Por favor, dígame con qué frecuencia, se ha sentido…

1. En ningún momento o en casi ningún momento
2. En algún momento,
3. Buena parte del tiempo,
4. Todo o casi todo el tiempo
5. No sabe

1. Deprimido/a
2. Feliz
3. Solo/a
4. Ha tenido la sensación de disfrutar de la vida

5. Rebosante de energía
6. Tranquilo/a y relajado/a

P.19. Si toma también como referencia **la última semana**, dígame Ud. su grado de acuerdo o desacuerdo con las siguientes frases.

1. Muy de acuerdo
2. De acuerdo
3. Ni de acuerdo ni en desacuerdo
4. En desacuerdo
5. Muy en desacuerdo
6. No sabe

1. Me he sido optimista respecto a mi futuro
2. Por lo general me he sentido bien conmigo mismo

Convivencia y relaciones

P.20. Dígame ahora el grado de acuerdo con las siguientes frases **durante el periodo de confinamiento.** Utilice una escala del 1 al 5, donde 1 es nada de acuerdo y 5 totalmente de acuerdo. (No Procede constará para los ítems uno y cinco)

1. Las relaciones con algunas personas con las que convivo han empeorado
2. Las relaciones con algunas personas con las que NO convivo han empeorado
3. He tenido ganas de hablar porque estaba angustiado/a, pero no lo he hecho
4. He sentido que estaba más unido/a con el resto de la sociedad.
5. Me preocupa el nivel de estrés de mis hijos.

P.21. ¿Cómo le ha resultado hasta ahora la situación de confinamiento, muy fácil, bastante fácil, bastante difícil o muy difícil?

1. Muy fácil
2. Bastante fácil
3. Ni fácil ni difícil (No leer)
4. Bastante difícil
5. Muy difícil

Felicidad y apoyo social y afectivo

P.22. En términos generales, ¿en qué medida se considera Ud. una persona feliz o infeliz? Por favor, use una escala de 0 a 10, en la que 0 significa que se considera "completamente infeliz" y 10 que se considera "completamente feliz".

0 1 2 3 4 5 6 7 8 9 10 Ns/Nc 99

P.23. En la siguiente lista se muestran algunas cosas que otras personas hacen por nosotros o nos proporcionan. **Desde el principio del confinamiento**:

1. Tanto como deseo
2. Casi como deseo
3. Ni mucho ni poco
4. Menos de lo que deseo
5. Mucho menos de lo que deseo

P.23.1. Recibo visitas/llamadas de mis amigos y familiares
P.23.2. Recibo ayuda en asuntos relacionados con mi casa
P.23.3. Recibo elogios y reconocimientos cuando hago bien mi trabajo
P.23.4. Cuento con personas que se preocupan de lo que me sucede

P.23.5. Recibo amor y afecto
P.23.6. Tengo la posibilidad de hablar con alguien de mis problemas en el trabajo y/o en la casa
P.23.7. Tengo la posibilidad de hablar con alguien de mis problemas personales y familiares
P.23.8. Tengo posibilidad de hablar con alguien de mis problemas económicos
P.23.9. Recibo invitaciones para distraerme y salir con otras personas
P.23.10. Recibo consejos útiles cuando me ocurre algún acontecimiento importante en mi vida
P.23.11. Recibo ayuda cuando estoy enfermo/a en la cama

Molestias y enfermedades crónicas

P.24. Durante el **confinamiento**, ¿hasta qué punto ha notado Vd. alguna de las siguientes molestias? Utilice una escala del 1 al 5 en la que 1 es Nunca he sentido esa molestia y 5 Con mucha frecuencia.

1. Problemas de sueño
2. Problemas de estómago
3. Incremento de la tensión arterial
4. Mareos
5. Dolores de cabeza
6. Dolores musculares o de articulaciones
6a. Otros dolores
7. Sensación de opresión en el pecho
8. Otra molestia

Filtro si en los ítems 5, 6 o 6a han puntuado con 2, 3, 4 o 5 en P.24.
P.24.a. Alguno de estos dolores, ¿ha aparecido por primera vez durante el confinamiento?
1- Sí
2- No
9- NS/NC

P.25. ¿Le ha dicho un/a profesional de la salud (médico/a, enfermero/a) que usted padece actualmente alguna de las siguientes enfermedades crónicas? Si responde "SI" hacer P.25.a. ¿Esa condición le limita alguna de sus actividades?

ENFERMEDAD CRÓNICA	P.25.			P.25.a	
	¿Le ha dicho un/a profesional de la salud si tiene esta condición? (si es 'No', saltar a la siguiente)			¿Esa condición le limita alguna de sus actividades?	
	Sí	No	No lo sabe	Sí	No
1. Diabetes/azúcar alta en sangre/azúcar en orina	☐	☐	☐	☐	☐
2. Hipertensión/presión arterial alta	☐	☐	☐	☐	☐
3. Colesterol alto	☐	☐	☐	☐	☐
4. Enfermedad crónica del pulmón / enfisema / bronquitis crónica	☐	☐	☐	☐	☐
5. Asma	☐	☐	☐	☐	☐
6. Enfermedades del corazón (Trastornos cardíacos/insufiencia cardíaca /insuficiencia cardíaca congestiva; angina de pecho; Infarto de miocardio/ataque al corazón)	☐	☐	☐	☐	☐
7. Dolor crónico (pecho; espalda, cuello, hombro, cintura (cervical/lumbar; Migrañas/Jaquecas/ Cefaleas crónicas / dolor de cabeza frecuente)	☐	☐	☐	☐	☐

8. Alergias crónicas (como rinitis, inflamación de ojos, dermatitis, alergia a los alimentos, etc), asma alérgica excluida	☐	☐	☐	☐	☐
9. Mala circulación	☐	☐	☐	☐	☐
10. Varices en las piernas	☐	☐	☐	☐	☐
11. Trastornos depresivos	☐	☐	☐	☐	☐
12. Trastornos de ansiedad	☐	☐	☐	☐	☐
13. Otra enfermedad crónica	☐	☐	☐	☐	☐

Medicamentos

P.26. ¿En qué medida se ha visto modificada la medicación que usted tomaba antes del confinamiento con respecto a ahora?

1. He disminuido mi medicación
2. Sigo igual, no ha afectado en nada
3. He aumentado mi medicación (incluye si antes no consumía, pero ahora sí)
4. No consumo medicación.

Filtro si 1,2 o 3 en la P.26.

P.26.a. Y En general, ha cambiado la manera en que tomo mis medicamentos

1. Sí, ahora estoy cumpliendo mejor con la medicación
2. Sí, ahora estoy cumpliendo peor con la medicación
3. Sigo tomando los mismos medicamentos de la misma manera

P.27. (PARA TODOS). Y en las **últimas 2 semanas**, ¿ha utilizado algún tipo de medicamento (gotas, pastillas, inyecciones, supositorios, etc.)?

1. Sí
2. No
9. N/C

Filtro Si 1 en P.27.

P.27.a. Si ha tomado medicamentos, ¿Qué tipo de medicamento?

1. Medicinas para el catarro, gripe, garganta, bronquios
2. Medicinas para el dolor
3. Medicinas para para bajar la fiebre
4. Antibióticos
5. Tranquilizantes, sedantes
6. Pastillas para dormir
7. Medicamentos antidepresivos
8. Medicamentos para la alergia
9. Medicinas para el corazón
10. Medicinas para la tensión arterial
11. Medicamentos para la diabetes
12. Medicamentos para bajar el colesterol
13. Otros medicamentos

BLOQUE III: HÁBITOS Y ESTILOS DE VIDA

P.28. En relación con el **confinamiento**, diría Vd. que…

	Mas	Igual	Menos	No procede	Ns/Nc

• 28.1 He realizado más, igual o menos ejercicio	1	2	3	8	9
• 28.2 He consumido más, igual o menos bebidas alcohólicas	1	2	3	8	9
• 28.3 He fumado más, igual, o menos	1	2	3	8	9

	Mejor	Igual	Peor	Ns/Nc
ido mejor, igual o peor	1	2	3	9
• Me he alimentado mejor, igual o peor que antes	1	2	3	9

Mi peso ha aumentado, se ha mantenido o ha disminuido

1. Aumentado
2. Mantenido
3. Disminuido
9. Ns/Nc

Alimentación

P.29. ¿Cuántas raciones de verdura u hortalizas consume al día (las guarniciones o acompañamientos contabilizan como ½ ración)?

1. Dos o más al día (al menos una de ellas en ensalada o crudas)
2. Dos o más al día (aunque ninguna de ellas en ensalada o crudas)
3. Diariamente, aunque menos de dos al día
4. No diariamente, pero tres o más por semana
5. Menos de tres veces por semana
6. No consume
9. NS/NC

P.30. ¿Cuántas piezas de fruta (incluyendo zumo natural) consume al día?

1. Tres o más al día
2. Diariamente, aunque menos de tres veces al día
3. No diariamente, pero tres o más veces por semana
4. Menos de tres veces por semana
5. No consume
9. NS/NC

Actividad física

P.31. Durante las **últimas dos semanas**, ¿Cuántos días a la semana practica normalmente durante más de 30 minutos …? *(incluyendo desplazamientos al trabajo o actividades similares)*

Nº de días/sem

1. Caminar ………………………………………………………………………….. |_|_|
2. Una actividad física ligera como gimnasia suave, juegos que requieren poco esfuerzo y similares …….…………………………………………………….……….. |_|_|
3. Una actividad física moderada como montar en bicicleta, gimnasia, aeróbic, correr, natación ………………………………………………………………….… |_|_|
4. Entrenamiento físico deportivo como fútbol, baloncesto, ciclismo, natación de competición, judo, kárate o similares ……...…….…..... |_|_|

P.32. ¿Podría decirme cuánto pesa, aproximadamente, sin zapatos ni ropa? (si está embarazada en la actualidad, debe indicar cuánto pesaba antes del inicio del embarazo)

Kilos: |_|_|_|

No sabe 998
No contesta 999

P.33.¿Y cuánto mide, aproximadamente, sin zapatos?
Cms.: |_|_|_|
No sabe 998
No contesta 999

Tabaco

P.34. Cambiando de tema, ¿podría usted decirme si fuma?
1. Sí, fuma diariamente
2. Sí fuma, pero no diariamente
3. No fuma, pero ha fumado
4. No fuma ni ha fumado nunca de manera habitual
9. No contesta

Alcohol

P.35. ¿Ha bebido usted en estas dos últimas semanas alguna bebida alcohólica?
1. Sí, diariamente → pasar a P.35.a
2. Sí, pero no diariamente. → pasar a P.35.a
3. No
9. NS/NC

P.35.a. Durante las **últimas 2 semanas** ¿alguna vez ha tomado 6/5 (hombre/mujer, respectivamente) (5/4 PARA MAYORES DE 65 AÑOS) o más bebidas estándar en una misma ocasión? (Por "ocasión" entendemos tomar las bebidas en un intervalo aproximado de cuatro a seis horas)."
1. Sí, alguna vez
2. Sí, varias veces
3. No
8. No sabe
9. No contesta

P.36. Durante las últimas 2 semanas, ¿ha utilizado la bebida para calmar su ansiedad?

1. Totalmente cierto
2. Bastante cierto
3. Bastanto falso
4. Totalmente falso

Sueño

P.37. ¿Podría indicarme, aproximadamente, cuántas horas al día duerme habitualmente?
Horas: |_|_|
NS/NC 99

P.37.a. Las horas que duerme, ¿le permiten descansar lo suficiente?
1. Sí
2. No
8. N/S
9. N/C

Vacunas

P.38. Pasando a otro tema, querríamos saber si se ha vacunado de la gripe en la última campaña

1. Sí
2. No
3. NS/NC

BLOQUE IV. EFECTOS ECONÓMICOS Y CARACTERÍSTICAS SOCIODEMOGRÁFICAS Y SOCIOECONÓMICAS.

Situación laboral

Para poder clasificar las respuestas que usted nos ha dado necesitamos que nos conteste las siguientes preguntas.

P.40. ¿En qué situación de las siguientes se encuentra en la actualidad? (Si se encuentra en varias situaciones, indique solo la que considere principal).

1.Asalariado/a a tiempo completo.
2.Asalariado/a a tiempo completo en ERTE
3.Asalariado/a a tiempo parcial
4.Asalariado/a a tiempo parcial por ERTE
5.Empresario/a con asalariados/as
6.Empresario/a sin asalariados/as o trabajador/a independiente o miembro de cooperativa
7.Trabajos familiares no remunerados (ayudas familiares).
8.Parado/a
9.Estudiante
10.Jubilado/a, retirado/a o jubilado/a anticipado/a
11.Incapacitado/a permanente
12.Realizando tareas de voluntariado social
13.Dedicado/a a las labores del hogar, al cuidado de niños u otras personas. 10
14.Otra clase de inactividad económica
98.No sabe
98.No contesta

Filtro: P40=1,2,3,4

P.41. ¿Qué tipo de contrato tiene en la actualidad?

1.Contrato indefinido
2.Contrato temporal.

Para trabajadores: Filtro: P40=1,2,3,4,5,6,7

P.42. Respecto a la situación en la que desarrolla su trabajo ¿cómo es **actualmente**?

1. Trabajo desde casa
2. Trabajo fuera de casa
3. Trabajo desde casa y desde fuera de casa
4. Estoy de permiso para ocuparme de mis hijos
5. Estoy permiso para ocuparme de otros familiares
6. Estoy de vacaciones anuales retribuidas
7. Estoy de permiso no retribuido
8. Otra situación

Para trabajadores Filtro: P40=1,2,3,4,5,6,7

P.43. Asumiendo que su capacidad laboral en su mejor estado de salud tuviera un valor de 10 puntos, ¿cuántos puntos daría a su capacidad laboral actual? 0 (totalmente incapacitado) …10 (muy buena capacidad laboral)

0 1 2 3 4 5 6 7 8 9 10   Ns/Nc 99

Filtro: P40=8 o P31=8 de la primera oleada
P.44. Me ha dicho que está Vd. en situación de desempleo. ¿En cuál de las siguientes situaciones se encuentra actualmente?

1. Estoy desempleado por un ERTE debido al coronavirus.
2. Estoy desempleado por un despido debido al coronavirus (sin ERTE).
3. He tenido que cerrar mi negocio.
4. He tenido que cesar la actividad que me aportaba ingresos
5. Tenía opciones de comenzar en un trabajo, pero se han postpuesto/cancelado debido al estado de alarma
6. Mi situación de desempleo es previa al estado de alarma y no se ha visto alterada
7. Otras situaciones:

Identificación de la persona de referencia del hogar (la que aporta más ingresos)

P.46. ¿En su hogar quién es la persona que aporta más ingresos?

1. Yo (ir a P.50.)
2. Otra persona (ir a P47)
3. Otra persona y yo a partes iguales (ir a P.50.)

P.47. Y esa persona es...

1. Su cónyuge/pareja
2. Su padre
3. Su madre
4. Otra persona.
5. No Contesta

P.48. ¿Cuáles son los estudios terminados de más alto nivel de la persona que aporta más ingresos en el hogar?

1. Analfabetos/as y estudios primarios incompletos.
2. Educación primaria.
3. Primera etapa de la Educación Secundaria y similar.
4. Segunda etapa de la Educación Secundaria y similar
5. Bachillerato y FP de Grado Medio.
6. Educación postsecundaria no superior.
7. Formación Profesional de Grado Superior y títulos propios De universidades de duración igual o superior a 2 años.
8. Diplomados/as universitarios/as, títulos propios universitarios de Experto o especialista y similares (Grados universitarios de 240 créditos ECTS, Bolonia).
9. Licenciados, másteres y especialidades en Ciencias de la Salud por el sistema de residencia y similares. Grados universitarios de más 240 créditos ECTS (Bolonia).
10. Título de Doctorado.
11. No se puede codificar.

P.49. ¿En qué situación de las siguientes se encuentra en la actualidad la persona que aporta más ingresos al hogar? (Si se encuentra en varias situaciones, indique solo la que considere principal).

1. Asalariado/a a tiempo completo
2. Asalariado/a a tiempo completo en ERTE
3. Asalariado/a a tiempo parcial

4. Asalariado/a a tiempo parcial por ERTE
5. Empresario/a con asalariados/as
6. Empresario/a sin asalariados/as o trabajador/a independiente o miembro de cooperativa
7. Trabajos familiares no remunerados (ayudas familiares).
8. Parado/a
9. Estudiante
10. Jubilado/a, retirado/a o jubilado/a anticipado/a
11. Incapacitado/a permanente
12. Realizando tareas de voluntariado social
13. Dedicado/a a las labores del hogar, al cuidado de niños u otras personas.
14. Otra clase de inactividad económica
98. No sabe
99. No contesta

Filtro: P49=1 a 8
P.49.a. En su empleo principal o en su último empleo ¿cuál es/era la ocupación, profesión u oficio que desempeña/ba la persona que aporta más ingresos al hogar?

Precise lo más posible, por ejemplo: dependiente de comercio, mecánico de automóviles, tornero, peón de la construcción, operador de grúa, etc.). Entrevistador: Anote el literal y señale la opción que corresponda según la tabla de clasificación del manual. El informante debe especificar suficientemente para que se pueda codificar correctamente
Ocupación: ___________________

Ingresos y situación económica del hogar

P.50. Un hogar puede tener diferentes fuentes de ingresos y más de un miembro del hogar puede contribuir con sus ingresos. En relación con el total de ingresos de su hogar, ¿cómo suelen llegar a fin de mes?
1. Con mucha facilidad.
2. Con facilidad.
3. Con cierta facilidad.
4. Con cierta dificultad.
5. Con dificultad
6. Con mucha dificultad
7. No sabe.
8. No contesta.

P.51. Desde que comenzó el confinamiento ¿ha tenido en su hogar algún retraso en el pago de recibos como la hipoteca, el alquiler, o suministros como la luz, el agua, impuestos, etc.? (Respuesta múltiple).
1. Hipoteca
2. Alquiler
3. Cuotas de compras aplazadas
4. Cuotas de seguros
5. Impuestos (IBI, los relacionados con vehículos, etc.)
6. Pagos de recibos de agua, gas, calefacción, electricidad, comunidad
7. Tasa de autónomo
8. Pago de colegios
9. Actividades extraescolares
10. Otros
11. Ninguna

P.51 a ¿Se ha resuelto? Filtro si en p51=si

1. Sí
2. No

P.53. Dígame en qué intervalo, de los que le leeré a continuación, se encuentran los ingresos mensuales netos del conjunto de miembros de su hogar EN EL MES ANTERIOR

1. Sin ingresos (no leer)
2. Menos de 450 euros
3. De 451 a 900 euros
4. De 901 a 1.600 euros
5. De 1.601 a 2.500 euros
6. De 2.501 a 3.000 euros
7. Más de 3.000 euros

98. NS
98. NC

P52a. ¿Cuáles son los estudios terminados de más alto nivel de su padre?
P52b. ¿Y de su madre?

- Primarios o menos
- Secundarios
- Universitarios
- No sabe
- No contesta

Ya para terminar… sobre la preocupación en el futuro

P.54. ¿Hasta qué punto le preocupa que a usted o a su familia le afecte directamente en el futuro alguna de las siguientes situaciones que le voy a nombrar?

1. Mucho
2. Bastante
3. Algo
4. Poco
5. Nada
6. Ya me OCURRE / ha ocurrido.
7. No sabe.
8. No contesta
9. No procede

1. Contagiarse por coronavirus
2. Crisis de abastecimiento (luz, agua, telefonía, alimentos, medicamentos)
3. Pérdida de ingresos
4. Desempleo
5. Problemas con los estudios
6. Revueltas, Inseguridad
7. No recibir una la atención sanitaria que necesite

Observaciones de la entrevista: ____

Esto es todo, muchas gracias por su colaboración y por el tiempo que nos ha dedicado respondiendo esta encuesta. Le recuerdo que tiene a su disposición el número de teléfono **900 10 17 49** por si necesita resolver cualquier duda o información.

Medición 3 - ESSOCgeneral. Cuestionario muestra longitudinal

Este cuestionario se ha elaborado a partir del cuestionario de la 2ª medición de la ESSOC. En gris aparecen las preguntas que tienen un filtro asociado. La mayoría son preguntas ya recogidas en anteriores mediciones de la ESSOC. La estructura del cuestionario se mantiene en cuatro grandes bloques:

- BLOQUE I. CARACTERÍSTICAS DEL HOGAR Y LA VIVIENDA
- BLOQUE II. SALUD, BIENESTAR EMOCIONAL
- BLOQUE III: HÁBITOS Y ESTILOS DE VIDA
- BLOQUE IV. SITUACIÓN ECONÓMICA

BLOQUE I. CARACTERÍSTICAS DEL HOGAR Y LA VIVIENDA

P.1. Desde la realización de la anterior encuesta, el día <*fecha de la entrevista de la M2*>, ¿Ha cambiado su residencia a una vivienda diferente?

1. Sí → pasar a P.2.
2. No → pasar a P.6.
3. No lo recuerdo/No estoy seguro/a → pasar a P.2.

P.2. ¿Actualmente se encuentra en su vivienda habitual o reside en una vivienda distinta?

1. Habitual → P.3.
2. Segunda residencia → P.2.a
3. Otras: → P.2.a

P.2.a. ¿En qué municipio se encuentra actualmente: Municipio: _________

P.3. La vivienda en la que se encuentra actualmente es una:

1. Vivienda unifamiliar independiente, adosada o pareada
2. Piso o apartamento en un edificio con menos de 10 viviendas
3. Piso o apartamento en un edificio con 10 o más viviendas
4. Otro tipo de vivienda (Ej: Vivienda situada en un edificio destinado principalmente a otros fines barraca, cabaña, chabola, etc.) Especifique.
5. NS/NC

P.4. ¿Cuál es la superficie útil de la vivienda en la que se encuentra actualmente?

1. Menos de 46 m² útiles
2. Entre 46 y 60
3. Entre 61 y 75
4. Entre 76 y 90
5. Entre 91 y 120
6. Más de 120
7. No sabe
8. No contesta

P.6. A raíz de la última entrevista ¿ha cambiado el grupo de personas con las que convive?

1. Sí → pasar a P.7.

2. No →pasar a P.11.
3. No lo recuerdo/No estoy seguro/a → pasar a P.7.

P.7. Para conocer las condiciones en las que está actualmente me podría decir ¿qué número de personas residen actualmente incluyéndose usted?
Número de personas: |_|_|

P.7.a. ¿De estas personas cuántas son mayores de 60 años?...... |_|_|
P.7.b. ¿Y cuántas son menores de 16 años?... |_|_|
P.7.c. ¿De las personas menores de 16, cuántas son menores de 6 años? I_I_I

P.8. A efectos de caracterizar su situación de convivencia, ¿me podría decir cuál de las siguientes alternativas lo refleja mejor?

1. Hogar unipersonal
2. Padre o madre solo/a que conviva con algún hijo/a
3. Pareja sin hijos que convivan en el hogar
4. Pareja con hijos/as que convivan en el hogar
5. Otro tipo de hogar
6. NC

P.11. ¿Cuántas habitaciones tiene su vivienda? (no contar la cocina, los baños/lavabos o el comedor/sala de estar).
Número de habitaciones: |_|_|

P.12. Indique el número de personas (<16; 16-59; >=60) que presentan alguna de las condiciones que voy a leerle…

P.12.a. Tener alguna limitación, discapacidad de tipo físico, sensorial o psíquico o mental

1. SI, de que tramo de edad:
   - <16 |_|_|
   - 16-59 |_|_|
   - >=60 |_|_|
2. No

P.12.b. Requiere algún tipo de dedicación o de cuidados especiales

1. SI, de que tramo de edad:
   - <16 |_|_|
   - 16-59 |_|_|
   - >=60 |_|_|
2. No

BLOQUE II. SALUD, BIENESTAR EMOCIONAL

Identificación de la infección

P.13. En primer lugar, nos gustaría confirmar si… ¿tiene o ha tenido diagnóstico de coronavirus?

1. Sí →pasar a P.13.a
2. No
3. Estoy a la espera del resultado
4. No sabe
5. No contesta

Filtro si 1 en P.13

P.13.a. ¿Cómo de grave ha sido o es su proceso con la enfermedad?

1. Muy leve
2. Leve
3. Grave
4. Muy grave

P.14. ¿Le han hecho alguna vez, alguna de las siguientes pruebas para ver si estaba infectado por el coronavirus? (MULTIRRESPUESTA)

1. Muestra de la garganta o la nariz	Sí/No/NC
2. Muestra de sangre en el dedo	Sí/No/NC
3. Muestra de sangre en vena	Sí/No/NC

P.15. ¿Hay en su entorno alguien que tiene o ha tenido algún caso diagnosticado de infección por coronavirus? Respuesta múltiple

1. Persona/s de mi familia con la que resido en el hogar
2. Persona/s con la que resido en el hogar que no es familiar
3. Familiar/es que no conviven conmigo
4. Amigos, compañeros de trabajo/estudios, vecinos con lo que no resido en el hogar
5. Nadie de mi entorno
6. NC

Hablemos ahora de forma general de cómo se encuentra…

Autopercepción de salud

P.16. En la actualidad, en general diría que su salud es...

1. Excelente
2. Muy buena
3. Buena
4. Regular
5. Mala
6. No sabe/No está seguro.
7. No contesta.

P.17. En la actualidad, en general diría que su salud mental es...

1. Excelente
2. Muy buena
3. Buena
4. Regular
5. Mala
6. No sabe/No está seguro.
7. No contesta.

Bienestar emocional

P.18. A continuación, le voy a leer una lista de sentimientos o comportamientos que quizás haya tenido Vd. **durante la última semana**. Por favor, dígame con qué frecuencia, se ha sentido...

1. En ningún momento o en casi ningún momento
2. En algún momento,
3. Buena parte del tiempo,
4. Todo o casi todo el tiempo

5. No sabe

1. Deprimido/a
2. Feliz
3. Solo/a
4. Ha tenido la sensación de disfrutar de la vida
5. Rebosante de energía
6. Tranquilo/a y relajado/a

P.19. Si toma también como referencia **la última semana**, dígame Ud. su grado de acuerdo o desacuerdo con las siguientes frases.

1. Muy de acuerdo
2. De acuerdo
3. Ni de acuerdo ni en desacuerdo
4. En desacuerdo
5. Muy en desacuerdo
6. No sabe

1. He sido optimista respecto a mi futuro
2. Por lo general me he sentido bien conmigo mismo

<u>Felicidad y apoyo social y afectivo</u>

P.22. En términos generales, ¿en qué medida se considera Ud. una persona feliz o infeliz? Por favor, use una escala de 0 a 10, en la que 0 significa que se considera "completamente infeliz" y 10 que se considera "completamente feliz".

0 1 2 3 4 5 6 7 8 9 10 Ns/Nc 99

P.23. En la siguiente lista se muestran algunas cosas que otras personas hacen por nosotros o nos proporcionan. Elija para cada una la respuesta que mejor refleje su situación:

1. Tanto como deseo
2. Casi como deseo
3. Ni mucho ni poco
4. Menos de lo que deseo
5. Mucho menos de lo que deseo

P.23.1. Recibo visitas/llamadas de mis amigos y familiares
P.23.2. Recibo ayuda en asuntos relacionados con mi casa
P.23.3. Recibo elogios y reconocimientos cuando hago bien mi trabajo
P.23.4. Cuento con personas que se preocupan de lo que me sucede
P.23.5. Recibo amor y afecto
P.23.6. Tengo la posibilidad de hablar con alguien de mis problemas en el trabajo y/o en la casa
P.23.7. Tengo la posibilidad de hablar con alguien de mis problemas personales y familiares
P.23.8. Tengo posibilidad de hablar con alguien de mis problemas económicos
P.23.9. Recibo invitaciones para distraerme y salir con otras personas
P.23.10. Recibo consejos útiles cuando me ocurre algún acontecimiento importante en mi vida
P.23.11. Recibo ayuda cuando estoy enfermo/a en la cama

<u>Molestias y enfermedades crónicas</u>

P.24. Durante el **último mes**, ¿hasta qué punto ha notado Vd. alguna de las siguientes molestias? Utilice una escala del 1 al 5 en la que 1 es Nunca he sentido esa molestia y 5 Con mucha frecuencia.

1. Problemas de sueño
2. Problemas de estómago
3. Incremento de la tensión arterial
4. Mareos
5. Dolores de cabeza
6. Dolores musculares o de articulaciones
6a. Otros dolores
7. Sensación de opresión en el pecho
8. Otra molestia

P.25. ¿Le ha dicho un/a profesional de la salud (médico/a, enfermero/a) que usted padece actualmente alguna de las siguientes enfermedades crónicas? Si responde "SI" hacer P.25.a. ¿Esa condición le limita alguna de sus actividades?

ENFERMEDAD CRÓNICA	P.25. ¿Le ha dicho un/a profesional de la salud si tiene esta condición? (si es 'No', saltar a la siguiente)			P.25.a ¿Esa condición le limita alguna de sus actividades?	
	Sí	No	No lo sabe	Sí	No
1. Diabetes/azúcar alta en sangre/azúcar en orina	☐	☐	☐	☐	☐
2. Hipertensión/presión arterial alta	☐	☐	☐	☐	☐
3. Colesterol alto	☐	☐	☐	☐	☐
4. Enfermedad crónica del pulmón / enfisema / bronquitis crónica	☐	☐	☐	☐	☐
5. Asma	☐	☐	☐	☐	☐
6. Enfermedades del corazón (Trastornos cardíacos/insufiencia cardíaca /insuficiencia cardíaca congestiva; angina de pecho; Infarto de miocardio/ataque al corazón)	☐	☐	☐	☐	☐
7. Dolor crónico (pecho; espalda, cuello, hombro, cintura (cervical/lumbar; Migrañas/Jaquecas/ Cefaleas crónicas / dolor de cabeza frecuente)	☐	☐	☐	☐	☐
8. Alergias crónicas (como rinitis, inflamación de ojos, dermatitis, alergia a los alimentos, etc), asma alérgica excluida	☐	☐	☐	☐	☐
9. Mala circulación	☐	☐	☐	☐	☐
10. Varices en las piernas	☐	☐	☐	☐	☐
11. Trastornos depresivos	☐	☐	☐	☐	☐
12. Trastornos de ansiedad	☐	☐	☐	☐	☐
13. Otra enfermedad crónica	☐	☐	☐	☐	☐

Medicamentos

P.26. ¿En qué medida se ha visto modificada la medicación que usted tomaba antes del último mes con respecto a ahora?

1. He disminuido mi medicación
2. Sigo igual, no ha afectado en nada
3. He aumentado mi medicación (incluye si antes no consumía, pero ahora sí)
4. No consumo medicación.

Filtro si 1,2 o 3 en la P.26.

P.26.a. Y En general, ha cambiado la manera en que toma sus medicamentos

1. Sí, ahora estoy cumpliendo mejor con la medicación
2. Sí, ahora estoy cumpliendo peor con la medicación
3. Sigo tomando los mismos medicamentos de la misma manera

P.27. (PARA TODOS). Y en las **últimas 2 semanas**, ¿ha utilizado algún tipo de medicamento (gotas, pastillas, inyecciones, supositorios, etc.)?

1. Sí
2. No
9. NC

Filtro Si 1 en P.27.

P.27.a. Si ha tomado medicamentos, ¿Qué tipo de medicamento?

1. Medicinas para el catarro, gripe, garganta, bronquios
2. Medicinas para el dolor
3. Medicinas para para bajar la fiebre
4. Antibióticos
5. Tranquilizantes, sedantes
6. Pastillas para dormir
7. Medicamentos antidepresivos
8. Medicamentos para la alergia
9. Medicinas para el corazón
10. Medicinas para la tensión arterial
11. Medicamentos para la diabetes
12. Medicamentos para bajar el colesterol
13. Otros medicamentos

BLOQUE III: HÁBITOS Y ESTILOS DE VIDA

P.28. En relación al **último mes**, diría Vd. que...

1. He realizado más, igual o menos ejercicio
2. He consumido más, igual o menos bebidas alcohólicas
3. He fumado más, igual, o menos
4. He dormido mejor, igual o peor
5. Me he alimentado mejor, igual o peor que antes
6. Mi peso ha aumentado, se ha mantenido o ha disminuido

Alimentación

P.29. ¿Cuántas raciones de verdura u hortalizas consume al día (las guarniciones o acompañamientos contabilizan como ½ ración)?

1. Dos o más al día (al menos una de ellas en ensalada o crudas)
2. Dos o más al día (aunque ninguna de ellas en ensalada o crudas)
3. Diariamente, aunque menos de dos al día

4. No diariamente, pero tres o más por semana
5. Menos de tres veces por semana
6. No consume
9. NS/NC

P.30. ¿Cuántas piezas de fruta (incluyendo zumo natural) consume al día?
1. Tres o más al día
2. Diariamente, aunque menos de tres veces al día
3. No diariamente, pero tres o más veces por semana
4. Menos de tres veces por semana
5. No consume
9. NS/NC

Actividad física

P.31. Durante las **últimas dos semanas**, ¿Cuántos días a la semana practica normalmente durante más de 30 minutos …? *(incluyendo desplazamientos al trabajo o actividades similares)*

Nº de días/sem

1. Caminar ………………………………………………………………………….. |_|_|
2. Una actividad física ligera como gimnasia suave, juegos que requieren poco esfuerzo y similares …….…………………………………………………..………. |_|_|
3. Una actividad física moderada como montar en bicicleta, gimnasia, aeróbic, correr, natación ………………………………………………………………….… |_|_|
4. Entrenamiento físico deportivo como fútbol, baloncesto, ciclismo, natación de competición, judo, kárate o similares ……..……........ |_|_|

P.32. ¿Podría decirme cuánto pesa, aproximadamente, sin zapatos ni ropa? (si está embarazada en la actualidad, debe indicar cuánto pesaba antes del inicio del embarazo)

Kilos: |_|_|_|
No sabe 998
No contesta 999

P.33.¿Y cuánto mide, aproximadamente, sin zapatos?

Cms.: |_|_|_|
No sabe 998
No contesta 999

Tabaco

P.34. Cambiando de tema, ¿podría usted decirme si fuma?
1. Sí, fuma diariamente
2. Sí fuma, pero no diariamente
3. No fuma, pero ha fumado
4. No fuma ni ha fumado nunca de manera habitual
9. No contesta

Alcohol

P.35. ¿Ha bebido usted en estas dos últimas semanas alguna bebida alcohólica?

1. Sí, diariamente → pasar a P.35.a
2. Sí, pero no diariamente. → pasar a P.35.a
3. No

9. NS/NC

P.35.a. Durante las **últimas 2 semanas** ¿alguna vez ha tomado 6/5 (hombre/mujer, respectivamente) (5/4 PARA MAYORES DE 65 AÑOS) o más bebidas estándar en una misma ocasión? (Por "ocasión" entendemos tomar las bebidas en un intervalo aproximado de cuatro a seis horas)."

1. Sí, alguna vez
2. Sí, varias veces
3. No

8. No sabe
9. No contesta

P.36. Durante las últimas 2 semanas, ¿ha utilizado la bebida para calmar su ansiedad?

1. Totalmente cierto
2. Bastante cierto
3. Bastanto falso
4. Totalmente falso
5. NS/NC

Sueño

P.37. ¿Podría indicarme, aproximadamente, cuántas horas al día duerme habitualmente?

Horas:	|_|_|
NS/NC	99

P.37.a. Las horas que duerme, ¿le permiten descansar lo suficiente?

1. Sí
2. No

8. NS
9. NC

Vacunas

P.38. Pasando a otro tema, querríamos saber si se ha vacunado de la gripe en la última campaña 2019

1. Sí
2. No
3. NS/NC

P.38.a. ¿Y en la campaña de 2020?

1. Sí
2. No, pero tengo intención de vacunarme
3. No y no tengo intención de vacunarme
4. NS/NC

BLOQUE IV. EFECTOS ECONÓMICOS Y CARACTERÍSTICAS SOCIODEMOGRÁFICAS Y SOCIOECONÓMICAS.

Situación laboral

P.40. ¿En qué situación de las siguientes se encuentra en la actualidad? (Si se encuentra en varias situaciones, indique solo la que considere principal).

1.Asalariado/a a tiempo completo.

2.Asalariado/a a tiempo completo en ERTE
3.Asalariado/a a tiempo parcial
4.Asalariado/a a tiempo parcial por ERTE
5.Empresario/a con asalariados/as
6.Empresario/a sin asalariados/as o trabajador/a independiente o miembro de cooperativa
7.Trabajos familiares no remunerados (ayudas familiares).
8.Parado/a
9.Estudiante
10.Jubilado/a, retirado/a o jubilado/a anticipado/a
11.Incapacitado/a permanente
12.Realizando tareas de voluntariado social
13.Dedicado/a a las labores del hogar, al cuidado de niños u otras personas. 10
14.Otra clase de inactividad económica
98.No sabe
98.No contesta

P.40.a. En su empleo principal o en su último empleo ¿cuál es/era la ocupación, profesión u oficio que desempeña/ba?

Precise lo más posible, por ejemplo: dependiente de comercio, mecánico de automóviles, tornero, peón de la construcción, operador de grúa, etc.). Entrevistador: Anote el literal y señale la opción que corresponda según la tabla de clasificación del manual. El informante debe especificar suficientemente para que se pueda codificar correctamente.
Ocupación: ______________________

P.40.b. ¿Cuáles son sus estudios terminados de más alto nivel?

Anote el literal y señale la opción que corresponda. El informante debe especificar en su respuesta lo suficiente como para que se pueda codificar correctamente, por ejemplo: ha asistido menos de cinco años a la escuela, tres cursos completos de Ciencias Económicas y Empresariales, doctorado en Ciencias Sociales, etc.
Estudios terminados de más alto nivel: _______________________
Agente CATI: no leer. Clasificar en función del literal contestado

1. Analfabetos/as y estudios primarios incompletos.
2. Educación primaria.
3. Primera etapa de la Educación Secundaria y similar.
4. Segunda etapa de la Educación Secundaria y similar
5. Bachillerato y FP de Grado Medio.
6. Educación postsecundaria no superior.
7. Formación Profesional de Grado Superior y títulos propios De universidades de duración igual o superior a 2 años.
8. Diplomados/as universitarios/as, títulos propios universitarios de Experto o especialista y similares (Grados universitarios de 240 créditos ECTS, Bolonia).
9. Licenciados, másteres y especialidades en Ciencias de la Salud por el sistema de residencia y similares. Grados universitarios de más 240 créditos ECTS (Bolonia).
10. Título de Doctorado.
11. No se puede codificar.

Filtro: P40=1,2,3,4
P.41. ¿Qué tipo de contrato tiene en la actualidad?

1.Contrato indefinido
2.Contrato temporal.

Para trabajadores: Filtro: P40=1,2,3,4,5,6,7 o P31= 1,2,3,4,5,6,7 de la primera oleada

P.42. Respecto a la situación en la que desarrolla su trabajo ¿cómo es **actualmente**?

1. Trabajo desde casa
2. Trabajo fuera de casa
3. Trabajo desde casa y desde fuera de casa
4. Estoy de permiso para ocuparme de mis hijos
5. Estoy permiso para ocuparme de otros familiares
6. Estoy de vacaciones anuales retribuidas
7. Estoy de permiso no retribuido
8. Otra situación

Para trabajadores Filtro: P40=1,2,3,4,5,6,7

P.43. Asumiendo que su capacidad laboral en su mejor estado de salud tuviera un valor de 10 puntos, ¿cuántos puntos daría a su capacidad laboral actual? 0 (totalmente incapacitado) …10 (muy buena capacidad laboral)

0 1 2 3 4 5 6 7 8 9 10 Ns/Nc 99

Filtro: P40=8

P.44. Me ha dicho que está Vd. en situación de desempleo. ¿En cuál de las siguientes situaciones se encuentra actualmente?

1. Estoy desempleado por un ERTE debido al coronavirus.
2. Estoy desempleado por un despido debido al coronavirus (sin ERTE).
3. He tenido que cerrar mi negocio.
4. He tenido que cesar la actividad que me aportaba ingresos
5. Tenía opciones de comenzar en un trabajo, pero se han pospuesto/cancelado debido al estado de alarma
6. Mi situación de desempleo es previa al estado de alarma y no se ha visto alterada
7. Otras situaciones:

Identificación de la persona de referencia del hogar (la que aporta más ingresos)

P.46. Actualmente, ¿En su hogar quién es la persona que aporta más ingresos?

1. Yo (ir a P.50)
2. Otra persona (ir a P.47)
3. Otra persona y yo a partes iguales (ir a P.47)

P.47. Y esa persona es...

1. Su cónyuge/pareja
2. Su padre
3. Su madre
4. Otra persona.
5. No Contesta

P.48. ¿Cuáles son los estudios terminados de más alto nivel de la persona que aporta más ingresos en el hogar?

Anote el literal y señale la opción que corresponda. El informante debe especificar en su respuesta lo suficiente como para que se pueda codificar correctamente, por ejemplo: ha asistido menos de cinco años a la escuela, tres cursos completos de Ciencias Económicas y Empresariales, doctorado en Ciencias Sociales, etc.

Estudios terminados de más alto nivel: _______________________

Agente CATI: no leer. Clasificar en función del literal contestado

1. Analfabetos/as y estudios primarios incompletos.
2. Educación primaria.

3. Primera etapa de la Educación Secundaria y similar.
4. Segunda etapa de la Educación Secundaria y similar
5. Bachillerato y FP de Grado Medio.
6. Educación postsecundaria no superior.
7. Formación Profesional de Grado Superior y títulos propios De universidades de duración igual o superior a 2 años.
8. Diplomados/as universitarios/as, títulos propios universitarios de Experto o especialista y similares (Grados universitarios de 240 créditos ECTS, Bolonia).
9. Licenciados, másteres y especialidades en Ciencias de la Salud por el sistema de residencia y similares. Grados universitarios de más 240 créditos ECTS (Bolonia).
10. Título de Doctorado.
11. No se puede codificar.

P.49. ¿En qué situación de las siguientes se encuentra en la actualidad la persona que aporta más ingresos al hogar? (Si se encuentra en varias situaciones, indique solo la que considere principal).

1. Asalariado/a a tiempo completo
2. Asalariado/a a tiempo completo en ERTE
3. Asalariado/a a tiempo parcial
4. Asalariado/a a tiempo parcial por ERTE
5. Empresario/a con asalariados/as
6. Empresario/a sin asalariados/as o trabajador/a independiente o miembro de cooperativa
7. Trabajos familiares no remunerados (ayudas familiares).
8. Parado/a
9. Estudiante
10. Jubilado/a, retirado/a o jubilado/a anticipado/a
11. Incapacitado/a permanente
12. Realizando tareas de voluntariado social
13. Dedicado/a a las labores del hogar, al cuidado de niños u otras personas.
14. Otra clase de inactividad económica
98. No sabe
99. No contesta

P.49.a. En su empleo principal o en su último empleo ¿cuál es/era la ocupación, profesión u oficio que desempeña/ba la persona que aporta más ingresos al hogar?

Precise lo más posible, por ejemplo: dependiente de comercio, mecánico de automóviles, tornero, peón de la construcción, operador de grúa, etc.). Entrevistador: Anote el literal y señale la opción que corresponda según la tabla de clasificación del manual. El informante debe especificar suficientemente para que se pueda codificar correctamente
Ocupación: ___________________

Ingresos y situación económica del hogar

P.50. Un hogar puede tener diferentes fuentes de ingresos y más de un miembro del hogar puede contribuir con sus ingresos. En relación con el total de ingresos de su hogar, ¿cómo suelen llegar a fin de mes?

1. Con mucha facilidad.
2. Con facilidad.
3. Con cierta facilidad.
4. Con cierta dificultad.
5. Con dificultad

6. Con mucha dificultad
7. No sabe.
8. No contesta.

P.51. Desde que comenzó la crisis sanitaria ¿ha tenido en su hogar algún retraso en el pago de recibos como la hipoteca, el alquiler, o suministros como la luz, el agua, impuestos, etc.? (Respuesta múltiple).

1. Hipoteca
2. Alquiler
3. Cuotas de compras aplazadas
4. Cuotas de seguros
5. Impuestos (IBI, los relacionados con vehículos, etc.)
6. Pagos de recibos de agua, gas, calefacción, electricidad, comunidad
7. Tasa de autónomo
8. Pago de colegios
9. Actividades extraescolares
10. Otros
11. Ninguna

P.51.a. ¿Se ha resuelto? Filtro si en p51=si

1. Sí
2. No

P.52. ¿Ha cambiado su situación económica desde que realizó la primera encuesta?

1. Sí, ha mejorado → pasar a P.53.
2. Sí, ha empeorado→ pasar a P.53.
3. No → pasar a P.54.
4. No lo recuerdo/Ne estoy seguro/a → pasar a P.53.

P.53. Dígame en qué intervalo, de los que le leeré a continuación, se encuentran los ingresos mensuales netos del conjunto de miembros de su hogar EN EL MES ANTERIOR

1. Sin ingresos (no leer)
2. Menos de 450 euros
3. De 451 a 900 euros
4. De 901 a 1.600 euros
5. De 1.601 a 2.500 euros
6. De 2.501 a 3.000 euros
7. Más de 3.000 euros

98. NS
98. NC

Ya para terminar… sobre la preocupación en el futuro

P.54. ¿Hasta qué punto le preocupa que a usted o a su familia le afecte directamente en el futuro alguna de las siguientes situaciones que le voy a nombrar?

1. Mucho
2. Bastante
3. Algo
4. Poco
5. Nada
6. Ya me OCURRE / ha ocurrido.
7. No sabe.
8. No contesta
9. No procede

1. Contagiarse por coronavirus
2. Crisis de abastecimiento (luz, agua, telefonía, alimentos, medicamentos)
3. Pérdida de ingresos
4. Desempleo
5. Problemas con los estudios
6. Revueltas, Inseguridad
7. No recibir una la atención sanitaria que necesite

Esto es todo, muchas gracias por su colaboración y por el tiempo que nos ha dedicado respondiendo esta encuesta. Le recuerdo que tiene a su disposición el número de teléfono **900 10 17 49** por si necesita resolver cualquier duda o información.

Medición 3 - ESSOCgeneral. Cuestionario muestra nueva

Este cuestionario se ha elaborado fundamentalmente a partir del cuestionario de la muestra longitudinal de la 3ª medición de la ESSOC modificando aquellas preguntas que hacían referencia a la encuesta anterior. La estructura del cuestionario se mantiene en cuatro grandes bloques.

- BLOQUE I. CARACTERÍSTICAS DEL HOGAR Y LA VIVIENDA
- BLOQUE II. SALUD, BIENESTAR EMOCIONAL
- BLOQUE III: HÁBITOS Y ESTILOS DE VIDA
- BLOQUE IV. SITUACIÓN ECONÓMICA Y CARACTERÍSTICAS SOCIODEMOGRÁFICAS.

BLOQUE I. CARACTERÍSTICAS DEL HOGAR Y LA VIVIENDA

P.2. ¿Actualmente se encuentra en su vivienda habitual o reside en una vivienda distinta?

1. Habitual → P.3.
2. Segunda residencia → P.2.a
3. Otras: → P.2.a

P.2.a. ¿En qué municipio se encuentra actualmente: Municipio: _________

P.3. La vivienda en la que se encuentra actualmente es una:

1. Vivienda unifamiliar independiente, adosada o pareada
2. Piso o apartamento en un edificio con menos de 10 viviendas
3. Piso o apartamento en un edificio con 10 o más viviendas
4. Otro tipo de vivienda (Ej: Vivienda situada en un edificio destinado principalmente a otros fines barraca, cabaña, chabola, etc.) Especifique.
5. NS/NC

P.4. ¿Cuál es la superficie útil de la vivienda en la que se encuentra actualmente?

1. Menos de 46 m² útiles
2. Entre 46 y 60
3. Entre 61 y 75
4. Entre 76 y 90
5. Entre 91 y 120

6. Más de 120
7. No sabe
8. No contesta

P.7. Para conocer las condiciones en las que está actualmente me podría decir ¿qué número de personas residen actualmente incluyéndose usted?
Número de personas: |_|_|

P.7.a. ¿De estas personas cuántas son mayores de 60 años?...... |_|_|
P.7.b. ¿Y cuántas son menores de 16 años?... |_|_|
P.7.c. ¿De las personas menores de 16, cuántas son menores de 6 años? I_I_I

P.8. A efectos de caracterizar su situación de convivencia, ¿me podría decir cuál de las siguientes alternativas lo refleja mejor?

1. Hogar unipersonal
2. Padre o madre solo/a que conviva con algún hijo/a
3. Pareja sin hijos que convivan en el hogar
4. Pareja con hijos/as que convivan en el hogar
5. Otro tipo de hogar
6. NC

P.11. ¿Cuántas habitaciones tiene su vivienda? (no contar la cocina, los baños/lavabos o el comedor/sala de estar).
Número de habitaciones: |_|_|

P.12. Indique el número de personas (<16; 16-59; >=60) que presentan alguna de las condiciones que voy a leerle…

P.12.a. Tener alguna limitación, discapacidad de tipo físico, sensorial o psíquico o mental

1. SI, de que tramo de edad:
   - <16 |_|_|
   - 16-59 |_|_|
   - >=60 |_|_|
2. No

P.12.b. Requiere algún tipo de dedicación o de cuidados especiales

1. SI, de que tramo de edad:
   - <16 |_|_|
   - 16-59 |_|_|
   - >=60 |_|_|
2. No

BLOQUE II. SALUD, BIENESTAR EMOCIONAL

Identificación de la infección

P.13. En primer lugar ¿tiene o ha tenido diagnóstico de coronavirus?

1. Sí →pasar a P.13.a
2. No
3. Estoy a la espera del resultado
4. No sabe

5. No contesta

Filtro si 1 en P.13
P.13.a. ¿Cómo de grave ha sido o es su proceso con la enfermedad?
1. Muy leve
2. Leve
3. Grave
4. Muy grave

P.14. ¿Le han hecho alguna vez, alguna de las siguientes pruebas para ver si estaba infectado por el coronavirus? (MULTIRRESPUESTA)

1. Muestra de la garganta o la nariz	Sí/No/NC
2. Muestra de sangre en el dedo	Sí/No/NC
3. Muestra de sangre en vena	Sí/No/NC

P.15. ¿Hay en su entorno alguien que tiene o ha tenido algún caso diagnosticado de infección por coronavirus? Respuesta múltiple
1. Persona/s de mi familia con la que resido en el hogar
2. Persona/s con la que resido en el hogar que no es familiar
3. Familiar/es que no conviven conmigo
4. Amigos, compañeros de trabajo/estudios, vecinos con lo que no resido en el hogar
5. Nadie de mi entorno
6. NC

Hablemos ahora de forma general de cómo se encuentra…

Autopercepción de salud

P.16. En la actualidad, en general diría que su salud es…
1. Excelente
2. Muy buena
3. Buena
4. Regular
5. Mala
6. No sabe/No está seguro.
7. No contesta.

P.17. En la actualidad, en general diría que su salud mental es…
1. Excelente
2. Muy buena
3. Buena
4. Regular
5. Mala
6. No sabe/No está seguro.
7. No contesta.

Bienestar emocional

P.18. A continuación, le voy a leer una lista de sentimientos o comportamientos que quizás haya tenido Vd. **durante la última semana**. Por favor, dígame con qué frecuencia, se ha sentido…
1. En ningún momento o en casi ningún momento
2. En algún momento,
3. Buena parte del tiempo,

4. Todo o casi todo el tiempo
5. No sabe

1. Deprimido/a
2. Feliz
3. Solo/a
4. Ha tenido la sensación de disfrutar de la vida
5. Rebosante de energía
6. Tranquilo/a y relajado/a

P.19. Si toma también como referencia **la última semana**, dígame Ud. su grado de acuerdo o desacuerdo con las siguientes frases.

1. Muy de acuerdo
2. De acuerdo
3. Ni de acuerdo ni en desacuerdo
4. En desacuerdo
5. Muy en desacuerdo
6. No sabe

1. He sido optimista respecto a mi futuro
2. Por lo general me he sentido bien conmigo mismo

Felicidad y apoyo social y afectivo

P.22. En términos generales, ¿en qué medida se considera Ud. una persona feliz o infeliz? Por favor, use una escala de 0 a 10, en la que 0 significa que se considera "completamente infeliz" y 10 que se considera "completamente feliz".

0 1 2 3 4 5 6 7 8 9 10 Ns/Nc 99

P.23. En la siguiente lista se muestran algunas cosas que otras personas hacen por nosotros o nos proporcionan. Elija para cada una la respuesta que mejor refleje su situación:

1. Tanto como deseo
2. Casi como deseo
3. Ni mucho ni poco
4. Menos de lo que deseo
5. Mucho menos de lo que deseo

P.23.1. Recibo visitas/llamadas de mis amigos y familiares
P.23.2. Recibo ayuda en asuntos relacionados con mi casa
P.23.3. Recibo elogios y reconocimientos cuando hago bien mi trabajo
P.23.4. Cuento con personas que se preocupan de lo que me sucede
P.23.5. Recibo amor y afecto
P.23.6. Tengo la posibilidad de hablar con alguien de mis problemas en el trabajo y/o en la casa
P.23.7. Tengo la posibilidad de hablar con alguien de mis problemas personales y familiares
P.23.8. Tengo posibilidad de hablar con alguien de mis problemas económicos
P.23.9. Recibo invitaciones para distraerme y salir con otras personas
P.23.10. Recibo consejos útiles cuando me ocurre algún acontecimiento importante en mi vida
P.23.11. Recibo ayuda cuando estoy enfermo/a en la cama

Molestias y enfermedades crónicas

P.24. Durante el **último mes**, ¿hasta qué punto ha notado Vd. alguna de las siguientes molestias? Utilice una escala del 1 al 5 en la que 1 es Nunca he sentido esa molestia y 5 Con mucha frecuencia.

1. Problemas de sueño
2. Problemas de estómago
3. Incremento de la tensión arterial
4. Mareos
5. Dolores de cabeza
6. Dolores musculares o de articulaciones

6a. Otros dolores

7. Sensación de opresión en el pecho
8. Otra molestia

P.25. ¿Le ha dicho un/a profesional de la salud (médico/a, enfermero/a) que usted padece actualmente alguna de las siguientes enfermedades crónicas? Si responde "SI" hacer P.25.a. ¿Esa condición le limita alguna de sus actividades?

ENFERMEDAD CRÓNICA	P.25.			P.25.a	
	¿Le ha dicho un/a profesional de la salud si tiene esta condición? (si es 'No', saltar a la siguiente)			¿Esa condición le limita alguna de sus actividades?	
	Sí	No	No lo sabe	Sí	No
1. Diabetes/azúcar alta en sangre/azúcar en orina	☐	☐	☐	☐	☐
2. Hipertensión/presión arterial alta	☐	☐	☐	☐	☐
3. Colesterol alto	☐	☐	☐	☐	☐
4. Enfermedad crónica del pulmón / enfisema / bronquitis crónica	☐	☐	☐	☐	☐
5. Asma	☐	☐	☐	☐	☐
6. Enfermedades del corazón (Trastornos cardíacos/insufiencia cardíaca /insuficiencia cardíaca congestiva; angina de pecho; Infarto de miocardio/ataque al corazón)	☐	☐	☐	☐	☐
7. Dolor crónico (pecho; espalda, cuello, hombro, cintura (cervical/lumbar; Migrañas/Jaquecas/ Cefaleas crónicas / dolor de cabeza frecuente)	☐	☐	☐	☐	☐
8. Alergias crónicas (como rinitis, inflamación de ojos, dermatitis, alergia a los alimentos, etc), asma alérgica excluida	☐	☐	☐	☐	☐
9. Mala circulación	☐	☐	☐	☐	☐
10. Varices en las piernas	☐	☐	☐	☐	☐
11. Trastornos depresivos	☐	☐	☐	☐	☐
12. Trastornos de ansiedad	☐	☐	☐	☐	☐
13. Otra enfermedad crónica	☐	☐	☐	☐	☐

Medicamentos

P.26. ¿En qué medida se ha visto modificada la medicación que usted tomaba antes del último mes con respecto a ahora?

1. He disminuido mi medicación
2. Sigo igual, no ha afectado en nada
3. He aumentado mi medicación (incluye si antes no consumía, pero ahora sí)
4. No consumo medicación.

Filtro si 1,2 o 3 en la P.26.
P.26.a. Y En general, ha cambiado la manera en que toma sus medicamentos
1. Sí, ahora estoy cumpliendo mejor con la medicación
2. Sí, ahora estoy cumpliendo peor con la medicación
3. Sigo tomando los mismos medicamentos de la misma manera

P.27. (PARA TODOS). Y en las **últimas 2 semanas**, ¿ha utilizado algún tipo de medicamento (gotas, pastillas, inyecciones, supositorios, etc.)?
1. Sí
2. No
9. N/C

Filtro Si 1 en P.27.
P.27.a. Si ha tomado medicamentos, ¿Qué tipo de medicamento?
1. Medicinas para el catarro, gripe, garganta, bronquios
2. Medicinas para el dolor
3. Medicinas para para bajar la fiebre
4. Antibióticos
5. Tranquilizantes, sedantes
6. Pastillas para dormir
7. Medicamentos antidepresivos
8. Medicamentos para la alergia
9. Medicinas para el corazón
10. Medicinas para la tensión arterial
11. Medicamentos para la diabetes
12. Medicamentos para bajar el colesterol
13. Otros medicamentos

BLOQUE III: HÁBITOS Y ESTILOS DE VIDA

P.28. En relación al último mes, diría Vd. que...

	Mas	Igual	Menos	No procede	Ns/Nc
• 28.1 He realizado más, igual o menos ejercicio	1	2	3	8	9
• 28.2 He consumido más, igual o menos bebidas alcohólicas	1	2	3	8	9
• 28.3 He fumado más, igual, o menos	1	2	3	8	9

	Mejor	Igual	Peor	Ns/Nc
ido mejor, igual o peor	1	2	3	9
• Me he alimentado mejor, igual o peor que antes	1	2	3	9

Mi peso ha aumentado, se ha mantenido o ha disminuido
1. Aumentado

2. Mantenido
3. Disminuido
9. Ns/Nc

Alimentación

P.29. ¿Cuántas raciones de verdura u hortalizas consume al día (las guarniciones o acompañamientos contabilizan como ½ ración)?

1. Dos o más al día (al menos una de ellas en ensalada o crudas)
2. Dos o más al día (aunque ninguna de ellas en ensalada o crudas)
3. Diariamente, aunque menos de dos al día
4. No diariamente, pero tres o más por semana
5. Menos de tres veces por semana
6. No consume
9. NS/NC

P.30. ¿Cuántas piezas de fruta (incluyendo zumo natural) consume al día?

1. Tres o más al día
2. Diariamente, aunque menos de tres veces al día
3. No diariamente, pero tres o más veces por semana
4. Menos de tres veces por semana
5. No consume
9. NS/NC

Actividad física

P.31. Durante las **últimas dos semanas**, ¿Cuántos días a la semana practica normalmente durante más de 30 minutos …? *(incluyendo desplazamientos al trabajo o actividades similares)*

Nº de días/sem

1. Caminar ……………………………………………………………………………….. |_|_|
2. Una actividad física ligera como gimnasia suave, juegos que requieren poco esfuerzo y similares …….………………………………………………….………. |_|_|
3. Una actividad física moderada como montar en bicicleta, gimnasia, aeróbic, correr, natación …………………………………………………………………….… |_|_|
4. Entrenamiento físico deportivo como fútbol, baloncesto, ciclismo, natación de competición, judo, kárate o similares ……...……........ |_|_|

P.32. ¿Podría decirme cuánto pesa, aproximadamente, sin zapatos ni ropa? (si está embarazada en la actualidad, debe indicar cuánto pesaba antes del inicio del embarazo)

Kilos: |_|_|_|
No sabe 998
No contesta 999

P.33.¿Y cuánto mide, aproximadamente, sin zapatos?

Cms.: |_|_|_|
No sabe 998
No contesta 999

Tabaco

P.34. Cambiando de tema, ¿podría usted decirme si fuma?

1. Sí, fuma diariamente
2. Sí fuma, pero no diariamente
3. No fuma, pero ha fumado
4. No fuma ni ha fumado nunca de manera habitual
9. No contesta

Alcohol

P.35. ¿Ha bebido usted en estas dos últimas semanas alguna bebida alcohólica?
1. Sí, diariamente → pasar a P.35.a
2. Sí, pero no diariamente. → pasar a P.35.a
3. No
9. NS/NC

P.35.a. Durante las **últimas 2 semanas** ¿alguna vez ha tomado 6/5 (hombre/mujer, respectivamente) (5/4 PARA MAYORES DE 65 AÑOS) o más bebidas estándar en una misma ocasión? (Por "ocasión" entendemos tomar las bebidas en un intervalo aproximado de cuatro a seis horas)."
1. Sí, alguna vez
2. Sí, varias veces
3. No
8. No sabe
9. No contesta

P.36. Durante las últimas 2 semanas, ¿ha utilizado la bebida para calmar su ansiedad?

1. Totalmente cierto
2. Bastante cierto
3. Bastanto falso
4. Totalmente falso

Sueño

P.37. ¿Podría indicarme, aproximadamente, cuántas horas al día duerme habitualmente?

Horas:	|_|_|
NS/NC	99

P.37.a. Las horas que duerme, ¿le permiten descansar lo suficiente?
1. Sí
2. No
8. N/S
9. N/C

Vacunas

P.38. Pasando a otro tema, querríamos saber si se ha vacunado de la gripe en la última campaña 2019
1. Sí
2. No
3. NS/NC

P.38.a. ¿Y en la campaña de 2020?
1. Sí
2. No, pero tengo intención de vacunarme
3. No, y no tengo intención de vacunarme

4. NS/NC

BLOQUE IV. EFECTOS ECONÓMICOS Y CARACTERÍSTICAS SOCIODEMOGRÁFICAS Y SOCIOECONÓMICAS.

Situación laboral

Para poder clasificar las respuestas que usted nos ha dado necesitamos que nos conteste las siguientes preguntas.

P.40. ¿En qué situación de las siguientes se encuentra en la actualidad? (Si se encuentra en varias situaciones, indique solo la que considere principal).

1.Asalariado/a a tiempo completo.
2.Asalariado/a a tiempo completo en ERTE
3.Asalariado/a a tiempo parcial
4.Asalariado/a a tiempo parcial por ERTE
5.Empresario/a con asalariados/as
6.Empresario/a sin asalariados/as o trabajador/a independiente o miembro de cooperativa
7.Trabajos familiares no remunerados (ayudas familiares).
8.Parado/a
9.Estudiante
10.Jubilado/a, retirado/a o jubilado/a anticipado/a
11.Incapacitado/a permanente
12.Realizando tareas de voluntariado social
13.Dedicado/a a las labores del hogar, al cuidado de niños u otras personas. 10
14.Otra clase de inactividad económica
98.No sabe
98.No contesta

P.40.a. En su empleo principal o en su último empleo ¿cuál es/era la ocupación, profesión u oficio que desempeña/ba?

Precise lo más posible, por ejemplo: dependiente de comercio, mecánico de automóviles, tornero, peón de la construcción, operador de grúa, etc.). Entrevistador: Anote el literal y señale la opción que corresponda según la tabla de clasificación del manual. El informante debe especificar suficientemente para que se pueda codificar correctamente.
Ocupación: _____________

P.40.b. ¿Cuáles son sus estudios terminados de más alto nivel?

Anote el literal y señale la opción que corresponda. El informante debe especificar en su respuesta lo suficiente como para que se pueda codificar correctamente, por ejemplo: ha asistido menos de cinco años a la escuela, tres cursos completos de Ciencias Económicas y Empresariales, doctorado en Ciencias Sociales, etc.
Estudios terminados de más alto nivel: ______________________
Agente CATI: no leer. Clasificar en función del literal contestado

1. Analfabetos/as y estudios primarios incompletos.
2. Educación primaria.
3. Primera etapa de la Educación Secundaria y similar.
4. Segunda etapa de la Educación Secundaria y similar
5. Bachillerato y FP de Grado Medio.
6. Educación postsecundaria no superior.

7. Formación Profesional de Grado Superior y títulos propios De universidades de duración igual o superior a 2 años.
8. Diplomados/as universitarios/as, títulos propios universitarios de Experto o especialista y similares (Grados universitarios de 240 créditos ECTS, Bolonia).
9. Licenciados, másteres y especialidades en Ciencias de la Salud por el sistema de residencia y similares. Grados universitarios de más 240 créditos ECTS (Bolonia).
10. Título de Doctorado.
11. No se puede codificar.

Filtro: P40=1,2,3,4
P.41. ¿Qué tipo de contrato tiene en la actualidad?
1.Contrato indefinido
2.Contrato temporal.

Para trabajadores: Filtro: P40=1,2,3,4,5,6,7
P.42. Respecto a la situación en la que desarrolla su trabajo ¿cómo es **actualmente**?
1. Trabajo desde casa
2. Trabajo fuera de casa
3. Trabajo desde casa y desde fuera de casa
4. Estoy de permiso para ocuparme de mis hijos
5. Estoy permiso para ocuparme de otros familiares
6. Estoy de vacaciones anuales retribuidas
7. Estoy de permiso no retribuido
8. Otra situación

Para trabajadores Filtro: P40=1,2,3,4,5,6,7
P.43. Asumiendo que su capacidad laboral en su mejor estado de salud tuviera un valor de 10 puntos, ¿cuántos puntos daría a su capacidad laboral actual? 0 (totalmente incapacitado) …10 (muy buena capacidad laboral)

0 1 2 3 4 5 6 7 8 9 10 Ns/Nc 99

Filtro: P40=8
P.44. Me ha dicho que está Vd. en situación de desempleo. ¿En cuál de las siguientes situaciones se encuentra actualmente?
1. Estoy desempleado por un ERTE debido al coronavirus.
2. Estoy desempleado por un despido debido al coronavirus (sin ERTE).
3. He tenido que cerrar mi negocio.
4. He tenido que cesar la actividad que me aportaba ingresos
5. Tenía opciones de comenzar en un trabajo, pero se han pospuesto/cancelado debido al estado de alarma
6. Mi situación de desempleo es previa al estado de alarma y no se ha visto alterada
7. Otras situaciones:

Identificación de la persona de referencia del hogar (la que aporta más ingresos)

P.46. Actualmente, ¿En su hogar quién es la persona que aporta más ingresos?
1. Yo (ir a P.50.)
2. Otra persona (ir a P.47)
3. Otra persona y yo a partes iguales (ir a P.47)

P.47. Y esa persona es...
1. Su cónyuge/pareja
2. Su padre

3. Su madre
4. Otra persona.
5. No Contesta

P.48. ¿Cuáles son los estudios terminados de más alto nivel de la persona que aporta más ingresos en el hogar?

1. Analfabetos/as y estudios primarios incompletos.
2. Educación primaria.
3. Primera etapa de la Educación Secundaria y similar.
4. Segunda etapa de la Educación Secundaria y similar
5. Bachillerato y FP de Grado Medio.
6. Educación postsecundaria no superior.
7. Formación Profesional de Grado Superior y títulos propios De universidades de duración igual o superior a 2 años.
8. Diplomados/as universitarios/as, títulos propios universitarios de Experto o especialista y similares (Grados universitarios de 240 créditos ECTS, Bolonia).
9. Licenciados, másteres y especialidades en Ciencias de la Salud por el sistema de residencia y similares. Grados universitarios de más 240 créditos ECTS (Bolonia).
10. Título de Doctorado.
11. No se puede codificar.

P.49. ¿En qué situación de las siguientes se encuentra en la actualidad la persona que aporta más ingresos al hogar? (Si se encuentra en varias situaciones, indique solo la que considere principal).

1. Asalariado/a a tiempo completo
2. Asalariado/a a tiempo completo en ERTE
3. Asalariado/a a tiempo parcial
4. Asalariado/a a tiempo parcial por ERTE
5. Empresario/a con asalariados/as
6. Empresario/a sin asalariados/as o trabajador/a independiente o miembro de cooperativa
7. Trabajos familiares no remunerados (ayudas familiares).
8. Parado/a
9. Estudiante
10. Jubilado/a, retirado/a o jubilado/a anticipado/a
11. Incapacitado/a permanente
12. Realizando tareas de voluntariado social
13. Dedicado/a a las labores del hogar, al cuidado de niños u otras personas.
14. Otra clase de inactividad económica
98. No sabe
99. No contesta

P.49.a. En su empleo principal o en su último empleo ¿cuál es/era la ocupación, profesión u oficio que desempeña/ba la persona que aporta más ingresos al hogar?

Precise lo más posible, por ejemplo: dependiente de comercio, mecánico de automóviles, tornero, peón de la construcción, operador de grúa, etc.). Entrevistador: Anote el literal y señale la opción que corresponda según la tabla de clasificación del manual. El informante debe especificar suficientemente para que se pueda codificar correctamente
Ocupación: ___________________

Ingresos y situación económica del hogar

P.50. Un hogar puede tener diferentes fuentes de ingresos y más de un miembro del hogar puede contribuir con sus ingresos. En relación con el total de ingresos de su hogar, ¿cómo suelen llegar a fin de mes?

1. Con mucha facilidad.
2. Con facilidad.
3. Con cierta facilidad.
4. Con cierta dificultad.
5. Con dificultad
6. Con mucha dificultad
7. No sabe.
8. No contesta.

P.51. Desde que comenzó la crisis sanitaria, ¿ha tenido en su hogar algún retraso en el pago de recibos como la hipoteca, el alquiler, o suministros como la luz, el agua, impuestos, etc.? (Respuesta múltiple).

1. Hipoteca
2. Alquiler
3. Cuotas de compras aplazadas
4. Cuotas de seguros
5. Impuestos (IBI, los relacionados con vehículos, etc.)
6. Pagos de recibos de agua, gas, calefacción, electricidad, comunidad
7. Tasa de autónomo
8. Pago de colegios
9. Actividades extraescolares
10. Otros
11. Ninguna

P.51 a ¿Se ha resuelto? Filtro si en p51=si

1. Sí
2. No

P.53. Dígame en qué intervalo, de los que le leeré a continuación, se encuentran los ingresos mensuales netos del conjunto de miembros de su hogar EN EL MES ANTERIOR

1. Sin ingresos (no leer)
2. Menos de 450 euros
3. De 451 a 900 euros
4. De 901 a 1.600 euros
5. De 1.601 a 2.500 euros
6. De 2.501 a 3.000 euros
7. Más de 3.000 euros

98. NS

98. NC

P52a. ¿Cuáles son los estudios terminados de más alto nivel de su padre?
P52b. ¿Y de su madre?

- Primarios o menos
- Secundarios
- Universitarios
- No sabe
- No contesta

Ya para terminar… sobre la preocupación en el futuro

P.54. ¿Hasta qué punto le preocupa que a usted o a su familia le afecte directamente en el futuro alguna de las siguientes situaciones que le voy a nombrar?

1. Mucho
2. Bastante
3. Algo
4. Poco
5. Nada
6. Ya me OCURRE / ha ocurrido.
7. No sabe.
8. No contesta
9. No procede

   1. Contagiarse por coronavirus
   2. Crisis de abastecimiento (luz, agua, telefonía, alimentos, medicamentos)
   3. Pérdida de ingresos
   4. Desempleo
   5. Problemas con los estudios
   6. Revueltas, Inseguridad
   7. No recibir una la atención sanitaria que necesite

Observaciones de la entrevista: ____

Esto es todo, muchas gracias por su colaboración y por el tiempo que nos ha dedicado respondiendo esta encuesta. Le recuerdo que tiene a su disposición el número de teléfono **900 10 17 49** por si necesita resolver cualquier duda o información.

Medición 4 - ESSOCgeneral. Cuestionario muestra longitudinal

Este cuestionario se ha elaborado a partir del cuestionario de la 3ª medición de la ESSOC. En gris aparecen las preguntas que tienen un filtro asociado. La mayoría son preguntas ya recogidas en anteriores mediciones de la ESSOC. La estructura del cuestionario se mantiene en cuatro grandes bloques:

- BLOQUE I. CARACTERÍSTICAS DEL HOGAR Y LA VIVIENDA
- BLOQUE II. SALUD, BIENESTAR EMOCIONAL
- BLOQUE III: HÁBITOS Y ESTILOS DE VIDA
- BLOQUE IV. SITUACIÓN ECONÓMICA Y CARACTERÍSTICAS SOCIODEMOGRÁFICAS

BLOQUE I. CARACTERÍSTICAS DEL HOGAR Y LA VIVIENDA

P.1. Desde la realización de la anterior encuesta, el día <*fecha de la entrevista de la M3*>, ¿Ha cambiado su residencia a una vivienda diferente?

1. Sí → pasar a P.2.
2. No → pasar a P.6.
3. No lo recuerdo/No estoy seguro/a → pasar a P.2.

P.2. ¿Actualmente se encuentra en su vivienda habitual o reside en una vivienda distinta?

1. Habitual → P.3.
2. Segunda residencia → P.2.a
3. Otras: → P.2.a

P.2.a. ¿En qué municipio se encuentra actualmente: Municipio: _________

P.3. La vivienda en la que se encuentra actualmente es una:

1. Vivienda unifamiliar independiente, adosada o pareada
2. Piso o apartamento en un edificio con menos de 10 viviendas
3. Piso o apartamento en un edificio con 10 o más viviendas
4. Otro tipo de vivienda (Ej: Vivienda situada en un edificio destinado principalmente a otros fines barraca, cabaña, chabola, etc.) Especifique.
5. NS/NC

P.4. ¿Cuál es la superficie útil de la vivienda en la que se encuentra actualmente?

1. Menos de 46 m^2 útiles
2. Entre 46 y 60
3. Entre 61 y 75
4. Entre 76 y 90
5. Entre 91 y 120
6. Más de 120
7. No sabe
8. No contesta

P.6. A raíz de la última entrevista ¿ha cambiado el grupo de personas con las que convive?

1. Sí → pasar a P.7.
2. No →pasar a P.11.
3. No lo recuerdo/No estoy seguro/a → pasar a P.7.

P.7. Para conocer las condiciones en las que está actualmente me podría decir ¿qué número de personas residen actualmente incluyéndose usted?
Número de personas: |_|_|

P.7.a. ¿De estas personas cuántas son mayores de 60 años?...... |_|_|
P.7.b. ¿Y cuántas son menores de 16 años?... |_|_|
P.7.c. ¿De las personas menores de 16, cuántas son menores de 6 años? I_I_I

P.8. A efectos de caracterizar su situación de convivencia, ¿me podría decir cuál de las siguientes alternativas lo refleja mejor?

1. Hogar unipersonal
2. Padre o madre solo/a que conviva con algún hijo/a
3. Pareja sin hijos que convivan en el hogar
4. Pareja con hijos/as que convivan en el hogar
5. Otro tipo de hogar
6. NC

(incorporar el filtro si P1=1)

P.11. ¿Cuántas habitaciones tiene su vivienda? (no contar la cocina, los baños/lavabos o el comedor/sala de estar).
Número de habitaciones: |_|_|

P6a. ¿Y han cambiado las condiciones de limitación o discapacidad de algún miembro del hogar desde la última entrevista?

1. Sí → pasar a P.12.

2. No →pasar a P.12.C.
3. No lo recuerdo/No estoy seguro/a → pasar a P.12.

(incorporar el filtro si P6a=1o3)

P.12. Indique el número de personas (<16; 16-59; >=60) que presentan alguna de las condiciones que voy a leerle…

P.12.a. Tener alguna limitación, discapacidad de tipo físico, sensorial o psíquico o mental

1. SI, de que tramo de edad:
   - <16 |_|_|
   - 16-59 |_|_|
   - >=60 |_|_|
2. No

P.12.b. Requiere algún tipo de dedicación o de cuidados especiales

1. SI, de que tramo de edad:
   - <16 |_|_|
   - 16-59 |_|_|
   - >=60 |_|_|
2. No

<u>Cuidado y uso del tiempo</u>

P.12.C. Por favor, indique quienes de las siguientes personas realizan las siguientes acciones en su hogar actualmente:

1. *Siempre yo*
2. *Habitualmente yo*
3. *Mi pareja y yo por igual*
4. *Habitualmente mi pareja*
5. *Siempre mi pareja*
6. *Madre de la persona entrevistada*
7. *Padre de la persona entrevistada*
8. *Persona remunerada*
9. *Otras personas. Especificar*
10. *No sabe/No procede*

1. Tareas domésticas (limpieza, cocina, etc…)
2. Tareas de cuidado de hijos/as u otros menores del hogar (general):

   a. Tareas de cuidado físico (alimentación, vestido, higiene, vigilancia)
   b. Tareas de cuidado interactivo (enseñar, leer, jugar, conversar y otras interacciones)
   c. Acompañamiento y transporte de hijos/as a diferentes espacios

3. Tareas de cuidados en adultos con problemas de salud, movilidad, etc. que requieren algún tipo de dedicación o de cuidados especiales
4. Tareas en menores con necesidades especiales de cuidado (problemas de salud, movilidad, etc. que requieren algún tipo de dedicación o de cuidados especiales).

BLOQUE II. SALUD, BIENESTAR EMOCIONAL

Identificación de la infección

P.13. En primer lugar, nos gustaría confirmar si… ¿tiene o ha tenido diagnóstico de coronavirus?

1. Sí →pasar a P.13.a
2. No
3. Estoy a la espera del resultado
4. No sabe
5. No contesta

Filtro si 1 en P.13

P.13.a. ¿Cómo de grave ha sido o es su proceso con la enfermedad?

1. Muy leve
2. Leve
3. Grave
4. Muy grave

P.14. ¿Le han hecho alguna vez, alguna de las siguientes pruebas para ver si estaba infectado por el coronavirus? (MULTIRRESPUESTA)

1. Muestra de la garganta o la nariz	Sí/No/NC
2. Muestra de sangre en el dedo	Sí/No/NC
3. Muestra de sangre en vena	Sí/No/NC

P.15. ¿Hay en su entorno alguien que tiene o ha tenido algún caso diagnosticado de infección por coronavirus? Respuesta múltiple

1. Persona/s de mi familia con la que resido en el hogar
2. Persona/s con la que resido en el hogar que no es familiar
3. Familiar/es que no conviven conmigo
4. Amigos, compañeros de trabajo/estudios, vecinos con lo que no resido en el hogar
5. Nadie de mi entorno
6. NC

Hablemos ahora de forma general de cómo se encuentra…

Autopercepción de salud

P.16. En la actualidad, en general diría que su salud es...

1. Excelente
2. Muy buena
3. Buena
4. Regular
5. Mala
6. No sabe/No está seguro.
7. No contesta.

P.17. En la actualidad, en general diría que su salud mental es...

1. Excelente
2. Muy buena
3. Buena
4. Regular
5. Mala
6. No sabe/No está seguro.

7. No contesta.

Bienestar emocional

P.18. A continuación, le voy a leer una lista de sentimientos o comportamientos que quizás haya tenido Vd. **durante la última semana**. Por favor, dígame con qué frecuencia, se ha sentido...

1. En ningún momento o en casi ningún momento
2. En algún momento,
3. Buena parte del tiempo,
4. Todo o casi todo el tiempo
5. No sabe

1. Deprimido/a
2. Feliz
3. Solo/a
4. Ha tenido la sensación de disfrutar de la vida
5. Rebosante de energía
6. Tranquilo/a y relajado/a

P.19. Si toma también como referencia **la última semana**, dígame Ud. su grado de acuerdo o desacuerdo con las siguientes frases.

1. Muy de acuerdo
2. De acuerdo
3. Ni de acuerdo ni en desacuerdo
4. En desacuerdo
5. Muy en desacuerdo
6. No sabe

1. He sido optimista respecto a mi futuro
2. Por lo general me he sentido bien conmigo mismo

Felicidad y apoyo social y afectivo

P.22. En términos generales, ¿en qué medida se considera Ud. una persona feliz o infeliz? Por favor, use una escala de 0 a 10, en la que 0 significa que se considera "completamente infeliz" y 10 que se considera "completamente feliz".

0 1 2 3 4 5 6 7 8 9 10 Ns/Nc 99

P.23. En la siguiente lista se muestran algunas cosas que otras personas hacen por nosotros o nos proporcionan. Elija para cada una la respuesta que mejor refleje su situación:

1. Tanto como deseo
2. Casi como deseo
3. Ni mucho ni poco
4. Menos de lo que deseo
5. Mucho menos de lo que deseo

P.23.1. Recibo visitas/llamadas de mis amigos y familiares
P.23.2. Recibo ayuda en asuntos relacionados con mi casa
P.23.3. Recibo elogios y reconocimientos cuando hago bien mi trabajo
P.23.4. Cuento con personas que se preocupan de lo que me sucede
P.23.5. Recibo amor y afecto

P.23.6. Tengo la posibilidad de hablar con alguien de mis problemas en el trabajo y/o en la casa
P.23.7. Tengo la posibilidad de hablar con alguien de mis problemas personales y familiares
P.23.8. Tengo posibilidad de hablar con alguien de mis problemas económicos
P.23.9. Recibo invitaciones para distraerme y salir con otras personas
P.23.10. Recibo consejos útiles cuando me ocurre algún acontecimiento importante en mi vida
P.23.11. Recibo ayuda cuando estoy enfermo/a en la cama

Molestias y enfermedades crónicas

P.24. Durante el **último mes**, ¿hasta qué punto ha notado Vd. alguna de las siguientes molestias? Utilice una escala del 1 al 5 en la que 1 es Nunca he sentido esa molestia y 5 Con mucha frecuencia.

1. Problemas de sueño
2. Problemas de estómago
3. Incremento de la tensión arterial
4. Mareos
5. Dolores de cabeza
6. Dolores musculares o de articulaciones

6a. Otros dolores

7. Sensación de opresión en el pecho
8. Otra molestia

P.25. ¿Le ha dicho un/a profesional de la salud (médico/a, enfermero/a) que usted padece actualmente alguna de las siguientes enfermedades crónicas? Si responde "SI" hacer P.25.a. ¿Esa condición le limita alguna de sus actividades?

ENFERMEDAD CRÓNICA	P.25.			P.25.a	
	¿Le ha dicho un/a profesional de la salud si tiene esta condición? (si es 'No', saltar a la siguiente)			¿Esa condición le limita alguna de sus actividades?	
	Sí	No	No lo sabe	Sí	No
1. Diabetes/azúcar alta en sangre/azúcar en orina	☐	☐	☐	☐	☐
2. Hipertensión/presión arterial alta	☐	☐	☐	☐	☐
3. Colesterol alto	☐	☐	☐	☐	☐
4. Enfermedad crónica del pulmón / enfisema / bronquitis crónica	☐	☐	☐	☐	☐
5. Asma	☐	☐	☐	☐	☐
6. Enfermedades del corazón (Trastornos cardíacos/insufiencia cardíaca /insuficiencia cardíaca congestiva; angina de pecho; Infarto de miocardio/ataque al corazón)	☐	☐	☐	☐	☐
7. Dolor crónico (pecho; espalda, cuello, hombro, cintura (cervical/lumbar; Migrañas/Jaquecas/ Cefaleas crónicas / dolor de cabeza frecuente)	☐	☐	☐	☐	☐
8. Alergias crónicas (como rinitis, inflamación de ojos, dermatitis, alergia a los alimentos, etc), asma alérgica excluida	☐	☐	☐	☐	☐
9. Mala circulación	☐	☐	☐	☐	☐

10. Varices en las piernas	☐	☐	☐	☐	☐
11. Trastornos depresivos	☐	☐	☐	☐	☐
12. Trastornos de ansiedad	☐	☐	☐	☐	☐
13. Otra enfermedad crónica	☐	☐	☐	☐	☐

Medicamentos

P.26. ¿En qué medida se ha visto modificada la medicación que usted tomaba antes del último mes con respecto a ahora?

1. He disminuido mi medicación
2. Sigo igual, no ha afectado en nada
3. He aumentado mi medicación (incluye si antes no consumía, pero ahora sí)
4. No consumo medicación.

Filtro si 1,2 o 3 en la P.26.

P.26.a. Y En general, ha cambiado la manera en que toma sus medicamentos

1. Sí, ahora estoy cumpliendo mejor con la medicación
2. Sí, ahora estoy cumpliendo peor con la medicación
3. Sigo tomando los mismos medicamentos de la misma manera

P.27. (PARA TODOS). Y en las **últimas 2 semanas**, ¿ha utilizado algún tipo de medicamento (gotas, pastillas, inyecciones, supositorios, etc.)?

1. Sí
2. No
9. NC

Filtro Si 1 en P.27.

P.27.a. Si ha tomado medicamentos, ¿Qué tipo de medicamento?

1. Medicinas para el catarro, gripe, garganta, bronquios
2. Medicinas para el dolor
3. Medicinas para para bajar la fiebre
4. Antibióticos
5. Tranquilizantes, sedantes
6. Pastillas para dormir
7. Medicamentos antidepresivos
8. Medicamentos para la alergia
9. Medicinas para el corazón
10. Medicinas para la tensión arterial
11. Medicamentos para la diabetes
12. Medicamentos para bajar el colesterol
13. Otros medicamentos

BLOQUE III: HÁBITOS Y ESTILOS DE VIDA

P.28. En relación al **último mes**, diría Vd. que…

1. He realizado más, igual o menos ejercicio
2. He consumido más, igual o menos bebidas alcohólicas
3. He fumado más, igual, o menos
4. He dormido mejor, igual o peor
5. Me he alimentado mejor, igual o peor que antes
6. Mi peso ha aumentado, se ha mantenido o ha disminuido

Alimentación

P.29. ¿Cuántas raciones de verdura u hortalizas consume al día (las guarniciones o acompañamientos contabilizan como ½ ración)?

1. Dos o más al día (al menos una de ellas en ensalada o crudas)
2. Dos o más al día (aunque ninguna de ellas en ensalada o crudas)
3. Diariamente, aunque menos de dos al día
4. No diariamente, pero tres o más por semana
5. Menos de tres veces por semana
6. No consume
9. NS/NC

P.30. ¿Cuántas piezas de fruta (incluyendo zumo natural) consume al día?

1. Tres o más al día
2. Diariamente, aunque menos de tres veces al día
3. No diariamente, pero tres o más veces por semana
4. Menos de tres veces por semana
5. No consume
9. NS/NC

Actividad física

P.31. Durante las **últimas dos semanas**, ¿Cuántos días a la semana practica normalmente durante más de 30 minutos …? *(incluyendo desplazamientos al trabajo o actividades similares)*

	Nº de días/sem
1. Caminar ………………………………………………………………………………..	|__|__|
2. Una actividad física ligera como gimnasia suave, juegos que requieren poco esfuerzo y similares …….…………………………………………………….……….	|__|__|
3. Una actividad física moderada como montar en bicicleta, gimnasia, aeróbic, correr, natación ………………………………………………………………….…	|__|__|
4. Entrenamiento físico deportivo como fútbol, baloncesto, ciclismo, natación de competición, judo, kárate o similares ……...................................……........	|__|__|

P.32. ¿Podría decirme cuánto pesa, aproximadamente, sin zapatos ni ropa? (si está embarazada en la actualidad, debe indicar cuánto pesaba antes del inicio del embarazo)

Kilos: |_|_|_|
No sabe 998
No contesta 999

P.33.¿Y cuánto mide, aproximadamente, sin zapatos?

Cms.: |_|_|_|
No sabe 998
No contesta 999

Tabaco

P.34. Cambiando de tema, ¿podría usted decirme si fuma?

1. Sí, fuma diariamente
2. Sí fuma, pero no diariamente
3. No fuma, pero ha fumado

4. No fuma ni ha fumado nunca de manera habitual
9. No contesta

Alcohol

P.35. ¿Ha bebido usted en estas dos últimas semanas alguna bebida alcohólica?

1. Sí, diariamente → pasar a P.35.a
2. Sí, pero no diariamente. → pasar a P.35.a
3. No
9. NS/NC

P.35.a. Durante las **últimas 2 semanas** ¿alguna vez ha tomado 6/5 (hombre/mujer, respectivamente) (5/4 PARA MAYORES DE 65 AÑOS) o más bebidas estándar en una misma ocasión? (Por "ocasión" entendemos tomar las bebidas en un intervalo aproximado de cuatro a seis horas)."

1. Sí, alguna vez
2. Sí, varias veces
3. No
8. No sabe
9. No contesta

P.36. Durante las últimas 2 semanas, ¿ha utilizado la bebida para calmar su ansiedad?

1. Totalmente cierto
2. Bastante cierto
3. Bastanto falso
4. Totalmente falso
5. NS/NC

Sueño

P.37. ¿Podría indicarme, aproximadamente, cuántas horas al día duerme habitualmente?

Horas: |_|_|
NS/NC 99

P.37.a. Las horas que duerme, ¿le permiten descansar lo suficiente?

1. Sí
2. No
8. NS
9. NC

Vacunas

P.38. Pasando a otro tema, querríamos saber si se ha vacunado de la gripe en la última campaña 2019

1. Sí
2. No
3. NS/NC

P.38.a. ¿Y en la campaña de 2020?

1. Sí
2. No, pero tengo intención de vacunarme
3. No y no tengo intención de vacunarme

4. NS/NC

P.38.b. ¿Y se ha vacunado de COVID-19?

1. Sí
2. No, pero tengo intención de vacunarme
3. No y no tengo intención de vacunarme
4. NS/NC

BLOQUE IV. EFECTOS ECONÓMICOS Y CARACTERÍSTICAS SOCIODEMOGRÁFICAS Y SOCIOECONÓMICAS.

<u>Situación laboral</u>

P.39. ¿Ha cambiado su situación laboral desde que realizó la última encuesta?

1. Sí → pasar a P.40.
2. No → pasar a P.39.a.
3. No lo recuerdo/No estoy seguro/a → pasar a P.40.

P.40. ¿En qué situación de las siguientes se encuentra en la actualidad? (Si se encuentra en varias situaciones, indique solo la que considere principal).

1.Asalariado/a a tiempo completo.
2.Asalariado/a a tiempo completo en ERTE
3.Asalariado/a a tiempo parcial
4.Asalariado/a a tiempo parcial por ERTE
5.Empresario/a con asalariados/as
6.Empresario/a sin asalariados/as o trabajador/a independiente o miembro de cooperativa
7.Trabajos familiares no remunerados (ayudas familiares).
8.Parado/a
9.Estudiante
10.Jubilado/a, retirado/a o jubilado/a anticipado/a
11.Incapacitado/a permanente
12.Realizando tareas de voluntariado social
13.Dedicado/a a las labores del hogar, al cuidado de niños u otras personas. 10
14.Otra clase de inactividad económica
98.No sabe
98.No contesta

P.40.a. En su empleo principal o en su último empleo ¿cuál es/era la ocupación, profesión u oficio que desempeña/ba?

Precise lo más posible, por ejemplo: dependiente de comercio, mecánico de automóviles, tornero, peón de la construcción, operador de grúa, etc.). Entrevistador: Anote el literal y señale la opción que corresponda según la tabla de clasificación del manual. El informante debe especificar suficientemente para que se pueda codificar correctamente.
Ocupación: ______________________

Filtro: P40=1,2,3,4
P.41. ¿Qué tipo de contrato tiene en la actualidad?
1.Contrato indefinido
2.Contrato temporal.

Para trabajadores: Filtro: P40=1,2,3,4,5,6,7

P.42. Respecto a la situación en la que desarrolla su trabajo ¿cómo es **actualmente**?

1. Trabajo desde casa
2. Trabajo fuera de casa
3. Trabajo desde casa y desde fuera de casa
4. Estoy de permiso para ocuparme de mis hijos
5. Estoy permiso para ocuparme de otros familiares
6. Estoy de vacaciones anuales retribuidas
7. Estoy de permiso no retribuido
8. Otra situación

Para trabajadores Filtro: P40=1,2,3,4,5,6,7

P.43. Asumiendo que su capacidad laboral en su mejor estado de salud tuviera un valor de 10 puntos, ¿cuántos puntos daría a su capacidad laboral actual? 0 (totalmente incapacitado) …10 (muy buena capacidad laboral)

0 1 2 3 4 5 6 7 8 9 10 Ns/Nc 99

Filtro: P40=8

P.44. Me ha dicho que está Vd. en situación de desempleo. ¿En cuál de las siguientes situaciones se encuentra actualmente?

1. Estoy desempleado por un ERTE debido al coronavirus.
2. Estoy desempleado por un despido debido al coronavirus (sin ERTE).
3. He tenido que cerrar mi negocio.
4. He tenido que cesar la actividad que me aportaba ingresos
5. Tenía opciones de comenzar en un trabajo, pero se han pospuesto/cancelado debido al estado de alarma
6. Mi situación de desempleo es previa al estado de alarma y no se ha visto alterada
7. Otras situaciones:

P.39.a ¿Ha cambiado su nivel de estudios desde que realizó la anterior encuesta?

1. Sí → pasar a P.40.b.
2. No → pasar a P.46.
3. No lo recuerdo/No estoy seguro/a → pasar a P.40.b.

P.40.b. ¿Cuáles son sus estudios terminados de más alto nivel?

Anote el literal y señale la opción que corresponda. El informante debe especificar en su respuesta lo suficiente como para que se pueda codificar correctamente, por ejemplo: ha asistido menos de cinco años a la escuela, tres cursos completos de Ciencias Económicas y Empresariales, doctorado en Ciencias Sociales, etc.

Estudios terminados de más alto nivel: ______________________

Agente CATI: no leer. Clasificar en función del literal contestado

1. Analfabetos/as y estudios primarios incompletos.
2. Educación primaria.
3. Primera etapa de la Educación Secundaria y similar.
4. Segunda etapa de la Educación Secundaria y similar
5. Bachillerato y FP de Grado Medio.
6. Educación postsecundaria no superior.
7. Formación Profesional de Grado Superior y títulos propios De universidades de duración igual o superior a 2 años.
8. Diplomados/as universitarios/as, títulos propios universitarios de Experto o especialista y similares (Grados universitarios de 240 créditos ECTS, Bolonia).

9. Licenciados, másteres y especialidades en Ciencias de la Salud por el sistema de residencia y similares. Grados universitarios de más 240 créditos ECTS (Bolonia).
10. Título de Doctorado.
11. No se puede codificar.

Identificación de la persona de referencia del hogar (la que aporta más ingresos)

P.45. Actualmente, la persona que aporta más ingresos al hogar, ¿es la misma que cuando le hicieron la anterior encuesta?

1. SÍ → pasar a P.50.
2. NO → pasar a P.46.
3. No lo recuerdo/No estoy seguro/a → pasar a P.46.

P.46. Actualmente, ¿En su hogar quién es la persona que aporta más ingresos?

1. Yo (ir a P.50)
2. Otra persona (ir a P.47)
3. Otra persona y yo a partes iguales (ir a P.47)

P.47. Y esa persona es...

1. Su cónyuge/pareja
2. Su padre
3. Su madre
4. Otra persona.
5. No Contesta

P.48. ¿Cuáles son los estudios terminados de más alto nivel de la persona que aporta más ingresos en el hogar?

Anote el literal y señale la opción que corresponda. El informante debe especificar en su respuesta lo suficiente como para que se pueda codificar correctamente, por ejemplo: ha asistido menos de cinco años a la escuela, tres cursos completos de Ciencias Económicas y Empresariales, doctorado en Ciencias Sociales, etc.
Estudios terminados de más alto nivel: ________________________
Agente CATI: no leer. Clasificar en función del literal contestado

1. Analfabetos/as y estudios primarios incompletos.
2. Educación primaria.
3. Primera etapa de la Educación Secundaria y similar.
4. Segunda etapa de la Educación Secundaria y similar
5. Bachillerato y FP de Grado Medio.
6. Educación postsecundaria no superior.
7. Formación Profesional de Grado Superior y títulos propios De universidades de duración igual o superior a 2 años.
8. Diplomados/as universitarios/as, títulos propios universitarios de Experto o especialista y similares (Grados universitarios de 240 créditos ECTS, Bolonia).
9. Licenciados, másteres y especialidades en Ciencias de la Salud por el sistema de residencia y similares. Grados universitarios de más 240 créditos ECTS (Bolonia).
10. Título de Doctorado.
11. No se puede codificar.

P.49. ¿En qué situación de las siguientes se encuentra en la actualidad la persona que aporta más ingresos al hogar? (Si se encuentra en varias situaciones, indique solo la que considere principal).

1. Asalariado/a a tiempo completo
2. Asalariado/a a tiempo completo en ERTE

3. Asalariado/a a tiempo parcial
4. Asalariado/a a tiempo parcial por ERTE
5. Empresario/a con asalariados/as
6. Empresario/a sin asalariados/as o trabajador/a independiente o miembro de cooperativa
7. Trabajos familiares no remunerados (ayudas familiares).
8. Parado/a
9. Estudiante
10. Jubilado/a, retirado/a o jubilado/a anticipado/a
11. Incapacitado/a permanente
12. Realizando tareas de voluntariado social
13. Dedicado/a a las labores del hogar, al cuidado de niños u otras personas.
14. Otra clase de inactividad económica
98. No sabe
99. No contesta

P.49.a. En su empleo principal o en su último empleo ¿cuál es/era la ocupación, profesión u oficio que desempeña/ba la persona que aporta más ingresos al hogar?

Precise lo más posible, por ejemplo: dependiente de comercio, mecánico de automóviles, tornero, peón de la construcción, operador de grúa, etc.). Entrevistador: Anote el literal y señale la opción que corresponda según la tabla de clasificación del manual. El informante debe especificar suficientemente para que se pueda codificar correctamente
Ocupación: ___________________

Ingresos y situación económica del hogar

P.50. Un hogar puede tener diferentes fuentes de ingresos y más de un miembro del hogar puede contribuir con sus ingresos. En relación con el total de ingresos de su hogar, ¿cómo suelen llegar a fin de mes?

1. Con mucha facilidad.
2. Con facilidad.
3. Con cierta facilidad.
4. Con cierta dificultad.
5. Con dificultad
6. Con mucha dificultad
7. No sabe.
8. No contesta.

P.51. Desde que comenzó la crisis sanitaria ¿ha tenido en su hogar algún retraso en el pago de recibos como la hipoteca, el alquiler, o suministros como la luz, el agua, impuestos, etc.? (Respuesta múltiple).

1. Hipoteca
2. Alquiler
3. Cuotas de compras aplazadas
4. Cuotas de seguros
5. Impuestos (IBI, los relacionados con vehículos, etc.)
6. Pagos de recibos de agua, gas, calefacción, electricidad, comunidad
7. Tasa de autónomo
8. Pago de colegios
9. Actividades extraescolares
10. Otros

P.51.a. ¿Se ha resuelto? Filtro si en p51=si

1. Sí
2. No

P.52. ¿Ha cambiado su situación económica desde que realizó la primera encuesta (**hace un año**)?
1. Sí, ha mejorado → pasar a P.53.
2. Sí, ha empeorado→ pasar a P.53.
3. No → pasar a P.54.
4. No lo recuerdo/Ne estoy seguro/a → pasar a P.53.

P.53. Dígame en qué intervalo, de los que le leeré a continuación, se encuentran los ingresos mensuales netos del conjunto de miembros de su hogar EN EL MES ANTERIOR

1. Sin ingresos (no leer)
2. Menos de 450 euros
3. De 451 a 900 euros
4. De 901 a 1.600 euros
5. De 1.601 a 2.500 euros
6. De 2.501 a 3.000 euros
7. Más de 3.000 euros
98. NS
98. NC

Observaciones de la entrevista: ____

Esto es todo, muchas gracias por su colaboración y por el tiempo que nos ha dedicado respondiendo esta encuesta. Le recuerdo que tiene a su disposición el número de teléfono **900 10 17 49** por si necesita resolver cualquier duda o información.

Medición 4 - ESSOCgeneral. Cuestionario muestra nueva

Este cuestionario se ha elaborado fundamentalmente a partir del cuestionario de la muestra longitudinal de la 4ª medición de la ESSOC modificando aquellas preguntas que hacían referencia a la encuesta anterior. La estructura del cuestionario se mantiene en cuatro grandes bloques.

- BLOQUE I. CARACTERÍSTICAS DEL HOGAR Y LA VIVIENDA
- BLOQUE II. SALUD, BIENESTAR EMOCIONAL
- BLOQUE III: HÁBITOS Y ESTILOS DE VIDA
- BLOQUE IV. SITUACIÓN ECONÓMICA Y CARACTERÍSTICAS SOCIODEMOGRÁFICAS.

BLOQUE I. CARACTERÍSTICAS DEL HOGAR Y LA VIVIENDA

P.2. ¿Actualmente se encuentra en su vivienda habitual o reside en una vivienda distinta?

1. Habitual → P.3.
2. Segunda residencia → P.2.a
3. Otras: → P.2.a

P.2.a. ¿En qué municipio se encuentra actualmente: Municipio: _________

P.3. La vivienda en la que se encuentra actualmente es una:

1. Vivienda unifamiliar independiente, adosada o pareada
2. Piso o apartamento en un edificio con menos de 10 viviendas
3. Piso o apartamento en un edificio con 10 o más viviendas
4. Otro tipo de vivienda (Ej: Vivienda situada en un edificio destinado principalmente a otros fines barraca, cabaña, chabola, etc.) Especifique.
5. NS/NC

P.4. ¿Cuál es la superficie útil de la vivienda en la que se encuentra actualmente?

1. Menos de 46 m^2 útiles
2. Entre 46 y 60
3. Entre 61 y 75
4. Entre 76 y 90
5. Entre 91 y 120
6. Más de 120
7. No sabe
8. No contesta

P.7. Para conocer las condiciones en las que está actualmente me podría decir ¿qué número de personas residen actualmente incluyéndose usted?
Número de personas: |_|_|

P.7.a. ¿De estas personas cuántas son mayores de 60 años?...... |_|_|
P.7.b. ¿Y cuántas son menores de 16 años?... |_|_|
P.7.c. ¿De las personas menores de 16, cuántas son menores de 6 años? I_I_I

P.8. A efectos de caracterizar su situación de convivencia, ¿me podría decir cuál de las siguientes alternativas lo refleja mejor?

1. Hogar unipersonal
2. Padre o madre solo/a que conviva con algún hijo/a
3. Pareja sin hijos que convivan en el hogar
4. Pareja con hijos/as que convivan en el hogar
5. Otro tipo de hogar
6. NC

P.11. ¿Cuántas habitaciones tiene su vivienda? (no contar la cocina, los baños/lavabos o el comedor/sala de estar).
Número de habitaciones: |_|_|

P.12. Indique el número de personas (<16; 16-59; >=60) que presentan alguna de las condiciones que voy a leerle…

P.12.a. Tener alguna limitación, discapacidad de tipo físico, sensorial o psíquico o mental

1. SI, de que tramo de edad:
   - <16 |_|_|
   - 16-59 |_|_|
   - >=60 |_|_|
2. No

P.12.b. Requiere algún tipo de dedicación o de cuidados especiales

1. SI, de que tramo de edad:
   - <16 |_|_|

- 16-59 |_|_|
- >=60 |_|_|

2. No

Cuidado y uso del tiempo

P.12.C. Por favor, indique quienes de las siguientes personas realizan las siguientes acciones en su hogar actualmente:

1. Siempre yo
2. Habitualmente yo
3. Mi pareja y yo por igual
4. Habitualmente mi pareja
5. Siempre mi pareja
6. Madre de la persona entrevistada
7. Padre de la persona entrevistada
8. Persona remunerada
9. Otras personas. Especificar
10. No sabe/No procede

1. Tareas domésticas (limpieza, cocina, etc...)
2. Tareas de cuidado de hijos/as u otros menores del hogar (general):
   a. Tareas de cuidado físico (alimentación, vestido, higiene, vigilancia)
   b. Tareas de cuidado interactivo (enseñar, leer, jugar, conversar y otras interacciones)
   c. Acompañamiento y transporte de hijos/as a diferentes espacios
3. Tareas de cuidados en adultos con problemas de salud, movilidad, etc. que requieren algún tipo de dedicación o de cuidados especiales
4. Tareas en menores con necesidades especiales de cuidado (problemas de salud, movilidad, etc. que requieren algún tipo de dedicación o de cuidados especiales).

BLOQUE II. SALUD, BIENESTAR EMOCIONAL

Identificación de la infección

P.13. En primer lugar ¿tiene o ha tenido diagnóstico de coronavirus?

1. Sí →pasar a P.13.a
2. No
3. Estoy a la espera del resultado
4. No sabe
5. No contesta

Filtro si 1 en P.13

P.13.a. ¿Cómo de grave ha sido o es su proceso con la enfermedad?

1. Muy leve
2. Leve
3. Grave
4. Muy grave

P.14. ¿Le han hecho alguna vez, alguna de las siguientes pruebas para ver si estaba infectado por el coronavirus? (MULTIRRESPUESTA)

1. Muestra de la garganta o la nariz Sí/No/NC
2. Muestra de sangre en el dedo Sí/No/NC
3. Muestra de sangre en vena Sí/No/NC

P.15. ¿Hay en su entorno alguien que tiene o ha tenido algún caso diagnosticado de infección por coronavirus? Respuesta múltiple

1. Persona/s de mi familia con la que resido en el hogar
2. Persona/s con la que resido en el hogar que no es familiar
3. Familiar/es que no conviven conmigo
4. Amigos, compañeros de trabajo/estudios, vecinos con lo que no resido en el hogar
5. Nadie de mi entorno
6. NC

Hablemos ahora de forma general de cómo se encuentra…

Autopercepción de salud

P.16. En la actualidad, en general diría que su salud es...

1. Excelente
2. Muy buena
3. Buena
4. Regular
5. Mala
6. No sabe/No está seguro.
7. No contesta.

P.17. En la actualidad, en general diría que su salud mental es...

1. Excelente
2. Muy buena
3. Buena
4. Regular
5. Mala
6. No sabe/No está seguro.
7. No contesta.

Bienestar emocional

P.18. A continuación, le voy a leer una lista de sentimientos o comportamientos que quizás haya tenido Vd. **durante la última semana**. Por favor, dígame con qué frecuencia, se ha sentido...

1. En ningún momento o en casi ningún momento
2. En algún momento,
3. Buena parte del tiempo,
4. Todo o casi todo el tiempo
5. No sabe

1. Deprimido/a
2. Feliz
3. Solo/a
4. Ha tenido la sensación de disfrutar de la vida
5. Rebosante de energía
6. Tranquilo/a y relajado/a

P.19. Si toma también como referencia **la última semana**, dígame Ud. su grado de acuerdo o desacuerdo con las siguientes frases.

1. Muy de acuerdo
2. De acuerdo
3. Ni de acuerdo ni en desacuerdo
4. En desacuerdo
5. Muy en desacuerdo
6. No sabe

1. He sido optimista respecto a mi futuro
2. Por lo general me he sentido bien conmigo mismo

Felicidad y apoyo social y afectivo

P.22. En términos generales, ¿en qué medida se considera Ud. una persona feliz o infeliz? Por favor, use una escala de 0 a 10, en la que 0 significa que se considera "completamente infeliz" y 10 que se considera "completamente feliz".

0 1 2 3 4 5 6 7 8 9 10 Ns/Nc 99

P.23. En la siguiente lista se muestran algunas cosas que otras personas hacen por nosotros o nos proporcionan. Elija para cada una la respuesta que mejor refleje su situación:

1. Tanto como deseo
2. Casi como deseo
3. Ni mucho ni poco
4. Menos de lo que deseo
5. Mucho menos de lo que deseo

P.23.1. Recibo visitas/llamadas de mis amigos y familiares
P.23.2. Recibo ayuda en asuntos relacionados con mi casa
P.23.3. Recibo elogios y reconocimientos cuando hago bien mi trabajo
P.23.4. Cuento con personas que se preocupan de lo que me sucede
P.23.5. Recibo amor y afecto
P.23.6. Tengo la posibilidad de hablar con alguien de mis problemas en el trabajo y/o en la casa
P.23.7. Tengo la posibilidad de hablar con alguien de mis problemas personales y familiares
P.23.8. Tengo posibilidad de hablar con alguien de mis problemas económicos
P.23.9. Recibo invitaciones para distraerme y salir con otras personas
P.23.10. Recibo consejos útiles cuando me ocurre algún acontecimiento importante en mi vida
P.23.11. Recibo ayuda cuando estoy enfermo/a en la cama

Molestias y enfermedades crónicas

P.24. Durante el **último mes**, ¿hasta qué punto ha notado Vd. alguna de las siguientes molestias? Utilice una escala del 1 al 5 en la que 1 es Nunca he sentido esa molestia y 5 Con mucha frecuencia.

1. Problemas de sueño
2. Problemas de estómago
3. Incremento de la tensión arterial
4. Mareos
5. Dolores de cabeza
6. Dolores musculares o de articulaciones

6a. Otros dolores

7. Sensación de opresión en el pecho
8. Otra molestia

P.25. ¿Le ha dicho un/a profesional de la salud (médico/a, enfermero/a) que usted padece actualmente alguna de las siguientes enfermedades crónicas? Si responde “SI” hacer P.25.a. ¿Esa condición le limita alguna de sus actividades?

ENFERMEDAD CRÓNICA	P.25. ¿Le ha dicho un/a profesional de la salud si tiene esta condición? (si es ‘No’, saltar a la siguiente)			P.25.a ¿Esa condición le limita alguna de sus actividades?	
	Sí	No	No lo sabe	Sí	No
1. Diabetes/azúcar alta en sangre/azúcar en orina	☐	☐	☐	☐	☐
2. Hipertensión/presión arterial alta	☐	☐	☐	☐	☐
3. Colesterol alto	☐	☐	☐	☐	☐
4. Enfermedad crónica del pulmón / enfisema / bronquitis crónica	☐	☐	☐	☐	☐
5. Asma	☐	☐	☐	☐	☐
6. Enfermedades del corazón (Trastornos cardíacos/insufiencia cardíaca /insuficiencia cardíaca congestiva; angina de pecho; Infarto de miocardio/ataque al corazón)	☐	☐	☐	☐	☐
7. Dolor crónico (pecho; espalda, cuello, hombro, cintura (cervical/lumbar; Migrañas/Jaquecas/ Cefaleas crónicas / dolor de cabeza frecuente)	☐	☐	☐	☐	☐
8. Alergias crónicas (como rinitis, inflamación de ojos, dermatitis, alergia a los alimentos, etc), asma alérgica excluida	☐	☐	☐	☐	☐
9. Mala circulación	☐	☐	☐	☐	☐
10. Varices en las piernas	☐	☐	☐	☐	☐
11. Trastornos depresivos	☐	☐	☐	☐	☐
12. Trastornos de ansiedad	☐	☐	☐	☐	☐
13. Otra enfermedad crónica	☐	☐	☐	☐	☐

Medicamentos

P.26. ¿En qué medida se ha visto modificada la medicación que usted tomaba antes del último mes con respecto a ahora?
1. He disminuido mi medicación
2. Sigo igual, no ha afectado en nada
3. He aumentado mi medicación (incluye si antes no consumía, pero ahora sí)
4. No consumo medicación.

Filtro si 1,2 o 3 en la P.26.
P.26.a. Y En general, ha cambiado la manera en que toma sus medicamentos
1. Sí, ahora estoy cumpliendo mejor con la medicación
2. Sí, ahora estoy cumpliendo peor con la medicación
3. Sigo tomando los mismos medicamentos de la misma manera

P.27. (PARA TODOS). Y en las **últimas 2 semanas**, ¿ha utilizado algún tipo de medicamento (gotas, pastillas, inyecciones, supositorios, etc.)?

1. Sí
2. No
9. N/C

Filtro Si 1 en P.27.

P.27.a. Si ha tomado medicamentos, ¿Qué tipo de medicamento?

1. Medicinas para el catarro, gripe, garganta, bronquios
2. Medicinas para el dolor
3. Medicinas para para bajar la fiebre
4. Antibióticos
5. Tranquilizantes, sedantes
6. Pastillas para dormir
7. Medicamentos antidepresivos
8. Medicamentos para la alergia
9. Medicinas para el corazón
10. Medicinas para la tensión arterial
11. Medicamentos para la diabetes
12. Medicamentos para bajar el colesterol
13. Otros medicamentos

BLOQUE III: HÁBITOS Y ESTILOS DE VIDA

P.28. En relación al último mes, diría Vd. que...

	Mas	Igual	Menos	No procede	Ns/Nc
• 28.1 He realizado más, igual o menos ejercicio	1	2	3	8	9
• 28.2 He consumido más, igual o menos bebidas alcohólicas	1	2	3	8	9
• 28.3 He fumado más, igual, o menos	1	2	3	8	9

	Mejor	Igual	Peor	Ns/Nc
ido mejor, igual o peor	1	2	3	9
• Me he alimentado mejor, igual o peor que antes	1	2	3	9

Mi peso ha aumentado, se ha mantenido o ha disminuido

1. Aumentado
2. Mantenido
3. Disminuido
9. Ns/Nc

Alimentación

P.29. ¿Cuántas raciones de verdura u hortalizas consume al día (las guarniciones o acompañamientos contabilizan como ½ ración)?

1. Dos o más al día (al menos una de ellas en ensalada o crudas)
2. Dos o más al día (aunque ninguna de ellas en ensalada o crudas)

3. Diariamente, aunque menos de dos al día
4. No diariamente, pero tres o más por semana
5. Menos de tres veces por semana
6. No consume
9. NS/NC

P.30. ¿Cuántas piezas de fruta (incluyendo zumo natural) consume al día?
1. Tres o más al día
2. Diariamente, aunque menos de tres veces al día
3. No diariamente, pero tres o más veces por semana
4. Menos de tres veces por semana
5. No consume
9. NS/NC

Actividad física

P.31. Durante las **últimas dos semanas**, ¿Cuántos días a la semana practica normalmente durante más de 30 minutos …? *(incluyendo desplazamientos al trabajo o actividades similares)*

Nº de días/sem

1. Caminar ……………………………………………………………………….. |_|_|
2. Una actividad física ligera como gimnasia suave, juegos que requieren poco esfuerzo y similares ……..…………………………………………………..………. |_|_|
3. Una actividad física moderada como montar en bicicleta, gimnasia, aeróbic, correr, natación ………………………………………………………………….… |_|_|
4. Entrenamiento físico deportivo como fútbol, baloncesto, ciclismo, natación de competición, judo, kárate o similares ……..……........ |_|_|

P.32. ¿Podría decirme cuánto pesa, aproximadamente, sin zapatos ni ropa? (si está embarazada en la actualidad, debe indicar cuánto pesaba antes del inicio del embarazo)

Kilos: |_|_|_|
No sabe 998
No contesta 999

P.33.¿Y cuánto mide, aproximadamente, sin zapatos?

Cms.: |_|_|_|
No sabe 998
No contesta 999

Tabaco

P.34. Cambiando de tema, ¿podría usted decirme si fuma?
1. Sí, fuma diariamente
2. Sí fuma, pero no diariamente
3. No fuma, pero ha fumado
4. No fuma ni ha fumado nunca de manera habitual
9. No contesta

Alcohol

P.35. ¿Ha bebido usted en estas dos últimas semanas alguna bebida alcohólica?
1. Sí, diariamente .... → pasar a P.35.a
2. Sí, pero no diariamente............ → pasar a P.35.a
3. No ....

9. NS/NC

P.35.a. Durante las **últimas 2 semanas** ¿alguna vez ha tomado 6/5 (hombre/mujer, respectivamente) (5/4 PARA MAYORES DE 65 AÑOS) o más bebidas estándar en una misma ocasión? (Por "ocasión" entendemos tomar las bebidas en un intervalo aproximado de cuatro a seis horas)."

1. Sí, alguna vez
2. Sí, varias veces
3. No

8. No sabe
9. No contesta

P.36. Durante las últimas 2 semanas, ¿ha utilizado la bebida para calmar su ansiedad?

1. Totalmente cierto
2. Bastante cierto
3. Bastanto falso
4. Totalmente falso

Sueño

P.37. ¿Podría indicarme, aproximadamente, cuántas horas al día duerme habitualmente?

Horas: |_|_|
NS/NC 99

P.37.a. Las horas que duerme, ¿le permiten descansar lo suficiente?

1. Sí
2. No
8. N/S
9. N/C

Vacunas

P.38. Pasando a otro tema, querríamos saber si se ha vacunado de la gripe en la última campaña 2019

1. Sí
2. No
3. NS/NC

P.38.a. ¿Y en la campaña de 2020?

1. Sí
2. No, pero tengo intención de vacunarme
3. No, y no tengo intención de vacunarme
4. NS/NC

P.38.b. ¿Y se ha vacunado de COVID-19?

1. Sí
2. No, pero tengo intención de vacunarme
3. No y no tengo intención de vacunarme
4. NS/NC

BLOQUE IV. EFECTOS ECONÓMICOS Y CARACTERÍSTICAS SOCIODEMOGRÁFICAS Y SOCIOECONÓMICAS.

Situación laboral

Para poder clasificar las respuestas que usted nos ha dado necesitamos que nos conteste las siguientes preguntas.

P.40. ¿En qué situación de las siguientes se encuentra en la actualidad? (Si se encuentra en varias situaciones, indique solo la que considere principal).

1.Asalariado/a a tiempo completo.
2.Asalariado/a a tiempo completo en ERTE
3.Asalariado/a a tiempo parcial
4.Asalariado/a a tiempo parcial por ERTE
5.Empresario/a con asalariados/as
6.Empresario/a sin asalariados/as o trabajador/a independiente o miembro de cooperativa
7.Trabajos familiares no remunerados (ayudas familiares).
8.Parado/a
9.Estudiante
10.Jubilado/a, retirado/a o jubilado/a anticipado/a
11.Incapacitado/a permanente
12.Realizando tareas de voluntariado social
13.Dedicado/a a las labores del hogar, al cuidado de niños u otras personas. 10
14.Otra clase de inactividad económica
98.No sabe
98.No contesta

P.40.a. En su empleo principal o en su último empleo ¿cuál es/era la ocupación, profesión u oficio que desempeña/ba?

Precise lo más posible, por ejemplo: dependiente de comercio, mecánico de automóviles, tornero, peón de la construcción, operador de grúa, etc.). Entrevistador: Anote el literal y señale la opción que corresponda según la tabla de clasificación del manual. El informante debe especificar suficientemente para que se pueda codificar correctamente.
Ocupación: _____________

P.40.b. ¿Cuáles son sus estudios terminados de más alto nivel?

Anote el literal y señale la opción que corresponda. El informante debe especificar en su respuesta lo suficiente como para que se pueda codificar correctamente, por ejemplo: ha asistido menos de cinco años a la escuela, tres cursos completos de Ciencias Económicas y Empresariales, doctorado en Ciencias Sociales, etc.
Estudios terminados de más alto nivel: ______________________
Agente CATI: no leer. Clasificar en función del literal contestado

1. Analfabetos/as y estudios primarios incompletos.
2. Educación primaria.
3. Primera etapa de la Educación Secundaria y similar.
4. Segunda etapa de la Educación Secundaria y similar
5. Bachillerato y FP de Grado Medio.
6. Educación postsecundaria no superior.
7. Formación Profesional de Grado Superior y títulos propios De universidades de duración igual o superior a 2 años.

8. Diplomados/as universitarios/as, títulos propios universitarios de Experto o especialista y similares (Grados universitarios de 240 créditos ECTS, Bolonia).
9. Licenciados, másteres y especialidades en Ciencias de la Salud por el sistema de residencia y similares. Grados universitarios de más 240 créditos ECTS (Bolonia).
10. Título de Doctorado.
11. No se puede codificar.

Filtro: P40=1,2,3,4
P.41. ¿Qué tipo de contrato tiene en la actualidad?
1.Contrato indefinido
2.Contrato temporal.

Para trabajadores: Filtro: P40=1,2,3,4,5,6,7
P.42. Respecto a la situación en la que desarrolla su trabajo ¿cómo es **actualmente**?
1. Trabajo desde casa
2. Trabajo fuera de casa
3. Trabajo desde casa y desde fuera de casa
4. Estoy de permiso para ocuparme de mis hijos
5. Estoy permiso para ocuparme de otros familiares
6. Estoy de vacaciones anuales retribuidas
7. Estoy de permiso no retribuido
8. Otra situación

Para trabajadores Filtro: P40=1,2,3,4,5,6,7
P.43. Asumiendo que su capacidad laboral en su mejor estado de salud tuviera un valor de 10 puntos, ¿cuántos puntos daría a su capacidad laboral actual? 0 (totalmente incapacitado) …10 (muy buena capacidad laboral)

0 1 2 3 4 5 6 7 8 9 10 Ns/Nc 99

Filtro: P40=8
P.44. Me ha dicho que está Vd. en situación de desempleo. ¿En cuál de las siguientes situaciones se encuentra actualmente?
1. Estoy desempleado por un ERTE debido al coronavirus.
2. Estoy desempleado por un despido debido al coronavirus (sin ERTE).
3. He tenido que cerrar mi negocio.
4. He tenido que cesar la actividad que me aportaba ingresos
5. Tenía opciones de comenzar en un trabajo, pero se han pospuesto/cancelado debido al estado de alarma
6. Mi situación de desempleo es previa al estado de alarma y no se ha visto alterada
7. Otras situaciones:

Identificación de la persona de referencia del hogar (la que aporta más ingresos)

P.46. Actualmente, ¿En su hogar quién es la persona que aporta más ingresos?
1. Yo (ir a P.50.)
2. Otra persona (ir a P.47)
3. Otra persona y yo a partes iguales (ir a P.47)

P.47. Y esa persona es…
1. Su cónyuge/pareja
2. Su padre
3. Su madre
4. Otra persona.

5. No Contesta

P.48. ¿Cuáles son los estudios terminados de más alto nivel de la persona que aporta más ingresos en el hogar?

1. Analfabetos/as y estudios primarios incompletos.
2. Educación primaria.
3. Primera etapa de la Educación Secundaria y similar.
4. Segunda etapa de la Educación Secundaria y similar
5. Bachillerato y FP de Grado Medio.
6. Educación postsecundaria no superior.
7. Formación Profesional de Grado Superior y títulos propios De universidades de duración igual o superior a 2 años.
8. Diplomados/as universitarios/as, títulos propios universitarios de Experto o especialista y similares (Grados universitarios de 240 créditos ECTS, Bolonia).
9. Licenciados, másteres y especialidades en Ciencias de la Salud por el sistema de residencia y similares. Grados universitarios de más 240 créditos ECTS (Bolonia).
10. Título de Doctorado.
11. No se puede codificar.

P.49. ¿En qué situación de las siguientes se encuentra en la actualidad la persona que aporta más ingresos al hogar? (Si se encuentra en varias situaciones, indique solo la que considere principal).

1. Asalariado/a a tiempo completo
2. Asalariado/a a tiempo completo en ERTE
3. Asalariado/a a tiempo parcial
4. Asalariado/a a tiempo parcial por ERTE
5. Empresario/a con asalariados/as
6. Empresario/a sin asalariados/as o trabajador/a independiente o miembro de cooperativa
7. Trabajos familiares no remunerados (ayudas familiares).
8. Parado/a
9. Estudiante
10. Jubilado/a, retirado/a o jubilado/a anticipado/a
11. Incapacitado/a permanente
12. Realizando tareas de voluntariado social
13. Dedicado/a a las labores del hogar, al cuidado de niños u otras personas.
14. Otra clase de inactividad económica
98. No sabe
99. No contesta

P.49.a. En su empleo principal o en su último empleo ¿cuál es/era la ocupación, profesión u oficio que desempeña/ba la persona que aporta más ingresos al hogar?

Precise lo más posible, por ejemplo: dependiente de comercio, mecánico de automóviles, tornero, peón de la construcción, operador de grúa, etc.). Entrevistador: Anote el literal y señale la opción que corresponda según la tabla de clasificación del manual. El informante debe especificar suficientemente para que se pueda codificar correctamente
Ocupación: ___________________

Ingresos y situación económica del hogar

P.50. Un hogar puede tener diferentes fuentes de ingresos y más de un miembro del hogar puede contribuir con sus ingresos. En relación con el total de ingresos de su hogar, ¿cómo suelen llegar a fin de mes?

1. Con mucha facilidad.
2. Con facilidad.
3. Con cierta facilidad.
4. Con cierta dificultad.
5. Con dificultad
6. Con mucha dificultad
7. No sabe.
8. No contesta.

P.51. Desde que comenzó la crisis sanitaria, ¿ha tenido en su hogar algún retraso en el pago de recibos como la hipoteca, el alquiler, o suministros como la luz, el agua, impuestos, etc.? (Respuesta múltiple).

1. Hipoteca
2. Alquiler
3. Cuotas de compras aplazadas
4. Cuotas de seguros
5. Impuestos (IBI, los relacionados con vehículos, etc.)
6. Pagos de recibos de agua, gas, calefacción, electricidad, comunidad
7. Tasa de autónomo
8. Pago de colegios
9. Actividades extraescolares
10. Otros

P.51 a ¿Se ha resuelto? Filtro si en p51=si

5. Sí
6. No

P.53. Dígame en qué intervalo, de los que le leeré a continuación, se encuentran los ingresos mensuales netos del conjunto de miembros de su hogar EN EL MES ANTERIOR

1. Sin ingresos (no leer)
2. Menos de 450 euros
3. De 451 a 900 euros
4. De 901 a 1.600 euros
5. De 1.601 a 2.500 euros
6. De 2.501 a 3.000 euros
7. Más de 3.000 euros

98. NS
98. NC

P52a. ¿Cuáles son los estudios terminados de más alto nivel de su padre?
P52b. ¿Y de su madre?

- Primarios o menos
- Secundarios
- Universitarios
- No sabe
- No contesta

Observaciones de la entrevista: ____

Esto es todo, muchas gracias por su colaboración y por el tiempo que nos ha dedicado respondiendo esta encuesta. Le recuerdo que tiene a su disposición el número de teléfono **900 10 17 49** por si necesita resolver cualquier duda o información.

Medición 5 - ESSOCgeneral. Cuestionario muestra longitudinal

Para la 5ª medición se propone utilizar el mismo cuestionario longitudinal de la 4ª medición de la ESSOC. Al ser una propuesta, este cuestionario puede sufrir modificaciones. En gris aparecen las preguntas que tienen un filtro asociado. La mayoría son preguntas ya recogidas en anteriores mediciones de la ESSOC. La estructura del cuestionario se mantiene en cuatro grandes bloques:

- BLOQUE I. CARACTERÍSTICAS DEL HOGAR Y LA VIVIENDA
- BLOQUE II. SALUD, BIENESTAR EMOCIONAL
- BLOQUE III: HÁBITOS Y ESTILOS DE VIDA
- BLOQUE IV. SITUACIÓN ECONÓMICA Y CARACTERÍSTICAS SOCIODEMOGRÁFICAS

BLOQUE I. CARACTERÍSTICAS DEL HOGAR Y LA VIVIENDA

P.1. Desde la realización de la anterior encuesta, el día <*fecha de la entrevista de la M4*>, ¿Ha cambiado su residencia a una vivienda diferente?

1. Sí → pasar a P.2.
2. No → pasar a P.6.
3. No lo recuerdo/No estoy seguro/a → pasar a P.2.

P.2. ¿Actualmente se encuentra en su vivienda habitual o reside en una vivienda distinta?

1. Habitual → P.3.
2. Segunda residencia → P.2.a
3. Otras: → P.2.a

P.2.a. ¿En qué municipio se encuentra actualmente: Municipio: _________

P.3. La vivienda en la que se encuentra actualmente es una:

1. Vivienda unifamiliar independiente, adosada o pareada
2. Piso o apartamento en un edificio con menos de 10 viviendas
3. Piso o apartamento en un edificio con 10 o más viviendas
4. Otro tipo de vivienda (Ej: Vivienda situada en un edificio destinado principalmente a otros fines barraca, cabaña, chabola, etc.) Especifique.
5. NS/NC

P.4. ¿Cuál es la superficie útil de la vivienda en la que se encuentra actualmente?

1. Menos de 46 m^2 útiles
2. Entre 46 y 60
3. Entre 61 y 75
4. Entre 76 y 90
5. Entre 91 y 120
6. Más de 120
7. No sabe
8. No contesta

P.6. A raíz de la última entrevista ¿ha cambiado el grupo de personas con las que convive?
1. Sí → pasar a P.7.
2. No →pasar a P.11.
3. No lo recuerdo/No estoy seguro/a → pasar a P.7.

P.7. Para conocer las condiciones en las que está actualmente me podría decir ¿qué número de personas residen actualmente incluyéndose usted?
Número de personas: |_|_|

P.7.a. ¿De estas personas cuántas son mayores de 60 años?...... |_|_|
P.7.b. ¿Y cuántas son menores de 16 años?... |_|_|
P.7.c. ¿De las personas menores de 16, cuántas son menores de 6 años? I_I_I

P.8. A efectos de caracterizar su situación de convivencia, ¿me podría decir cuál de las siguientes alternativas lo refleja mejor?
1. Hogar unipersonal
2. Padre o madre solo/a que conviva con algún hijo/a
3. Pareja sin hijos que convivan en el hogar
4. Pareja con hijos/as que convivan en el hogar
5. Otro tipo de hogar
6. NC

(incorporar el filtro si P1=1)
P.11. ¿Cuántas habitaciones tiene su vivienda? (no contar la cocina, los baños/lavabos o el comedor/sala de estar).
Número de habitaciones: |_|_|

P6a. ¿Y han cambiado las condiciones de limitación o discapacidad de algún miembro del hogar desde la última entrevista?
1. Sí → pasar a P.12.
2. No →pasar a P.12.C.
3. No lo recuerdo/No estoy seguro/a → pasar a P.12.

(incorporar el filtro si P6a=1o3)
P.12. Indique el número de personas (<16; 16-59; >=60) que presentan alguna de las condiciones que voy a leerle…

P.12.a. Tener alguna limitación, discapacidad de tipo físico, sensorial o psíquico o mental
1. SI, de que tramo de edad:
    - <16 |_|_|
    - 16-59 |_|_|
    - >=60 |_|_|
2. No

P.12.b. Requiere algún tipo de dedicación o de cuidados especiales
1. SI, de que tramo de edad:
    - <16 |_|_|
    - 16-59 |_|_|
    - >=60 |_|_|
2. No

Cuidado y uso del tiempo

P.12.C. Por favor, indique quienes de las siguientes personas realizan las siguientes acciones en su hogar actualmente:

1. *Siempre yo*
2. *Habitualmente yo*
3. *Mi pareja y yo por igual*
4. *Habitualmente mi pareja*
5. *Siempre mi pareja*
6. *Madre de la persona entrevistada*
7. *Padre de la persona entrevistada*
8. *Persona remunerada*
9. *Otras personas. Especificar*
10. *No sabe/No procede*

1. Tareas domésticas (limpieza, cocina, etc...)
2. Tareas de cuidado de hijos/as u otros menores del hogar (general):
   a. Tareas de cuidado físico (alimentación, vestido, higiene, vigilancia)
   b. Tareas de cuidado interactivo (enseñar, leer, jugar, conversar y otras interacciones)
   c. Acompañamiento y transporte de hijos/as a diferentes espacios

3. Tareas de cuidados en adultos con problemas de salud, movilidad, etc. que requieren algún tipo de dedicación o de cuidados especiales
4. Tareas en menores con necesidades especiales de cuidado (problemas de salud, movilidad, etc. que requieren algún tipo de dedicación o de cuidados especiales).

BLOQUE II. SALUD, BIENESTAR EMOCIONAL

Identificación de la infección

P.13. En primer lugar, nos gustaría confirmar si… ¿tiene o ha tenido diagnóstico de coronavirus?
1. Sí →pasar a P.13.a
2. No
3. Estoy a la espera del resultado
4. No sabe
5. No contesta

Filtro si 1 en P.13
P.13.a. ¿Cómo de grave ha sido o es su proceso con la enfermedad?
1. Muy leve
2. Leve
3. Grave
4. Muy grave

P.14. ¿Le han hecho alguna vez, alguna de las siguientes pruebas para ver si estaba infectado por el coronavirus? (MULTIRRESPUESTA)
1. Muestra de la garganta o la nariz Sí/No/NC
2. Muestra de sangre en el dedo Sí/No/NC
3. Muestra de sangre en vena Sí/No/NC

P.15. ¿Hay en su entorno alguien que tiene o ha tenido algún caso diagnosticado de infección por coronavirus? Respuesta múltiple

1. Persona/s de mi familia con la que resido en el hogar
2. Persona/s con la que resido en el hogar que no es familiar
3. Familiar/es que no conviven conmigo
4. Amigos, compañeros de trabajo/estudios, vecinos con lo que no resido en el hogar
5. Nadie de mi entorno
6. NC

Hablemos ahora de forma general de cómo se encuentra…

Autopercepción de salud

P.16. En la actualidad, en general diría que su salud es...

1. Excelente
2. Muy buena
3. Buena
4. Regular
5. Mala
6. No sabe/No está seguro.
7. No contesta.

P.17. En la actualidad, en general diría que su salud mental es...

1. Excelente
2. Muy buena
3. Buena
4. Regular
5. Mala
6. No sabe/No está seguro.
7. No contesta.

Bienestar emocional

P.18. A continuación, le voy a leer una lista de sentimientos o comportamientos que quizás haya tenido Vd. **durante la última semana**. Por favor, dígame con qué frecuencia, se ha sentido...

1. En ningún momento o en casi ningún momento
2. En algún momento,
3. Buena parte del tiempo,
4. Todo o casi todo el tiempo
5. No sabe

1. Deprimido/a
2. Feliz
3. Solo/a
4. Ha tenido la sensación de disfrutar de la vida
5. Rebosante de energía
6. Tranquilo/a y relajado/a

P.19. Si toma también como referencia **la última semana**, dígame Ud. su grado de acuerdo o desacuerdo con las siguientes frases.

1. Muy de acuerdo
2. De acuerdo

3. Ni de acuerdo ni en desacuerdo
4. En desacuerdo
5. Muy en desacuerdo
6. No sabe

1. He sido optimista respecto a mi futuro
2. Por lo general me he sentido bien conmigo mismo

Felicidad y apoyo social y afectivo

P.22. En términos generales, ¿en qué medida se considera Ud. una persona feliz o infeliz? Por favor, use una escala de 0 a 10, en la que 0 significa que se considera "completamente infeliz" y 10 que se considera "completamente feliz".

0 1 2 3 4 5 6 7 8 9 10 Ns/Nc 99

P.23. En la siguiente lista se muestran algunas cosas que otras personas hacen por nosotros o nos proporcionan. Elija para cada una la respuesta que mejor refleje su situación:

6. Tanto como deseo
7. Casi como deseo
8. Ni mucho ni poco
9. Menos de lo que deseo
10. Mucho menos de lo que deseo

P.23.1. Recibo visitas/llamadas de mis amigos y familiares
P.23.2. Recibo ayuda en asuntos relacionados con mi casa
P.23.3. Recibo elogios y reconocimientos cuando hago bien mi trabajo
P.23.4. Cuento con personas que se preocupan de lo que me sucede
P.23.5. Recibo amor y afecto
P.23.6. Tengo la posibilidad de hablar con alguien de mis problemas en el trabajo y/o en la casa
P.23.7. Tengo la posibilidad de hablar con alguien de mis problemas personales y familiares
P.23.8. Tengo posibilidad de hablar con alguien de mis problemas económicos
P.23.9. Recibo invitaciones para distraerme y salir con otras personas
P.23.10. Recibo consejos útiles cuando me ocurre algún acontecimiento importante en mi vida
P.23.11. Recibo ayuda cuando estoy enfermo/a en la cama

Molestias y enfermedades crónicas

P.24. Durante el **último mes**, ¿hasta qué punto ha notado Vd. alguna de las siguientes molestias? Utilice una escala del 1 al 5 en la que 1 es Nunca he sentido esa molestia y 5 Con mucha frecuencia.

1. Problemas de sueño
2. Problemas de estómago
3. Incremento de la tensión arterial
4. Mareos
5. Dolores de cabeza
6. Dolores musculares o de articulaciones

6a. Otros dolores

7. Sensación de opresión en el pecho
8. Otra molestia

P.25. ¿Le ha dicho un/a profesional de la salud (médico/a, enfermero/a) que usted padece actualmente alguna de las siguientes enfermedades crónicas? Si responde "SI" hacer P.25.a. ¿Esa condición le limita alguna de sus actividades?

ENFERMEDAD CRÓNICA	P.25. ¿Le ha dicho un/a profesional de la salud si tiene esta condición? (si es 'No', saltar a la siguiente)			P.25.a ¿Esa condición le limita alguna de sus actividades?	
	Sí	No	No lo sabe	Sí	No
1. Diabetes/azúcar alta en sangre/azúcar en orina	☐	☐	☐	☐	☐
2. Hipertensión/presión arterial alta	☐	☐	☐	☐	☐
3. Colesterol alto	☐	☐	☐	☐	☐
4. Enfermedad crónica del pulmón / enfisema / bronquitis crónica	☐	☐	☐	☐	☐
5. Asma	☐	☐	☐	☐	☐
6. Enfermedades del corazón (Trastornos cardíacos/insufiencia cardíaca /insuficiencia cardíaca congestiva; angina de pecho; Infarto de miocardio/ataque al corazón)	☐	☐	☐	☐	☐
7. Dolor crónico (pecho; espalda, cuello, hombro, cintura (cervical/lumbar; Migrañas/Jaquecas/ Cefaleas crónicas / dolor de cabeza frecuente)	☐	☐	☐	☐	☐
8. Alergias crónicas (como rinitis, inflamación de ojos, dermatitis, alergia a los alimentos, etc), asma alérgica excluida	☐	☐	☐	☐	☐
9. Mala circulación	☐	☐	☐	☐	☐
10. Varices en las piernas	☐	☐	☐	☐	☐
11. Trastornos depresivos	☐	☐	☐	☐	☐
12. Trastornos de ansiedad	☐	☐	☐	☐	☐
13. Otra enfermedad crónica	☐	☐	☐	☐	☐

Medicamentos

P.26. ¿En qué medida se ha visto modificada la medicación que usted tomaba antes del último mes con respecto a ahora?

1. He disminuido mi medicación
2. Sigo igual, no ha afectado en nada
3. He aumentado mi medicación (incluye si antes no consumía, pero ahora sí)
4. No consumo medicación.

Filtro si 1,2 o 3 en la P.26.

P.26.a. Y En general, ha cambiado la manera en que toma sus medicamentos

1. Sí, ahora estoy cumpliendo mejor con la medicación
2. Sí, ahora estoy cumpliendo peor con la medicación
3. Sigo tomando los mismos medicamentos de la misma manera

P.27. (PARA TODOS). Y en las **últimas 2 semanas**, ¿ha utilizado algún tipo de medicamento (gotas, pastillas, inyecciones, supositorios, etc.)?

1. Sí
2. No
9. NC

Filtro Si 1 en P.27.

P.27.a. Si ha tomado medicamentos, ¿Qué tipo de medicamento?

14. Medicinas para el catarro, gripe, garganta, bronquios
15. Medicinas para el dolor
16. Medicinas para para bajar la fiebre
17. Antibióticos
18. Tranquilizantes, sedantes
19. Pastillas para dormir
20. Medicamentos antidepresivos
21. Medicamentos para la alergia
22. Medicinas para el corazón
23. Medicinas para la tensión arterial
24. Medicamentos para la diabetes
25. Medicamentos para bajar el colesterol
26. Otros medicamentos

BLOQUE III: HÁBITOS Y ESTILOS DE VIDA

P.28. En relación al **último mes**, diría Vd. que…

1. He realizado más, igual o menos ejercicio
2. He consumido más, igual o menos bebidas alcohólicas
3. He fumado más, igual, o menos
4. He dormido mejor, igual o peor
5. Me he alimentado mejor, igual o peor que antes
6. Mi peso ha aumentado, se ha mantenido o ha disminuido

<u>Alimentación</u>

P.29. ¿Cuántas raciones de verdura u hortalizas consume al día (las guarniciones o acompañamientos contabilizan como ½ ración)?

1. Dos o más al día (al menos una de ellas en ensalada o crudas)
2. Dos o más al día (aunque ninguna de ellas en ensalada o crudas)
3. Diariamente, aunque menos de dos al día
4. No diariamente, pero tres o más por semana
5. Menos de tres veces por semana
6. No consume
9. NS/NC

P.30. ¿Cuántas piezas de fruta (incluyendo zumo natural) consume al día?

1. Tres o más al día
2. Diariamente, aunque menos de tres veces al día
3. No diariamente, pero tres o más veces por semana
4. Menos de tres veces por semana
5. No consume

9. NS/NC

Actividad física

P.31. Durante las **últimas dos semanas**, ¿Cuántos días a la semana practica normalmente durante más de 30 minutos …? *(incluyendo desplazamientos al trabajo o actividades similares)*

Nº de días/sem

1. Caminar ………………………………………………………………………………….. |_|_|
2. Una actividad física ligera como gimnasia suave, juegos que requieren poco esfuerzo y similares …….…………………………………………………….……….. |_|_|
3. Una actividad física moderada como montar en bicicleta, gimnasia, aeróbic,correr, natación …………………………………………………………………….… |_|_|
4. Entrenamiento físico deportivo como fútbol, baloncesto, ciclismo, natación de competición, judo, kárate o similares ……..……........ |_|_|

P.32. ¿Podría decirme cuánto pesa, aproximadamente, sin zapatos ni ropa? (si está embarazada en la actualidad, debe indicar cuánto pesaba antes del inicio del embarazo)

Kilos: |_|_|_|
No sabe 998
No contesta 999

P.33.¿Y cuánto mide, aproximadamente, sin zapatos?

Cms.: |_|_|_|
No sabe 998
No contesta 999

Tabaco

P.34. Cambiando de tema, ¿podría usted decirme si fuma?

1. Sí, fuma diariamente
2. Sí fuma, pero no diariamente
3. No fuma, pero ha fumado
4. No fuma ni ha fumado nunca de manera habitual
9. No contesta

Alcohol

P.35. ¿Ha bebido usted en estas dos últimas semanas alguna bebida alcohólica?

1. Sí, diariamente → pasar a P.35.a
2. Sí, pero no diariamente. → pasar a P.35.a
3. No
9. NS/NC

P.35.a. Durante las **últimas 2 semanas** ¿alguna vez ha tomado 6/5 (hombre/mujer, respectivamente) (5/4 PARA MAYORES DE 65 AÑOS) o más bebidas estándar en una misma ocasión? (Por “ocasión” entendemos tomar las bebidas en un intervalo aproximado de cuatro a seis horas).”

1. Sí, alguna vez
2. Sí, varias veces
3. No
8. No sabe
9. No contesta

P.36. Durante las últimas 2 semanas, ¿ha utilizado la bebida para calmar su ansiedad?

1. Totalmente cierto
2. Bastante cierto
3. Bastanto falso
4. Totalmente falso
5. NS/NC

Sueño

P.37. ¿Podría indicarme, aproximadamente, cuántas horas al día duerme habitualmente?

Horas:	|_|_|
NS/NC	99

P.37.a. Las horas que duerme, ¿le permiten descansar lo suficiente?

1. Sí
2. No
8. NS
9. NC

Vacunas

P.38. Pasando a otro tema, querríamos saber si se ha vacunado de la gripe en la última campaña 2019

1. Sí
2. No
3. NS/NC

P.38.a. ¿Y en la campaña de 2020?

1. Sí
2. No, pero tengo intención de vacunarme
3. No y no tengo intención de vacunarme
4. NS/NC

P.38.b. ¿Y se ha vacunado de COVID-19?

1. Sí
2. No, pero tengo intención de vacunarme
3. No y no tengo intención de vacunarme
4. NS/NC

BLOQUE IV. EFECTOS ECONÓMICOS Y CARACTERÍSTICAS SOCIODEMOGRÁFICAS Y SOCIOECONÓMICAS.

Situación laboral

P.39. ¿Ha cambiado su situación laboral desde que realizó la última encuesta?

1. Sí → pasar a P.40.
2. No → pasar a P.39.a.
3. No lo recuerdo/No estoy seguro/a → pasar a P.40.

P.40. ¿En qué situación de las siguientes se encuentra en la actualidad? (Si se encuentra en varias situaciones, indique solo la que considere principal).

1.Asalariado/a a tiempo completo.
2.Asalariado/a a tiempo completo en ERTE
3.Asalariado/a a tiempo parcial
4.Asalariado/a a tiempo parcial por ERTE
5.Empresario/a con asalariados/as
6.Empresario/a sin asalariados/as o trabajador/a independiente o miembro de cooperativa
7.Trabajos familiares no remunerados (ayudas familiares).
8.Parado/a
9.Estudiante
10.Jubilado/a, retirado/a o jubilado/a anticipado/a
11.Incapacitado/a permanente
12.Realizando tareas de voluntariado social
13.Dedicado/a a las labores del hogar, al cuidado de niños u otras personas. 10
14.Otra clase de inactividad económica
98.No sabe
98.No contesta

P.40.a. En su empleo principal o en su último empleo ¿cuál es/era la ocupación, profesión u oficio que desempeña/ba?

Precise lo más posible, por ejemplo: dependiente de comercio, mecánico de automóviles, tornero, peón de la construcción, operador de grúa, etc.). Entrevistador: Anote el literal y señale la opción que corresponda según la tabla de clasificación del manual. El informante debe especificar suficientemente para que se pueda codificar correctamente.
Ocupación: ______________________

Filtro: P40=1,2,3,4
P.41. ¿Qué tipo de contrato tiene en la actualidad?

1.Contrato indefinido
2.Contrato temporal.

Para trabajadores: Filtro: P40=1,2,3,4,5,6,7
P.42. Respecto a la situación en la que desarrolla su trabajo ¿cómo es **actualmente**?

1. Trabajo desde casa
2. Trabajo fuera de casa
3. Trabajo desde casa y desde fuera de casa
4. Estoy de permiso para ocuparme de mis hijos
5. Estoy permiso para ocuparme de otros familiares
6. Estoy de vacaciones anuales retribuidas
7. Estoy de permiso no retribuido

8. Otra situación

Para trabajadores Filtro: P40=1,2,3,4,5,6,7
P.43. Asumiendo que su capacidad laboral en su mejor estado de salud tuviera un valor de 10 puntos, ¿cuántos puntos daría a su capacidad laboral actual? 0 (totalmente incapacitado) …10 (muy buena capacidad laboral)

0 1 2 3 4 5 6 7 8 9 10 Ns/Nc 99

Filtro: P40=8
P.44. Me ha dicho que está Vd. en situación de desempleo. ¿En cuál de las siguientes situaciones se encuentra actualmente?

1. Estoy desempleado por un ERTE debido al coronavirus.
2. Estoy desempleado por un despido debido al coronavirus (sin ERTE).
3. He tenido que cerrar mi negocio.
4. He tenido que cesar la actividad que me aportaba ingresos
5. Tenía opciones de comenzar en un trabajo, pero se han pospuesto/cancelado debido al estado de alarma
6. Mi situación de desempleo es previa al estado de alarma y no se ha visto alterada
7. Otras situaciones:

P.39.a ¿Ha cambiado su nivel de estudios desde que realizó la anterior encuesta?

1. Sí → pasar a P.40.b.
2. No → pasar a P.46.
3. No lo recuerdo/No estoy seguro/a → pasar a P.40.b.

P.40.b. ¿Cuáles son sus estudios terminados de más alto nivel?

Anote el literal y señale la opción que corresponda. El informante debe especificar en su respuesta lo suficiente como para que se pueda codificar correctamente, por ejemplo: ha asistido menos de cinco años a la escuela, tres cursos completos de Ciencias Económicas y Empresariales, doctorado en Ciencias Sociales, etc.
Estudios terminados de más alto nivel: ______________________
Agente CATI: no leer. Clasificar en función del literal contestado

1. Analfabetos/as y estudios primarios incompletos.
2. Educación primaria.
3. Primera etapa de la Educación Secundaria y similar.
4. Segunda etapa de la Educación Secundaria y similar
5. Bachillerato y FP de Grado Medio.
6. Educación postsecundaria no superior.
7. Formación Profesional de Grado Superior y títulos propios De universidades de duración igual o superior a 2 años.
8. Diplomados/as universitarios/as, títulos propios universitarios de Experto o especialista y similares (Grados universitarios de 240 créditos ECTS, Bolonia).
9. Licenciados, másteres y especialidades en Ciencias de la Salud por el sistema de residencia y similares. Grados universitarios de más 240 créditos ECTS (Bolonia).
10. Título de Doctorado.
11. No se puede codificar.

Identificación de la persona de referencia del hogar (la que aporta más ingresos)

P.45. Actualmente, la persona que aporta más ingresos al hogar, ¿es la misma que cuando le hicieron la anterior encuesta?

1. SÍ → pasar a P.50.
2. NO → pasar a P.46.
3. No lo recuerdo/No estoy seguro/a → pasar a P.46.

P.46. Actualmente, ¿En su hogar quién es la persona que aporta más ingresos?
1. Yo (ir a P.50)
2. Otra persona (ir a P.47)
3. Otra persona y yo a partes iguales (ir a P.47)

P.47. Y esa persona es...
1. Su cónyuge/pareja
2. Su padre
3. Su madre
4. Otra persona.
5. No Contesta

P.48. ¿Cuáles son los estudios terminados de más alto nivel de la persona que aporta más ingresos en el hogar?

Anote el literal y señale la opción que corresponda. El informante debe especificar en su respuesta lo suficiente como para que se pueda codificar correctamente, por ejemplo: ha asistido menos de cinco años a la escuela, tres cursos completos de Ciencias Económicas y Empresariales, doctorado en Ciencias Sociales, etc.
Estudios terminados de más alto nivel: ________________________
Agente CATI: no leer. Clasificar en función del literal contestado
1. Analfabetos/as y estudios primarios incompletos.
2. Educación primaria.
3. Primera etapa de la Educación Secundaria y similar.
4. Segunda etapa de la Educación Secundaria y similar
5. Bachillerato y FP de Grado Medio.
6. Educación postsecundaria no superior.
7. Formación Profesional de Grado Superior y títulos propios De universidades de duración igual o superior a 2 años.
8. Diplomados/as universitarios/as, títulos propios universitarios de Experto o especialista y similares (Grados universitarios de 240 créditos ECTS, Bolonia).
9. Licenciados, másteres y especialidades en Ciencias de la Salud por el sistema de residencia y similares. Grados universitarios de más 240 créditos ECTS (Bolonia).
10. Título de Doctorado.
11. No se puede codificar.

P.49. ¿En qué situación de las siguientes se encuentra en la actualidad la persona que aporta más ingresos al hogar? (Si se encuentra en varias situaciones, indique solo la que considere principal).
1. Asalariado/a a tiempo completo
2. Asalariado/a a tiempo completo en ERTE
3. Asalariado/a a tiempo parcial
4. Asalariado/a a tiempo parcial por ERTE
5. Empresario/a con asalariados/as
6. Empresario/a sin asalariados/as o trabajador/a independiente o miembro de cooperativa
7. Trabajos familiares no remunerados (ayudas familiares).
8. Parado/a
9. Estudiante
10. Jubilado/a, retirado/a o jubilado/a anticipado/a

11. Incapacitado/a permanente
12. Realizando tareas de voluntariado social
13. Dedicado/a a las labores del hogar, al cuidado de niños u otras personas.
14. Otra clase de inactividad económica
98. No sabe
99. No contesta

P.49.a. En su empleo principal o en su último empleo ¿cuál es/era la ocupación, profesión u oficio que desempeña/ba la persona que aporta más ingresos al hogar?

Precise lo más posible, por ejemplo: dependiente de comercio, mecánico de automóviles, tornero, peón de la construcción, operador de grúa, etc.). Entrevistador: Anote el literal y señale la opción que corresponda según la tabla de clasificación del manual. El informante debe especificar suficientemente para que se pueda codificar correctamente
Ocupación: ___________________

Ingresos y situación económica del hogar

P.50. Un hogar puede tener diferentes fuentes de ingresos y más de un miembro del hogar puede contribuir con sus ingresos. En relación con el total de ingresos de su hogar, ¿cómo suelen llegar a fin de mes?

1. Con mucha facilidad.
2. Con facilidad.
3. Con cierta facilidad.
4. Con cierta dificultad.
5. Con dificultad
6. Con mucha dificultad
7. No sabe.
8. No contesta.

P.51. Desde que comenzó la crisis sanitaria ¿ha tenido en su hogar algún retraso en el pago de recibos como la hipoteca, el alquiler, o suministros como la luz, el agua, impuestos, etc.? (Respuesta múltiple).

1. Hipoteca
2. Alquiler
3. Cuotas de compras aplazadas
4. Cuotas de seguros
5. Impuestos (IBI, los relacionados con vehículos, etc.)
6. Pagos de recibos de agua, gas, calefacción, electricidad, comunidad
7. Tasa de autónomo
8. Pago de colegios
9. Actividades extraescolares
10. Otros

P.51.a. ¿Se ha resuelto? Filtro si en p51=si

1. Sí
2. No

P.52. ¿Ha cambiado su situación económica desde que realizó la primera encuesta (**hace un año**)?

1. Sí, ha mejorado → pasar a P.53.
2. Sí, ha empeorado→ pasar a P.53.
3. No → pasar a P.54.
4. No lo recuerdo/Ne estoy seguro/a → pasar a P.53.

P.53. Dígame en qué intervalo, de los que le leeré a continuación, se encuentran los ingresos mensuales netos del conjunto de miembros de su hogar EN EL MES ANTERIOR

1. Sin ingresos (no leer)
2. Menos de 450 euros
3. De 451 a 900 euros
4. De 901 a 1.600 euros
5. De 1.601 a 2.500 euros
6. De 2.501 a 3.000 euros
7. Más de 3.000 euros

98. NS

98. NC

Observaciones de la entrevista: ____

Esto es todo, muchas gracias por su colaboración y por el tiempo que nos ha dedicado respondiendo esta encuesta. Le recuerdo que tiene a su disposición el número de teléfono **900 10 17 49** por si necesita resolver cualquier duda o información.

Medición 5 - ESSOCgeneral. Cuestionario muestra nueva

Este cuestionario se ha elaborado fundamentalmente a partir del cuestionario de la muestra longitudinal de la 5ª medición de la ESSOC modificando aquellas preguntas que hacían referencia a la encuesta anterior. Este cuestionario puede sufrir modificaciones. La estructura del cuestionario se mantiene en cuatro grandes bloques.

- BLOQUE I. CARACTERÍSTICAS DEL HOGAR Y LA VIVIENDA
- BLOQUE II. SALUD, BIENESTAR EMOCIONAL
- BLOQUE III: HÁBITOS Y ESTILOS DE VIDA
- BLOQUE IV. SITUACIÓN ECONÓMICA Y CARACTERÍSTICAS SOCIODEMOGRÁFICAS.

BLOQUE I. CARACTERÍSTICAS DEL HOGAR Y LA VIVIENDA

P.2. ¿Actualmente se encuentra en su vivienda habitual o reside en una vivienda distinta?

1. Habitual → P.3.
2. Segunda residencia → P.2.a
3. Otras: → P.2.a

P.2.a. ¿En qué municipio se encuentra actualmente: Municipio: _________

P.3. La vivienda en la que se encuentra actualmente es una:

1. Vivienda unifamiliar independiente, adosada o pareada
2. Piso o apartamento en un edificio con menos de 10 viviendas
3. Piso o apartamento en un edificio con 10 o más viviendas
4. Otro tipo de vivienda (Ej: Vivienda situada en un edificio destinado principalmente a otros fines barraca, cabaña, chabola, etc.) Especifique.
5. NS/NC

P.4. ¿Cuál es la superficie útil de la vivienda en la que se encuentra actualmente?

1. Menos de 46 m^2 útiles
2. Entre 46 y 60
3. Entre 61 y 75
4. Entre 76 y 90
5. Entre 91 y 120
6. Más de 120
7. No sabe
8. No contesta

P.7. Para conocer las condiciones en las que está actualmente me podría decir ¿qué número de personas residen actualmente incluyéndose usted?
Número de personas: |_|_|

P.7.a. ¿De estas personas cuántas son mayores de 60 años?...... |_|_|
P.7.b. ¿Y cuántas son menores de 16 años?... |_|_|
P.7.c. ¿De las personas menores de 16, cuántas son menores de 6 años? I_I_I

P.8. A efectos de caracterizar su situación de convivencia, ¿me podría decir cuál de las siguientes alternativas lo refleja mejor?

1. Hogar unipersonal
2. Padre o madre solo/a que conviva con algún hijo/a
3. Pareja sin hijos que convivan en el hogar
4. Pareja con hijos/as que convivan en el hogar
5. Otro tipo de hogar
6. NC

P.11. ¿Cuántas habitaciones tiene su vivienda? (no contar la cocina, los baños/lavabos o el comedor/sala de estar).
Número de habitaciones: |_|_|

P.12. Indique el número de personas (<16; 16-59; >=60) que presentan alguna de las condiciones que voy a leerle…

P.12.a. Tener alguna limitación, discapacidad de tipo físico, sensorial o psíquico o mental

1. SI, de que tramo de edad:
   - <16 |_|_|
   - 16-59 |_|_|
   - >=60 |_|_|
2. No

P.12.b. Requiere algún tipo de dedicación o de cuidados especiales

1. SI, de que tramo de edad:
   - <16 |_|_|
   - 16-59 |_|_|
   - >=60 |_|_|
2. No

Cuidado y uso del tiempo

P.12.C. Por favor, indique quienes de las siguientes personas realizan las siguientes acciones en su hogar actualmente:

1. Siempre yo
2. Habitualmente yo
3. Mi pareja y yo por igual
4. Habitualmente mi pareja
5. Siempre mi pareja
6. Madre de la persona entrevistada
7. Padre de la persona entrevistada
8. Persona remunerada
9. Otras personas. Especificar
10. No sabe/No procede

1. Tareas domésticas (limpieza, cocina, etc…)
2. Tareas de cuidado de hijos/as u otros menores del hogar (general):

a. Tareas de cuidado físico (alimentación, vestido, higiene, vigilancia)
b. Tareas de cuidado interactivo (enseñar, leer, jugar, conversar y otras interacciones)
c. Acompañamiento y transporte de hijos/as a diferentes espacios

3. Tareas de cuidados en adultos con problemas de salud, movilidad, etc. que requieren algún tipo de dedicación o de cuidados especiales
4. Tareas en menores con necesidades especiales de cuidado (problemas de salud, movilidad, etc. que requieren algún tipo de dedicación o de cuidados especiales).

BLOQUE II. SALUD, BIENESTAR EMOCIONAL

<u>Identificación de la infección</u>

P.13. En primer lugar ¿tiene o ha tenido diagnóstico de coronavirus?
1. Sí →pasar a P.13.a
2. No
3. Estoy a la espera del resultado
4. No sabe
5. No contesta

Filtro si 1 en P.13
P.13.a. ¿Cómo de grave ha sido o es su proceso con la enfermedad?
1. Muy leve
2. Leve
3. Grave
4. Muy grave

P.14. ¿Le han hecho alguna vez, alguna de las siguientes pruebas para ver si estaba infectado por el coronavirus? (MULTIRRESPUESTA)

1. Muestra de la garganta o la nariz	Sí/No/NC
2. Muestra de sangre en el dedo	Sí/No/NC
3. Muestra de sangre en vena	Sí/No/NC

P.15. ¿Hay en su entorno alguien que tiene o ha tenido algún caso diagnosticado de infección por coronavirus? Respuesta múltiple

1. Persona/s de mi familia con la que resido en el hogar
2. Persona/s con la que resido en el hogar que no es familiar
3. Familiar/es que no conviven conmigo
4. Amigos, compañeros de trabajo/estudios, vecinos con lo que no resido en el hogar
5. Nadie de mi entorno
6. NC

Hablemos ahora de forma general de cómo se encuentra…

Autopercepción de salud

P.16. En la actualidad, en general diría que su salud es…

1. Excelente
2. Muy buena
3. Buena
4. Regular
5. Mala
6. No sabe/No está seguro.
7. No contesta.

P.17. En la actualidad, en general diría que su salud mental es…

1. Excelente
2. Muy buena
3. Buena
4. Regular
5. Mala
6. No sabe/No está seguro.
7. No contesta.

Bienestar emocional

P.18. A continuación, le voy a leer una lista de sentimientos o comportamientos que quizás haya tenido Vd. **durante la última semana**. Por favor, dígame con qué frecuencia, se ha sentido…

1. En ningún momento o en casi ningún momento
2. En algún momento,
3. Buena parte del tiempo,
4. Todo o casi todo el tiempo
5. No sabe

1. Deprimido/a
2. Feliz
3. Solo/a
4. Ha tenido la sensación de disfrutar de la vida
5. Rebosante de energía
6. Tranquilo/a y relajado/a

P.19. Si toma también como referencia **la última semana**, dígame Ud. su grado de acuerdo o desacuerdo con las siguientes frases.

1. Muy de acuerdo
2. De acuerdo
3. Ni de acuerdo ni en desacuerdo

4. En desacuerdo
5. Muy en desacuerdo
6. No sabe

1. He sido optimista respecto a mi futuro
2. Por lo general me he sentido bien conmigo mismo

Felicidad y apoyo social y afectivo

P.22. En términos generales, ¿en qué medida se considera Ud. una persona feliz o infeliz? Por favor, use una escala de 0 a 10, en la que 0 significa que se considera "completamente infeliz" y 10 que se considera "completamente feliz".

0 1 2 3 4 5 6 7 8 9 10 Ns/Nc 99

P.23. En la siguiente lista se muestran algunas cosas que otras personas hacen por nosotros o nos proporcionan. Elija para cada una la respuesta que mejor refleje su situación:

1. Tanto como deseo
2. Casi como deseo
3. Ni mucho ni poco
4. Menos de lo que deseo
5. Mucho menos de lo que deseo

P.23.1. Recibo visitas/llamadas de mis amigos y familiares
P.23.2. Recibo ayuda en asuntos relacionados con mi casa
P.23.3. Recibo elogios y reconocimientos cuando hago bien mi trabajo
P.23.4. Cuento con personas que se preocupan de lo que me sucede
P.23.5. Recibo amor y afecto
P.23.6. Tengo la posibilidad de hablar con alguien de mis problemas en el trabajo y/o en la casa
P.23.7. Tengo la posibilidad de hablar con alguien de mis problemas personales y familiares
P.23.8. Tengo posibilidad de hablar con alguien de mis problemas económicos
P.23.9. Recibo invitaciones para distraerme y salir con otras personas
P.23.10. Recibo consejos útiles cuando me ocurre algún acontecimiento importante en mi vida
P.23.11. Recibo ayuda cuando estoy enfermo/a en la cama

Molestias y enfermedades crónicas

P.24. Durante el **último mes**, ¿hasta qué punto ha notado Vd. alguna de las siguientes molestias? Utilice una escala del 1 al 5 en la que 1 es Nunca he sentido esa molestia y 5 Con mucha frecuencia.

1. Problemas de sueño
2. Problemas de estómago
3. Incremento de la tensión arterial
4. Mareos
5. Dolores de cabeza
6. Dolores musculares o de articulaciones
6a. Otros dolores
7. Sensación de opresión en el pecho
8. Otra molestia

P.25. ¿Le ha dicho un/a profesional de la salud (médico/a, enfermero/a) que usted padece actualmente alguna de las siguientes enfermedades crónicas? Si responde "SI" hacer P.25.a. ¿Esa condición le limita alguna de sus actividades?

ENFERMEDAD CRÓNICA	P.25. ¿Le ha dicho un/a profesional de la salud si tiene esta condición? (si es 'No', saltar a la siguiente)			P.25.a ¿Esa condición le limita alguna de sus actividades?	
	Sí	No	No lo sabe	Sí	No
1. Diabetes/azúcar alta en sangre/azúcar en orina	☐	☐	☐	☐	☐
2. Hipertensión/presión arterial alta	☐	☐	☐	☐	☐
3. Colesterol alto	☐	☐	☐	☐	☐
4. Enfermedad crónica del pulmón / enfisema / bronquitis crónica	☐	☐	☐	☐	☐
5. Asma	☐	☐	☐	☐	☐
6. Enfermedades del corazón (Trastornos cardíacos/insufiencia cardíaca /insuficiencia cardíaca congestiva; angina de pecho; Infarto de miocardio/ataque al corazón)	☐	☐	☐	☐	☐
7. Dolor crónico (pecho; espalda, cuello, hombro, cintura (cervical/lumbar; Migrañas/Jaquecas/ Cefaleas crónicas / dolor de cabeza frecuente)	☐	☐	☐	☐	☐
8. Alergias crónicas (como rinitis, inflamación de ojos, dermatitis, alergia a los alimentos, etc), asma alérgica excluida	☐	☐	☐	☐	☐
9. Mala circulación	☐	☐	☐	☐	☐
10. Varices en las piernas	☐	☐	☐	☐	☐
11. Trastornos depresivos	☐	☐	☐	☐	☐
12. Trastornos de ansiedad	☐	☐	☐	☐	☐
13. Otra enfermedad crónica	☐	☐	☐	☐	☐

Medicamentos

P.26. ¿En qué medida se ha visto modificada la medicación que usted tomaba antes del último mes con respecto a ahora?

1. He disminuido mi medicación
2. Sigo igual, no ha afectado en nada
3. He aumentado mi medicación (incluye si antes no consumía, pero ahora sí)
4. No consumo medicación.

Filtro si 1,2 o 3 en la P.26.

P.26.a. Y En general, ha cambiado la manera en que toma sus medicamentos

1. Sí, ahora estoy cumpliendo mejor con la medicación
2. Sí, ahora estoy cumpliendo peor con la medicación
3. Sigo tomando los mismos medicamentos de la misma manera

P.27. (PARA TODOS). Y en las **últimas 2 semanas**, ¿ha utilizado algún tipo de medicamento (gotas, pastillas, inyecciones, supositorios, etc.)?

1. Sí

2. No
9. N/C

Filtro Si 1 en P.27.
P.27.a. Si ha tomado medicamentos, ¿Qué tipo de medicamento?
1. Medicinas para el catarro, gripe, garganta, bronquios
2. Medicinas para el dolor
3. Medicinas para para bajar la fiebre
4. Antibióticos
5. Tranquilizantes, sedantes
6. Pastillas para dormir
7. Medicamentos antidepresivos
8. Medicamentos para la alergia
9. Medicinas para el corazón
10. Medicinas para la tensión arterial
11. Medicamentos para la diabetes
12. Medicamentos para bajar el colesterol
13. Otros medicamentos

BLOQUE III: HÁBITOS Y ESTILOS DE VIDA

P.28. En relación al último mes, diría Vd. que...

	Mas	Igual	Menos	No procede	Ns/Nc
• 28.1 He realizado más, igual o menos ejercicio	1	2	3	8	9
• 28.2 He consumido más, igual o menos bebidas alcohólicas	1	2	3	8	9
• 28.3 He fumado más, igual, o menos	1	2	3	8	9

	Mejor	Igual	Peor	Ns/Nc
ido mejor, igual o peor	1	2	3	9
• Me he alimentado mejor, igual o peor que antes	1	2	3	9

Mi peso ha aumentado, se ha mantenido o ha disminuido
1. Aumentado
2. Mantenido
3. Disminuido
9. Ns/Nc

Alimentación

P.29. ¿Cuántas raciones de verdura u hortalizas consume al día (las guarniciones o acompañamientos contabilizan como ½ ración)?
1. Dos o más al día (al menos una de ellas en ensalada o crudas)
2. Dos o más al día (aunque ninguna de ellas en ensalada o crudas)
3. Diariamente, aunque menos de dos al día
4. No diariamente, pero tres o más por semana
5. Menos de tres veces por semana
6. No consume

9. NS/NC

P.30. ¿Cuántas piezas de fruta (incluyendo zumo natural) consume al día?

1. Tres o más al día
2. Diariamente, aunque menos de tres veces al día
3. No diariamente, pero tres o más veces por semana
4. Menos de tres veces por semana
5. No consume
9. NS/NC

Actividad física

P.31. Durante las **últimas dos semanas**, ¿Cuántos días a la semana practica normalmente durante más de 30 minutos …? *(incluyendo desplazamientos al trabajo o actividades similares)*

Nº de días/sem

1. Caminar ……………………………………………………………………………….. |__|__|
2. Una actividad física ligera como gimnasia suave, juegos que requieren poco esfuerzo y similares …….…………………………………………………….………. |__|__|
3. Una actividad física moderada como montar en bicicleta, gimnasia, aeróbic,correr, natación ……………………………………………………………………….… |__|__|
4. Entrenamiento físico deportivo como fútbol, baloncesto, ciclismo, natación de competición, judo, kárate o similares ……..……........ |__|__|

P.32. ¿Podría decirme cuánto pesa, aproximadamente, sin zapatos ni ropa? (si está embarazada en la actualidad, debe indicar cuánto pesaba antes del inicio del embarazo)

Kilos: |_|_|_|
No sabe 998
No contesta 999

P.33. ¿Y cuánto mide, aproximadamente, sin zapatos?

Cms.: |_|_|_|
No sabe 998
No contesta 999

Tabaco

P.34. Cambiando de tema, ¿podría usted decirme si fuma?

1. Sí, fuma diariamente
2. Sí fuma, pero no diariamente
3. No fuma, pero ha fumado
4. No fuma ni ha fumado nunca de manera habitual
9. No contesta

Alcohol

P.35. ¿Ha bebido usted en estas dos últimas semanas alguna bebida alcohólica?

1. Sí, diariamente .... → pasar a P.35.a
2. Sí, pero no diariamente..... → pasar a P.35.a
3. No ....
9. NS/NC

P.35.a. Durante las **últimas 2 semanas** ¿alguna vez ha tomado 6/5 (hombre/mujer, respectivamente) (5/4 PARA MAYORES DE 65 AÑOS) o más bebidas estándar en una misma

ocasión? (Por "ocasión" entendemos tomar las bebidas en un intervalo aproximado de cuatro a seis horas)."

1. Sí, alguna vez
2. Sí, varias veces
3. No
8. No sabe
9. No contesta

P.36. Durante las últimas 2 semanas, ¿ha utilizado la bebida para calmar su ansiedad?

1. Totalmente cierto
2. Bastante cierto
3. Bastanto falso
4. Totalmente falso

Sueño

P.37. ¿Podría indicarme, aproximadamente, cuántas horas al día duerme habitualmente?

Horas: |_|_|
NS/NC 99

P.37.a. Las horas que duerme, ¿le permiten descansar lo suficiente?

1. Sí
2. No
8. N/S
9. N/C

Vacunas

P.38. Pasando a otro tema, querríamos saber si se ha vacunado de la gripe en la última campaña 2019

1. Sí
2. No
3. NS/NC

P.38.a. ¿Y en la campaña de 2020?

1. Sí
2. No, pero tengo intención de vacunarme
3. No, y no tengo intención de vacunarme
4. NS/NC

P.38.b. ¿Y se ha vacunado de COVID-19?

1. Sí
2. No, pero tengo intención de vacunarme
3. No y no tengo intención de vacunarme
4. NS/NC

BLOQUE IV. EFECTOS ECONÓMICOS Y CARACTERÍSTICAS SOCIODEMOGRÁFICAS Y SOCIOECONÓMICAS.

Situación laboral

Para poder clasificar las respuestas que usted nos ha dado necesitamos que nos conteste las siguientes preguntas.

P.40. ¿En qué situación de las siguientes se encuentra en la actualidad? (Si se encuentra en varias situaciones, indique solo la que considere principal).

1.Asalariado/a a tiempo completo.
2.Asalariado/a a tiempo completo en ERTE
3.Asalariado/a a tiempo parcial
4.Asalariado/a a tiempo parcial por ERTE
5.Empresario/a con asalariados/as
6.Empresario/a sin asalariados/as o trabajador/a independiente o miembro de cooperativa
7.Trabajos familiares no remunerados (ayudas familiares).
8.Parado/a
9.Estudiante
10.Jubilado/a, retirado/a o jubilado/a anticipado/a
11.Incapacitado/a permanente
12.Realizando tareas de voluntariado social
13.Dedicado/a a las labores del hogar, al cuidado de niños u otras personas. 10
14.Otra clase de inactividad económica
98.No sabe
98.No contesta

P.40.a. En su empleo principal o en su último empleo ¿cuál es/era la ocupación, profesión u oficio que desempeña/ba?

Precise lo más posible, por ejemplo: dependiente de comercio, mecánico de automóviles, tornero, peón de la construcción, operador de grúa, etc.). Entrevistador: Anote el literal y señale la opción que corresponda según la tabla de clasificación del manual. El informante debe especificar suficientemente para que se pueda codificar correctamente.
Ocupación: _____________

P.40.b. ¿Cuáles son sus estudios terminados de más alto nivel?

Anote el literal y señale la opción que corresponda. El informante debe especificar en su respuesta lo suficiente como para que se pueda codificar correctamente, por ejemplo: ha asistido menos de cinco años a la escuela, tres cursos completos de Ciencias Económicas y Empresariales, doctorado en Ciencias Sociales, etc.
Estudios terminados de más alto nivel: ______________________
Agente CATI: no leer. Clasificar en función del literal contestado

1. Analfabetos/as y estudios primarios incompletos.
2. Educación primaria.
3. Primera etapa de la Educación Secundaria y similar.
4. Segunda etapa de la Educación Secundaria y similar
5. Bachillerato y FP de Grado Medio.
6. Educación postsecundaria no superior.
7. Formación Profesional de Grado Superior y títulos propios De universidades de duración igual o superior a 2 años.

8. Diplomados/as universitarios/as, títulos propios universitarios de Experto o especialista y similares (Grados universitarios de 240 créditos ECTS, Bolonia).
9. Licenciados, másteres y especialidades en Ciencias de la Salud por el sistema de residencia y similares. Grados universitarios de más 240 créditos ECTS (Bolonia).
10. Título de Doctorado.
11. No se puede codificar.

Filtro: P40=1,2,3,4
P.41. ¿Qué tipo de contrato tiene en la actualidad?
1.Contrato indefinido
2.Contrato temporal.

Para trabajadores: Filtro: P40=1,2,3,4,5,6,7
P.42. Respecto a la situación en la que desarrolla su trabajo ¿cómo es **actualmente**?
1. Trabajo desde casa
2. Trabajo fuera de casa
3. Trabajo desde casa y desde fuera de casa
4. Estoy de permiso para ocuparme de mis hijos
5. Estoy permiso para ocuparme de otros familiares
6. Estoy de vacaciones anuales retribuidas
7. Estoy de permiso no retribuido
8. Otra situación

Para trabajadores Filtro: P40=1,2,3,4,5,6,7
P.43. Asumiendo que su capacidad laboral en su mejor estado de salud tuviera un valor de 10 puntos, ¿cuántos puntos daría a su capacidad laboral actual? 0 (totalmente incapacitado) …10 (muy buena capacidad laboral)

0 1 2 3 4 5 6 7 8 9 10 Ns/Nc 99

Filtro: P40=8
P.44. Me ha dicho que está Vd. en situación de desempleo. ¿En cuál de las siguientes situaciones se encuentra actualmente?
1. Estoy desempleado por un ERTE debido al coronavirus.
2. Estoy desempleado por un despido debido al coronavirus (sin ERTE).
3. He tenido que cerrar mi negocio.
4. He tenido que cesar la actividad que me aportaba ingresos
5. Tenía opciones de comenzar en un trabajo, pero se han pospuesto/cancelado debido al estado de alarma
6. Mi situación de desempleo es previa al estado de alarma y no se ha visto alterada
7. Otras situaciones:

Identificación de la persona de referencia del hogar (la que aporta más ingresos)

P.46. Actualmente, ¿En su hogar quién es la persona que aporta más ingresos?
1. Yo (ir a P.50.)
2. Otra persona (ir a P.47)
3. Otra persona y yo a partes iguales (ir a P.47)

P.47. Y esa persona es...
1. Su cónyuge/pareja
2. Su padre
3. Su madre
4. Otra persona.
5. No Contesta

P.48. ¿Cuáles son los estudios terminados de más alto nivel de la persona que aporta más ingresos en el hogar?

1. Analfabetos/as y estudios primarios incompletos.
2. Educación primaria.
3. Primera etapa de la Educación Secundaria y similar.
4. Segunda etapa de la Educación Secundaria y similar
5. Bachillerato y FP de Grado Medio.
6. Educación postsecundaria no superior.
7. Formación Profesional de Grado Superior y títulos propios De universidades de duración igual o superior a 2 años.
8. Diplomados/as universitarios/as, títulos propios universitarios de Experto o especialista y similares (Grados universitarios de 240 créditos ECTS, Bolonia).
9. Licenciados, másteres y especialidades en Ciencias de la Salud por el sistema de residencia y similares. Grados universitarios de más 240 créditos ECTS (Bolonia).
10. Título de Doctorado.
11. No se puede codificar.

P.49. ¿En qué situación de las siguientes se encuentra en la actualidad la persona que aporta más ingresos al hogar? (Si se encuentra en varias situaciones, indique solo la que considere principal).

1. Asalariado/a a tiempo completo
2. Asalariado/a a tiempo completo en ERTE
3. Asalariado/a a tiempo parcial
4. Asalariado/a a tiempo parcial por ERTE
5. Empresario/a con asalariados/as
6. Empresario/a sin asalariados/as o trabajador/a independiente o miembro de cooperativa
7. Trabajos familiares no remunerados (ayudas familiares).
8. Parado/a
9. Estudiante
10. Jubilado/a, retirado/a o jubilado/a anticipado/a
11. Incapacitado/a permanente
12. Realizando tareas de voluntariado social
13. Dedicado/a a las labores del hogar, al cuidado de niños u otras personas.
14. Otra clase de inactividad económica
98. No sabe
99. No contesta

P.49.a. En su empleo principal o en su último empleo ¿cuál es/era la ocupación, profesión u oficio que desempeña/ba la persona que aporta más ingresos al hogar?

Precise lo más posible, por ejemplo: dependiente de comercio, mecánico de automóviles, tornero, peón de la construcción, operador de grúa, etc.). Entrevistador: Anote el literal y señale la opción que corresponda según la tabla de clasificación del manual. El informante debe especificar suficientemente para que se pueda codificar correctamente
Ocupación: ___________________

Ingresos y situación económica del hogar

P.50. Un hogar puede tener diferentes fuentes de ingresos y más de un miembro del hogar puede contribuir con sus ingresos. En relación con el total de ingresos de su hogar, ¿cómo suelen llegar a fin de mes?

1. Con mucha facilidad.
2. Con facilidad.
3. Con cierta facilidad.
4. Con cierta dificultad.
5. Con dificultad
6. Con mucha dificultad
7. No sabe.
8. No contesta.

P.51. Desde que comenzó la crisis sanitaria, ¿ha tenido en su hogar algún retraso en el pago de recibos como la hipoteca, el alquiler, o suministros como la luz, el agua, impuestos, etc.? (Respuesta múltiple).

1. Hipoteca
2. Alquiler
3. Cuotas de compras aplazadas
4. Cuotas de seguros
5. Impuestos (IBI, los relacionados con vehículos, etc.)
6. Pagos de recibos de agua, gas, calefacción, electricidad, comunidad
7. Tasa de autónomo
8. Pago de colegios
9. Actividades extraescolares
10. Otros

P.51 a ¿Se ha resuelto? Filtro si en p51=si

1. Sí
2. No

P.53. Dígame en qué intervalo, de los que le leeré a continuación, se encuentran los ingresos mensuales netos del conjunto de miembros de su hogar EN EL MES ANTERIOR

1. Sin ingresos (no leer)
2. Menos de 450 euros
3. De 451 a 900 euros
4. De 901 a 1.600 euros
5. De 1.601 a 2.500 euros
6. De 2.501 a 3.000 euros
7. Más de 3.000 euros

98. NS
98. NC

P52a. ¿Cuáles son los estudios terminados de más alto nivel de su padre?
P52b. ¿Y de su madre?

- Primarios o menos
- Secundarios
- Universitarios
- No sabe
- No contesta

Observaciones de la entrevista: ____

Esto es todo, muchas gracias por su colaboración y por el tiempo que nos ha dedicado respondiendo esta encuesta. Le recuerdo que tiene a su disposición el número de teléfono **900 10 17 49** por si necesita resolver cualquier duda o información.

4. Cuestionarios de ESSOCzonas

La muestra de zonas desfavorecidas consta de dos mediciones. Los cuestionarios están basados en el cuestionario de ESSOCgeneral de la 4ª medición con el único añadido sobre la etnia.

Medición 1 - ESSOCzonas. Cuestionario

- BLOQUE I. CARACTERÍSTICAS DEL HOGAR Y LA VIVIENDA
- BLOQUE II. SALUD, BIENESTAR EMOCIONAL
- BLOQUE III: HÁBITOS Y ESTILOS DE VIDA
- BLOQUE IV. SITUACIÓN ECONÓMICA Y CARACTERÍSTICAS SOCIODEMOGRÁFICAS.

BLOQUE I. CARACTERÍSTICAS DEL HOGAR Y LA VIVIENDA

P.2. ¿Actualmente se encuentra en su vivienda habitual o reside en una vivienda distinta?

1. Habitual → P.3.
2. Segunda residencia → P.2.a
3. Otras: → P.2.a

P.2.a. ¿En qué municipio se encuentra actualmente: Municipio: _________

P.3. La vivienda en la que se encuentra actualmente es una:

1. Vivienda unifamiliar independiente, adosada o pareada
2. Piso o apartamento en un edificio con menos de 10 viviendas
3. Piso o apartamento en un edificio con 10 o más viviendas
4. Otro tipo de vivienda (Ej: Vivienda situada en un edificio destinado principalmente a otros fines barraca, cabaña, chabola, etc.) Especifique.
5. NS/NC

P.4. ¿Cuál es la superficie útil de la vivienda en la que se encuentra actualmente?

1. Menos de 46 m^2 útiles
2. Entre 46 y 60
3. Entre 61 y 75
4. Entre 76 y 90
5. Entre 91 y 120
6. Más de 120
7. No sabe
8. No contesta

P.7. Para conocer las condiciones en las que está actualmente me podría decir ¿qué número de personas residen actualmente incluyéndose usted?
Número de personas: |_|_|

P.7.a. ¿De estas personas cuántas son mayores de 60 años?...... |_|_|
P.7.b. ¿Y cuántas son menores de 16 años?... |_|_|
P.7.c. ¿De las personas menores de 16, cuántas son menores de 6 años? I_I_I

P.8. A efectos de caracterizar su situación de convivencia, ¿me podría decir cuál de las siguientes alternativas lo refleja mejor?

1. Hogar unipersonal
2. Padre o madre solo/a que conviva con algún hijo/a
3. Pareja sin hijos que convivan en el hogar
4. Pareja con hijos/as que convivan en el hogar
5. Otro tipo de hogar
6. NC

P.11. ¿Cuántas habitaciones tiene su vivienda? (no contar la cocina, los baños/lavabos o el comedor/sala de estar).
Número de habitaciones: |_|_|

P.12. Indique el número de personas (<16; 16-59; >=60) que presentan alguna de las condiciones que voy a leerle…

P.12.a. Tener alguna limitación, discapacidad de tipo físico, sensorial o psíquico o mental

3. SI, de que tramo de edad:
    - <16 |_|_|
    - 16-59 |_|_|
    - >=60 |_|_|
4. No

P.12.b. Requiere algún tipo de dedicación o de cuidados especiales

3. SI, de que tramo de edad:
    - <16 |_|_|
    - 16-59 |_|_|
    - >=60 |_|_|
4. No

Cuidado y uso del tiempo

P.12.C. Por favor, indique quienes de las siguientes personas realizan las siguientes acciones en su hogar actualmente:

11. Siempre yo
12. Habitualmente yo
13. Mi pareja y yo por igual
14. Habitualmente mi pareja
15. Siempre mi pareja
16. Madre de la persona entrevistada
17. Padre de la persona entrevistada
18. Persona remunerada
19. Otras personas. Especificar
20. No sabe/No procede

1. Tareas domésticas (limpieza, cocina, etc...)

2. Tareas de cuidado de hijos/as u otros menores del hogar (general):
    d. Tareas de cuidado físico (alimentación, vestido, higiene, vigilancia)
    e. Tareas de cuidado interactivo (enseñar, leer, jugar, conversar y otras interacciones)
    f. Acompañamiento y transporte de hijos/as a diferentes espacios
3. Tareas de cuidados en adultos con problemas de salud, movilidad, etc. que requieren algún tipo de dedicación o de cuidados especiales
4. Tareas en menores con necesidades especiales de cuidado (problemas de salud, movilidad, etc. que requieren algún tipo de dedicación o de cuidados especiales).

BLOQUE II. SALUD, BIENESTAR EMOCIONAL

Identificación de la infección

P.13. En primer lugar ¿tiene o ha tenido diagnóstico de coronavirus?

1. Sí →pasar a P.13.a
2. No
3. Estoy a la espera del resultado
4. No sabe
5. No contesta

Filtro si 1 en P.13

P.13.a. ¿Cómo de grave ha sido o es su proceso con la enfermedad?

1. Muy leve
2. Leve
3. Grave
4. Muy grave

P.14. ¿Le han hecho alguna vez, alguna de las siguientes pruebas para ver si estaba infectado por el coronavirus? (MULTIRRESPUESTA)

1. Muestra de la garganta o la nariz	Sí/No/NC
2. Muestra de sangre en el dedo	Sí/No/NC
3. Muestra de sangre en vena	Sí/No/NC

P.15. ¿Hay en su entorno alguien que tiene o ha tenido algún caso diagnosticado de infección por coronavirus? Respuesta múltiple

1. Persona/s de mi familia con la que resido en el hogar
2. Persona/s con la que resido en el hogar que no es familiar
3. Familiar/es que no conviven conmigo
4. Amigos, compañeros de trabajo/estudios, vecinos con lo que no resido en el hogar
5. Nadie de mi entorno
6. NC

Hablemos ahora de forma general de cómo se encuentra…

Autopercepción de salud

P.16. En la actualidad, en general diría que su salud es...

8. Excelente
9. Muy buena
10. Buena
11. Regular

12. Mala
13. No sabe/No está seguro.
14. No contesta.

P.17. En la actualidad, en general diría que su salud mental es…

8. Excelente
9. Muy buena
10. Buena
11. Regular
12. Mala
13. No sabe/No está seguro.
14. No contesta.

Bienestar emocional

P.18. A continuación, le voy a leer una lista de sentimientos o comportamientos que quizás haya tenido Vd. **durante la última semana**. Por favor, dígame con qué frecuencia, se ha sentido…

1. En ningún momento o en casi ningún momento
2. En algún momento,
3. Buena parte del tiempo,
4. Todo o casi todo el tiempo
5. No sabe

1. Deprimido/a
2. Feliz
3. Solo/a
4. Ha tenido la sensación de disfrutar de la vida
5. Rebosante de energía
6. Tranquilo/a y relajado/a

P.19. Si toma también como referencia **la última semana**, dígame Ud. su grado de acuerdo o desacuerdo con las siguientes frases.

1. Muy de acuerdo
2. De acuerdo
3. Ni de acuerdo ni en desacuerdo
4. En desacuerdo
5. Muy en desacuerdo
6. No sabe

1. He sido optimista respecto a mi futuro
2. Por lo general me he sentido bien conmigo mismo

Felicidad y apoyo social y afectivo

P.22. En términos generales, ¿en qué medida se considera Ud. una persona feliz o infeliz? Por favor, use una escala de 0 a 10, en la que 0 significa que se considera "completamente infeliz" y 10 que se considera "completamente feliz".

0 1 2 3 4 5 6 7 8 9 10 Ns/Nc 99

P.23. En la siguiente lista se muestran algunas cosas que otras personas hacen por nosotros o nos proporcionan. Elija para cada una la respuesta que mejor refleje su situación:

6. Tanto como deseo
7. Casi como deseo
8. Ni mucho ni poco
9. Menos de lo que deseo
10. Mucho menos de lo que deseo

P.23.1. Recibo visitas/llamadas de mis amigos y familiares
P.23.2. Recibo ayuda en asuntos relacionados con mi casa
P.23.3. Recibo elogios y reconocimientos cuando hago bien mi trabajo
P.23.4. Cuento con personas que se preocupan de lo que me sucede
P.23.5. Recibo amor y afecto
P.23.6. Tengo la posibilidad de hablar con alguien de mis problemas en el trabajo y/o en la casa
P.23.7. Tengo la posibilidad de hablar con alguien de mis problemas personales y familiares
P.23.8. Tengo posibilidad de hablar con alguien de mis problemas económicos
P.23.9. Recibo invitaciones para distraerme y salir con otras personas
P.23.10. Recibo consejos útiles cuando me ocurre algún acontecimiento importante en mi vida
P.23.11. Recibo ayuda cuando estoy enfermo/a en la cama

<u>Molestias y enfermedades crónicas</u>

P.24. Durante el **último mes**, ¿hasta qué punto ha notado Vd. alguna de las siguientes molestias? Utilice una escala del 1 al 5 en la que 1 es Nunca he sentido esa molestia y 5 Con mucha frecuencia.

1. Problemas de sueño
2. Problemas de estómago
3. Incremento de la tensión arterial
4. Mareos
5. Dolores de cabeza
6. Dolores musculares o de articulaciones

6a. Otros dolores

7. Sensación de opresión en el pecho
8. Otra molestia

P.25. ¿Le ha dicho un/a profesional de la salud (médico/a, enfermero/a) que usted padece actualmente alguna de las siguientes enfermedades crónicas? Si responde "SI" hacer P.25.a. ¿Esa condición le limita alguna de sus actividades?

ENFERMEDAD CRÓNICA	P.25.			P.25.a	
	¿Le ha dicho un/a profesional de la salud si tiene esta condición? (si es 'No', saltar a la siguiente)			¿Esa condición le limita alguna de sus actividades?	
	Sí	No	No lo sabe	Sí	No
14. Diabetes/azúcar alta en sangre/azúcar en orina	☐	☐	☐	☐	☐
15. Hipertensión/presión arterial alta	☐	☐	☐	☐	☐
16. Colesterol alto	☐	☐	☐	☐	☐
17. Enfermedad crónica del pulmón / enfisema / bronquitis crónica	☐	☐	☐	☐	☐
18. Asma	☐	☐	☐	☐	☐

19. Enfermedades del corazón (Trastornos cardíacos/insufiencia cardíaca /insuficiencia cardíaca congestiva; angina de pecho; Infarto de miocardio/ataque al corazón)	☐	☐	☐	☐	☐
20. Dolor crónico (pecho; espalda, cuello, hombro, cintura (cervical/lumbar; Migrañas/Jaquecas/ Cefaleas crónicas / dolor de cabeza frecuente)	☐	☐	☐	☐	☐
21. Alergias crónicas (como rinitis, inflamación de ojos, dermatitis, alergia a los alimentos, etc), asma alérgica excluida	☐	☐	☐	☐	☐
22. Mala circulación	☐	☐	☐	☐	☐
23. Varices en las piernas	☐	☐	☐	☐	☐
24. Trastornos depresivos	☐	☐	☐	☐	☐
25. Trastornos de ansiedad	☐	☐	☐	☐	☐
26. Otra enfermedad crónica	☐	☐	☐	☐	☐

Medicamentos

P.26. ¿En qué medida se ha visto modificada la medicación que usted tomaba antes del último mes con respecto a ahora?

1. He disminuido mi medicación
2. Sigo igual, no ha afectado en nada
3. He aumentado mi medicación (incluye si antes no consumía, pero ahora sí)
4. No consumo medicación.

Filtro si 1,2 o 3 en la P.26.

P.26.a. Y En general, ha cambiado la manera en que toma sus medicamentos

1. Sí, ahora estoy cumpliendo mejor con la medicación
2. Sí, ahora estoy cumpliendo peor con la medicación
3. Sigo tomando los mismos medicamentos de la misma manera

P.27. (PARA TODOS). Y en las **últimas 2 semanas**, ¿ha utilizado algún tipo de medicamento (gotas, pastillas, inyecciones, supositorios, etc.)?

1. Sí
2. No
9. N/C

Filtro Si 1 en P.27.

P.27.a. Si ha tomado medicamentos, ¿Qué tipo de medicamento?

14. Medicinas para el catarro, gripe, garganta, bronquios
15. Medicinas para el dolor
16. Medicinas para para bajar la fiebre
17. Antibióticos
18. Tranquilizantes, sedantes
19. Pastillas para dormir
20. Medicamentos antidepresivos
21. Medicamentos para la alergia
22. Medicinas para el corazón
23. Medicinas para la tensión arterial
24. Medicamentos para la diabetes
25. Medicamentos para bajar el colesterol

26. Otros medicamentos

BLOQUE III: HÁBITOS Y ESTILOS DE VIDA

P.28. En relación al último mes, diría Vd. que…

	Mas	Igual	Menos	No procede	Ns/Nc
• 28.1 He realizado más, igual o menos ejercicio	1	2	3	8	9
• 28.2 He consumido más, igual o menos bebidas alcohólicas	1	2	3	8	9
• 28.3 He fumado más, igual, o menos	1	2	3	8	9

	Mejor	Igual	Peor	Ns/Nc
ido mejor, igual o peor	1	2	3	9
• Me he alimentado mejor, igual o peor que antes	1	2	3	9

Mi peso ha aumentado, se ha mantenido o ha disminuido

1. Aumentado
2. Mantenido
3. Disminuido
9. Ns/Nc

Alimentación

P.29. ¿Cuántas raciones de verdura u hortalizas consume al día (las guarniciones o acompañamientos contabilizan como ½ ración)?

1. Dos o más al día (al menos una de ellas en ensalada o crudas)
2. Dos o más al día (aunque ninguna de ellas en ensalada o crudas)
3. Diariamente, aunque menos de dos al día
4. No diariamente, pero tres o más por semana
5. Menos de tres veces por semana
6. No consume
9. NS/NC

P.30. ¿Cuántas piezas de fruta (incluyendo zumo natural) consume al día?

1. Tres o más al día
2. Diariamente, aunque menos de tres veces al día
3. No diariamente, pero tres o más veces por semana
4. Menos de tres veces por semana
5. No consume
9. NS/NC

Actividad física

P.31. Durante las **últimas dos semanas**, ¿Cuántos días a la semana practica normalmente durante más de 30 minutos …? *(incluyendo desplazamientos al trabajo o actividades similares)*

Nº de días/sem

5. Caminar ……………………………………………………………………………….. |__|__|
6. Una actividad física ligera como gimnasia suave, juegos que requieren poco esfuerzo y similares …….………………………………………………….………. |__|__|
7. Una actividad física moderada como montar en bicicleta, gimnasia, aeróbic, correr, natación …………………………………………………………………….… |__|__|
8. Entrenamiento físico deportivo como fútbol, baloncesto, ciclismo, natación de competición, judo, kárate o similares ……......................................……........ |__|__|

P.32. ¿Podría decirme cuánto pesa, aproximadamente, sin zapatos ni ropa? (si está embarazada en la actualidad, debe indicar cuánto pesaba antes del inicio del embarazo)

Kilos: |_|_|_|

No sabe 998

No contesta 999

P.33.¿Y cuánto mide, aproximadamente, sin zapatos?

Cms.: |_|_|_|

No sabe 998

No contesta 999

Tabaco

P.34. Cambiando de tema, ¿podría usted decirme si fuma?

5. Sí, fuma diariamente
6. Sí fuma, pero no diariamente
7. No fuma, pero ha fumado
8. No fuma ni ha fumado nunca de manera habitual
9. No contesta

Alcohol

P.35. ¿Ha bebido usted en estas dos últimas semanas alguna bebida alcohólica?

4. Sí, diariamente .... → pasar a P.35.a
5. Sí, pero no diariamente............ → pasar a P.35.a
6. No ....
9. NS/NC

P.35.a. Durante las **últimas 2 semanas** ¿alguna vez ha tomado 6/5 (hombre/mujer, respectivamente) (5/4 PARA MAYORES DE 65 AÑOS) o más bebidas estándar en una misma ocasión? (Por "ocasión" entendemos tomar las bebidas en un intervalo aproximado de cuatro a seis horas)."

4. Sí, alguna vez
5. Sí, varias veces
6. No
8. No sabe
9. No contesta

P.36. Durante las últimas 2 semanas, ¿ha utilizado la bebida para calmar su ansiedad?

1. Totalmente cierto
2. Bastante cierto
3. Bastanto falso
4. Totalmente falso

Sueño

P.37. ¿Podría indicarme, aproximadamente, cuántas horas al día duerme habitualmente?

Horas: |_|_|
NS/NC 99

P.37.a. Las horas que duerme, ¿le permiten descansar lo suficiente?

3. Sí
4. No
8. N/S
9. N/C

Vacunas

P.38. Pasando a otro tema, querríamos saber si se ha vacunado de la gripe en la última campaña 2019

4. Sí
5. No
6. NS/NC

P.38.a. ¿Y en la campaña de 2020?

5. Sí
6. No, pero tengo intención de vacunarme
7. No, y no tengo intención de vacunarme
8. NS/NC

P.38.b. ¿Y se ha vacunado de COVID-19?

5. Sí
6. No, pero tengo intención de vacunarme
7. No y no tengo intención de vacunarme
8. NS/NC

BLOQUE IV. EFECTOS ECONÓMICOS Y CARACTERÍSTICAS SOCIODEMOGRÁFICAS Y SOCIOECONÓMICAS.

Situación laboral

Para poder clasificar las respuestas que usted nos ha dado necesitamos que nos conteste las siguientes preguntas.

P.40. ¿En qué situación de las siguientes se encuentra en la actualidad? (Si se encuentra en varias situaciones, indique solo la que considere principal).

1.Asalariado/a a tiempo completo.
2.Asalariado/a a tiempo completo en ERTE
3.Asalariado/a a tiempo parcial
4.Asalariado/a a tiempo parcial por ERTE
5.Empresario/a con asalariados/as
6.Empresario/a sin asalariados/as o trabajador/a independiente o miembro de cooperativa
7.Trabajos familiares no remunerados (ayudas familiares).
8.Parado/a
9.Estudiante
10.Jubilado/a, retirado/a o jubilado/a anticipado/a
11.Incapacitado/a permanente
12.Realizando tareas de voluntariado social

13.Dedicado/a a las labores del hogar, al cuidado de niños u otras personas. 10
14.Otra clase de inactividad económica
98.No sabe
98.No contesta

P.40.a. En su empleo principal o en su último empleo ¿cuál es/era la ocupación, profesión u oficio que desempeña/ba?

Precise lo más posible, por ejemplo: dependiente de comercio, mecánico de automóviles, tornero, peón de la construcción, operador de grúa, etc.). Entrevistador: Anote el literal y señale la opción que corresponda según la tabla de clasificación del manual. El informante debe especificar suficientemente para que se pueda codificar correctamente.
Ocupación: _____________

P.40.b. ¿Cuáles son sus estudios terminados de más alto nivel?

Anote el literal y señale la opción que corresponda. El informante debe especificar en su respuesta lo suficiente como para que se pueda codificar correctamente, por ejemplo: ha asistido menos de cinco años a la escuela, tres cursos completos de Ciencias Económicas y Empresariales, doctorado en Ciencias Sociales, etc.
Estudios terminados de más alto nivel: _______________________
Agente CATI: no leer. Clasificar en función del literal contestado

1. Analfabetos/as y estudios primarios incompletos.
2. Educación primaria.
3. Primera etapa de la Educación Secundaria y similar.
4. Segunda etapa de la Educación Secundaria y similar
5. Bachillerato y FP de Grado Medio.
6. Educación postsecundaria no superior.
7. Formación Profesional de Grado Superior y títulos propios De universidades de duración igual o superior a 2 años.
8. Diplomados/as universitarios/as, títulos propios universitarios de Experto o especialista y similares (Grados universitarios de 240 créditos ECTS, Bolonia).
9. Licenciados, másteres y especialidades en Ciencias de la Salud por el sistema de residencia y similares. Grados universitarios de más 240 créditos ECTS (Bolonia).
10. Título de Doctorado.
11. No se puede codificar.

Filtro: P40=1,2,3,4
P.41. ¿Qué tipo de contrato tiene en la actualidad?
1.Contrato indefinido
2.Contrato temporal.

Para trabajadores: Filtro: P40=1,2,3,4,5,6,7
P.42. Respecto a la situación en la que desarrolla su trabajo ¿cómo es **actualmente**?
1. Trabajo desde casa
2. Trabajo fuera de casa
3. Trabajo desde casa y desde fuera de casa
4. Estoy de permiso para ocuparme de mis hijos
5. Estoy permiso para ocuparme de otros familiares
6. Estoy de vacaciones anuales retribuidas
7. Estoy de permiso no retribuido
8. Otra situación

Para trabajadores Filtro: P40=1,2,3,4,5,6,7

P.43. Asumiendo que su capacidad laboral en su mejor estado de salud tuviera un valor de 10 puntos, ¿cuántos puntos daría a su capacidad laboral actual? 0 (totalmente incapacitado) …10 (muy buena capacidad laboral)

0 1 2 3 4 5 6 7 8 9 10   Ns/Nc   99

Filtro: P40=8
P.44. Me ha dicho que está Vd. en situación de desempleo. ¿En cuál de las siguientes situaciones se encuentra actualmente?

1. Estoy desempleado por un ERTE debido al coronavirus.
2. Estoy desempleado por un despido debido al coronavirus (sin ERTE).
3. He tenido que cerrar mi negocio.
4. He tenido que cesar la actividad que me aportaba ingresos
5. Tenía opciones de comenzar en un trabajo, pero se han pospuesto/cancelado debido al estado de alarma
6. Mi situación de desempleo es previa al estado de alarma y no se ha visto alterada
7. Otras situaciones:

Identificación de la persona de referencia del hogar (la que aporta más ingresos)

P.46. Actualmente, ¿En su hogar quién es la persona que aporta más ingresos?

1. Yo (ir a P.50.)
2. Otra persona (ir a P.47)
3. Otra persona y yo a partes iguales (ir a P.47)

P.47. Y esa persona es...

1. Su cónyuge/pareja
2. Su padre
3. Su madre
4. Otra persona.
5. No Contesta

P.48. ¿Cuáles son los estudios terminados de más alto nivel de la persona que aporta más ingresos en el hogar?

1. Analfabetos/as y estudios primarios incompletos.
2. Educación primaria.
3. Primera etapa de la Educación Secundaria y similar.
4. Segunda etapa de la Educación Secundaria y similar
5. Bachillerato y FP de Grado Medio.
6. Educación postsecundaria no superior.
7. Formación Profesional de Grado Superior y títulos propios De universidades de duración igual o superior a 2 años.
8. Diplomados/as universitarios/as, títulos propios universitarios de Experto o especialista y similares (Grados universitarios de 240 créditos ECTS, Bolonia).
9. Licenciados, másteres y especialidades en Ciencias de la Salud por el sistema de residencia y similares. Grados universitarios de más 240 créditos ECTS (Bolonia).
10. Título de Doctorado.
11. No se puede codificar.

P.49. ¿En qué situación de las siguientes se encuentra en la actualidad la persona que aporta más ingresos al hogar? (Si se encuentra en varias situaciones, indique solo la que considere principal).

1. Asalariado/a a tiempo completo
2. Asalariado/a a tiempo completo en ERTE

3. Asalariado/a a tiempo parcial
4. Asalariado/a a tiempo parcial por ERTE
5. Empresario/a con asalariados/as
6. Empresario/a sin asalariados/as o trabajador/a independiente o miembro de cooperativa
7. Trabajos familiares no remunerados (ayudas familiares).
8. Parado/a
9. Estudiante
10. Jubilado/a, retirado/a o jubilado/a anticipado/a
11. Incapacitado/a permanente
12. Realizando tareas de voluntariado social
13. Dedicado/a a las labores del hogar, al cuidado de niños u otras personas.
14. Otra clase de inactividad económica
98. No sabe
99. No contesta

P.49.a. En su empleo principal o en su último empleo ¿cuál es/era la ocupación, profesión u oficio que desempeña/ba la persona que aporta más ingresos al hogar?

Precise lo más posible, por ejemplo: dependiente de comercio, mecánico de automóviles, tornero, peón de la construcción, operador de grúa, etc.). Entrevistador: Anote el literal y señale la opción que corresponda según la tabla de clasificación del manual. El informante debe especificar suficientemente para que se pueda codificar correctamente
Ocupación: ___________________

Ingresos y situación económica del hogar

P.50. Un hogar puede tener diferentes fuentes de ingresos y más de un miembro del hogar puede contribuir con sus ingresos. En relación con el total de ingresos de su hogar, ¿cómo suelen llegar a fin de mes?

1. Con mucha facilidad.
2. Con facilidad.
3. Con cierta facilidad.
4. Con cierta dificultad.
5. Con dificultad
6. Con mucha dificultad
7. No sabe.
8. No contesta.

P.51. Desde que comenzó la crisis sanitaria, ¿ha tenido en su hogar algún retraso en el pago de recibos como la hipoteca, el alquiler, o suministros como la luz, el agua, impuestos, etc.? (Respuesta múltiple).

1. Hipoteca
2. Alquiler
3. Cuotas de compras aplazadas
4. Cuotas de seguros
5. Impuestos (IBI, los relacionados con vehículos, etc.)
6. Pagos de recibos de agua, gas, calefacción, electricidad, comunidad
7. Tasa de autónomo
8. Pago de colegios
9. Actividades extraescolares
10. Otros

P.51 a ¿Se ha resuelto? Filtro si en p51=si

7. Sí
8. No

P.53. Dígame en qué intervalo, de los que le leeré a continuación, se encuentran los ingresos mensuales netos del conjunto de miembros de su hogar EN EL MES ANTERIOR

1. Sin ingresos (no leer)
2. Menos de 450 euros
3. De 451 a 900 euros
4. De 901 a 1.600 euros
5. De 1.601 a 2.500 euros
6. De 2.501 a 3.000 euros
7. Más de 3.000 euros
98. NS
98. NC

P52a. ¿Cuáles son los estudios terminados de más alto nivel de su padre?
P52b. ¿Y de su madre?

- Primarios o menos
- Secundarios
- Universitarios
- No sabe
- No contesta

Observaciones de la entrevista: ____

Esto es todo, muchas gracias por su colaboración y por el tiempo que nos ha dedicado respondiendo esta encuesta. Le recuerdo que tiene a su disposición el número de teléfono **900 10 17 49** por si necesita resolver cualquier duda o información.

Medición 2 - ESSOCzonas. Cuestionario muestra longitudinal

- BLOQUE I. CARACTERÍSTICAS DEL HOGAR Y LA VIVIENDA
- BLOQUE II. SALUD, BIENESTAR EMOCIONAL
- BLOQUE III: HÁBITOS Y ESTILOS DE VIDA
- BLOQUE IV. SITUACIÓN ECONÓMICA Y CARACTERÍSTICAS SOCIODEMOGRÁFICAS

BLOQUE I. CARACTERÍSTICAS DEL HOGAR Y LA VIVIENDA

P.1. Desde la realización de la anterior encuesta, el día <*fecha de la entrevista de la M3*>, ¿Ha cambiado su residencia a una vivienda diferente?

1. Sí → pasar a P.2.
2. No → pasar a P.6.
3. No lo recuerdo/No estoy seguro/a → pasar a P.2.

P.2. ¿Actualmente se encuentra en su vivienda habitual o reside en una vivienda distinta?

1. Habitual → P.3.
2. Segunda residencia → P.2.a
3. Otras: → P.2.a

P.2.a. ¿En qué municipio se encuentra actualmente: Municipio: _________

P.3. La vivienda en la que se encuentra actualmente es una:

1. Vivienda unifamiliar independiente, adosada o pareada
2. Piso o apartamento en un edificio con menos de 10 viviendas
3. Piso o apartamento en un edificio con 10 o más viviendas
4. Otro tipo de vivienda (Ej: Vivienda situada en un edificio destinado principalmente a otros fines barraca, cabaña, chabola, etc.) Especifique.
5. NS/NC

P.4. ¿Cuál es la superficie útil de la vivienda en la que se encuentra actualmente?

1. Menos de 46 m² útiles
2. Entre 46 y 60
3. Entre 61 y 75
4. Entre 76 y 90
5. Entre 91 y 120
6. Más de 120
7. No sabe
8. No contesta

P.6. A raíz de la última entrevista ¿ha cambiado el grupo de personas con las que convive?

1. Sí → pasar a P.7.
2. No →pasar a P.11.
3. No lo recuerdo/No estoy seguro/a → pasar a P.7.

P.7. Para conocer las condiciones en las que está actualmente me podría decir ¿qué número de personas residen actualmente incluyéndose usted?
Número de personas: |_|_|

P.7.a. ¿De estas personas cuántas son mayores de 60 años?…… |_|_|
P.7.b. ¿Y cuántas son menores de 16 años?… |_|_|
P.7.c. ¿De las personas menores de 16, cuántas son menores de 6 años? I_I_I

P.8. A efectos de caracterizar su situación de convivencia, ¿me podría decir cuál de las siguientes alternativas lo refleja mejor?

1. Hogar unipersonal
2. Padre o madre solo/a que conviva con algún hijo/a
3. Pareja sin hijos que convivan en el hogar
4. Pareja con hijos/as que convivan en el hogar
5. Otro tipo de hogar
6. NC

(incorporar el filtro si P1=1)
P.11. ¿Cuántas habitaciones tiene su vivienda? (no contar la cocina, los baños/lavabos o el comedor/sala de estar).
Número de habitaciones: |_|_|

P6a. ¿Y han cambiado las condiciones de limitación o discapacidad de algún miembro del hogar desde la última entrevista?

1. Sí → pasar a P.12.
2. No →pasar a P.12.C.
3. No lo recuerdo/No estoy seguro/a → pasar a P.12.

(incorporar el filtro si P6a=1o3)

P.12. Indique el número de personas (<16; 16-59; >=60) que presentan alguna de las condiciones que voy a leerle…

P.12.a. Tener alguna limitación, discapacidad de tipo físico, sensorial o psíquico o mental

3. SI, de que tramo de edad:
    - <16 |_|_|
    - 16-59 |_|_|
    - >=60 |_|_|
4. No

P.12.b. Requiere algún tipo de dedicación o de cuidados especiales

3. SI, de que tramo de edad:
    - <16 |_|_|
    - 16-59 |_|_|
    - >=60 |_|_|
4. No

<u>Cuidado y uso del tiempo</u>

P.12.C. Por favor, indique quienes de las siguientes personas realizan las siguientes acciones en su hogar actualmente:

11. *Siempre yo*
12. *Habitualmente yo*
13. *Mi pareja y yo por igual*
14. *Habitualmente mi pareja*
15. *Siempre mi pareja*
16. *Madre de la persona entrevistada*
17. *Padre de la persona entrevistada*
18. *Persona remunerada*
19. *Otras personas. Especificar*
20. *No sabe/No procede*

5. Tareas domésticas (limpieza, cocina, etc…)

6. Tareas de cuidado de hijos/as u otros menores del hogar (general):

- **d.** Tareas de cuidado físico (alimentación, vestido, higiene, vigilancia)
- **e.** Tareas de cuidado interactivo (enseñar, leer, jugar, conversar y otras interacciones)
- **f.** Acompañamiento y transporte de hijos/as a diferentes espacios

7. Tareas de cuidados en adultos con problemas de salud, movilidad, etc. que requieren algún tipo de dedicación o de cuidados especiales

8. Tareas en menores con necesidades especiales de cuidado (problemas de salud, movilidad, etc. que requieren algún tipo de dedicación o de cuidados especiales).

BLOQUE II. SALUD, BIENESTAR EMOCIONAL

Identificación de la infección

P.13. En primer lugar, nos gustaría confirmar si… ¿tiene o ha tenido diagnóstico de coronavirus?

1. Sí →pasar a P.13.a
2. No
3. Estoy a la espera del resultado
4. No sabe
5. No contesta

Filtro si 1 en P.13

P.13.a. ¿Cómo de grave ha sido o es su proceso con la enfermedad?

1. Muy leve
2. Leve
3. Grave
4. Muy grave

P.14. ¿Le han hecho alguna vez, alguna de las siguientes pruebas para ver si estaba infectado por el coronavirus? (MULTIRRESPUESTA)

1. Muestra de la garganta o la nariz	Sí/No/NC
2. Muestra de sangre en el dedo	Sí/No/NC
3. Muestra de sangre en vena	Sí/No/NC

P.15. ¿Hay en su entorno alguien que tiene o ha tenido algún caso diagnosticado de infección por coronavirus? Respuesta múltiple

1. Persona/s de mi familia con la que resido en el hogar
2. Persona/s con la que resido en el hogar que no es familiar
3. Familiar/es que no conviven conmigo
4. Amigos, compañeros de trabajo/estudios, vecinos con lo que no resido en el hogar
5. Nadie de mi entorno
6. NC

Hablemos ahora de forma general de cómo se encuentra…

Autopercepción de salud

P.16. En la actualidad, en general diría que su salud es...

8. Excelente
9. Muy buena
10. Buena
11. Regular
12. Mala
13. No sabe/No está seguro.
14. No contesta.

P.17. En la actualidad, en general diría que su salud mental es...

1. Excelente
2. Muy buena
3. Buena
4. Regular
5. Mala
6. No sabe/No está seguro.

7. No contesta.

Bienestar emocional

P.18. A continuación, le voy a leer una lista de sentimientos o comportamientos que quizás haya tenido Vd. **durante la última semana**. Por favor, dígame con qué frecuencia, se ha sentido…

1. En ningún momento o en casi ningún momento
2. En algún momento,
3. Buena parte del tiempo,
4. Todo o casi todo el tiempo
5. No sabe

1. Deprimido/a
2. Feliz
3. Solo/a
4. Ha tenido la sensación de disfrutar de la vida
5. Rebosante de energía
6. Tranquilo/a y relajado/a

P.19. Si toma también como referencia **la última semana**, dígame Ud. su grado de acuerdo o desacuerdo con las siguientes frases.

1. Muy de acuerdo
2. De acuerdo
3. Ni de acuerdo ni en desacuerdo
4. En desacuerdo
5. Muy en desacuerdo
6. No sabe

1. He sido optimista respecto a mi futuro
2. Por lo general me he sentido bien conmigo mismo

Felicidad y apoyo social y afectivo

P.22. En términos generales, ¿en qué medida se considera Ud. una persona feliz o infeliz? Por favor, use una escala de 0 a 10, en la que 0 significa que se considera "completamente infeliz" y 10 que se considera "completamente feliz".

0 1 2 3 4 5 6 7 8 9 10 Ns/Nc 99

P.23. En la siguiente lista se muestran algunas cosas que otras personas hacen por nosotros o nos proporcionan. Elija para cada una la respuesta que mejor refleje su situación:

11. Tanto como deseo
12. Casi como deseo
13. Ni mucho ni poco
14. Menos de lo que deseo
15. Mucho menos de lo que deseo

P.23.1. Recibo visitas/llamadas de mis amigos y familiares
P.23.2. Recibo ayuda en asuntos relacionados con mi casa
P.23.3. Recibo elogios y reconocimientos cuando hago bien mi trabajo
P.23.4. Cuento con personas que se preocupan de lo que me sucede
P.23.5. Recibo amor y afecto

P.23.6. Tengo la posibilidad de hablar con alguien de mis problemas en el trabajo y/o en la casa
P.23.7. Tengo la posibilidad de hablar con alguien de mis problemas personales y familiares
P.23.8. Tengo posibilidad de hablar con alguien de mis problemas económicos
P.23.9. Recibo invitaciones para distraerme y salir con otras personas
P.23.10. Recibo consejos útiles cuando me ocurre algún acontecimiento importante en mi vida
P.23.11. Recibo ayuda cuando estoy enfermo/a en la cama

Molestias y enfermedades crónicas

P.24. Durante el **último mes**, ¿hasta qué punto ha notado Vd. alguna de las siguientes molestias? Utilice una escala del 1 al 5 en la que 1 es Nunca he sentido esa molestia y 5 Con mucha frecuencia.

1. Problemas de sueño
2. Problemas de estómago
3. Incremento de la tensión arterial
4. Mareos
5. Dolores de cabeza
6. Dolores musculares o de articulaciones

6a. Otros dolores

7. Sensación de opresión en el pecho
8. Otra molestia

P.25. ¿Le ha dicho un/a profesional de la salud (médico/a, enfermero/a) que usted padece actualmente alguna de las siguientes enfermedades crónicas? Si responde "SI" hacer P.25.a. ¿Esa condición le limita alguna de sus actividades?

ENFERMEDAD CRÓNICA	P.25. ¿Le ha dicho un/a profesional de la salud si tiene esta condición? (si es 'No', saltar a la siguiente)			P.25.a ¿Esa condición le limita alguna de sus actividades?	
	Sí	No	No lo sabe	Sí	No
14. Diabetes/azúcar alta en sangre/azúcar en orina	☐	☐	☐	☐	☐
15. Hipertensión/presión arterial alta	☐	☐	☐	☐	☐
16. Colesterol alto	☐	☐	☐	☐	☐
17. Enfermedad crónica del pulmón / enfisema / bronquitis crónica	☐	☐	☐	☐	☐
18. Asma	☐	☐	☐	☐	☐
19. Enfermedades del corazón (Trastornos cardíacos/insufiencia cardíaca /insuficiencia cardíaca congestiva; angina de pecho; Infarto de miocardio/ataque al corazón)	☐	☐	☐	☐	☐
20. Dolor crónico (pecho; espalda, cuello, hombro, cintura (cervical/lumbar; Migrañas/Jaquecas/ Cefaleas crónicas / dolor de cabeza frecuente)	☐	☐	☐	☐	☐
21. Alergias crónicas (como rinitis, inflamación de ojos, dermatitis, alergia a los alimentos, etc), asma alérgica excluida	☐	☐	☐	☐	☐
22. Mala circulación	☐	☐	☐	☐	☐

23. Varices en las piernas	☐	☐	☐	☐	☐
24. Trastornos depresivos	☐	☐	☐	☐	☐
25. Trastornos de ansiedad	☐	☐	☐	☐	☐
26. Otra enfermedad crónica	☐	☐	☐	☐	☐

Medicamentos

P.26. ¿En qué medida se ha visto modificada la medicación que usted tomaba antes del último mes con respecto a ahora?

1. He disminuido mi medicación
2. Sigo igual, no ha afectado en nada
3. He aumentado mi medicación (incluye si antes no consumía, pero ahora sí)
4. No consumo medicación.

Filtro si 1,2 o 3 en la P.26.

P.26.a. Y En general, ha cambiado la manera en que toma sus medicamentos

1. Sí, ahora estoy cumpliendo mejor con la medicación
2. Sí, ahora estoy cumpliendo peor con la medicación
3. Sigo tomando los mismos medicamentos de la misma manera

P.27. (PARA TODOS). Y en las **últimas 2 semanas**, ¿ha utilizado algún tipo de medicamento (gotas, pastillas, inyecciones, supositorios, etc.)?

1. Sí
2. No
9. NC

Filtro Si 1 en P.27.

P.27.a. Si ha tomado medicamentos, ¿Qué tipo de medicamento?

27. Medicinas para el catarro, gripe, garganta, bronquios
28. Medicinas para el dolor
29. Medicinas para para bajar la fiebre
30. Antibióticos
31. Tranquilizantes, sedantes
32. Pastillas para dormir
33. Medicamentos antidepresivos
34. Medicamentos para la alergia
35. Medicinas para el corazón
36. Medicinas para la tensión arterial
37. Medicamentos para la diabetes
38. Medicamentos para bajar el colesterol
39. Otros medicamentos

BLOQUE III: HÁBITOS Y ESTILOS DE VIDA

P.28. En relación al **último mes**, diría Vd. que…

1. He realizado más, igual o menos ejercicio
2. He consumido más, igual o menos bebidas alcohólicas
3. He fumado más, igual, o menos
4. He dormido mejor, igual o peor
5. Me he alimentado mejor, igual o peor que antes
6. Mi peso ha aumentado, se ha mantenido o ha disminuido

Alimentación

P.29. ¿Cuántas raciones de verdura u hortalizas consume al día (las guarniciones o acompañamientos contabilizan como ½ ración)?

1. Dos o más al día (al menos una de ellas en ensalada o crudas)
2. Dos o más al día (aunque ninguna de ellas en ensalada o crudas)
3. Diariamente, aunque menos de dos al día
4. No diariamente, pero tres o más por semana
5. Menos de tres veces por semana
6. No consume
9. NS/NC

P.30. ¿Cuántas piezas de fruta (incluyendo zumo natural) consume al día?

1. Tres o más al día
2. Diariamente, aunque menos de tres veces al día
3. No diariamente, pero tres o más veces por semana
4. Menos de tres veces por semana
5. No consume
9. NS/NC

Actividad física

P.31. Durante las **últimas dos semanas**, ¿Cuántos días a la semana practica normalmente durante más de 30 minutos …? *(incluyendo desplazamientos al trabajo o actividades similares)*

Nº de días/sem

5. Caminar ………………………………………………………………………….. |_|_|
6. Una actividad física ligera como gimnasia suave, juegos que requieren poco esfuerzo y similares …….…………………………………………………….………. |_|_|
7. Una actividad física moderada como montar en bicicleta, gimnasia, aeróbic, correr, natación …………………………………………………………………….… |_|_|
8. Entrenamiento físico deportivo como fútbol, baloncesto, ciclismo, natación de competición, judo, kárate o similares ……......................................……........ |_|_|

P.32. ¿Podría decirme cuánto pesa, aproximadamente, sin zapatos ni ropa? (si está embarazada en la actualidad, debe indicar cuánto pesaba antes del inicio del embarazo)

Kilos: |_|_|_|
No sabe 998
No contesta 999

P.33.¿Y cuánto mide, aproximadamente, sin zapatos?

Cms.: |_|_|_|
No sabe 998
No contesta 999

Tabaco

P.34. Cambiando de tema, ¿podría usted decirme si fuma?

5. Sí, fuma diariamente
6. Sí fuma, pero no diariamente
7. No fuma, pero ha fumado

8. No fuma ni ha fumado nunca de manera habitual
9. No contesta

Alcohol

P.35. ¿Ha bebido usted en estas dos últimas semanas alguna bebida alcohólica?

4. Sí, diariamente → pasar a P.35.a
5. Sí, pero no diariamente. → pasar a P.35.a
6. No
9. NS/NC

P.35.a. Durante las **últimas 2 semanas** ¿alguna vez ha tomado 6/5 (hombre/mujer, respectivamente) (5/4 PARA MAYORES DE 65 AÑOS) o más bebidas estándar en una misma ocasión? (Por "ocasión" entendemos tomar las bebidas en un intervalo aproximado de cuatro a seis horas)."

4. Sí, alguna vez
5. Sí, varias veces
6. No
8. No sabe
9. No contesta

P.36. Durante las últimas 2 semanas, ¿ha utilizado la bebida para calmar su ansiedad?

1. Totalmente cierto
2. Bastante cierto
3. Bastanto falso
4. Totalmente falso
5. NS/NC

Sueño

P.37. ¿Podría indicarme, aproximadamente, cuántas horas al día duerme habitualmente?

Horas:	|_|_|
NS/NC	99

P.37.a. Las horas que duerme, ¿le permiten descansar lo suficiente?

3. Sí
4. No
8. NS
9. NC

Vacunas

P.38. Pasando a otro tema, querríamos saber si se ha vacunado de la gripe en la última campaña 2019

4. Sí
5. No
6. NS/NC

P.38.a. ¿Y en la campaña de 2020?

5. Sí
6. No, pero tengo intención de vacunarme
7. No y no tengo intención de vacunarme

8. NS/NC

P.38.b. ¿Y se ha vacunado de COVID-19?

5. Sí
6. No, pero tengo intención de vacunarme
7. No y no tengo intención de vacunarme
8. NS/NC

BLOQUE IV. EFECTOS ECONÓMICOS Y CARACTERÍSTICAS SOCIODEMOGRÁFICAS Y SOCIOECONÓMICAS.

<u>Situación laboral</u>

P.39. ¿Ha cambiado su situación laboral desde que realizó la última encuesta?

4. Sí → pasar a P.40.
5. No → pasar a P.39.a.
6. No lo recuerdo/No estoy seguro/a → pasar a P.40.

P.40. ¿En qué situación de las siguientes se encuentra en la actualidad? (Si se encuentra en varias situaciones, indique solo la que considere principal).

1.Asalariado/a a tiempo completo.
2.Asalariado/a a tiempo completo en ERTE
3.Asalariado/a a tiempo parcial
4.Asalariado/a a tiempo parcial por ERTE
5.Empresario/a con asalariados/as
6.Empresario/a sin asalariados/as o trabajador/a independiente o miembro de cooperativa
7.Trabajos familiares no remunerados (ayudas familiares).
8.Parado/a
9.Estudiante
10.Jubilado/a, retirado/a o jubilado/a anticipado/a
11.Incapacitado/a permanente
12.Realizando tareas de voluntariado social
13.Dedicado/a a las labores del hogar, al cuidado de niños u otras personas. 10
14.Otra clase de inactividad económica
98.No sabe
98.No contesta

P.40.a. En su empleo principal o en su último empleo ¿cuál es/era la ocupación, profesión u oficio que desempeña/ba?

Precise lo más posible, por ejemplo: dependiente de comercio, mecánico de automóviles, tornero, peón de la construcción, operador de grúa, etc.). Entrevistador: Anote el literal y señale la opción que corresponda según la tabla de clasificación del manual. El informante debe especificar suficientemente para que se pueda codificar correctamente.
Ocupación: ______________________

Filtro: P40=1,2,3,4
P.41. ¿Qué tipo de contrato tiene en la actualidad?
1.Contrato indefinido
2.Contrato temporal.

Para trabajadores: Filtro: P40=1,2,3,4,5,6,7

P.42. Respecto a la situación en la que desarrolla su trabajo ¿cómo es **actualmente**?

1. Trabajo desde casa
2. Trabajo fuera de casa
3. Trabajo desde casa y desde fuera de casa
4. Estoy de permiso para ocuparme de mis hijos
5. Estoy permiso para ocuparme de otros familiares
6. Estoy de vacaciones anuales retribuidas
7. Estoy de permiso no retribuido
8. Otra situación

Para trabajadores Filtro: P40=1,2,3,4,5,6,7
P.43. Asumiendo que su capacidad laboral en su mejor estado de salud tuviera un valor de 10 puntos, ¿cuántos puntos daría a su capacidad laboral actual? 0 (totalmente incapacitado) …10 (muy buena capacidad laboral)

0 1 2 3 4 5 6 7 8 9 10 Ns/Nc 99

Filtro: P40=8
P.44. Me ha dicho que está Vd. en situación de desempleo. ¿En cuál de las siguientes situaciones se encuentra actualmente?

1. Estoy desempleado por un ERTE debido al coronavirus.
2. Estoy desempleado por un despido debido al coronavirus (sin ERTE).
3. He tenido que cerrar mi negocio.
4. He tenido que cesar la actividad que me aportaba ingresos
5. Tenía opciones de comenzar en un trabajo, pero se han pospuesto/cancelado debido al estado de alarma
6. Mi situación de desempleo es previa al estado de alarma y no se ha visto alterada
7. Otras situaciones:

P.39.a ¿Ha cambiado su nivel de estudios desde que realizó la anterior encuesta?

4. Sí → pasar a P.40.b.
5. No → pasar a P.46.
6. No lo recuerdo/No estoy seguro/a → pasar a P.40.b.

P.40.b. ¿Cuáles son sus estudios terminados de más alto nivel?

Anote el literal y señale la opción que corresponda. El informante debe especificar en su respuesta lo suficiente como para que se pueda codificar correctamente, por ejemplo: ha asistido menos de cinco años a la escuela, tres cursos completos de Ciencias Económicas y Empresariales, doctorado en Ciencias Sociales, etc.
Estudios terminados de más alto nivel: ______________________
Agente CATI: no leer. Clasificar en función del literal contestado

1. Analfabetos/as y estudios primarios incompletos.
2. Educación primaria.
3. Primera etapa de la Educación Secundaria y similar.
4. Segunda etapa de la Educación Secundaria y similar
5. Bachillerato y FP de Grado Medio.
6. Educación postsecundaria no superior.
7. Formación Profesional de Grado Superior y títulos propios De universidades de duración igual o superior a 2 años.
8. Diplomados/as universitarios/as, títulos propios universitarios de Experto o especialista y similares (Grados universitarios de 240 créditos ECTS, Bolonia).

9. Licenciados, másteres y especialidades en Ciencias de la Salud por el sistema de residencia y similares. Grados universitarios de más 240 créditos ECTS (Bolonia).
10. Título de Doctorado.
11. No se puede codificar.

Identificación de la persona de referencia del hogar (la que aporta más ingresos)

P.45. Actualmente, la persona que aporta más ingresos al hogar, ¿es la misma que cuando le hicieron la anterior encuesta?

1. SÍ → pasar a P.50.
2. NO → pasar a P.46.
3. No lo recuerdo/No estoy seguro/a → pasar a P.46.

P.46. Actualmente, ¿En su hogar quién es la persona que aporta más ingresos?

1. Yo (ir a P.50)
2. Otra persona (ir a P.47)
3. Otra persona y yo a partes iguales (ir a P.47)

P.47. Y esa persona es...

1. Su cónyuge/pareja
2. Su padre
3. Su madre
4. Otra persona.
5. No Contesta

P.48. ¿Cuáles son los estudios terminados de más alto nivel de la persona que aporta más ingresos en el hogar?

Anote el literal y señale la opción que corresponda. El informante debe especificar en su respuesta lo suficiente como para que se pueda codificar correctamente, por ejemplo: ha asistido menos de cinco años a la escuela, tres cursos completos de Ciencias Económicas y Empresariales, doctorado en Ciencias Sociales, etc.
Estudios terminados de más alto nivel: ______________________
Agente CATI: no leer. Clasificar en función del literal contestado

1. Analfabetos/as y estudios primarios incompletos.
2. Educación primaria.
3. Primera etapa de la Educación Secundaria y similar.
4. Segunda etapa de la Educación Secundaria y similar
5. Bachillerato y FP de Grado Medio.
6. Educación postsecundaria no superior.
7. Formación Profesional de Grado Superior y títulos propios De universidades de duración igual o superior a 2 años.
8. Diplomados/as universitarios/as, títulos propios universitarios de Experto o especialista y similares (Grados universitarios de 240 créditos ECTS, Bolonia).
9. Licenciados, másteres y especialidades en Ciencias de la Salud por el sistema de residencia y similares. Grados universitarios de más 240 créditos ECTS (Bolonia).
10. Título de Doctorado.
11. No se puede codificar.

P.49. ¿En qué situación de las siguientes se encuentra en la actualidad la persona que aporta más ingresos al hogar? (Si se encuentra en varias situaciones, indique solo la que considere principal).

1. Asalariado/a a tiempo completo
2. Asalariado/a a tiempo completo en ERTE

3. Asalariado/a a tiempo parcial
4. Asalariado/a a tiempo parcial por ERTE
5. Empresario/a con asalariados/as
6. Empresario/a sin asalariados/as o trabajador/a independiente o miembro de cooperativa
7. Trabajos familiares no remunerados (ayudas familiares).
8. Parado/a
9. Estudiante
10. Jubilado/a, retirado/a o jubilado/a anticipado/a
11. Incapacitado/a permanente
12. Realizando tareas de voluntariado social
13. Dedicado/a a las labores del hogar, al cuidado de niños u otras personas.
14. Otra clase de inactividad económica
98. No sabe
99. No contesta

P.49.a. En su empleo principal o en su último empleo ¿cuál es/era la ocupación, profesión u oficio que desempeña/ba la persona que aporta más ingresos al hogar?

Precise lo más posible, por ejemplo: dependiente de comercio, mecánico de automóviles, tornero, peón de la construcción, operador de grúa, etc.). Entrevistador: Anote el literal y señale la opción que corresponda según la tabla de clasificación del manual. El informante debe especificar suficientemente para que se pueda codificar correctamente
Ocupación: ___________________

Ingresos y situación económica del hogar

P.50. Un hogar puede tener diferentes fuentes de ingresos y más de un miembro del hogar puede contribuir con sus ingresos. En relación con el total de ingresos de su hogar, ¿cómo suelen llegar a fin de mes?

1. Con mucha facilidad.
2. Con facilidad.
3. Con cierta facilidad.
4. Con cierta dificultad.
5. Con dificultad
6. Con mucha dificultad
7. No sabe.
8. No contesta.

P.51. Desde que comenzó la crisis sanitaria ¿ha tenido en su hogar algún retraso en el pago de recibos como la hipoteca, el alquiler, o suministros como la luz, el agua, impuestos, etc.? (Respuesta múltiple).

1. Hipoteca
2. Alquiler
3. Cuotas de compras aplazadas
4. Cuotas de seguros
5. Impuestos (IBI, los relacionados con vehículos, etc.)
6. Pagos de recibos de agua, gas, calefacción, electricidad, comunidad
7. Tasa de autónomo
8. Pago de colegios
9. Actividades extraescolares
10. Otros

P.51.a. ¿Se ha resuelto? Filtro si en p51=si

3. Sí
4. No

P.52. ¿Ha cambiado su situación económica desde que realizó la primera encuesta (**hace un año**)?

5. Sí, ha mejorado → pasar a P.53.
6. Sí, ha empeorado→ pasar a P.53.
7. No → pasar a P.54.
8. No lo recuerdo/Ne estoy seguro/a → pasar a P.53.

P.53. Dígame en qué intervalo, de los que le leeré a continuación, se encuentran los ingresos mensuales netos del conjunto de miembros de su hogar EN EL MES ANTERIOR

1. Sin ingresos (no leer)
2. Menos de 450 euros
3. De 451 a 900 euros
4. De 901 a 1.600 euros
5. De 1.601 a 2.500 euros
6. De 2.501 a 3.000 euros
7. Más de 3.000 euros

98. NS
98. NC

Observaciones de la entrevista: ____

Esto es todo, muchas gracias por su colaboración y por el tiempo que nos ha dedicado respondiendo esta encuesta. Le recuerdo que tiene a su disposición el número de teléfono **900 10 17 49** por si necesita resolver cualquier duda o información.

Medición 2 - ESSOCzonas. Cuestionario muestra nueva

- BLOQUE I. CARACTERÍSTICAS DEL HOGAR Y LA VIVIENDA
- BLOQUE II. SALUD, BIENESTAR EMOCIONAL
- BLOQUE III: HÁBITOS Y ESTILOS DE VIDA
- BLOQUE IV. SITUACIÓN ECONÓMICA Y CARACTERÍSTICAS SOCIODEMOGRÁFICAS.

BLOQUE I. CARACTERÍSTICAS DEL HOGAR Y LA VIVIENDA

P.2. ¿Actualmente se encuentra en su vivienda habitual o reside en una vivienda distinta?

1. Habitual → P.3.
2. Segunda residencia → P.2.a
3. Otras: → P.2.a

P.2.a. ¿En qué municipio se encuentra actualmente: Municipio: _________

P.3. La vivienda en la que se encuentra actualmente es una:

1. Vivienda unifamiliar independiente, adosada o pareada
2. Piso o apartamento en un edificio con menos de 10 viviendas

3. Piso o apartamento en un edificio con 10 o más viviendas
4. Otro tipo de vivienda (Ej: Vivienda situada en un edificio destinado principalmente a otros fines barraca, cabaña, chabola, etc.) Especifique.
5. NS/NC

P.4. ¿Cuál es la superficie útil de la vivienda en la que se encuentra actualmente?

1. Menos de 46 m^2 útiles
2. Entre 46 y 60
3. Entre 61 y 75
4. Entre 76 y 90
5. Entre 91 y 120
6. Más de 120
7. No sabe
8. No contesta

P.7. Para conocer las condiciones en las que está actualmente me podría decir ¿qué número de personas residen actualmente incluyéndose usted?
Número de personas: |_|_|

P.7.a. ¿De estas personas cuántas son mayores de 60 años?...... |_|_|
P.7.b. ¿Y cuántas son menores de 16 años?... |_|_|
P.7.c. ¿De las personas menores de 16, cuántas son menores de 6 años? I_I_I

P.8. A efectos de caracterizar su situación de convivencia, ¿me podría decir cuál de las siguientes alternativas lo refleja mejor?

1. Hogar unipersonal
2. Padre o madre solo/a que conviva con algún hijo/a
3. Pareja sin hijos que convivan en el hogar
4. Pareja con hijos/as que convivan en el hogar
5. Otro tipo de hogar
6. NC

P.11. ¿Cuántas habitaciones tiene su vivienda? (no contar la cocina, los baños/lavabos o el comedor/sala de estar).
Número de habitaciones: |_|_|

P.12. Indique el número de personas (<16; 16-59; >=60) que presentan alguna de las condiciones que voy a leerle…

P.12.a. Tener alguna limitación, discapacidad de tipo físico, sensorial o psíquico o mental

5. SI, de que tramo de edad:
   - <16 |_|_|
   - 16-59 |_|_|
   - >=60 |_|_|
6. No

P.12.b. Requiere algún tipo de dedicación o de cuidados especiales

5. SI, de que tramo de edad:
   - <16 |_|_|
   - 16-59 |_|_|
   - >=60 |_|_|

6. No

Cuidado y uso del tiempo

P.12.C. Por favor, indique quienes de las siguientes personas realizan las siguientes acciones en su hogar actualmente:

21. Siempre yo
22. Habitualmente yo
23. Mi pareja y yo por igual
24. Habitualmente mi pareja
25. Siempre mi pareja
26. Madre de la persona entrevistada
27. Padre de la persona entrevistada
28. Persona remunerada
29. Otras personas. Especificar
30. No sabe/No procede

1. Tareas domésticas (limpieza, cocina, etc…)
2. Tareas de cuidado de hijos/as u otros menores del hogar (general):
   g. Tareas de cuidado físico (alimentación, vestido, higiene, vigilancia)
   h. Tareas de cuidado interactivo (enseñar, leer, jugar, conversar y otras interacciones)
   i. Acompañamiento y transporte de hijos/as a diferentes espacios
3. Tareas de cuidados en adultos con problemas de salud, movilidad, etc. que requieren algún tipo de dedicación o de cuidados especiales
4. Tareas en menores con necesidades especiales de cuidado (problemas de salud, movilidad, etc. que requieren algún tipo de dedicación o de cuidados especiales).

BLOQUE II. SALUD, BIENESTAR EMOCIONAL

Identificación de la infección

P.13. En primer lugar ¿tiene o ha tenido diagnóstico de coronavirus?

1. Sí →pasar a P.13.a
2. No
3. Estoy a la espera del resultado
4. No sabe
5. No contesta

Filtro si 1 en P.13

P.13.a. ¿Cómo de grave ha sido o es su proceso con la enfermedad?

1. Muy leve
2. Leve
3. Grave
4. Muy grave

P.14. ¿Le han hecho alguna vez, alguna de las siguientes pruebas para ver si estaba infectado por el coronavirus? (MULTIRRESPUESTA)

1. Muestra de la garganta o la nariz Sí/No/NC
2. Muestra de sangre en el dedo Sí/No/NC

3. Muestra de sangre en vena Sí/No/NC

P.15. ¿Hay en su entorno alguien que tiene o ha tenido algún caso diagnosticado de infección por coronavirus? Respuesta múltiple

1. Persona/s de mi familia con la que resido en el hogar
2. Persona/s con la que resido en el hogar que no es familiar
3. Familiar/es que no conviven conmigo
4. Amigos, compañeros de trabajo/estudios, vecinos con lo que no resido en el hogar
5. Nadie de mi entorno
6. NC

Hablemos ahora de forma general de cómo se encuentra…

Autopercepción de salud

P.16. En la actualidad, en general diría que su salud es...

15. Excelente
16. Muy buena
17. Buena
18. Regular
19. Mala
20. No sabe/No está seguro.
21. No contesta.

P.17. En la actualidad, en general diría que su salud mental es...

15. Excelente
16. Muy buena
17. Buena
18. Regular
19. Mala
20. No sabe/No está seguro.
21. No contesta.

Bienestar emocional

P.18. A continuación, le voy a leer una lista de sentimientos o comportamientos que quizás haya tenido Vd. **durante la última semana**. Por favor, dígame con qué frecuencia, se ha sentido...

1. En ningún momento o en casi ningún momento
2. En algún momento,
3. Buena parte del tiempo,
4. Todo o casi todo el tiempo
5. No sabe

1. Deprimido/a
2. Feliz
3. Solo/a
4. Ha tenido la sensación de disfrutar de la vida
5. Rebosante de energía
6. Tranquilo/a y relajado/a

P.19. Si toma también como referencia **la última semana**, dígame Ud. su grado de acuerdo o desacuerdo con las siguientes frases.

1. Muy de acuerdo

2. De acuerdo
3. Ni de acuerdo ni en desacuerdo
4. En desacuerdo
5. Muy en desacuerdo
6. No sabe

1. He sido optimista respecto a mi futuro
2. Por lo general me he sentido bien conmigo mismo

Felicidad y apoyo social y afectivo

P.22. En términos generales, ¿en qué medida se considera Ud. una persona feliz o infeliz? Por favor, use una escala de 0 a 10, en la que 0 significa que se considera "completamente infeliz" y 10 que se considera "completamente feliz".

0 1 2 3 4 5 6 7 8 9 10 Ns/Nc 99

P.23. En la siguiente lista se muestran algunas cosas que otras personas hacen por nosotros o nos proporcionan. Elija para cada una la respuesta que mejor refleje su situación:

11. Tanto como deseo
12. Casi como deseo
13. Ni mucho ni poco
14. Menos de lo que deseo
15. Mucho menos de lo que deseo

P.23.1. Recibo visitas/llamadas de mis amigos y familiares
P.23.2. Recibo ayuda en asuntos relacionados con mi casa
P.23.3. Recibo elogios y reconocimientos cuando hago bien mi trabajo
P.23.4. Cuento con personas que se preocupan de lo que me sucede
P.23.5. Recibo amor y afecto
P.23.6. Tengo la posibilidad de hablar con alguien de mis problemas en el trabajo y/o en la casa
P.23.7. Tengo la posibilidad de hablar con alguien de mis problemas personales y familiares
P.23.8. Tengo posibilidad de hablar con alguien de mis problemas económicos
P.23.9. Recibo invitaciones para distraerme y salir con otras personas
P.23.10. Recibo consejos útiles cuando me ocurre algún acontecimiento importante en mi vida
P.23.11. Recibo ayuda cuando estoy enfermo/a en la cama

Molestias y enfermedades crónicas

P.24. Durante el **último mes**, ¿hasta qué punto ha notado Vd. alguna de las siguientes molestias? Utilice una escala del 1 al 5 en la que 1 es Nunca he sentido esa molestia y 5 Con mucha frecuencia.

1. Problemas de sueño
2. Problemas de estómago
3. Incremento de la tensión arterial
4. Mareos
5. Dolores de cabeza
6. Dolores musculares o de articulaciones
6a. Otros dolores
7. Sensación de opresión en el pecho
8. Otra molestia

P.25. ¿Le ha dicho un/a profesional de la salud (médico/a, enfermero/a) que usted padece actualmente alguna de las siguientes enfermedades crónicas? Si responde "SI" hacer P.25.a. ¿Esa condición le limita alguna de sus actividades?

ENFERMEDAD CRÓNICA	P.25. ¿Le ha dicho un/a profesional de la salud si tiene esta condición? (si es 'No', saltar a la siguiente)			P.25.a ¿Esa condición le limita alguna de sus actividades?	
	Sí	No	No lo sabe	Sí	No
27. Diabetes/azúcar alta en sangre/azúcar en orina	☐	☐	☐	☐	☐
28. Hipertensión/presión arterial alta	☐	☐	☐	☐	☐
29. Colesterol alto	☐	☐	☐	☐	☐
30. Enfermedad crónica del pulmón / enfisema / bronquitis crónica	☐	☐	☐	☐	☐
31. Asma	☐	☐	☐	☐	☐
32. Enfermedades del corazón (Trastornos cardíacos/insufiencia cardíaca /insuficiencia cardíaca congestiva; angina de pecho; Infarto de miocardio/ataque al corazón)	☐	☐	☐	☐	☐
33. Dolor crónico (pecho; espalda, cuello, hombro, cintura (cervical/lumbar; Migrañas/Jaquecas/ Cefaleas crónicas / dolor de cabeza frecuente)	☐	☐	☐	☐	☐
34. Alergias crónicas (como rinitis, inflamación de ojos, dermatitis, alergia a los alimentos, etc), asma alérgica excluida	☐	☐	☐	☐	☐
35. Mala circulación	☐	☐	☐	☐	☐
36. Varices en las piernas	☐	☐	☐	☐	☐
37. Trastornos depresivos	☐	☐	☐	☐	☐
38. Trastornos de ansiedad	☐	☐	☐	☐	☐
39. Otra enfermedad crónica	☐	☐	☐	☐	☐

Medicamentos

P.26. ¿En qué medida se ha visto modificada la medicación que usted tomaba antes del último mes con respecto a ahora?

1. He disminuido mi medicación
2. Sigo igual, no ha afectado en nada
3. He aumentado mi medicación (incluye si antes no consumía, pero ahora sí)
4. No consumo medicación.

Filtro si 1,2 o 3 en la P.26.

P.26.a. Y En general, ha cambiado la manera en que toma sus medicamentos

1. Sí, ahora estoy cumpliendo mejor con la medicación
2. Sí, ahora estoy cumpliendo peor con la medicación
3. Sigo tomando los mismos medicamentos de la misma manera

P.27. (PARA TODOS). Y en las **últimas 2 semanas**, ¿ha utilizado algún tipo de medicamento (gotas, pastillas, inyecciones, supositorios, etc.)?

1. Sí

2. No
9. N/C

Filtro Si 1 en P.27.
P.27.a. Si ha tomado medicamentos, ¿Qué tipo de medicamento?

27. Medicinas para el catarro, gripe, garganta, bronquios
28. Medicinas para el dolor
29. Medicinas para para bajar la fiebre
30. Antibióticos
31. Tranquilizantes, sedantes
32. Pastillas para dormir
33. Medicamentos antidepresivos
34. Medicamentos para la alergia
35. Medicinas para el corazón
36. Medicinas para la tensión arterial
37. Medicamentos para la diabetes
38. Medicamentos para bajar el colesterol
39. Otros medicamentos

BLOQUE III: HÁBITOS Y ESTILOS DE VIDA

P.28. En relación al último mes, diría Vd. que…

	Mas	Igual	Menos	No procede	Ns/Nc
• 28.1 He realizado más, igual o menos ejercicio	1	2	3	8	9
• 28.2 He consumido más, igual o menos bebidas alcohólicas	1	2	3	8	9
• 28.3 He fumado más, igual, o menos	1	2	3	8	9

	Mejor	Igual	Peor	Ns/Nc
ido mejor, igual o peor	1	2	3	9
• Me he alimentado mejor, igual o peor que antes	1	2	3	9

Mi peso ha aumentado, se ha mantenido o ha disminuido

1. Aumentado
2. Mantenido
3. Disminuido
9. Ns/Nc

<u>Alimentación</u>

P.29. ¿Cuántas raciones de verdura u hortalizas consume al día (las guarniciones o acompañamientos contabilizan como ½ ración)?

1. Dos o más al día (al menos una de ellas en ensalada o crudas)
2. Dos o más al día (aunque ninguna de ellas en ensalada o crudas)
3. Diariamente, aunque menos de dos al día
4. No diariamente, pero tres o más por semana
5. Menos de tres veces por semana
6. No consume

9. NS/NC

P.30. ¿Cuántas piezas de fruta (incluyendo zumo natural) consume al día?

1. Tres o más al día
2. Diariamente, aunque menos de tres veces al día
3. No diariamente, pero tres o más veces por semana
4. Menos de tres veces por semana
5. No consume
9. NS/NC

Actividad física

P.31. Durante las **últimas dos semanas**, ¿Cuántos días a la semana practica normalmente durante más de 30 minutos …? *(incluyendo desplazamientos al trabajo o actividades similares)*

Nº de días/sem

9. Caminar ……………………………………………………………………….. |__|__|
10. Una actividad física ligera como gimnasia suave, juegos que requieren poco esfuerzo y similares …….……………………………………………….………. |__|__|
11. Una actividad física moderada como montar en bicicleta, gimnasia, aeróbic, correr, natación ……………………………………………………………….… |__|__|
12. Entrenamiento físico deportivo como fútbol, baloncesto, ciclismo, natación de competición, judo, kárate o similares ……......................................……........ |__|__|

P.32. ¿Podría decirme cuánto pesa, aproximadamente, sin zapatos ni ropa? (si está embarazada en la actualidad, debe indicar cuánto pesaba antes del inicio del embarazo)

Kilos: |_|_|_|
No sabe 998
No contesta 999

P.33.¿Y cuánto mide, aproximadamente, sin zapatos?

Cms.: |_|_|_|
No sabe 998
No contesta 999

Tabaco

P.34. Cambiando de tema, ¿podría usted decirme si fuma?

9. Sí, fuma diariamente
10. Sí fuma, pero no diariamente
11. No fuma, pero ha fumado
12. No fuma ni ha fumado nunca de manera habitual
9. No contesta

Alcohol

P.35. ¿Ha bebido usted en estas dos últimas semanas alguna bebida alcohólica?

7. Sí, diariamente .... → pasar a P.35.a
8. Sí, pero no diariamente............ → pasar a P.35.a
9. No ....
9. NS/NC

P.35.a. Durante las **últimas 2 semanas** ¿alguna vez ha tomado 6/5 (hombre/mujer, respectivamente) (5/4 PARA MAYORES DE 65 AÑOS) o más bebidas estándar en una misma

ocasión? (Por “ocasión” entendemos tomar las bebidas en un intervalo aproximado de cuatro a seis horas).”

7. Sí, alguna vez
8. Sí, varias veces
9. No

8. No sabe
9. No contesta

P.36. Durante las últimas 2 semanas, ¿ha utilizado la bebida para calmar su ansiedad?

1. Totalmente cierto
2. Bastante cierto
3. Bastanto falso
4. Totalmente falso

Sueño

P.37. ¿Podría indicarme, aproximadamente, cuántas horas al día duerme habitualmente?

Horas: |_|_|
NS/NC 99

P.37.a. Las horas que duerme, ¿le permiten descansar lo suficiente?

5. Sí
6. No
8. N/S
9. N/C

Vacunas

P.38. Pasando a otro tema, querríamos saber si se ha vacunado de la gripe en la última campaña 2019

7. Sí
8. No
9. NS/NC

P.38.a. ¿Y en la campaña de 2020?

9. Sí
10. No, pero tengo intención de vacunarme
11. No, y no tengo intención de vacunarme
12. NS/NC

P.38.b. ¿Y se ha vacunado de COVID-19?

9. Sí
10. No, pero tengo intención de vacunarme
11. No y no tengo intención de vacunarme
12. NS/NC

BLOQUE IV. EFECTOS ECONÓMICOS Y CARACTERÍSTICAS SOCIODEMOGRÁFICAS Y SOCIOECONÓMICAS.

Situación laboral

Para poder clasificar las respuestas que usted nos ha dado necesitamos que nos conteste las siguientes preguntas.

P.40. ¿En qué situación de las siguientes se encuentra en la actualidad? (Si se encuentra en varias situaciones, indique solo la que considere principal).

1.Asalariado/a a tiempo completo.
2.Asalariado/a a tiempo completo en ERTE
3.Asalariado/a a tiempo parcial
4.Asalariado/a a tiempo parcial por ERTE
5.Empresario/a con asalariados/as
6.Empresario/a sin asalariados/as o trabajador/a independiente o miembro de cooperativa
7.Trabajos familiares no remunerados (ayudas familiares).
8.Parado/a
9.Estudiante
10.Jubilado/a, retirado/a o jubilado/a anticipado/a
11.Incapacitado/a permanente
12.Realizando tareas de voluntariado social
13.Dedicado/a a las labores del hogar, al cuidado de niños u otras personas. 10
14.Otra clase de inactividad económica
98.No sabe
98.No contesta

P.40.a. En su empleo principal o en su último empleo ¿cuál es/era la ocupación, profesión u oficio que desempeña/ba?

Precise lo más posible, por ejemplo: dependiente de comercio, mecánico de automóviles, tornero, peón de la construcción, operador de grúa, etc.). Entrevistador: Anote el literal y señale la opción que corresponda según la tabla de clasificación del manual. El informante debe especificar suficientemente para que se pueda codificar correctamente.
Ocupación: _____________

P.40.b. ¿Cuáles son sus estudios terminados de más alto nivel?

Anote el literal y señale la opción que corresponda. El informante debe especificar en su respuesta lo suficiente como para que se pueda codificar correctamente, por ejemplo: ha asistido menos de cinco años a la escuela, tres cursos completos de Ciencias Económicas y Empresariales, doctorado en Ciencias Sociales, etc.
Estudios terminados de más alto nivel: ______________________
Agente CATI: no leer. Clasificar en función del literal contestado

1. Analfabetos/as y estudios primarios incompletos.
2. Educación primaria.
3. Primera etapa de la Educación Secundaria y similar.
4. Segunda etapa de la Educación Secundaria y similar
5. Bachillerato y FP de Grado Medio.
6. Educación postsecundaria no superior.
7. Formación Profesional de Grado Superior y títulos propios De universidades de duración igual o superior a 2 años.
8. Diplomados/as universitarios/as, títulos propios universitarios de Experto o especialista y similares (Grados universitarios de 240 créditos ECTS, Bolonia).
9. Licenciados, másteres y especialidades en Ciencias de la Salud por el sistema de residencia y similares. Grados universitarios de más 240 créditos ECTS (Bolonia).
10. Título de Doctorado.
11. No se puede codificar.

Filtro: P40=1,2,3,4
P.41. ¿Qué tipo de contrato tiene en la actualidad?
1.Contrato indefinido
2.Contrato temporal.

Para trabajadores: Filtro: P40=1,2,3,4,5,6,7
P.42. Respecto a la situación en la que desarrolla su trabajo ¿cómo es **actualmente**?
1. Trabajo desde casa
2. Trabajo fuera de casa
3. Trabajo desde casa y desde fuera de casa
4. Estoy de permiso para ocuparme de mis hijos
5. Estoy permiso para ocuparme de otros familiares
6. Estoy de vacaciones anuales retribuidas
7. Estoy de permiso no retribuido
8. Otra situación

Para trabajadores Filtro: P40=1,2,3,4,5,6,7
P.43. Asumiendo que su capacidad laboral en su mejor estado de salud tuviera un valor de 10 puntos, ¿cuántos puntos daría a su capacidad laboral actual? 0 (totalmente incapacitado) …10 (muy buena capacidad laboral)

0 1 2 3 4 5 6 7 8 9 10 Ns/Nc 99

Filtro: P40=8
P.44. Me ha dicho que está Vd. en situación de desempleo. ¿En cuál de las siguientes situaciones se encuentra actualmente?
1. Estoy desempleado por un ERTE debido al coronavirus.
2. Estoy desempleado por un despido debido al coronavirus (sin ERTE).
3. He tenido que cerrar mi negocio.
4. He tenido que cesar la actividad que me aportaba ingresos
5. Tenía opciones de comenzar en un trabajo, pero se han pospuesto/cancelado debido al estado de alarma
6. Mi situación de desempleo es previa al estado de alarma y no se ha visto alterada
7. Otras situaciones:

Identificación de la persona de referencia del hogar (la que aporta más ingresos)

P.46. Actualmente, ¿En su hogar quién es la persona que aporta más ingresos?
1. Yo (ir a P.50.)
2. Otra persona (ir a P.47)
3. Otra persona y yo a partes iguales (ir a P.47)

P.47. Y esa persona es...
1. Su cónyuge/pareja
2. Su padre
3. Su madre
4. Otra persona.
5. No Contesta

P.48. ¿Cuáles son los estudios terminados de más alto nivel de la persona que aporta más ingresos en el hogar?
1. Analfabetos/as y estudios primarios incompletos.
2. Educación primaria.
3. Primera etapa de la Educación Secundaria y similar.

4. Segunda etapa de la Educación Secundaria y similar
5. Bachillerato y FP de Grado Medio.
6. Educación postsecundaria no superior.
7. Formación Profesional de Grado Superior y títulos propios De universidades de duración igual o superior a 2 años.
8. Diplomados/as universitarios/as, títulos propios universitarios de Experto o especialista y similares (Grados universitarios de 240 créditos ECTS, Bolonia).
9. Licenciados, másteres y especialidades en Ciencias de la Salud por el sistema de residencia y similares. Grados universitarios de más 240 créditos ECTS (Bolonia).
10. Título de Doctorado.
11. No se puede codificar.

P.49. ¿En qué situación de las siguientes se encuentra en la actualidad la persona que aporta más ingresos al hogar? (Si se encuentra en varias situaciones, indique solo la que considere principal).

1. Asalariado/a a tiempo completo
2. Asalariado/a a tiempo completo en ERTE
3. Asalariado/a a tiempo parcial
4. Asalariado/a a tiempo parcial por ERTE
5. Empresario/a con asalariados/as
6. Empresario/a sin asalariados/as o trabajador/a independiente o miembro de cooperativa
7. Trabajos familiares no remunerados (ayudas familiares).
8. Parado/a
9. Estudiante
10. Jubilado/a, retirado/a o jubilado/a anticipado/a
11. Incapacitado/a permanente
12. Realizando tareas de voluntariado social
13. Dedicado/a a las labores del hogar, al cuidado de niños u otras personas.
14. Otra clase de inactividad económica
98. No sabe
99. No contesta

P.49.a. En su empleo principal o en su último empleo ¿cuál es/era la ocupación, profesión u oficio que desempeña/ba la persona que aporta más ingresos al hogar?

Precise lo más posible, por ejemplo: dependiente de comercio, mecánico de automóviles, tornero, peón de la construcción, operador de grúa, etc.). Entrevistador: Anote el literal y señale la opción que corresponda según la tabla de clasificación del manual. El informante debe especificar suficientemente para que se pueda codificar correctamente
Ocupación: ___________________

Ingresos y situación económica del hogar

P.50. Un hogar puede tener diferentes fuentes de ingresos y más de un miembro del hogar puede contribuir con sus ingresos. En relación con el total de ingresos de su hogar, ¿cómo suelen llegar a fin de mes?

1. Con mucha facilidad.
2. Con facilidad.
3. Con cierta facilidad.
4. Con cierta dificultad.
5. Con dificultad
6. Con mucha dificultad

7. No sabe.
8. No contesta.

P.51. Desde que comenzó la crisis sanitaria, ¿ha tenido en su hogar algún retraso en el pago de recibos como la hipoteca, el alquiler, o suministros como la luz, el agua, impuestos, etc.? (Respuesta múltiple).

1. Hipoteca
2. Alquiler
3. Cuotas de compras aplazadas
4. Cuotas de seguros
5. Impuestos (IBI, los relacionados con vehículos, etc.)
6. Pagos de recibos de agua, gas, calefacción, electricidad, comunidad
7. Tasa de autónomo
8. Pago de colegios
9. Actividades extraescolares
10. Otros

P.51 a ¿Se ha resuelto? Filtro si en p51=si

9. Sí
10. No

P.53. Dígame en qué intervalo, de los que le leeré a continuación, se encuentran los ingresos mensuales netos del conjunto de miembros de su hogar EN EL MES ANTERIOR

1. Sin ingresos (no leer)
2. Menos de 450 euros
3. De 451 a 900 euros
4. De 901 a 1.600 euros
5. De 1.601 a 2.500 euros
6. De 2.501 a 3.000 euros
7. Más de 3.000 euros
98. NS
98. NC

P52a. ¿Cuáles son los estudios terminados de más alto nivel de su padre?
P52b. ¿Y de su madre?

- Primarios o menos
- Secundarios
- Universitarios
- No sabe
- No contesta

Observaciones de la entrevista: ____

Esto es todo, muchas gracias por su colaboración y por el tiempo que nos ha dedicado respondiendo esta encuesta. Le recuerdo que tiene a su disposición el número de teléfono **900 10 17 49** por si necesita resolver cualquier duda o información.

5. Cuestionarios de ESSOC+55

La muestra de mayores de 55 años está compuesta por dos mediciones. Los cuestionarios están basados en la 3ª medición de la ESSOCgeneral con algunas modificaciones en relación a la población especifica de mayores y al uso de las tecnologías.

Medición 1 – ESSOC+55. Cuestionario

La información se organiza en 5 grandes bloques:

- BLOQUE INICIAL. INFORMACIÓN DE LA PERSONA ENTREVISTADA Y DEL AGENTE GUADALINFO
- BLOQUE I. CARACTERÍSTICAS DEL HOGAR Y LA VIVIENDA
- BLOQUE II. SALUD, BIENESTAR EMOCIONAL
- BLOQUE III: HÁBITOS Y ESTILOS DE VIDA
- BLOQUE IV. SITUACIÓN ECONÓMICA Y CARACTERÍSTICAS SOCIODEMOGRÁFICAS.

Datos de la persona entrevistada y Agente Guadalinfo

1. Sexo de la persona entrevistada
   - Femenino
   - Masculino

2. Edad de la persona entrevistada
(Si no contesta a la pregunta poner 999)
(El entrevistador dispone de los datos de edad y sexo previamente a la entrevista, ya que el muestreo se ha realizado por grupo de edad y sexo)
Cifra mínima: 49

3. Código Persona entrevistada

4. Tipo de Centro Guadalinfo.
   - Centro Guadalinfo
   - Centros CAPI
   - Centro CAE
   - Centros ELA
   - Telecentros
   - Otro:

5. Nombre del Agente Guadalinfo que realiza la entrevista

6. Tras las explicaciones indicadas, ¿la persona accede a realizar la entrevista?
   - Si
   - No

Filtro. Si….Pasa a pregunta 8 y se realiza la entrevista

No…Pasa a pregunta 7 y finaliza la entrevista

7. Por favor, indica las razones por las que la persona rechaza realizar la entrevista
- No quiere/no le interesa
- No tiene tiempo para hacer la encuesta
- Desconfía y no quiere participar
- Es muy mayor y no quiere
- Persona Fallecida
- Residencia Cambio de hogar y no facilitan otro teléfono
- El teléfono no corresponde con la persona
- Se encuentra enferma (en casa)
- Se encuentra enferma ingresada en el hospital
- No contesta
- Otros motivos

8. Canal mediante el que se realiza la entrevista
- Entrevista por teléfono
- Entrevista mediante videoconferencia
- Entrevista presencial
- Otro:

9. Provincia
- Almería
- Cádiz
- Córdoba
- Granada
- Huelva
- Jaén
- Málaga
- Sevilla

10. Centro Guadalinfo del que la persona es usuaria
(Selección de centro según la provincia indicada en la pregunta anterior)

Bloque I. Características del hogar y la vivienda

P.I.1. ¿Actualmente se encuentra en su vivienda habitual o reside en una vivienda distinta?
1. Habitual
2. Segunda residencia
3. Otras:

P.I.2. La vivienda en la que se encuentra actualmente es una:
1. Vivienda unifamiliar independiente, adosada o pareada (casa baja en una calle, chalet,etc)
2. Piso o apartamento en un edificio con menos de 10 viviendas
3. Piso o apartamento en un edificio con 10 o más viviendas
4. Otro tipo de vivienda (Ej: Vivienda situada en un edificio destinado principalmente a otros fines barraca, cabaña, chabola, etc.) Especifique.
5. NS/NC

P.I.3. ¿Cuál es la superficie útil de la vivienda en la que se encuentra actualmente?
1. Menos de 46 m^2 útiles
2. Entre 46 y 60

3. Entre 61 y 75
4. Entre 76 y 90
5. Entre 91 y 120
6. Más de 120
7. No sabe / No contesta

P.I.4. A efectos de caracterizar su situación de convivencia, ¿me podría decir cuál de las siguientes alternativas lo refleja mejor?

1. Hogar unipersonal (vive solo/a) ・Pasar a P. I. 6
2. Vive con su pareja
3. Vive con pareja e hijos
4. Vive con hijos/nietos sin pareja
5. Residencia/Otra institución
6. Vive con padre/madre
7. Otro tipo de hogar
8. No responde

P.I.5. Para conocer las condiciones en las que está actualmente me podría decir ¿qué número de personas residen actualmente incluyéndose usted?
Número de personas: |_|_|

P.I.5.a. ¿De estas personas cuántas son mayores de 60 años?...... |_|_|
P.I.5.b. ¿Y cuántas son menores de 16 años?... |_|_|
P.I.5.c. ¿De las personas menores de 16, cuántas son menores de 6 años? I_I_I

P.6. ¿Cuántas habitaciones tiene su vivienda? (no contar la cocina, los baños/lavabos o el comedor/sala de estar).
Número de habitaciones: |_|_|

P.I.7. Indique si en su hogar viven personas que presenten alguna limitación, discapacidad de tipo físico, sensorial o psíquico o mental o bien personas que requieran algún tipo de dedicación o cuidados especiales (ejemplo: niños/as, mayores dependientes etc)

- Si ・ Pasar a P. I. 8
- No ・ Pasar a P. I. 9

P.I. 8. Indique el número de personas (<16; 16-59; >=60) que presentan alguna de las condiciones que voy a leerle…

P.I.8..a. Tener alguna limitación, discapacidad de tipo físico, sensorial o psíquico o mental

1. SI, de que tramo de edad:
   - <16 |_|_|
   - 16-59 |_|_|
   - >=60 |_|_|

P.I.8.b. Requiere algún tipo de dedicación o de cuidados especiales

1. SI, de que tramo de edad:
   - <16 |_|_|
   - 16-59 |_|_|
   - >=60 |_|_|

P.I.9. ¿Realiza usted las tareas principales de cuidado especial a algún miembro de su familia?

- Si
- No
- No sabe/No contesta
- Otro:

P.I.10. ¿Requiere usted algún tipo de cuidado o dedicación especial?

- Si
- No
- No sabe/No contesta

P.I.11 En relación con el equipamiento, en su vivienda actual ¿Dispone en su hogar de algún tipo de dispositivo de conexión a Internet?, (Teléfono móvil, ordenador, portátil, tableta...)

- Si • Pasar a P. I. 12
- No • Pasar a P. I. 13

P.I.12 Indique el tipo de dispositivos, (señalar todos los que corresponda).

- Teléfono móvil
- Ordenador/portátil
- Tableta
- Otro:

P.I.13. ¿Dispone en su vivienda actual de acceso Internet/Wifi (a través de cualquier dispositivo fijo o móvil, se incluye teléfono móvil)?

- Sí, con datos de teléfono móvil
- Sí, con Wifi
- Sí, con datos de telefóno móvil y Wifi
- No
- No sabe/No contesta
- Otro:

Bloque II. Salud y bienestar emocional

Las siguientes preguntas se refieren a su estado de salud actual, o del periodo de referencia que le indicaremos en cada momento

P.II.14. En primer lugar, nos gustaría conocer si ¿tiene o ha tenido diagnóstico de coronavirus?

1. Sí • pasar a P.II.14.a
2. No
3. Estoy a la espera del resultado
4. No sabe
5. No contesta

Filtro si 1 en P.II.14

P.II.14.a. ¿Cómo de grave ha sido o es su proceso con la enfermedad?

1. Muy leve
2. Leve
3. Grave
4. Muy grave
5. Sin respuesta

P.II.15 ¿Le han hecho algún tipo de prueba para saber si estaba infectado/a por coronavirus? (Toma de muestras en nariz, sangre etc)

- Sí ・pasar a P.II.15.a
- No

P.II. 15.a. Por favor indique cual o cuales de las siguientes pruebas se le han realizado
Respuesta múltiple. Consultar una a una y señalar si la respuesta es afirmativa

1. Muestra de la garganta o la nariz
2. Muestra de sangre en el dedo
3. Muestra de sangre en vena

P.II.16. ¿Hay en su entorno alguien que tiene o ha tenido algún caso diagnosticado de infección por coronavirus? Respuesta múltiple

1. Persona/s de mi familia con la que resido en el hogar
2. Persona/s con la que resido en el hogar que no es familiar
3. Familiar/es que no conviven conmigo
4. Amigos, compañeros de trabajo/estudios, vecinos con lo que no resido en el hogar
5. Nadie de mi entorno
6. NC

Hablemos ahora de forma general de cómo se encuentra…

Autopercepción de salud

P.II.17. En la actualidad, en general diría que su salud es...

1. Excelente
2. Muy buena
3. Buena
4. Regular
5. Mala
6. No sabe/No está seguro.
7. No contesta.

P.II.18. En la actualidad, en general diría que su salud mental es...

1. Excelente
2. Muy buena
3. Buena
4. Regular
5. Mala
6. No sabe/No está seguro.
7. No contesta.

Bienestar emocional

P.II.19. A continuación, le voy a leer una lista de sentimientos o comportamientos que quizás haya tenido Vd. durante la última semana. Por favor, dígame con qué frecuencia, se ha sentido...

1. En ningún momento o en casi ningún momento
2. En algún momento,
3. Buena parte del tiempo,

4. Todo o casi todo el tiempo
5. No sabe

1. Deprimido/a
2. Feliz
3. Solo/a
4. Ha tenido la sensación de disfrutar de la vida
5. Rebosante de energía
6. Tranquilo/a y relajado/a

P.II. 20. Si toma también como referencia la última semana, dígame Ud. su grado de acuerdo o desacuerdo con las siguientes frases.

1. Muy de acuerdo
2. De acuerdo
3. Ni de acuerdo ni en desacuerdo
4. En desacuerdo
5. Muy en desacuerdo
6. No sabe

1. He sido optimista respecto a mi futuro
2. Por lo general me he sentido bien conmigo mismo

Felicidad y apoyo social y afectivo

P.II. 21. En términos generales, ¿en qué medida se considera Ud. una persona feliz o infeliz? Por favor, use una escala de 0 a 10, en la que 0 significa que se considera "completamente infeliz" y 10 que se considera "completamente feliz".

0 1 2 3 4 5 6 7 8 9 10 Ns/Nc 99

P.II.23. En la siguiente lista se muestran algunas cosas que otras personas hacen por nosotros o nos proporcionan. Elija para cada una la respuesta que mejor refleje su situación:

1. Tanto como deseo
2. Casi como deseo
3. Ni mucho ni poco
4. Menos de lo que deseo
5. Mucho menos de lo que deseo

P.23.1. Recibo visitas/llamadas de mis amigos y familiares
P.23.2. Recibo ayuda en asuntos relacionados con mi casa
P.23.3. Recibo elogios y reconocimientos cuando hago bien mi trabajo
P.23.4. Cuento con personas que se preocupan de lo que me sucede
P.23.5. Recibo amor y afecto
P.23.6. Tengo la posibilidad de hablar con alguien de mis problemas en el trabajo y/o en la casa
P.23.7. Tengo la posibilidad de hablar con alguien de mis problemas personales y familiares
P.23.8. Tengo posibilidad de hablar con alguien de mis problemas económicos
P.23.9. Recibo invitaciones para distraerme y salir con otras personas
P.23.10. Recibo consejos útiles cuando me ocurre algún acontecimiento importante en mi vida
P.23.11. Recibo ayuda cuando estoy enfermo/a en la cama

Molestias y enfermedades crónicas

P.II.24. Durante el último mes, ¿hasta qué punto ha notado Vd. alguna de las siguientes molestias? Utilice una escala del 1 al 5 en la que 1 es Nunca he sentido esa molestia y 5 Con mucha frecuencia.

1. Problemas de sueño
2. Problemas de estómago
3. Incremento de la tensión arterial
4. Mareos
5. Dolores de cabeza
6. Dolores musculares o de articulaciones
6a. Otros dolores
7. Sensación de opresión en el pecho
8. Otra molestia

P.II.25. ¿Le ha dicho un/a profesional de la salud (médico/a, enfermero/a) que usted padece actualmente alguna de las siguientes enfermedades crónicas? Si responde "SI" hacer P.25.a. ¿Esa condición le limita alguna de sus actividades? *

ENFERMEDAD CRÓNICA	P.25.			P.25.a	
	¿Le ha dicho un/a profesional de la salud si tiene esta condición? (si es 'No', saltar a la siguiente)			¿Esa condición le limita alguna de sus actividades?	
	Sí	No	No lo sabe	Sí	No
1. Diabetes/azúcar alta en sangre/azúcar en orina	☐	☐	☐	☐	☐
2. Hipertensión/presión arterial alta	☐	☐	☐	☐	☐
3. Colesterol alto	☐	☐	☐	☐	☐
4. Enfermedad crónica del pulmón / enfisema / bronquitis crónica	☐	☐	☐	☐	☐
5. Asma	☐	☐	☐	☐	☐
6. Enfermedades del corazón (Trastornos cardíacos/insufiencia cardíaca /insuficiencia cardíaca congestiva; angina de pecho; Infarto de miocardio/ataque al corazón)	☐	☐	☐	☐	☐
7. Dolor crónico (pecho; espalda, cuello, hombro, cintura (cervical/lumbar; Migrañas/Jaquecas/ Cefaleas crónicas / dolor de cabeza frecuente)	☐	☐	☐	☐	☐
8. Alergias crónicas (como rinitis, inflamación de ojos, dermatitis, alergia a los alimentos, etc), asma alérgica excluida	☐	☐	☐	☐	☐
9. Mala circulación	☐	☐	☐	☐	☐
10. Varices en las piernas	☐	☐	☐	☐	☐
11. Trastornos depresivos	☐	☐	☐	☐	☐
12. Trastornos de ansiedad	☐	☐	☐	☐	☐
13. Otra enfermedad crónica	☐	☐	☐	☐	☐

* Se consultan todos los diagnósticos seguidos y en las que se ha respondido afirmativamente se pregunta del siguiente modo:

P. II. 25. a. Ahora, le voy a ir indicando las enfermedades crónicas que me ha comunicado que tiene diagnosticadas, le pedimos que indique para cada una si le supone alguna limitación en sus actividades de la vida diaria

Medicamentos

P.II.26. ¿En qué medida se ha visto modificada la medicación que usted tomaba antes del último mes con respecto a ahora?

1. He disminuido mi medicación
2. Sigo igual, no ha afectado en nada
3. He aumentado mi medicación (incluye si antes no consumía, pero ahora sí)
4. No consumo medicación.

Filtro si 1,2 o 3 en la P.26.
P.26.a. Y En general, ha cambiado la manera en que toma sus medicamentos

1. Sí, ahora estoy cumpliendo mejor con la medicación
2. Sí, ahora estoy cumpliendo peor con la medicación
3. Sigo tomando los mismos medicamentos de la misma manera

P.II.27. (PARA TODOS). Y en las últimas 2 semanas, ¿ha utilizado algún tipo de medicamento (gotas, pastillas, inyecciones, supositorios, etc.)?

1. Sí
2. No
9. N/C

Filtro Si 1 en P.27.
P.27.a. Si ha tomado medicamentos, ¿Qué tipo de medicamento?

1. Medicinas para el catarro, gripe, garganta, bronquios
2. Medicinas para el dolor
3. Medicinas para para bajar la fiebre
4. Antibióticos
5. Tranquilizantes, sedantes
6. Pastillas para dormir
7. Medicamentos antidepresivos
8. Medicamentos para la alergia
9. Medicinas para el corazón
10. Medicinas para la tensión arterial
11. Medicamentos para la diabetes
12. Medicamentos para bajar el colesterol
13. Otros medicamentos

P.II.28. ¿Le han facilitado/ayudado las nuevas tecnologías (Whatsapp, facebook, zoom, face time) durante el último mes?

1. Si, me han ayudado
2. No me han sido de ayuda
3. No he tenido acceso a ese tipo de herramientas
4. No sabe

P.II.29. ¿Cree necesario reforzar la formación en nuevas tecnologías para las personas mayores de 50 años?

- Si
- No
- No sabe

Bloque III: Hábitos y estilos de vida

P.28. En relación al último mes, diría Vd. que...

	Más que antes	Igual que antes	Menos que antes	No tenía ese hábito	Ns/Nc
• 28.1 He realizado ejercicio	1	2	3	8	9
• 28.2 He consumido bebidas alcohólicas	1	2	3	8	9
• 28.3 He fumado	1	2	3	8	9

	Mejor	Igual	Peor	Ns/Nc
	1	2	3	9
• Mi alimentación ha sido	1	2	3	9

Mi peso ha aumentado, se ha mantenido o ha disminuido

1. Aumentado
2. Mantenido
3. Disminuido
9. Ns/Nc

Alimentación

P.29. ¿Cuántas raciones de verdura u hortalizas consume al día (las guarniciones o acompañamientos contabilizan como ½ ración)?

1. Dos o más al día (al menos una de ellas en ensalada o crudas)
2. Dos o más al día (aunque ninguna de ellas en ensalada o crudas)
3. Diariamente, aunque menos de dos al día
4. No diariamente, pero tres o más por semana
5. Menos de tres veces por semana
6. No consume
9. NS/NC

P.30. ¿Cuántas piezas de fruta (incluyendo zumo natural) consume al día?

1. Tres o más al día
2. Diariamente, aunque menos de tres veces al día
3. No diariamente, pero tres o más veces por semana
4. Menos de tres veces por semana
5. No consume
9. NS/NC

Actividad física

P.31. Durante las últimas dos semanas, ¿Cuántos días a la semana practica normalmente durante más de 30 minutos …? (incluyendo desplazamientos al trabajo o actividades similares)

Nº de días/sem

1. Caminar ……………………………………………………………………………..
2. Una actividad física ligera como gimnasia suave, juegos que requieren poco esfuerzo y similares …….………………………………………………….……….
3. Una actividad física moderada como montar en bicicleta, gimnasia, aeróbic, correr, natación ……………………………………………………………………….…
4. Entrenamiento físico deportivo como fútbol, baloncesto, ciclismo, natación de competición, judo, kárate o similares ……......................................……........

P.32. ¿Podría decirme cuánto pesa, aproximadamente, sin zapatos ni ropa?

Kilos: |_|_|_|

No sabe 998

No contesta 999

P.33.¿Y cuánto mide, aproximadamente, sin zapatos?

Cms.: |_|_|_|

No sabe 998

No contesta 999

Tabaco

P.34. Cambiando de tema, ¿podría usted decirme si fuma?

1. Sí, fuma diariamente
2. Sí fuma, pero no diariamente
3. No fuma, pero ha fumado
4. No fuma ni ha fumado nunca de manera habitual
9. No contesta

Alcohol

P.35. ¿Ha bebido usted en estas dos últimas semanas alguna bebida alcohólica?

1. Sí, diariamente • pasar a P.35.a
2. Sí, pero no diariamente. • pasar a P.35.a
3. No
9. NS/NC

P.35.a. Durante las últimas 2 semanas ¿alguna vez ha tomado más de 6/5/4* bebidas alcohólicas estándar en una misma ocasión? (Por "ocasión" entendemos tomar las bebidas en un intervalo aproximado de cuatro a seis horas)."

Especificaremos los siguientes números según el perfil del entrevistado: 6: Hombre menor de 65 años; 5: Hombre mayor de 65 años o mujer menor de 65 años; 4: Mujer mayor de 65 años

(Si no recuerda la edad de la persona entrevistada, puede volver a preguntar)

1. Sí, alguna vez
2. Sí, varias veces
3. No
8. No sabe
9. No contesta

P.36. Durante las últimas 2 semanas, ¿ha utilizado la bebida para calmar su ansiedad?

1. Totalmente cierto

2. Bastante cierto
3. Bastanto falso
4. Totalmente falso

Sueño

P.37. ¿Podría indicarme, aproximadamente, cuántas horas al día duerme habitualmente?

Horas:	|_|_|
NS/NC	99

P.37.a. Las horas que duerme, ¿le permiten descansar lo suficiente?
1. Sí
2. No
8. N/S
9. N/C

Vacunas

P.38. Pasando a otro tema, querríamos saber si se ha vacunado de la gripe en la última campaña 2019
1. Sí
2. No
3. NS/NC

P.38.a. ¿Y en la campaña de 2020?
1. Sí
2. No, pero tengo intención de vacunarme
3. No, y no tengo intención de vacunarme
4. NS/NC

Bloque IV. Efectos económicos y características sociodemográficas y socioeconómicas.

Situación laboral

Para poder clasificar las respuestas que usted nos ha dado necesitamos que nos conteste las siguientes preguntas.

P.40. ¿En qué situación de las siguientes se encuentra en la actualidad? (Si se encuentra en varias situaciones, indique solo la que considere principal).

1.Asalariado/a a tiempo completo.
2.Asalariado/a a tiempo completo en ERTE
3.Asalariado/a a tiempo parcial
4.Asalariado/a a tiempo parcial por ERTE
5.Empresario/a con asalariados/as
6.Empresario/a sin asalariados/as o trabajador/a independiente o miembro de cooperativa
7.Trabajos familiares no remunerados (ayudas familiares).
8.Parado/a
9.Estudiante
10.Jubilado/a, retirado/a o jubilado/a anticipado/a
11.Incapacitado/a permanente

12.Realizando tareas de voluntariado social
13.Dedicado/a a las labores del hogar, al cuidado de niños u otras personas. 10
14.Otra clase de inactividad económica
98.No sabe
98.No contesta

P.40.a. En su empleo principal o en su último empleo ¿cuál es/era la ocupación, profesión u oficio que desempeña/ba?

Precise lo más posible, por ejemplo: dependiente de comercio, mecánico de automóviles, tornero, peón de la construcción, operador de grúa, etc.). Entrevistador: Anote el literal y señale la opción que corresponda según la tabla de clasificación del manual. El informante debe especificar suficientemente para que se pueda codificar correctamente.
Ocupación: _____________

P.40.b. ¿Cuáles son sus estudios terminados de más alto nivel?

Anote el literal y señale la opción que corresponda. El informante debe especificar en su respuesta lo suficiente como para que se pueda codificar correctamente, por ejemplo: ha asistido menos de cinco años a la escuela, tres cursos completos de Ciencias Económicas y Empresariales, doctorado en Ciencias Sociales, etc.
Estudios terminados de más alto nivel: _______________________
Agente CATI: no leer. Clasificar en función del literal contestado

1. Analfabetos/as y estudios primarios incompletos.
2. Educación primaria.
3. Primera etapa de la Educación Secundaria y similar.
4. Segunda etapa de la Educación Secundaria y similar
5. Bachillerato y FP de Grado Medio.
6. Educación postsecundaria no superior.
7. Formación Profesional de Grado Superior y títulos propios De universidades de duración igual o superior a 2 años.
8. Diplomados/as universitarios/as, títulos propios universitarios de Experto o especialista y similares (Grados universitarios de 240 créditos ECTS, Bolonia).
9. Licenciados, másteres y especialidades en Ciencias de la Salud por el sistema de residencia y similares. Grados universitarios de más 240 créditos ECTS (Bolonia).
10. Título de Doctorado.
11. No se puede codificar.

Filtro: P40=1,2,3,4
P.41. ¿Qué tipo de contrato tiene en la actualidad?

1.Contrato indefinido
2.Contrato temporal.

Para trabajadores: Filtro: P40=1,2,3,4,5,6,7
P.42. Respecto a la situación en la que desarrolla su trabajo ¿cómo es actualmente?

1. Trabajo desde casa
2. Trabajo fuera de casa
3. Trabajo desde casa y desde fuera de casa
4. Estoy de permiso para ocuparme de mis hijos
5. Estoy permiso para ocuparme de otros familiares

6. Estoy de vacaciones anuales retribuidas
7. Estoy de permiso no retribuido
8. Estoy de baja laboral
9. Otra situación

Para trabajadores Filtro: P40=1,2,3,4,5,6,7

P.43. Asumiendo que su capacidad laboral en su mejor estado de salud tuviera un valor de 10 puntos, ¿cuántos puntos daría a su capacidad laboral actual? 0 (totalmente incapacitado) …10 (muy buena capacidad laboral)

0 1 2 3 4 5 6 7 8 9 10   Ns/Nc 99

Filtro: P40=8

P.44. Me ha dicho que está Vd. en situación de desempleo. ¿En cuál de las siguientes situaciones se encuentra actualmente?

1. Estoy desempleado por un ERTE debido al coronavirus.
2. Estoy desempleado por un despido debido al coronavirus (sin ERTE).
3. He tenido que cerrar mi negocio.
4. He tenido que cesar la actividad que me aportaba ingresos
5. Tenía opciones de comenzar en un trabajo, pero se han pospuesto/cancelado debido al estado de alarma
6. Mi situación de desempleo es previa al estado de alarma y no se ha visto alterada
7. Otras situaciones:

Identificación de la persona de referencia del hogar (la que aporta más ingresos)

P.46. Actualmente, ¿En su hogar quién es la persona que aporta más ingresos?

1. Yo (ir a P.50.)
2. Otra persona (ir a P.47)
3. Otra persona y yo a partes iguales (ir a P.47)

P.47. Y esa persona es...

1. Su cónyuge/pareja
2. Su padre
3. Su madre
4. Su hijo
5. Su hija
6. Su nieto
7. Su nieta
8. Su nuera
9. Su yerno
10. No contesta

P.48. ¿Cuáles son los estudios terminados de más alto nivel de la persona que aporta más ingresos en el hogar?

1. Analfabetos/as y estudios primarios incompletos.
2. Educación primaria.
3. Primera etapa de la Educación Secundaria y similar.
4. Segunda etapa de la Educación Secundaria y similar
5. Bachillerato y FP de Grado Medio.
6. Educación postsecundaria no superior.

7. Formación Profesional de Grado Superior y títulos propios De universidades de duración igual o superior a 2 años.
8. Diplomados/as universitarios/as, títulos propios universitarios de Experto o especialista y similares (Grados universitarios de 240 créditos ECTS, Bolonia).
9. Licenciados, másteres y especialidades en Ciencias de la Salud por el sistema de residencia y similares. Grados universitarios de más 240 créditos ECTS (Bolonia).
10. Título de Doctorado.
11. No se puede codificar.

P.49. ¿En qué situación de las siguientes se encuentra en la actualidad la persona que aporta más ingresos al hogar? (Si se encuentra en varias situaciones, indique solo la que considere principal).

1. Asalariado/a a tiempo completo
2. Asalariado/a a tiempo completo en ERTE
3. Asalariado/a a tiempo parcial
4. Asalariado/a a tiempo parcial por ERTE
5. Empresario/a con asalariados/as
6. Empresario/a sin asalariados/as o trabajador/a independiente o miembro de cooperativa
7. Trabajos familiares no remunerados (ayudas familiares).
8. Parado/a
9. Estudiante
10. Jubilado/a, retirado/a o jubilado/a anticipado/a
11. Incapacitado/a permanente
12. Realizando tareas de voluntariado social
13. Dedicado/a a las labores del hogar, al cuidado de niños u otras personas.
14. Otra clase de inactividad económica
98. No sabe
99. No contesta

P.49.a. En su empleo principal o en su último empleo ¿cuál es/era la ocupación, profesión u oficio que desempeña/ba la persona que aporta más ingresos al hogar?

Precise lo más posible, por ejemplo: dependiente de comercio, mecánico de automóviles, tornero, peón de la construcción, operador de grúa, etc.). Entrevistador: Anote el literal y señale la opción que corresponda según la tabla de clasificación del manual. El informante debe especificar suficientemente para que se pueda codificar correctamente
Ocupación: ___________________

Ingresos y situación económica del hogar

P.50. Un hogar puede tener diferentes fuentes de ingresos y más de un miembro del hogar puede contribuir con sus ingresos. En relación con el total de ingresos de su hogar, ¿cómo suelen llegar a fin de mes?

1. Con mucha facilidad.
2. Con facilidad.
3. Con cierta facilidad.
4. Con cierta dificultad.
5. Con dificultad
6. Con mucha dificultad
7. No sabe.
8. No contesta.

P.51. Desde que comenzó la crisis sanitaria, ¿ha tenido en su hogar algún retraso en el pago de recibos como la hipoteca, el alquiler, o suministros como la luz, el agua, impuestos, etc.? (Respuesta múltiple).

1. Hipoteca
2. Alquiler
3. Cuotas de compras aplazadas
4. Cuotas de seguros
5. Impuestos (IBI, los relacionados con vehículos, etc.)
6. Pagos de recibos de agua, gas, calefacción, electricidad, comunidad
7. Tasa de autónomo
8. Pago de colegios
9. Actividades extraescolares
10. Otros

P.51 a ¿Se ha resuelto? Filtro si en p51=si

1. Sí
2. No

P.53. Dígame en qué intervalo, de los que le leeré a continuación, se encuentran los ingresos mensuales netos del conjunto de miembros de su hogar EN EL MES ANTERIOR

1. Sin ingresos (no leer)
2. Menos de 450 euros
3. De 451 a 900 euros
4. De 901 a 1.600 euros
5. De 1.601 a 2.500 euros
6. De 2.501 a 3.000 euros
7. Más de 3.000 euros
98. NS
98. NC

P52 Ya para terminar… sobre la preocupación en el futuro
¿Hasta qué punto le preocupa que a usted o a su familia le afecte directamente en el futuro alguna de las siguientes situaciones que le voy a nombrar?

1. Mucho
2. Bastante
3. Algo
4. Poco
5. Nada
6. Ya me OCURRE/ o me ha ocurrido
7. No sabe/No contesta
8. No procede

1. Contagiarse por coronavirus
2. Crisis de abastecimiento (luz, agua, telefonía, alimentos, medicamentos)
3. Pérdida de ingresos
4. Desempleo
5. Problemas con los estudios
6. Revueltas, Inseguridad
7. No recibir la atención sanitaria que necesite
8. Problemas con el cobro de las pensiones

Para el entrevistador/a
Por favor, si hay alguna incidencia en la entrevista o tiene alguna observación que realizar, indíquelo aquí
Observaciones de la entrevista: ____

Esto es todo, muchas gracias por su colaboración y por el tiempo que nos ha dedicado respondiendo esta encuesta. Le recuerdo que nos tiene a su disposición en su centro Guadalinfo

Medición 2 – ESSOC+55. Cuestionario muestra longitudinal

Este cuestionario está basado en de la muestra nueva de la medición 2 de la ESSOC+55, filtrando las variables de las que ya se tiene el resultado en la anterior medición siempre que no haya habido modificaciones en la situación actual respecto de la anterior entrevista. La información se organiza en 5 grandes bloques igual que en el resto de mediciones.

Medición 2 – ESSOC+55. Cuestionario muestra nueva

Este cuestionario es igual al de la medición 1 de la ESSOC+55, aunque puede sufrir alguna modificación. La información se organiza en 5 grandes bloques:

- BLOQUE INICIAL. INFORMACIÓN DE LA PERSONA ENTREVISTADA Y DEL AGENTE GUADALINFO
- BLOQUE I. CARACTERÍSTICAS DEL HOGAR Y LA VIVIENDA
- BLOQUE II. SALUD, BIENESTAR EMOCIONAL
- BLOQUE III: HÁBITOS Y ESTILOS DE VIDA
- BLOQUE IV. SITUACIÓN ECONÓMICA Y CARACTERÍSTICAS SOCIODEMOGRÁFICAS.

Datos de la persona entrevistada y Agente Guadalinfo

1. Sexo de la persona entrevistada
   - Femenino
   - Masculino

2. Edad de la persona entrevistada
(Si no contesta a la pregunta poner 999)
(El entrevistador dispone de los datos de edad y sexo previamente a la entrevista, ya que el muestreo se ha realizado por grupo de edad y sexo)
Cifra mínima: 49

3. Código Persona entrevistada

4. Tipo de Centro Guadalinfo.
   - Centro Guadalinfo
   - Centros CAPI
   - Centro CAE

- Centros ELA
- Telecentros
- Otro:

5. Nombre del Agente Guadalinfo que realiza la entrevista

6. Tras las explicaciones indicadas, ¿la persona accede a realizar la entrevista?
   - Si
   - No

Filtro. Si….Pasa a pregunta 8 y se realiza la entrevista
No…Pasa a pregunta 7 y finaliza la entrevista

7. Por favor, indica las razones por las que la persona rechaza realizar la entrevista
   - No quiere/no le interesa
   - No tiene tiempo para hacer la encuesta
   - Desconfía y no quiere participar
   - Es muy mayor y no quiere
   - Persona Fallecida
   - Residencia Cambio de hogar y no facilitan otro teléfono
   - El teléfono no corresponde con la persona
   - Se encuentra enferma (en casa)
   - Se encuentra enferma ingresada en el hospital
   - No contesta
   - Otros motivos

8. Canal mediante el que se realiza la entrevista
   - Entrevista por teléfono
   - Entrevista mediante videoconferencia
   - Entrevista presencial
   - Otro:
9. Provincia
   - Almería
   - Cádiz
   - Córdoba
   - Granada
   - Huelva
   - Jaén
   - Málaga
   - Sevilla
10. Centro Guadalinfo del que la persona es usuaria
(Selección de centro según la provincia indicada en la pregunta anterior)

Bloque I. Características del hogar y la vivienda

P.I.1. ¿Actualmente se encuentra en su vivienda habitual o reside en una vivienda distinta?

1. Habitual
2. Segunda residencia
3. Otras:

P.I.2. La vivienda en la que se encuentra actualmente es una:

1. Vivienda unifamiliar independiente, adosada o pareada (casa baja en una calle, chalet,etc)
2. Piso o apartamento en un edificio con menos de 10 viviendas
3. Piso o apartamento en un edificio con 10 o más viviendas
4. Otro tipo de vivienda (Ej: Vivienda situada en un edificio destinado principalmente a otros fines barraca, cabaña, chabola, etc.) Especifique.
5. NS/NC

P.I.3. ¿Cuál es la superficie útil de la vivienda en la que se encuentra actualmente?

1. Menos de 46 m² útiles
2. Entre 46 y 60
3. Entre 61 y 75
4. Entre 76 y 90
5. Entre 91 y 120
6. Más de 120
7. No sabe / No contesta

P.I.4. A efectos de caracterizar su situación de convivencia, ¿me podría decir cuál de las siguientes alternativas lo refleja mejor?

1. Hogar unipersonal (vive solo/a) ・Pasar a P. I. 6
2. Vive con su pareja
3. Vive con pareja e hijos
4. Vive con hijos/nietos sin pareja
5. Residencia/Otra institución
6. Vive con padre/madre
7. Otro tipo de hogar
8. No responde

P.I.5. Para conocer las condiciones en las que está actualmente me podría decir ¿qué número de personas residen actualmente incluyéndose usted?
Número de personas: |_|_|

P.I.5.a. ¿De estas personas cuántas son mayores de 60 años?...... |_|_|
P.I.5.b. ¿Y cuántas son menores de 16 años?... |_|_|
P.I.5.c. ¿De las personas menores de 16, cuántas son menores de 6 años? I_I_I

P.6. ¿Cuántas habitaciones tiene su vivienda? (no contar la cocina, los baños/lavabos o el comedor/sala de estar).
Número de habitaciones: |_|_|

P.I.7. Indique si en su hogar viven personas que presenten alguna limitación, discapacidad de tipo físico, sensorial o psíquico o mental o bien personas que requieran algún tipo de dedicación o cuidados especiales (ejemplo: niños/as, mayores dependientes etc)

- Si ・ Pasar a P. I. 8
- No ・ Pasar a P. I. 9

P.I. 8. Indique el número de personas (<16; 16-59; >=60) que presentan alguna de las condiciones que voy a leerle…

P.I.8..a. Tener alguna limitación, discapacidad de tipo físico, sensorial o psíquico o mental

1. SI, de que tramo de edad:
    - <16 |_|_|
    - 16-59 |_|_|
    - >=60 |_|_|

P.I.8.b. Requiere algún tipo de dedicación o de cuidados especiales

1. SI, de que tramo de edad:
    - <16 |_|_|
    - 16-59 |_|_|
    - >=60 |_|_|

P.I.9. ¿Realiza usted las tareas principales de cuidado especial a algún miembro de su familia?

- Si
- No
- No sabe/No contesta
- Otro:

P.I.10. ¿Requiere usted algún tipo de cuidado o dedicación especial?

- Si
- No
- No sabe/No contesta

P.I.11 En relación con el equipamiento, en su vivienda actual ¿Dispone en su hogar de algún tipo de dispositivo de conexión a Internet?, (Teléfono móvil, ordenador, portátil, tableta...)

- Si ・ Pasar a P. I. 12
- No ・ Pasar a P. I. 13

P.I.12 Indique el tipo de dispositivos, (señalar todos los que corresponda).

- Teléfono móvil
- Ordenador/portátil
- Tableta
- Otro:

P.I.13. ¿Dispone en su vivienda actual de acceso Internet/Wifi (a través de cualquier dispositivo fijo o móvil, se incluye teléfono móvil)?

- Sí, con datos de teléfono móvil
- Sí, con Wifi
- Sí, con datos de telefóno móvil y Wifi
- No
- No sabe/No contesta
- Otro:

Bloque II. Salud y bienestar emocional

Las siguientes preguntas se refieren a su estado de salud actual, o del periodo de referencia que le indicaremos en cada momento

P.II.14. En primer lugar, nos gustaría conocer si ¿tiene o ha tenido diagnóstico de coronavirus?

1. Sí ・pasar a P.II.14.a
2. No

3. Estoy a la espera del resultado
4. No sabe
5. No contesta

Filtro si 1 en P.II.14

P.II.14.a. ¿Cómo de grave ha sido o es su proceso con la enfermedad?

1. Muy leve
2. Leve
3. Grave
4. Muy grave
5. Sin respuesta

P.II.15 ¿Le han hecho algún tipo de prueba para saber si estaba infectado/a por coronavirus? (Toma de muestras en nariz, sangre etc)

- Sí ・pasar a P.II.15.a
- No

P.II. 15.a. Por favor indique cual o cuales de las siguientes pruebas se le han realizado

Respuesta múltiple. Consultar una a una y señalar si la respuesta es afirmativa

1. Muestra de la garganta o la nariz
2. Muestra de sangre en el dedo
3. Muestra de sangre en vena

P.II.16. ¿Hay en su entorno alguien que tiene o ha tenido algún caso diagnosticado de infección por coronavirus? Respuesta múltiple

1. Persona/s de mi familia con la que resido en el hogar
2. Persona/s con la que resido en el hogar que no es familiar
3. Familiar/es que no conviven conmigo
4. Amigos, compañeros de trabajo/estudios, vecinos con lo que no resido en el hogar
5. Nadie de mi entorno
6. NC

Hablemos ahora de forma general de cómo se encuentra…

Autopercepción de salud

P.II.17. En la actualidad, en general diría que su salud es...

1. Excelente
2. Muy buena
3. Buena
4. Regular
5. Mala
6. No sabe/No está seguro.
7. No contesta.

P.II.18. En la actualidad, en general diría que su salud mental es...

1. Excelente
2. Muy buena
3. Buena
4. Regular

5. Mala
6. No sabe/No está seguro.
7. No contesta.

Bienestar emocional

P.II.19. A continuación, le voy a leer una lista de sentimientos o comportamientos que quizás haya tenido Vd. durante la última semana. Por favor, dígame con qué frecuencia, se ha sentido...

1. En ningún momento o en casi ningún momento
2. En algún momento,
3. Buena parte del tiempo,
4. Todo o casi todo el tiempo
5. No sabe

1. Deprimido/a
2. Feliz
3. Solo/a
4. Ha tenido la sensación de disfrutar de la vida
5. Rebosante de energía
6. Tranquilo/a y relajado/a

P.II. 20. Si toma también como referencia la última semana, dígame Ud. su grado de acuerdo o desacuerdo con las siguientes frases.

1. Muy de acuerdo
2. De acuerdo
3. Ni de acuerdo ni en desacuerdo
4. En desacuerdo
5. Muy en desacuerdo
6. No sabe

1. He sido optimista respecto a mi futuro
2. Por lo general me he sentido bien conmigo mismo

Felicidad y apoyo social y afectivo

P.II. 21. En términos generales, ¿en qué medida se considera Ud. una persona feliz o infeliz? Por favor, use una escala de 0 a 10, en la que 0 significa que se considera "completamente infeliz" y 10 que se considera "completamente feliz".

0 1 2 3 4 5 6 7 8 9 10 Ns/Nc 99

P.II.23. En la siguiente lista se muestran algunas cosas que otras personas hacen por nosotros o nos proporcionan. Elija para cada una la respuesta que mejor refleje su situación:

1. Tanto como deseo
2. Casi como deseo
3. Ni mucho ni poco
4. Menos de lo que deseo
5. Mucho menos de lo que deseo

P.23.1. Recibo visitas/llamadas de mis amigos y familiares

P.23.2. Recibo ayuda en asuntos relacionados con mi casa
P.23.3. Recibo elogios y reconocimientos cuando hago bien mi trabajo
P.23.4. Cuento con personas que se preocupan de lo que me sucede
P.23.5. Recibo amor y afecto
P.23.6. Tengo la posibilidad de hablar con alguien de mis problemas en el trabajo y/o en la casa
P.23.7. Tengo la posibilidad de hablar con alguien de mis problemas personales y familiares
P.23.8. Tengo posibilidad de hablar con alguien de mis problemas económicos
P.23.9. Recibo invitaciones para distraerme y salir con otras personas
P.23.10. Recibo consejos útiles cuando me ocurre algún acontecimiento importante en mi vida
P.23.11. Recibo ayuda cuando estoy enfermo/a en la cama

Molestias y enfermedades crónicas

P.II.24. Durante el último mes, ¿hasta qué punto ha notado Vd. alguna de las siguientes molestias? Utilice una escala del 1 al 5 en la que 1 es Nunca he sentido esa molestia y 5 Con mucha frecuencia.

1. Problemas de sueño
2. Problemas de estómago
3. Incremento de la tensión arterial
4. Mareos
5. Dolores de cabeza
6. Dolores musculares o de articulaciones
6a. Otros dolores
7. Sensación de opresión en el pecho
8. Otra molestia

P.II.25. ¿Le ha dicho un/a profesional de la salud (médico/a, enfermero/a) que usted padece actualmente alguna de las siguientes enfermedades crónicas? Si responde "SI" hacer P.25.a. ¿Esa condición le limita alguna de sus actividades? *

ENFERMEDAD CRÓNICA	P.25.			P.25.a	
	¿Le ha dicho un/a profesional de la salud si tiene esta condición? (si es 'No', saltar a la siguiente)			¿Esa condición le limita alguna de sus actividades?	
	Sí	No	No lo sabe	Sí	No
14. Diabetes/azúcar alta en sangre/azúcar en orina	☐	☐	☐	☐	☐
15. Hipertensión/presión arterial alta	☐	☐	☐	☐	☐
16. Colesterol alto	☐	☐	☐	☐	☐
17. Enfermedad crónica del pulmón / enfisema / bronquitis crónica	☐	☐	☐	☐	☐
18. Asma	☐	☐	☐	☐	☐
19. Enfermedades del corazón (Trastornos cardíacos/insufiencia cardíaca /insuficiencia cardíaca congestiva; angina de pecho; Infarto de miocardio/ataque al corazón)	☐	☐	☐	☐	☐

20. Dolor crónico (pecho; espalda, cuello, hombro, cintura (cervical/lumbar; Migrañas/Jaquecas/ Cefaleas crónicas / dolor de cabeza frecuente)	☐	☐	☐	☐	☐
21. Alergias crónicas (como rinitis, inflamación de ojos, dermatitis, alergia a los alimentos, etc), asma alérgica excluida	☐	☐	☐	☐	☐
22. Mala circulación	☐	☐	☐	☐	☐
23. Varices en las piernas	☐	☐	☐	☐	☐
24. Trastornos depresivos	☐	☐	☐	☐	☐
25. Trastornos de ansiedad	☐	☐	☐	☐	☐
26. Otra enfermedad crónica	☐	☐	☐	☐	☐

* Se consultan todos los diagnósticos seguidos y en las que se ha respondido afirmativamente se pregunta del siguiente modo:
P. II. 25. a. Ahora, le voy a ir indicando las enfermedades crónicas que me ha comunicado que tiene diagnosticadas, le pedimos que indique para cada una si le supone alguna limitación en sus actividades de la vida diaria

<u>Medicamentos</u>

P.II.26. ¿En qué medida se ha visto modificada la medicación que usted tomaba antes del último mes con respecto a ahora?

1. He disminuido mi medicación
2. Sigo igual, no ha afectado en nada
3. He aumentado mi medicación (incluye si antes no consumía, pero ahora sí)
4. No consumo medicación.

Filtro si 1,2 o 3 en la P.26.
P.26.a. Y En general, ha cambiado la manera en que toma sus medicamentos

1. Sí, ahora estoy cumpliendo mejor con la medicación
2. Sí, ahora estoy cumpliendo peor con la medicación
3. Sigo tomando los mismos medicamentos de la misma manera

P.II.27. (PARA TODOS). Y en las últimas 2 semanas, ¿ha utilizado algún tipo de medicamento (gotas, pastillas, inyecciones, supositorios, etc.)?

1. Sí
2. No
9. N/C

Filtro Si 1 en P.27.
P.27.a. Si ha tomado medicamentos, ¿Qué tipo de medicamento?

1. Medicinas para el catarro, gripe, garganta, bronquios
2. Medicinas para el dolor
3. Medicinas para para bajar la fiebre
4. Antibióticos
5. Tranquilizantes, sedantes
6. Pastillas para dormir
7. Medicamentos antidepresivos
8. Medicamentos para la alergia
9. Medicinas para el corazón

10. Medicinas para la tensión arterial
11. Medicamentos para la diabetes
12. Medicamentos para bajar el colesterol
13. Otros medicamentos

P.II.28. ¿Le han facilitado/ayudado las nuevas tecnologías (Whatsapp, facebook, zoom, face time) durante el último mes?

1. Si, me han ayudado
2. No me han sido de ayuda
3. No he tenido acceso a ese tipo de herramientas
4. No sabe

P.II.29. ¿Cree necesario reforzar la formación en nuevas tecnologías para las personas mayores de 50 años?

- Si
- No
- No sabe

Bloque III: Hábitos y estilos de vida

P.28. En relación al último mes, diría Vd. que...

	Más que antes	Igual que antes	Menos que antes	No tenía ese hábito	Ns/Nc
• 28.1 He realizado ejercicio	1	2	3	8	9
• 28.2 He consumido bebidas alcohólicas	1	2	3	8	9
• 28.3 He fumado	1	2	3	8	9

	Mejor	Igual	Peor	Ns/Nc
	1	2	3	9
• Mi alimentación ha sido	1	2	3	9

Mi peso ha aumentado, se ha mantenido o ha disminuido

1. Aumentado
2. Mantenido
3. Disminuido
9. Ns/Nc

Alimentación

P.29. ¿Cuántas raciones de verdura u hortalizas consume al día (las guarniciones o acompañamientos contabilizan como ½ ración)?

1. Dos o más al día (al menos una de ellas en ensalada o crudas)
2. Dos o más al día (aunque ninguna de ellas en ensalada o crudas)
3. Diariamente, aunque menos de dos al día
4. No diariamente, pero tres o más por semana
5. Menos de tres veces por semana
6. No consume

9. NS/NC

P.30. ¿Cuántas piezas de fruta (incluyendo zumo natural) consume al día?

1. Tres o más al día
2. Diariamente, aunque menos de tres veces al día
3. No diariamente, pero tres o más veces por semana
4. Menos de tres veces por semana
5. No consume
9. NS/NC

Actividad física

P.31. Durante las últimas dos semanas, ¿Cuántos días a la semana practica normalmente durante más de 30 minutos …? (incluyendo desplazamientos al trabajo o actividades similares)

Nº de días/sem

1. Caminar ……………………………………………………………………………..
2. Una actividad física ligera como gimnasia suave, juegos que requieren poco esfuerzo y similares …….………………………………………………….……….
3. Una actividad física moderada como montar en bicicleta, gimnasia, aeróbic, correr, natación …………………………………………………………………….…
4. Entrenamiento físico deportivo como fútbol, baloncesto, ciclismo, natación de competición, judo, kárate o similares ……..……........

P.32. ¿Podría decirme cuánto pesa, aproximadamente, sin zapatos ni ropa?

Kilos: |_|_|_|

No sabe 998

No contesta 999

P.33.¿Y cuánto mide, aproximadamente, sin zapatos?

Cms.: |_|_|_|

No sabe 998

No contesta 999

Tabaco

P.34. Cambiando de tema, ¿podría usted decirme si fuma?

1. Sí, fuma diariamente
2. Sí fuma, pero no diariamente
3. No fuma, pero ha fumado
4. No fuma ni ha fumado nunca de manera habitual
9. No contesta

Alcohol

P.35. ¿Ha bebido usted en estas dos últimas semanas alguna bebida alcohólica?

1. Sí, diariamente • pasar a P.35.a
2. Sí, pero no diariamente. • pasar a P.35.a
3. No
9. NS/NC

P.35.a. Durante las últimas 2 semanas ¿alguna vez ha tomado más de 6/5/4* bebidas alcohólicas estándar en una misma ocasión? (Por "ocasión" entendemos tomar las bebidas en un intervalo aproximado de cuatro a seis horas)."
Especificaremos los siguientes números según el perfil del entrevistado: 6: Hombre menor de 65 años; 5: Hombre mayor de 65 años o mujer menor de 65 años; 4: Mujer mayor de 65 años
(Si no recuerda la edad de la persona entrevistada, puede volver a preguntar)

1. Sí, alguna vez
2. Sí, varias veces
3. No

8. No sabe
9. No contesta

P.36. Durante las últimas 2 semanas, ¿ha utilizado la bebida para calmar su ansiedad?

1. Totalmente cierto
2. Bastante cierto
3. Bastanto falso
4. Totalmente falso

<u>Sueño</u>

P.37. ¿Podría indicarme, aproximadamente, cuántas horas al día duerme habitualmente?

Horas:	|_|_|
NS/NC	99

P.37.a. Las horas que duerme, ¿le permiten descansar lo suficiente?

1. Sí
2. No

8. N/S
9. N/C

<u>Vacunas</u>

P.38. Pasando a otro tema, querríamos saber si se ha vacunado de la gripe en la última campaña 2019

1. Sí
2. No
3. NS/NC

P.38.a. ¿Y en la campaña de 2020?

1. Sí
2. No, pero tengo intención de vacunarme
3. No, y no tengo intención de vacunarme
4. NS/NC

Bloque IV. Efectos económicos y características sociodemográficas y socioeconómicas.

<u>Situación laboral</u>

Para poder clasificar las respuestas que usted nos ha dado necesitamos que nos conteste las siguientes preguntas.

P.40. ¿En qué situación de las siguientes se encuentra en la actualidad? (Si se encuentra en varias situaciones, indique solo la que considere principal).

1.Asalariado/a a tiempo completo.
2.Asalariado/a a tiempo completo en ERTE
3.Asalariado/a a tiempo parcial
4.Asalariado/a a tiempo parcial por ERTE
5.Empresario/a con asalariados/as
6.Empresario/a sin asalariados/as o trabajador/a independiente o miembro de cooperativa
7.Trabajos familiares no remunerados (ayudas familiares).
8.Parado/a
9.Estudiante
10.Jubilado/a, retirado/a o jubilado/a anticipado/a
11.Incapacitado/a permanente
12.Realizando tareas de voluntariado social
13.Dedicado/a a las labores del hogar, al cuidado de niños u otras personas. 10
14.Otra clase de inactividad económica
98.No sabe
98.No contesta

P.40.a. En su empleo principal o en su último empleo ¿cuál es/era la ocupación, profesión u oficio que desempeña/ba?

Precise lo más posible, por ejemplo: dependiente de comercio, mecánico de automóviles, tornero, peón de la construcción, operador de grúa, etc.). Entrevistador: Anote el literal y señale la opción que corresponda según la tabla de clasificación del manual. El informante debe especificar suficientemente para que se pueda codificar correctamente.
Ocupación: _____________

P.40.b. ¿Cuáles son sus estudios terminados de más alto nivel?

Anote el literal y señale la opción que corresponda. El informante debe especificar en su respuesta lo suficiente como para que se pueda codificar correctamente, por ejemplo: ha asistido menos de cinco años a la escuela, tres cursos completos de Ciencias Económicas y Empresariales, doctorado en Ciencias Sociales, etc.
Estudios terminados de más alto nivel: _______________________
Agente CATI: no leer. Clasificar en función del literal contestado

1. Analfabetos/as y estudios primarios incompletos.
2. Educación primaria.
3. Primera etapa de la Educación Secundaria y similar.
4. Segunda etapa de la Educación Secundaria y similar
5. Bachillerato y FP de Grado Medio.
6. Educación postsecundaria no superior.
7. Formación Profesional de Grado Superior y títulos propios De universidades de duración igual o superior a 2 años.
8. Diplomados/as universitarios/as, títulos propios universitarios de Experto o especialista y similares (Grados universitarios de 240 créditos ECTS, Bolonia).
9. Licenciados, másteres y especialidades en Ciencias de la Salud por el sistema de residencia y similares. Grados universitarios de más 240 créditos ECTS (Bolonia).
10. Título de Doctorado.

11. No se puede codificar.

Filtro: P40=1,2,3,4

P.41. ¿Qué tipo de contrato tiene en la actualidad?

1.Contrato indefinido

2.Contrato temporal.

Para trabajadores: Filtro: P40=1,2,3,4,5,6,7

P.42. Respecto a la situación en la que desarrolla su trabajo ¿cómo es actualmente?

1. Trabajo desde casa
2. Trabajo fuera de casa
3. Trabajo desde casa y desde fuera de casa
4. Estoy de permiso para ocuparme de mis hijos
5. Estoy permiso para ocuparme de otros familiares
6. Estoy de vacaciones anuales retribuidas
7. Estoy de permiso no retribuido
8. Estoy de baja laboral
9. Otra situación

Para trabajadores Filtro: P40=1,2,3,4,5,6,7

P.43. Asumiendo que su capacidad laboral en su mejor estado de salud tuviera un valor de 10 puntos, ¿cuántos puntos daría a su capacidad laboral actual? 0 (totalmente incapacitado) …10 (muy buena capacidad laboral)

0 1 2 3 4 5 6 7 8 9 10 Ns/Nc 99

Filtro: P40=8

P.44. Me ha dicho que está Vd. en situación de desempleo. ¿En cuál de las siguientes situaciones se encuentra actualmente?

1. Estoy desempleado por un ERTE debido al coronavirus.
2. Estoy desempleado por un despido debido al coronavirus (sin ERTE).
3. He tenido que cerrar mi negocio.
4. He tenido que cesar la actividad que me aportaba ingresos
5. Tenía opciones de comenzar en un trabajo, pero se han pospuesto/cancelado debido al estado de alarma
6. Mi situación de desempleo es previa al estado de alarma y no se ha visto alterada
7. Otras situaciones:

Identificación de la persona de referencia del hogar (la que aporta más ingresos)

P.46. Actualmente, ¿En su hogar quién es la persona que aporta más ingresos?

1. Yo (ir a P.50.)
2. Otra persona (ir a P.47)
3. Otra persona y yo a partes iguales (ir a P.47)

P.47. Y esa persona es...

1. Su cónyuge/pareja
2. Su padre
3. Su madre
4. Su hijo
5. Su hija

6. Su nieto
7. Su nieta
8. Su nuera
9. Su yerno
10. No contesta

P.48. ¿Cuáles son los estudios terminados de más alto nivel de la persona que aporta más ingresos en el hogar?

1. Analfabetos/as y estudios primarios incompletos.
2. Educación primaria.
3. Primera etapa de la Educación Secundaria y similar.
4. Segunda etapa de la Educación Secundaria y similar
5. Bachillerato y FP de Grado Medio.
6. Educación postsecundaria no superior.
7. Formación Profesional de Grado Superior y títulos propios De universidades de duración igual o superior a 2 años.
8. Diplomados/as universitarios/as, títulos propios universitarios de Experto o especialista y similares (Grados universitarios de 240 créditos ECTS, Bolonia).
9. Licenciados, másteres y especialidades en Ciencias de la Salud por el sistema de residencia y similares. Grados universitarios de más 240 créditos ECTS (Bolonia).
10. Título de Doctorado.
11. No se puede codificar.

P.49. ¿En qué situación de las siguientes se encuentra en la actualidad la persona que aporta más ingresos al hogar? (Si se encuentra en varias situaciones, indique solo la que considere principal).

1. Asalariado/a a tiempo completo
2. Asalariado/a a tiempo completo en ERTE
3. Asalariado/a a tiempo parcial
4. Asalariado/a a tiempo parcial por ERTE
5. Empresario/a con asalariados/as
6. Empresario/a sin asalariados/as o trabajador/a independiente o miembro de cooperativa
7. Trabajos familiares no remunerados (ayudas familiares).
8. Parado/a
9. Estudiante
10. Jubilado/a, retirado/a o jubilado/a anticipado/a
11. Incapacitado/a permanente
12. Realizando tareas de voluntariado social
13. Dedicado/a a las labores del hogar, al cuidado de niños u otras personas.
14. Otra clase de inactividad económica
98. No sabe
99. No contesta

P.49.a. En su empleo principal o en su último empleo ¿cuál es/era la ocupación, profesión u oficio que desempeña/ba la persona que aporta más ingresos al hogar?

Precise lo más posible, por ejemplo: dependiente de comercio, mecánico de automóviles, tornero, peón de la construcción, operador de grúa, etc.). Entrevistador: Anote el literal y señale la opción que corresponda según la tabla de clasificación del manual. El informante debe especificar suficientemente para que se pueda codificar correctamente
Ocupación: ___________________

Ingresos y situación económica del hogar

P.50. Un hogar puede tener diferentes fuentes de ingresos y más de un miembro del hogar puede contribuir con sus ingresos. En relación con el total de ingresos de su hogar, ¿cómo suelen llegar a fin de mes?

1. Con mucha facilidad.
2. Con facilidad.
3. Con cierta facilidad.
4. Con cierta dificultad.
5. Con dificultad
6. Con mucha dificultad
7. No sabe.
8. No contesta.

P.51. Desde que comenzó la crisis sanitaria, ¿ha tenido en su hogar algún retraso en el pago de recibos como la hipoteca, el alquiler, o suministros como la luz, el agua, impuestos, etc.? (Respuesta múltiple).

1. Hipoteca
2. Alquiler
3. Cuotas de compras aplazadas
4. Cuotas de seguros
5. Impuestos (IBI, los relacionados con vehículos, etc.)
6. Pagos de recibos de agua, gas, calefacción, electricidad, comunidad
7. Tasa de autónomo
8. Pago de colegios
9. Actividades extraescolares
10. Otros

P.51 a ¿Se ha resuelto? Filtro si en p51=si

1. Sí
2. No

P.53. Dígame en qué intervalo, de los que le leeré a continuación, se encuentran los ingresos mensuales netos del conjunto de miembros de su hogar EN EL MES ANTERIOR

1. Sin ingresos (no leer)
2. Menos de 450 euros
3. De 451 a 900 euros
4. De 901 a 1.600 euros
5. De 1.601 a 2.500 euros
6. De 2.501 a 3.000 euros
7. Más de 3.000 euros

98. NS
98. NC

P52 Ya para terminar… sobre la preocupación en el futuro
¿Hasta qué punto le preocupa que a usted o a su familia le afecte directamente en el futuro alguna de las siguientes situaciones que le voy a nombrar?

1. Mucho
2. Bastante
3. Algo

4. Poco
5. Nada
6. Ya me OCURRE/ o me ha ocurrido
7. No sabe/No contesta
8. No procede

1. Contagiarse por coronavirus
2. Crisis de abastecimiento (luz, agua, telefonía, alimentos, medicamentos)
3. Pérdida de ingresos
4. Desempleo
5. Problemas con los estudios
6. Revueltas, Inseguridad
7. No recibir la atención sanitaria que necesite
8. Problemas con el cobro de las pensiones

Para el entrevistador/a
Por favor, si hay alguna incidencia en la entrevista o tiene alguna observación que realizar, indíquelo aquí
Observaciones de la entrevista: ____

Esto es todo, muchas gracias por su colaboración y por el tiempo que nos ha dedicado respondiendo esta encuesta. Le recuerdo que nos tiene a su disposición en su centro Guadalinfo.
