# Supplementary material for: Impact of COVID-19 on the Health of the General and More Vulnerable Population and Its Determinants: Health Care and Social Survey–ESSOC, Study Protocol
Source: Int J Environ Res Public Health. 2021 Jul 31;18(15):8120. doi: 10.3390/ijerph18158120 (PMC8345631; doi:10.3390/ijerph18158120)
Supplement: Supplementary file 1 [file ijerph-18-08120-s001.zip › SupplementaryMaterial3_AuxiliaryVariablesEnglish.pdf]

Impact of COVID-19 on the health of the general and more vulnerable population and its determinants: Health care and social survey-ESSOC, study protocol

General data specification (Administrative records)

Health and Social Survey (ESSOC)

Spatiotemporal distribution and socioeconomic and environmental factors of SARS-Cov-2 infection

Junta de Andalucía
Consejería de Salud y Familias
Escuela Andaluza de Salud Pública

Table of contents

1. Introduction 3
2. Sources of Information 4
3. Cohort 6
4. Location and Characteristics of the Person 6
5. COVID – 19 7
6. Diagnostics 9
7. Other (Scales) 13
8. Risk factors 13
9. Hospitalizations 14
10. Socioeconomic Information 16
11. Environmental Information 18

1. Introduction

This document identifies the variables necessary for the development of the projects "Sanitary and Social Survey (ESSOC)" and "Spatiotemporal distribution and socioeconomic and environmental factors of infection by SARS-CoV-2".

These projects have received funding from the competitive calls of SUPERA COVID19 (SAUN: Santander Universidades, CRUE and CSIC) and the COVID-19 program of Pfizer Global Medical Grants, and the research team is made up of different organizations (IECA, Consejería de Salud and Families, Andalusian Health Service and University of Granada), being led by the Andalusian School of Public Health (EASP) and the University of Girona. In addition, the ESSOC has received the approval of the Research Ethics Committee of the Andalusian Regional Government's Ministry of Health and Families and has an Impact Assessment on Data Protection with an acceptable result.

The information requested will be used for the purposes of:

1. Knowing the spatial and temporal distribution of COVID-19 in Andalusia,

2. Analyzing the association between the pandemic and the social, economic and environmental determinants of the Andalusian areas of residence.

3. Knowing the characteristics and evolution of the social, economic, health and well-being impact of COVID-19 in the general Andalusian population.

Linking the information provided by the ESSOC survey with the information requested in this document will provide the extraordinary opportunity to, in addition to exploring and applying powerful and novel methodologies, greatly expand the scope of the global project, since it will jointly use individual information, both clinical and perceived, and contextual, of Andalusia.

The topics, sources and variables requested in the framework of these projects are specified below.

2. Sources of Information

The secondary information sources are briefly described below with the auxiliary variables to be used in the project:

- Longitudinal Database of Health Population (BDLPS, Base de Datos Longitudinal de Andalucía): The consolidated information in BDLPA originates from the integration of information on stocks, flows and variations from the registry coordination system, which together with the data from the Civil Registries referring to births, deaths and marriages and that contained in the population and housing censuses, give rise to a longitudinal integrated framework for population and territory statistics in Andalusia. From this database, the sampling frame for the ESSOC of the general population and disadvantaged areas is extracted as a cross-section with a reference date of January 1 of the most current year possible in each measurement. Therefore, the data necessary for the selection of the person such as name and surname, age and sex are identified; as well as the geographical data of the address to identify the province and the degree of urbanization through the georeferenced coordinates that identify the 250mx250m grid in which geographical and environmental variables are classified. The variables to be extracted from this source are defined in the sections on Location and characteristics of the person and Socioeconomic.
- User Database of the Andalusian Public Health System (BDU, Base de datos de Usuarios): this database contains the identifying data of each user of the Andalusian Public Health System. It is used to obtain the telephone numbers of the people selected in each sample to facilitate the conduct of the surveys. The coverage of the BDU in terms of contact phones for the selected samples is usually higher than 96%, containing, at the end of 2018, almost 8,450,000 people.
- Andalusian Population Health Base (BPS, Base de Datos Poblacional): health information system that collects demographic data, diagnoses, use of health resources and their providers of each of the people who receive health care in the Andalusian Service of Health (SAS). Users and their identification are provided by the BDU. The clinical information and the use of health resources comes from the primary care and hospital care settings. Among the administrative-clinical data sources, the Digital Clinical Record (Diraya) and the hospital CMBD (hospitalization, medical and surgical day hospitals and emergencies) stand out. From the data collected in this database, estimates can be obtained on health, the behavior of users in relation to health services and stratify the population to guide the provision of these services. The BPS also allows longitudinal studies to be carried out, estimating the incidence of pathologies and making projections on the state of the population's health and its resource needs. The variables to be extracted from this source are defined in the sections of COVID-19, Diagnoses, Others (scales), Risk Factors, Hospitalizations and Pharmacy.

- Epidemiological Surveillance System (SVEA, Sistema de Vigilancia Epidemiológica de Andalucía): functional organization for health surveillance that collects, among other things, epidemiological information related to SARS-COV-2 infection. The variables to be extracted from this source are defined in the COVID-19 section.
- Database of the Guadalinfo Network of Andalusia: Guadalinfo is the Andalusian public network of more than 800 centers of digital skills, open innovation and Internet access, co-financed by the Junta de Andalucía and the eight Andalusian provincial councils, and managed by the Fernando de los Ríos Consortium. The centers are located in rural municipalities (less than 20,000 inhabitants) and in certain neighborhoods of larger cities where they serve groups at risk of social exclusion. The objective of Guadalinfo is to train people (using ICT as a tool) so that they are the engine of transformation of their communities: training adapted to different ages and profiles in digital skills, entrepreneurial skills, technological and professional skills, and awareness and community cohesion, with special attention to the prevention of digital, gender and social gaps. This database is used as the ESSOC sampling frame for people over 55 years of age.
- Andalusian Environmental Information Network (REDIAM, Red de medioambiente): Its objective is the integration, standardization and dissemination of all information on the environment generated by all types of environmental information producing centers in Andalusia. Its main functions include coordinating the activities related to environmental information of the Administration of the Junta de Andalucía; promote that the environmental information of Andalusia is integrated into the Environmental Information Infrastructure of Andalusia; develop the technical instructions necessary for the production, organization, management, access and dissemination of information of environmental interest in the autonomous sphere and manage environmental information, in any medium, both for internal use by the Administration of the Junta de Andalucía and for the general public, guaranteeing the effectiveness of the right of access to environmental information; and coordinate and cooperate with other environmental information centers both locally and regionally, nationally or internationally. The variables to be extracted from this source are defined in the Environmental section.

3. Cohort

Objectives 1 and 2:

- Residents in Andalusia with positive COVID-19 through a diagnostic test for active infection.
- Residents in Andalusia without positive COVID-19 and who underwent a diagnostic test for active infection, from the first positive COVID-19 case to the last registered in the previous sample. Sample (control) of 550,000 people matched with COVID-19 positives by sex, age (+/-5 years) and residence (e.g., health area / area).
- Residents in Andalusia without positive COVID-19 and those who did not undergo a diagnostic test for active infection, from the first positive COVID-19 case to the last registered in the first sample. Sample (control) of 550,000 people matched with COVID-19 positives by sex, age (+/-5 years) and residence (e.g., health area / area).

Objectives 2 and 3:

- People who have been interviewed in the ESSOC (effective samples of the four measurements, April 2020 to May 2021).

4. Location and Characteristics of the Person

For each subject:

1. Sex
2. Year of birth or, in any case, the age group of the subject (0 to 15 years, 16 to 24 years, 25 to 34 years, 35 to 44 years, 45 to 54 years, 55 to 64 years, 65 to 74 years, 75 to 84 years and 85 years or more)
3. Census section of the subject's residence address
4. Municipality of the subject's residence address
5. Basic health zones (ZBS) in which it is assigned

5. COVID – 19

For each subject:

From the Andalusian Epidemiological Surveillance System (SVEA):

1. Date and result of the diagnostic test for active infection (PDIA) to detect the presence of active infection by SARSCoV-2 that includes both reverse transcription – polymerase chain reaction (real time RT – PCR) and antigen (Ag) rapid test.
2. Symptoms of COVID-19:
    - o Date of diagnosis (day / month / year)
    - o Body temperature
    - o General condition (normal, mild involvement, severe involvement)
    - o Mental state (normal, altered)
    - o Persistent cough (Yes or no)
    - o Fever (Yes or no)
    - o Dyspnea (No, not serious, serious)
    - o Broken speech (Yes or no)
    - o Pleuritic pain (Yes or no)
    - o Coughing up blood (Yes or no)
    - o Vomiting (Yes or no)
    - o Diarrhea (Yes or no)
    - o Taste / smell disturbances (Yes or no)
    - o Cyanosis (Yes or no)
    - o Chest X-ray (Yes or no)
3. Date of symptom onset.
4. Close contact of case confirmed by PDIA (Yes / No)
5. Local or imported case (Yes / No)
6. Country from which the case comes
7. Health professional (Yes / No) or socio-health (Yes / No)
8. Hospitalization of confirmed Covid-19 cases (Yes / No) and discharge / discharge date (day / month / year).

9. Admission to the ICU of cases confirmed by Covid-19 (Yes / No) and date of entry / exit (day / month / year)
10. Death of confirmed cases by Covid-19 and Date of death (day / month / year)
11. Death from other causes (classified by cause) and date of death (day / month / year)
12. Resident in closed institution (Yes / No)

From the Population Health Base (BPS):

- Need for mechanical ventilation.
- Clinical evolution.
- Clinical presentation (acute respiratory infection, pneumonia, respiratory distress syndrome, other severe respiratory symptoms, acute renal failure).
- Basic disease (diabetes, cardiovascular disease, high blood pressure, COPD, cancer, immunosuppression).
- Risk factors (pregnancy, over 60 years old, others).

From Diraya's COVID-19 Care Questionnaire:

1. Identification data.
2. Type of visit: suspicion, follow-up, national seroprevalence study, asymptomatic screening.
3. Categories and groups of special consideration.
4. Supported symptoms, onset date.
5. PCR positive and first date.
6. Scope of possible exposure, imported case and contact with confirmed case.
7. Isolation.
8. Rapid test, serology and PCR results.
9. Contact study.
10. Information to Public Health or referrer.

6. Diagnostics

For each subject, diagnosis (yes / no), first diagnosis, date of this first diagnosis (day / month / year) of chronic pathologies (permanent and transitory) collected in the Population Health Base (BPS) composed of sets of diagnoses CIE belonging to the following devices or systems:

● Infectious

● Circulatory:

- o Acute cerebrovascular disease
- o Ill-defined cerebrovascular disease and other
- o Transient cerebral ischemia
- o Precerebral artery occlusion or stenosis
- o Sequel to cerebrovascular disease
- o Arteriopathy of extremities
- o Intra-abdominal arteriopathy
- o Aortic, peripheral and visceral arteries aneurysm
- o Atrial fibrillation
- o Ischemic heart disease
- o Acquired valve disease
- o Congenital heart and circulatory abnormality
- o Hypertension
- o Heart failure

● Neoplasms:

- o Head and neck cancer
- o Stomach cancer
- o Colorectal cancer
- o Liver and bile duct cancer
- o Pancreatic cancer
- o Bronchus and lung cancer

- Thyroid cancer
    - Bone and soft tissue cancer
    - Skin melanoma
    - Breast cancer
    - Uterine cancer
    - Cervical cancer
    - Ovarian cancer
    - Prostate cancer
    - Testicular cancer
    - Kidney and renal pelvis cancer
    - Bladder cancer
    - Hodgkin's disease
    - Non-Hodgkin's lymphoma
    - Leukemia
    - Immunoproliferative cancer
    - Kaposi's sarcoma
- Respiratory:
  - COPD
  - asthma
- Endocrine:
    - Diabetes
    - Dyslipidemia
    - Hypothyroidism
    - Obesity
- Digestive:
    - Chronic liver disease except cirrhosis
    - Hepatic steatosis
    - Liver cirrhosis

- o Gastroesophageal reflux disease
- o Regional enteritis and ulcerative colitis
- o Food intolerance and malabsorption syndrome

● Mental Health:

- o dementia
- o Other organic mental disorder
- o Alcohol dependence
- o Tobacco dependence
- o Dependence on other substances
- o Schizophrenic disorder
- o Mood disorder
- o Anxiety disorder
- o Eating behavior disorder
- o Other functional disorder
- o Adult personality and behavior disorder
- o Intellectual disability
- o Autism spectrum disorder
- o Other developmental disorder
- o Childhood adolescent initiation disorder

● Skin:

- o Psoriasis
- o Atopic dermatitis

● Nervous System:

- o Epilepsy
- o Parkinson's disease
- o Extrapyramidal syndrome
- o Neurological disease with non-stroke motor deficit

● Osteomuscular:

- Osteoporosis
- Osteoarthritis, spondylosis
- Gout and other crystal arthropathies
- Other arthropathy
- Rheumatoid arthritis and related diseases
- Collagen disease and vasculitis
- Fibromyalgia

● Eye:

- Age-related macular degeneration
- Glaucoma
- Retinopathy

● Genitourinary:

- Chronic kidney failure
- Urinary lithiasis

7. Other (Scales)

For the following scales, all the values of 2019 and 2020. day / month / year:

- Barthel scale. Daily life activity.
- Degree of severity COPD (i, II, III, IV).
- Coronary risk (REGICOR or other).

Source: BPS, although with low completion and high variability depending on the district.

- Fracture risk index (FRAX).
- Classification of functional capacity Heart failure (NYHA)

8. Risk factors

For each subject, diagnosis (yes / no), discharge date (day / month / year) and discharge date (if applicable) of:

- Obesity diagnosis: Yes or No.
  - CIE-10: E66, E66.0, E66.01, E66.09, E66.1, E66.2. E66.8, E66.9
- Dyslipidemia diagnosis: Yes or No.
  - CIE-10: E78, E78.0, E78.00, E78.01, E78.1, E78.2, E78.3, E78.4, E78.41, E78.49, E78.5, E78.6, E78.7, E78.70, E78.71, E78.72, E78.79, E78.8, E78.81, E78.81, E78.9
- Diagnosis of hypertriglyceridemia: Yes or No.
  - CIE-10: E78.1, E78.2, E78.3
- Diagnosis of hypercholesterolemia: Yes or No.
  - CIE-10: E78.0, E78.00, E78.01
- Diagnosis of smoking: Yes or No, Ex-smoker
  - CIE-10: F17, F17.2, F17.20, F17.200, F17.201, F17.203, F17.208, F17.209, F17.21, F17.210, F17.211, F17.213, F17.218, F17.219, F17.22,F17.220, F17.221, F17.223, F17.228, F17.229, F17.29, F17.290, F17.291, F17.293, F17.298, F17.299
- Diagnosis of alcoholism: Yes or No.
  - CIE-10: F10, F10.1, F10.10, F10.11, F10.12, F10.120, F10.121, F10.129,F10.14, F10.15, F10.150, F10.151, F10.159, F10.18, F10.180, F10.181, F10.182, F10.188, F10.19,F10.2,

F10.20, F10.21, F10.22, F10.220, F10.221, F10.229, F10.23, F10.230, F10.231, F10.232, F10.239, F10.24, F10.25, F10.250, F10.251, F10.259, F10.26, F10.27, F10.28, F10.280, F10.281, F10.282, F10.289, F10.29, F10.9, F10.92, F10.92, F10.920, F10.921, F10.929, F10.94, F10.95, F10.950, F10.951, F10.959, F10.96, F10.97, F10.98, F10.980, F10.981, F10.982, F10.988. F10.99, E52, G62.1, I42.6, K29.2, K70.0, K70.3, K70.9, T51, Z50.2, Z71.4, Z72.1

- Diagnosis of drug abuse: Yes or No.
  - CIE-10: F11, F12, F13, F14, F15, F16, F18, F19, Z71.5, Z72.2

Source: BPS, although mostly poorly completed or poorly collected in alcoholism and smoking.

For the subject of the sample, all the values of, date of the measurement (day / month / year), all the values of 2019 and 2020.

- Fasting venous blood glucose, sugar
- Glycated hemoglobin (HbA1c)
- Total cholesterol
- HDL cholesterol
- LDL cholesterol
- Triglycerides (fasting)
- Glomerular filtration in blood

On the other hand:

- Body mass index
- Systolic blood pressure
- Diastolic blood pressure
- SpO2 (pulse oximetry)

9. Hospitalizations

CMBD records of hospitalization

For each episode:

o Start date / end date

o ABS of origin

o Reason for admission

o Origin of income

o Circumstance of admission

o Provider unit

o Type of destination unit

o Service

o Circumstance of discharge

o Diagnosis - position 1

o Procedures

The CMBD records of emergencies. For each contact:

o Start date / end date

o basic health area of origin

o Reason for urgency

o Urgency initiative

o Level of urgency selection

o Means of arrival to the emergency

o Provider unit

o Type of destination unit

o Service

o Situation of the patient at discharge

Source: BPS.

10. Pharmacy

Pharmacy indicators: Prescription consumed (drugs).

10. Socioeconomic Information

For each subject, discharge date (day / month / year) and discharge date (day / month / year) if it were the case of:

- Diagnosis of "Problems related to education and literacy":
    - CIE-10: Z55
- Diagnosis of "Problems related to work and unemployment":
    - CIE-10: Z56
- Diagnosis of "Occupational exposure to risk factors":
    - CIE-10: Z57
- Diagnosis of "Problems related to economic and housing circumstances":
    - CIE-10: Z59
- Diagnosis of "Problems related to the social environment":
    - CIE-10: Z60
- Diagnosis of "Problems related to education":
    - CIE-10: Z62
- Diagnosis of "Other problems related to the closest environment, including family circumstances":
    - CIE-10: Z63
- Diagnosis of "Problems related to certain psychosocial circumstances":
    - CIE-10: Z64
- Diagnosis of "Problems related to other psychosocial circumstances":
    - CIE-10: Z55

Source: BPS with very low completion.

Contextual variables:

- Unemployment rate by census section. Last year available. The latest information available corresponds to 2011. Source: INE, Population and Housing Census 2011.
- Sub-housing. Percentage of dwellings with less than 45 m2 of habitable area, by census section. Last year available. The latest information available corresponds to 2011. Source: INE, Population and Housing Census 2011.
- Construction typologies of the cadastre (250x250m; available in http://www.juntadeandalucia.es/institutodeestadisticaycartografia/tipologiasconstructivas/index.htm):
    - 011 Collective housing. Urban (250mx250m)

- 012 Single-family homes. Urban (250mx250m)
- 013 Rural building (250mx250m)
- 013 Home garages (250mx250m)
- 022 Other garages and car parks (250mx250m)
- 02 Industrial (250mx250m)
- 03 Offices (250mx250m)
- 04 Trade (250mx250m)
- 05 Sports (250mx250m)
- 05P Pools (250mx250m)
- 06 Shows (250mx250m)
- 07 Hospitality and Leisure (250mx250m)
- 08 Health, welfare and charity (250mx250m)
- 09 Cultural (250mx250m)
- 093 Religious (250mx250m)
- 10 Singular (250mx250m)
- 1034 Golf courses (250mx250m) with indicators such as Collective housing, Single-family housing and Rural building.

- Sociodemographic information at the cell level (available at http://www.juntadeandalucia.es/institutodeestadisticaycartografia/distribucionpob/index.htm; Fichero mallapob.xls). Some available indicators:
  - Registered and unregistered employment, by age group
  - Population by sex
  - Population with contributory pension and amount
  - Population by nationality,
  - Population staying in municipality
  - Population residing in a cell that was born in the same municipality
- Socio-demographic information at the census section level (available at http://www.juntadeandalucia.es/institutodeestadisticaycartografia/poblacion_registros/index.htm).
- Degree of urbanization available in: (http://www.juntadeandalucia.es/institutodeestadisticaycartografia/gradourbanizacion/index.htm).
- Deprivation index of the study census sections (Source: MEDEA III).
- Average income per person (Source: INE. Household income distribution atlas 2020; (Available at: https://www.ine.es/en/experimental/atlas/exp_atlas_tab_en.html)
- Geographical demarcation of disadvantaged areas of Andalusia.

11. Environmental Information

Daily averages, ideally from January 1, 2011 to March 31, 2021 (otherwise, from January 1, 2019 to December 31, 2020) and at the census section level or, if not possible, by collecting / meteorological station :

- Air pollutants:
  - o Particles (PM10, PM2,5)
  - o Nitrogen oxides (NO, NO2)
  - o Sulfur dioxide (SO2)
  - o Carbon monoxide (CO)
  - o Sulfur dioxide (SO2)
  - o Ozone (O3)
  - o Aromatic hydrocarbons (benzene C6H6)
  - o Heavy metals (mercury, lead, arsenic, cadmium, nickel)
- Weather variables:
  - o Temperature (minimum, maximum, average)
  - o Relative humidity
  - o Wind speed at 10 m
  - o Atmospheric pressure
  - o Solar radiation
- Information on the collecting / meteorological station: code, name, municipality, coordinates (UTM or geographical), altitude.

Source:

Andalusian Environmental Information Network (Rediam):

- Downloads:

    - Weather: https://descargasrediam.cica.es/repo/s/RUR?path=%2F04_RECURSOS_NATURALES%2F03_CLIMA
    - Stations: https://descargasrediam.cica.es/repo/s/RUR?path=%2F04_RECURSOS_NATURALES%2F03_CLIMA%2F02_CARACTERIZACION_CLIMATICA
- Environmental Climatology Information Subsystem (CLIMA): http://www.juntadeandalucia.es/medioambiente/servtc5/WebClima/menu_consultas.jsp?b=s
- Air quality:
    - http://www.juntadeandalucia.es/medioambiente/atmosfera/informes_siva/ene21/ca210129.htm
    - Official statistics http://www.juntadeandalucia.es/medioambiente/vem/?c=Menu/tema/582.
- List of Environmental Indicators of Andalusia 2019: http://www.juntadeandalucia.es/medioambiente/site/rediam/menuitem.04dc44281e5d53cf8ca78ca731525ea0/?vgnextoid=efaebb669ed16710VgnVCM100000341de50aRCRD&vgnextchannel=64899dde67bbf310VgnVCM1000001325e50aRCRD&vgnextfmt=rediam&lr=lang_es#apartado3faebb669ed16710VgnVCM100000341de50a
- Impact of COVID and the environment: https://comunidadrediam.cica.es/la-rediam-analiza-las-consecuencias-que-el-confinamiento-por-covid-19-puede-tener-en-el-medio-ambiente

The products available in REDIAM can be consulted at http://www.juntadeandalucia.es/medioambiente/site/rediam/menuitem.aedc2250f6db83cf8ca78ca731525ea0/?vgnextoid=373203d78270f210VgnVCM2000000624e50aRCRD&lr=lang_es

The web services prepared by the Andalusian Environmental Information Network following the interoperability standards approved by the Open Geospatial Consortium (OGC) can be consulted at At

http://www.juntadeandalucia.es/medioambiente/site/rediam/menuitem.aedc2250f6db83cf8ca78ca731525ea0/?vgnextoid=f7db7c119370f210VgnVCM2000000624e50aRCRD&lr=lang_es

- Wastewater: General Directorate of Public Health and Pharmaceutical Management
