# Supplementary material for: Impact of COVID-19 on the Health of the General and More Vulnerable Population and Its Determinants: Health Care and Social Survey–ESSOC, Study Protocol
Source: Int J Environ Res Public Health. 2021 Jul 31;18(15):8120. doi: 10.3390/ijerph18158120 (PMC8345631; doi:10.3390/ijerph18158120)
Supplement: Supplementary file 1 [file ijerph-18-08120-s001.zip › SupplementaryMaterial3_AuxiliaryVariablesSpanish.pdf]

Impact of COVID-19 on the health of the general and more vulnerable population and its determinants: Health care and social survey-ESSOC, study protocol

Especificación general de datos (Registros administrativos)

Encuesta sanitaria y social (ESSOC)

Distribución espaciotemporal y factores socioeconómicos y ambientales de la infección por el SARS-Cov-2

Junta de Andalucía
Consejería de Salud y Familias
Escuela Andaluza de Salud Pública

Índice

1. Introducción 3
2. Fuentes de Información 4
3. Cohorte 6
4. Localización y Características de la Persona 6
5. COVID – 19 7
6. Diagnósticos 9
7. Otras (Escalas) 12
8. Factores de Riesgo 12
9. Hospitalizaciones 13
10. Farmacia 14
11. Información Socioeconómica 15
12. Información Ambiental 17

1. Introducción

Mediante el presente documento se solicitan las variables necesarias para el desarrollo de los proyectos "Encuesta Sanitaria y Social (ESSOC)" y "Distribución espaciotemporal y factores socioeconómicos y ambientales de la infección por el SARS-CoV-2".

Estos proyectos han recibido financiación de las convocatorias competitivas de SUPERA COVID19 (SAUN: Santander Universidades, CRUE y CSIC)[1] y del programa COVID-19 de Pfizer Global Medical Grants[2], y el equipo de investigación está compuesto por diferentes organizaciones (IECA, Consejería de Salud y Familias, Servicio Andaluz de Salud y Universidad de Granada), siendo liderados por la Escuela Andaluza de Salud Pública (EASP) y la Universidad de Girona. Además, la ESSOC ha recibido la aprobación del Comité Ético de Investigación de la Consejería de Salud[3] y Familias de la Junta de Andalucía y cuenta con una Evaluación de Impacto en Protección de Datos con resultado aceptable[4].

La información solicitada se utilizará con los objetivos de:

1. Conocer la distribución espacial y temporal de la COVID-19 en Andalucía,
2. Analizar la asociación entre la pandemia y los determinantes sociales, económicos y ambientales de las áreas andaluzas de residencia.
3. Conocer las características y evolución del impacto social, económico, en la salud y el bienestar de la COVID-19 en la población general andaluza.

La vinculación de información proporcionada por la encuesta ESSOC con la información solicitada en este documento brindará la extraordinaria oportunidad de, además de explorar y aplicar metodologías potentes y novedosas, ampliar enormemente el alcance del proyecto global, ya que utilizará conjuntamente información individual, tanto clínica como percibida, y contextual de Andalucía.

A continuación, se especifican las temáticas, fuentes y variables solicitadas en el marco de esos proyectos.

[1] Protocolo disponible en https://drive.google.com/file/d/1cFmX0B4VY35-EL6ETwddbYWBLy2uunmG/view?usp=sharing
[2] Protocolo disponible en https://drive.google.com/file/d/1Jiq4fUUpvlEjSdDeplYCngWj3fLuWB15/view?usp=sharing
[3] Certificado disponible en: https://drive.google.com/file/d/1qQ5LUv4_mvrzaNSFKjsVQTGu4Iy4jvpB/view?usp=sharing
[4] Protocolo disponible en: https://drive.google.com/file/d/1KbyhT0qWvg38KTHpkUNLaeysz5Bniw5K/view?usp=sharing

2. Fuentes de Información

A continuación, se describen brevemente las fuentes de información secundarias con las variables auxiliares a utilizar en el proyecto:

- Base de Datos Longitudinal de Población en Salud (BDLPS)[5]: La información consolidada en BDLPA se origina a partir de la integración de información de stocks, flujos y variaciones procedente del sistema de coordinación padronal, que junto con los datos provenientes de los Registros Civiles referidos a nacimientos, defunciones y matrimonios y la contenida en los censos de población y viviendas, dan lugar a un marco integrado longitudinal para las estadísticas de población y territorio en Andalucía. De esta base de datos se extrae el marco muestral para la ESSOC de población general y de zonas desfavorecidas como corte transversal con fecha de referencia 1 de enero del año más actual posible en cada medición. Se identifica, por tanto, los datos necesarios para la selección de la persona como nombre y apellidos, edad y sexo; así como los datos geográficos del domicilio para identificar la provincia y el grado de urbanización a través de las coordenadas georreferenciadas que identifican la cuadrícula de 250mx250m en la que se clasifican variables geográficas y ambientales. Las variables a extraer de esta fuente se encuentran definidas en los apartados de Localización y características de la persona y de Socioeconómicas.
- Base de Datos de Usuario del Sistema Sanitario Público de Andalucía (BDU)[6]: esta base de datos contiene los datos identificativos de cada usuario del Sistema Sanitario Público de Andalucía (SSPA). Se utiliza para obtener los números de teléfono de las personas seleccionadas en cada muestra que facilite la realización de las encuestas. La cobertura de la BDU en términos de teléfonos de contacto para las muestras seleccionadas es habitualmente superior al 96% conteniendo, al final de 2018, casi 8.450.000 personas.
- Base Poblacional de Salud de Andalucía (BPS)[7]: sistema de información sanitaria que recoge datos demográficos, diagnósticos, de utilización de los recursos sanitarios y de los proveedores de los mismos de cada una de las personas que reciben asistencia sanitaria en el Servicio Andaluz de Salud (SAS). Los usuarios y su identificación los proporciona la BDU. La información clínica y de utilización de los recursos sanitarios proviene de los ámbitos asistenciales de atención primaria y hospitalaria. Entre las fuentes de datos administrativo-clínicas destacan la Historia Clínica Digital (Diraya) y los CMBD hospitalarios (hospitalización, hospitales de día médico y quirúrgico y urgencias). A partir de los datos que se recogen en esta base se pueden obtener estimaciones sobre la salud, el comportamiento de las personas usuarias en relación con los servicios sanitarios

[5] Más información en: https://juntadeandalucia.es/protecciondedatos/detalle/187464.html
[6] Más información en: https://web.sas.junta-andalucia.es/servicioandaluzdesalud/archivo-estadisticas/base-de-datos-de-personas-usuarias-bdu-2
[7] Más información en: https://web.sas.junta-andalucia.es/servicioandaluzdesalud/profesionales/sistemas-de-informacion/base-poblacional-de-salud

y estratificar la población para orientar la prestación de estos servicios. La BPS, además, permite realizar estudios longitudinales, estimar la incidencia de las patologías y realizar proyecciones sobre el estado de la salud de la población y sus necesidades de recursos. Las variables a extraer de esta fuente se encuentran definidas en los apartados de COVID-19, Diagnósticos, Otras (escalas), Factores de Riesgo, Hospitalizaciones y de Farmacia.

- Sistema de Vigilancia Epidemiológica (SVEA)[8]: organización funcional para la vigilancia de la salud que recoge, entre otra, información epidemiológica relacionada con la infección por SARS-COV-2. Las variables a extraer de esta fuente se encuentran definidas en el apartado de COVID-19.
- Base de Datos de la Red Guadalinfo de Andalucía[9]: Guadalinfo es la red pública andaluza de más de 800 centros de competencias digitales, innovación abierta y acceso a Internet, cofinanciada por la Junta de Andalucía y las ocho diputaciones provinciales andaluzas, y gestionada por el Consorcio Fernando de los Ríos. Los centros se ubican en municipios rurales (menos de 20.000 habitantes) y en determinados barrios de ciudades mayores donde atienden a colectivos en riesgo de exclusión social. El objetivo de Guadalinfo es capacitar a las personas (usando las TIC como herramienta) para que sean ellas el motor de transformación de sus comunidades: capacitación adaptada a diferentes edades y perfiles en competencias digitales, competencias de emprendimiento, destrezas tecnológicas y profesionales, y sensibilización y cohesión comunitaria, con especial atención a la prevención de brechas digitales, de género y sociales. Esta base de datos se utiliza como marco muestral de la ESSOC a mayores de 55 años.
- Red de Información Ambiental de Andalucía (REDIAM)[10]: tiene como objeto la integración, normalización y difusión de toda la información sobre el medio ambiente generada por todo tipo de centros productores de información ambiental en Andalucía. Entre sus funciones principales se encuentran: coordinar las actividades relativas a la información ambiental de la Administración de la Junta de Andalucía; impulsar que la información ambiental de Andalucía se integre en la Infraestructura de Información Ambiental de Andalucía; desarrollar las instrucciones técnicas necesarias para la producción, organización, gestión, acceso y difusión de la información de interés ambiental en el ámbito autonómico y gestionar la información ambiental, en cualquier soporte, tanto para uso interno de la Administración de la Junta de Andalucía como para el público en general, garantizando la efectividad del derecho de acceso a la información ambiental; y coordinarse y cooperar con otros centros de información ambiental tanto de ámbito local como autonómico, estatal o internacional. Las variables a extraer de esta fuente se encuentran definidas en el apartado de Ambientales.

[8] https://juntadeandalucia.es/organismos/saludyfamilias/areas/salud-vida/vigilancia/paginas/vigilancia-transmisibles.html
[9] http://www.guadalinfo.es/
[10] http://www.cma.junta-andalucia.es/medioambiente/site/rediam/menuitem.aedc2250f6db83cf8ca78ca731525ea0/?vgnextoid=c9587d087270f210VgnVCM1000001325e50aRCRD&lr=lang_es

3. Cohorte

Objetivos 1 y 2:

- Residentes en Andalucía con positivo COVID-19 a través de una prueba diagnóstica de infección activa.
- Residentes en Andalucía sin positivo COVID-19 y a los que se les realizó una prueba diagnóstica de infección activa, desde el primer caso positivo COVID-19 hasta el último registrado en la muestra anterior. Muestra (control) de 550.000 personas emparejadas con la de positivos COVID-19 por sexo, edad (+/- 5 años) y residencia (p.ej. zona/área de salud).
- Residentes en Andalucía sin positivo COVID-19 y a los que no se les realizó prueba diagnóstica de infección activa, desde el primer caso positivo COVID-19 hasta el último registrado en la muestra primera. Muestra (control) de 550.000 personas emparejadas con la de positivos COVID-19 por sexo, edad (+/- 5 años) y residencia (p.ej. zona/área de salud).

Objetivos 2 y 3:

- Personas que han sido entrevistadas en la ESSOC (muestras efectivas de las cuatro mediciones, abril 2020 a mayo 2021).

4. Localización y Características de la Persona

Para cada sujeto

1. Sexo
2. Año de nacimiento o, en todo caso, el grupo de edad del sujeto (0 a 15 años, 16 a 24 años, 25 a 34 años, 35 a 44 años, 45 a 54 años, 55 a 64 años, 65 a 74 años, 75 a 84 años y 85 años o más)
3. Sección censal del domicilio de residencia del sujeto
4. Municipio del domicilio de residencia del sujeto
5. Zonas básicas de salud (ZBS) en la que está asignado

5. COVID – 19

Para cada sujeto desde el primer caso hasta el más reciente posible.

A partir del Sistema de Vigilancia Epidemiológica de Andalucía (SVEA):

1. Fecha y resultado de la prueba diagnóstica de infección activa (PDIA) para detectar la presencia de infección activa por el SARSCoV-2 que incluye tanto reverse transcription–polymerase chain reaction (real time RT–PCR) como antigen (Ag) rapid test.
2. Síntomas de la COVID-19:
    - o Fecha del diagnóstico (día / mes / año)
    - o Temperatura corporal
    - o Estado general (normal, afectación leve, afectación grave)
    - o Estado mental (normal, alterado)
    - o Tos persistente (Si o no)
    - o Fiebre (Si o no)
    - o Disnea (No, no grave, grave)
    - o Habla entrecortada (Si o no)
    - o Dolor pleurítico (Si o no)
    - o Expectoración con sangre (Si o no)
    - o Vómitos (Si o no)
    - o Diarrea (Si o no)
    - o Alteraciones del gusto / olfato (Si o no)
    - o Cianosis (Si o no)
    - o Radiografía de tórax (Si o no)
3. Fecha de inicio de síntomas.
4. Contacto estrecho de caso confirmado por PDIA (Sí/No)
5. Caso local o importado (Sí/No)
6. País del que procede el caso
7. Profesional sanitario (Sí/No) o sociosanitario (Sí/No)
8. Hospitalización de casos confirmados por Covid-19 (Sí/No) y fecha de alta/baja (día/mes/año).

9. Ingreso en la UCI de casos confirmados por Covid-19 (Sí/No) y fecha de ingreso/salida (día/mes/año)
10. Muerte de casos confirmados por Covid-19 y Fecha de la muerte (día / mes / año)
11. Muerte por otras causas (clasificadas por causa) y fecha de la muerte (día / mes / año)
12. Residente en institución cerrada (Sí/No)

A partir de la Base Poblacional de Salud (BPS):

- Necesidad de ventilación mecánica.
- Evolución clínica.
- Presentación clínica (Infección respiratoria aguda, Neumonía, Síndrome distress respiratorio, otros cuadros respiratorios graves, fallo renal agudo).
- Enfermedad de base (diabetes, enfermedad cardiovascular, hipertensión arterial, EPOC, cáncer, inmunosupresión).
- Factores de riesgo (embarazo, mayor 60 años, otros).

A partir del Cuestionario de atención COVID-19 de Diraya:

1. Datos de identificación.
2. Tipo de visita: sospecha, seguimiento, estudio nacional seroprevalencia, cribado asintomáticos.
3. Categorías y grupos de especial consideración.
4. Síntomas compatibles, fecha de inicio.
5. PCR positiva y fecha primera.
6. Ámbito de posible exposición, caso importado y contacto con caso confirmado.
7. Aislamiento.
8. Resultados de test rápidos, de serología y PCR.
9. Estudio de contacto.
10. Información a Salud Pública o referente.

6. Diagnósticos

Para cada sujeto, diagnóstico (sí / no), primer diagnóstico, fecha de este primer diagnóstico (día / mes / año) de las patologías crónicas (permanentes y transitorias) recogidas en la Base Poblacional de Salud (BPS) compuestas por conjuntos de diagnósticos CIE que pertenecen a los siguientes aparatos o sistemas[11]:

- Infeccioso
- Circulatorio:
  - Enfermedad cerebrovascular aguda
  - Enfermedad cerebrovascular mal definida y otra
  - Isquemia cerebral transitoria
  - Oclusión o estenosis arterias precerebrales
  - Secuela enfermedad cerebrovascular
  - Arteriopatía de extremidades
  - Arteriopatía intraabdominal
  - Aneurisma arterias aorta, periféricas y viscerales
  - Fibrilación auricular
  - Cardiopatía isquémica
  - Enfermedad valvular adquirida
  - Anomalía congénita cardíaca y circulatoria
  - Hipertensión
  - Insuficiencia cardiaca
- Neoplasias:
  - Cáncer de cabeza y cuello
  - Cáncer de estómago
  - Cáncer colorrectal
  - Cáncer de hígado y vías biliares
  - Cáncer de páncreas
  - Cáncer de bronquio y pulmón
  - Cáncer de tiroides
  - Cáncer de hueso y tejidos blandos
  - Melanoma de piel
  - Cáncer de mama

[11] Metodología disponible en: https://www.sspa.juntadeandalucia.es/servicioandaluzdesalud/sites/default/files/sincfiles/wsas-media-mediafile_sasdocumento/2019/patologias_bps.pdf

    - Cáncer de útero
    - Cáncer de cuello uterino
    - Cáncer de ovario
    - Cáncer de próstata
    - Cáncer de testículo
    - Cáncer de riñón y pelvis renal
    - Cáncer de vejiga
    - Enfermedad de Hodgkin
    - Linfoma no Hodgkin
    - Leucemia
    - Cáncer inmunoproliferativo
    - Sarcoma de Kaposi
- Respiratorio:
    - EPOC
    - Asma
- Endocrino:
    - Diabetes
    - Dislipemia
    - Hipotiroidismo
    - Obesidad
- Digestivo:
    - Hepatopatía crónica excepto cirrosis
    - Esteatosis hepática
    - Cirrosis hepática
    - Enfermedad por reflujo gastroesofágico
    - Enteritis regional y colitis ulcerosa
    - Síndrome de malabsorción e intolerancia alimenticia
- Salud Mental:
    - Demencia
    - Otro trastorno mental orgánico
    - Dependencia alcohol
    - Dependencia tabaco
    - Dependencia otras sustancias
    - Trastorno esquizofrénico
    - Trastorno estado animo
    - Trastorno de ansiedad

  - Trastorno conducta alimentaria
  - Otro trastorno funcional
  - Trastorno personalidad y comportamiento adulto
  - Discapacidad intelectual
  - Trastorno del espectro autista
  - Otro trastorno del desarrollo
  - Trastorno inicio infancia adolescencia
- Piel:
  - Psoriasis
  - Dermatitis atópica
- Sistema Nervioso:
  - Epilepsia
  - Enfermedad de Parkinson
  - Síndrome extrapiramidal
  - Enfermedad neurológica con déficit motor no ACV
- Osteomuscular:
  - Osteoporosis
  - Artrosis, espondilosis
  - Gota y otras artropatías por cristales
  - Otra artropatía
  - Artritis reumatoide y enfermedades relacionadas
  - Enfermedad del colágeno y vasculitis
  - Fibromialgia
- Ojo:
  - Degeneración macular asociada a la edad
  - Glaucoma
  - Retinopatía
- Genitourinario:
  - Insuficiencia renal crónica
  - Litiasis urinaria

7. Otras (Escalas)

Para las siguientes escalas, todos los valores del 2019 y 2020. día/mes/año:

- Escala de Barthel. Actividad vida diaria.
- Grado Gravedad EPOC (i, II, III, IV).
- Riesgo coronario (REGICOR u otra).

Fuente: BPS, aunque con baja cumplimentación y alta variabilidad en función del distrito.

- Índice de riesgo de fracturas (FRAX).
- Clasificación Capacidad funcional Insuficiencia cardíaca (NYHA)

8. Factores de Riesgo

Para cada sujeto, diagnóstico (sí / no), fecha de alta (día / mes / año) y fecha de baja (si fuese el caso) de:

- Diagnosis de obesidad: Sí o No.
    - CIE-10: E66, E66.0, E66.01, E66.09, E66.1, E66.2. E66.8, E66.9
- Diagnosis de dislipemia: Sí o No.
    - CIE-10: E78, E78.0, E78.00, E78.01, E78.1, E78.2, E78.3, E78.4, E78.41, E78.49, E78.5, E78.6, E78.7, E78.70, E78.71, E78.72, E78.79, E78.8, E78.81, E78.81, E78.9
- Diagnosis de hipertrigliceridemia: Sí o No.
    - CIE-10: E78.1, E78.2, E78.3
- Diagnosis de hipercolesterolemia: Sí o No.
    - CIE-10: E78.0, E78.00, E78.01
- Diagnosis de tabaquismo: Sí o No, Exfumador.
    - CIE-10: F17, F17.2, F17.20, F17.200, F17.201, F17.203, F17.208, F17.209, F17.21, F17.210, F17.211, F17.213, F17.218, F17.219, F17.22,F17.220, F17.221, F17.223, F17.228, F17.229, F17.29, F17.290, F17.291, F17.293, F17.298, F17.299
- Diagnosis de alcoholismo: Sí o No.
    - CIE-10: F10, F10.1, F10.10, F10.11, F10.12, F10.120, F10.121, F10.129,F10.14, F10.15, F10.150, F10.151, F10.159, F10.18, F10.180, F10.181, F10.182, F10.188, F10.19,F10.2, F10.20, F10.21, F10.22, F10.220, F10.221, F10.229, F10.23, F10.230, F10.231, F10.232, F10.239, F10.24, F10.25, F10.250, F10.251, F10.259, F10.26, F10.27, F10.28, F10.280, F10.281, F10.282, F10.289, F10.29, F10.9, F10.92, F10.92, F10.920, F10.921, F10.929,

F10.94, F10.95, F10.950, F10.951, F10.959, F10.96, F10.97, F10.98, F10.980, F10.981, F10.982, F10.988. F10.99, E52, G62.1, I42.6, K29.2, K70.0, K70.3, K70.9, T51, Z50.2, Z71.4, Z72.1

- Diagnosis de abusos de drogas: Sí o No.
    - CIE-10: F11, F12, F13, F14, F15, F16, F18, F19, Z71.5, Z72.2

Fuente: BPS, aunque con baja cumplimentación en lo mayoría o mal recogido en alcoholismo y tabaquismo.

Para sujeto de la muestra todos los valores de, fecha de la medida (día/mes/año), todos los valores del 2019 y 2020.

- Glucemia venosa ayunas, azúcar
- Hemoglobina glucosilada (HbA1c)
- Colesterol total
- HDL colesterol
- LDL colesterol
- Triglicéridos (ayunas)
- Filtrado glomerular en sangre

Por otro lado:

- Índice de masa corporal
- Presión arterial sistólica
- Presión arterial diastólica
- SpO2 (pulsioximetría)

9. Hospitalizaciones

Los registros de CMBD de hospitalización

- Para cada episodio:
    - Fecha inicio / fecha de fin
    - ABS de procedencia
    - Motivo del ingreso
    - Procedencia del ingreso
    - Circunstancia del ingreso
    - Unidad proveedora
    - Tipo unidad de destino
    - Servicio

- Circunstancia del alta
- Diagnóstico - posición 1
- Procedimientos

Los registros de CMBD de urgencias

- Para cada contacto:
    - Fecha inicio / fecha de fin
    - ABS de procedencia
    - Motivo de la urgencia
    - Iniciativa de la urgencia
    - Nivel de selección de la urgencia
    - Medio de llegada a la urgencia
    - Unidad proveedora
    - Tipo unidad de destino
    - Servicio
    - Situación del enfermo al alta
    - UP procedencia
    - Tipo UP procedencia
    - UP destino
    - Tipo UP destino

Fuente: BPS.

10. Farmacia

Los indicadores de farmacia:

- Prescripción consumida: fármacos.

11. Información Socioeconómica

Para cada sujeto, fecha de alta (día / mes / año) y fecha de baja (día/mes/año) si fuese el caso de:

- Diagnosis de "Problemas relacionados con la educación y la alfabetización'':
  - CIE-10: Z55
- Diagnosis de "Problemas relacionados con el trabajo y el paro ":
  - CIE-10: Z56
- Diagnosis de "Exposición por causa profesional a factores de riesgo":
  - CIE-10: Z57
- Diagnosis de "Problemas relacionados con circunstancias económicas y de vivienda":
  - CIE-10: Z59
- Diagnosis de "Problemas relacionados con el entorno social ":
  - CIE-10: Z60
- Diagnosis de "Problemas relacionados con la educación":
  - CIE-10: Z62
- Diagnosis de "Otros problemas relacionados con el entorno más cercano, incluyendo circunstancias familiares":
  - CIE-10: Z63
- Diagnosis de "Problemas relacionados con determinadas circunstancias psicosociales":
  - CIE-10: Z64
- Diagnosis de "Problemas relacionados con otras circunstancias psicosociales":
  - CIE-10: Z55

Fuente: BPS con muy baja cumplimentación.

Variables contextuales:

- Tasa de paro por sección censal. Último año disponible. La última información disponible corresponde a 2011. Fuente: INE, Censo de Poblaciones y Viviendas 2011.
- Infraviviendas. Porcentaje de viviendas de menos de 45 m^2 de área habitable, por sección censal. Último año disponible. La última información disponible corresponde a 2011. Fuente: INE, Censo de Poblaciones y Viviendas 2011.
- Tipologías constructivas del catastro (250x250; disponible en http://www.juntadeandalucia.es/institutodeestadisticaycartografia/tipologiasconstructivas/index.htm):
  - 011 Viviendas colectivas. Urbano (250mx250m)
  - 012 Viviendas unifamiliares. Urbano (250mx250m)

    - 013 Edificación rural (250mx250m)
    - 013 Garajes viviendas (250mx250m)
    - 022 Otros garajes y aparcamientos (250mx250m)
    - 02 Industrial (250mx250m)
    - 03 Oficinas (250mx250m)
    - 04 Comercio (250mx250m)
    - 05 Deportes (250mx250m)
    - 05P Piscinas (250mx250m)
    - 06 Espectáculos (250mx250m)
    - 07 Hostelería y Ocio (250mx250m)
    - 08 Sanitarios, asistenciales y benéficos (250mx250m)
    - 09 Culturales (250mx250m)
    - 093 Religiosos (250mx250m)
    - 10 Singulares (250mx250m)
    - 1034 Campos de golf (250mx250m) con indicadores como Viviendas colectivas, Viviendas unifamiliares y Edificación rural.
- Información sociodemográfica a nivel de celda (disponible en http://www.juntadeandalucia.es/institutodeestadisticaycartografia/distribucionpob/index.htm; Fichero mallapob.xls). Algunos indicadores disponibles:
    - Empleo registrado y no registrado, por grupos de edad
    - Población por sexo
    - Población con pensión contributiva e importe
    - Población por nacionalidad,
    - Población permanencia en municipio
    - Población residente en una celda que nació en ese mismo municipio
- Información sociodemográfica a nivel de sección censal (disponible en http://www.juntadeandalucia.es/institutodeestadisticaycartografia/poblacion_registros/index.htm).
- Grado de urbanización disponible en: (http://www.juntadeandalucia.es/institutodeestadisticaycartografia/gradourbanizacion/index.htm).
- Índice de privación de las secciones censales de estudio (Fuente: MEDEA III).
- Renta media por persona y renta media por hogar, ambas por secciones censales (Fuente: INE. Atlas de distribución de renta de los hogares 2021; Disponible en: (https://www.ine.es/experimental/atlas/exp_atlas_tab.htm)

- Índice de Gini por sección censal (Fuente: INE. Atlas de distribución de renta de los hogares 2021; Disponible en: (https://www.ine.es/experimental/atlas/exp_atlas_tab.htm)
- Distribución de la renta p80/p20 por sección censal (Fuente: INE. Atlas de distribución de renta de los hogares 2021; Disponible en: (https://www.ine.es/experimental/atlas/exp_atlas_tab.htm)
- Demarcación geográfica de las zonas desfavorecidas de Andalucía.

12. Información Ambiental

Promedios diarios, idealmente desde 1 de enero de 2011 hasta 31 de marzo de 2021 (en otro caso desde 1 de enero de 2019 a 31 de diciembre de 2020) y a nivel de sección censal o, si no fuera posible, por estación captadora/meteorológica:

- Contaminantes atmosféricos:
  - Partículas (PM_{10}, $PM_{2,5}$)
  - Óxidos de nitrógeno (NO, NO_2)
  - Dióxido de azufre (SO_2)
  - Monóxido de carbono (CO)
  - Dióxido de azufre (SO_2)
  - Ozono (O_3)
  - Hidrocarburos aromáticos (benceno C_6H_6)
  - Metales pesados (mercurio, plomo, arsénico, cadmio, níquel)
- Variables meteorológicas
  - Temperatura (mínima, máxima, media)
  - Humedad relativa
  - Velocidad del viento a los 10 m
  - Presión atmosférica
  - Radiación solar
- Información sobre la estación captadora/meteorológica: código, nombre, municipio, coordenadas (UTM o geográficas), altitud.

Fuente:

Red de Información Ambiental de Andalucía (Rediam):

- Descargas:

    - Clima: https://descargasrediam.cica.es/repo/s/RUR?path=%2F04_RECURSOS_NATURALES%2F03_CLIMA
    - Estaciones: https://descargasrediam.cica.es/repo/s/RUR?path=%2F04_RECURSOS_NATURALES%2F03_CLIMA%2F02_CARACTERIZACION_CLIMATICA
- Subsistema de información de Climatología Ambiental (CLIMA): http://www.juntadeandalucia.es/medioambiente/servtc5/WebClima/menu_consultas.jsp?b=s
- Calidad del aire:
    - Crudos http://www.juntadeandalucia.es/medioambiente/atmosfera/informes_siva/ene21/ca210129.htm
    - Estadísticas oficiales depuradas http://www.juntadeandalucia.es/medioambiente/vem/?c=Menu/tema/582 no automatizada.
- Relación de Indicadores Ambientales de Andalucía 2019: http://www.juntadeandalucia.es/medioambiente/site/rediam/menuitem.04dc44281e5d53cf8ca78ca731525ea0/?vgnextoid=efaebb669ed16710VgnVCM100000341de50aRCRD&vgnextchannel=64899dde67bbf310VgnVCM1000001325e50aRCRD&vgnextfmt=rediam&lr=lang_es#apartado3faebb669ed16710VgnVCM100000341de50a
- Impacto de la COVID y medioambiente: https://comunidadrediam.cica.es/la-rediam-analiza-las-consecuencias-que-el-confinamiento-por-covid-19-puede-tener-en-el-medio-ambiente

Los productos disponibles en REDIAM se pueden consultar en http://www.juntadeandalucia.es/medioambiente/site/rediam/menuitem.aedc2250f6db83cf8ca78ca731525ea0/?vgnextoid=373203d78270f210VgnVCM2000000624e50aRCRD&lr=lang_es

En

http://www.juntadeandalucia.es/medioambiente/site/rediam/menuitem.aedc2250f6db83cf8ca78ca731525ea0/?vgnextoid=f7db7c119370f210VgnVCM2000000624e50aRCRD&lr=lang_es

se pueden consultar los servicios web elaborados por la Red de Información Ambiental de Andalucía siguiendo los estándares de interoperabilidad aprobados por el Open Geospatial Consortium (OGC)

- Aguas residuales: Dirección General de Salud Pública y Ordenación Farmacéutica
